# Health outcomes associated with micronutrient-fortified complementary foods in infants and young children aged 6–23 months: a systematic review and meta-analysis

**DOI:** 10.1016/S2352-4642(22)00147-X

**Published:** 2022-08

**Authors:** Ildikó Csölle, Regina Felső, Éva Szabó, Maria-Inti Metzendorf, Lukas Schwingshackl, Tamás Ferenci, Szimonetta Lohner

**Affiliations:** a Department of Paediatrics, University of Pécs, Pécs, Hungary; b Cochrane Hungary, University of Pécs, Pécs, Hungary; c Clinical Centre, Department of Biochemistry and Medical Chemistry, University of Pécs, Pécs, Hungary; d Department of Public Health Medicine, University of Pécs, Pécs, Hungary; e Medical School, Szentágothai Research Centre, University of Pécs, Pécs, Hungary; f Cochrane Metabolic and Endocrine Disorders Group, Medical Faculty, Heinrich Heine University Düsseldorf, Düsseldorf, Germany; g Institute for Evidence in Medicine, Medical Center, University of Freiburg, Faculty of Medicine, University of Freiburg, Freiburg, Germany; h Physiological Controls Research Center, Obuda University, Budapest, Hungary; i Department of Statistics, Corvinus University of Budapest, Budapest, Hungary

## Abstract

**Background:**

Appropriate feeding of infants and young children is essential for healthy growth and the prevention of stunting, wasting, and overweight. We aimed to assess the beneficial versus harmful effects of providing fortified complementary foods to children in the complementary feeding period.

**Methods:**

In this systematic review and meta-analysis, we searched the databases Cochrane Central Register of Controlled Trials, MEDLINE, Embase, Cumulative Index to Nursing and Allied Health Literature, Global Index Medicus, Web of Science, ClinicalTrials.gov, and WHO International Clinical Trials Registry Platform from inception to March 9, 2021. We included randomised controlled trials and controlled clinical trials done in infants and children aged 6–23 months with no identified health problems. Consumption of foods fortified centrally (ie, during industrial processing) with one micronutrient or a combination of vitamins, minerals, or both was compared with the same complementary foods, but without micronutrient fortification. Two review authors independently screened studies for eligibility, extracted data, assessed risk of bias, and rated the certainty of the evidence. The main outcomes were growth (measured by Z scores for weight for age, weight for height or length, and height or length for age, or other growth measures), stunting, wasting, nutrient adequacy or excess, anaemia, haemoglobin concentration, iron status, serum zinc concentration, and serum retinol concentration. We used a random-effects meta-analysis for combining data. This study is registered with PROSPERO, CRD42021245876.

**Findings:**

We included 16 studies with 6423 participants, 13 of which were done in malaria-endemic areas. Overall, 12 studies were included in the quantitative syntheses. We identified five further ongoing studies. There was no difference between participants who received fortified complementary foods and those who received non-fortified complementary foods in weight-for-age Z scores (mean difference −0·01, 95% CI −0·07 to 0·06; five trials; 1206 participants; moderate-certainty evidence), weight-for-height or length Z scores (−0·05, −0·19 to 0·10; four trials; 1109 participants; moderate-certainty evidence), and height or length-for-age Z scores (−0·01, −0·21 to 0·20; four trials; 811 participants; low-certainty evidence); stunting and wasting were not assessed in any study as outcomes. Moderate-certainty evidence from six trials with 1209 patients showed that providing fortified complementary foods to children aged 6–23 months reduced the risk of anaemia (risk ratio 0·57, 95% CI 0·39 to 0·82). Those who received fortified complementary foods compared with those who did not had higher haemoglobin concentrations (mean difference 3·44 g/L, 95% CI 1·33 to 5·55; 11 trials; 2175 participants; moderate-certainty evidence) and ferritin concentration (0·43 μg/L on log scale, 0·14 to 0·72; six trials; 903 participants; low-certainty evidence). The intervention led to no effects on serum zinc concentration (−0·13 g/dL, −0·82 to 0·56; two trials; 333 participants; low-certainty evidence) and serum retinol concentration (0·03 μmol/L, −0·02 to 0·08; five trials; 475 participants; moderate-certainty evidence).

**Interpretation:**

Fortified complementary foods are effective strategies to prevent anaemia in infants and young children aged 6–23 months in malaria-endemic regions. Effects of complementary food fortification should be further investigated in low-income and middle-income countries, but should also be assessed in high-income countries, and in regions where malaria is not endemic.

**Funding:**

WHO.

## Introduction

Complementary feeding is the transition from exclusive breastfeeding to family foods, typically from 6 months to 23 months of age.^1^ This period is crucial for physical, cognitive, and motor development, and during this time infants and young children need a great dietary diversity to ensure their nutrient needs are met.^2^

The low mineral content of breast milk, the relatively small amount of complementary foods consumed, their potentially inadequate nutrient density, low consumption of haem iron-containing meat, and the relatively high requirement of micronutrients can lead to nutrient deficiencies during this period of growth.^3^ Micronutrient deficiencies are common in low-income and middle-income countries, affecting more than 2 billion people worldwide.^4^, ^5^ The highest nutrient gaps in the complementary feeding period have been described for iron, vitamin A, vitamin B12, zinc, and calcium.^4^, ^5^

**Research in context**

**Evidence before this study**

Fortified complementary foods are processed foods enriched with essential micronutrients, with the aim of preventing or correcting deficiency of one or more micronutrients in the critical period of complementary feeding. They provide an alternative to home or point-of-use fortification with micronutrient powders or crushable or soluble micronutrient tablets, which were shown in recent systematic reviews to be effective tools to reduce anaemia in children in the complementary feeding period in low-income and middle-income countries. We did a preliminary search of PubMed, Embase, and the Cochrane Library for existing systematic reviews and meta-analyses evaluating the health impact of fortified complementary foods in infants and young children aged 6–23 months using the search terms “fortified” OR “fortification” AND “complementary” published up to Jan 15, 2021. No language restriction was applied. The preliminary investigation revealed systematic reviews assessing the effects of other existing nutritional strategies, including interventions with small-quantity lipid-based nutrient supplements, fortified milk, and home fortification with micronutrient powders. Some systematic reviews included a wide range of interventions, including industrially fortified foods. However, these reviews were narrow in terms of investigated outcomes and described studies from 2006 to 2014. The present systematic review and meta-analysis was done to address the existing gap and to inform the process of updating WHO guidance on feeding of infants and young children aged 6–23 months.

**Added value of this study**

To our knowledge, this is the first systematic review summarising evidence on the consumption of fortified complementary foods compared with the unfortified version of the same complementary foods in infants and young children aged 6–23 months. We showed that fortified complementary foods probably reduce anaemia in infants and young children in malaria-endemic regions. We showed that fortification with the applied micronutrient composition and doses probably makes little or no difference to growth outcomes. However, the diversity of foods fortified and the micronutrients used for fortification, and the differences in micronutrient doses used for fortification and in baseline characteristics of children, including their anaemia and malaria status, limit our ability to understand how, when, and where this intervention is the most effective and safe.

**Implications of all the available evidence**

Our study suggests that interventions with fortified complementary foods are effective strategies to prevent anaemia in infants and young children aged 6 months to 2 years in malaria-endemic regions. Our findings support the need for further studies investigating health-related outcomes associated with fortified complementary foods in regions where malaria is not endemic and in high-income countries. More evidence is needed to better understand what level of fortification can lead to adequate or excess nutrient intake and related safety issues. It should be further investigated whether these products lead to the displacement of other foods and how they affect vitamin and mineral status, stunting, and wasting.

Several strategies have been proposed to provide target nutrients to infants and young children. These strategies include, besides diversified diets, fortified complementary foods, fortified animal milk,^6^ micronutrient powders,^7^ and small quantities of lipid-based nutrient supplements.^8^

Food fortification is defined as the addition of micronutrients to processed foods with the aim to increase the intake of these micronutrients and thereby correct or prevent micronutrient deficiencies.^9^ Complementary foods can be fortified either centrally (ie, during industrial processing) or at the point of use (home fortification).^9^

In malaria-endemic regions, the safety of iron preparations administered through home fortification has raised many questions,^10^ as the malaria parasite requires iron for growth^11^ and randomised controlled trials have indicated that a bolus of iron taken in a single dose might lead to adverse effects, such as increased risk of hospital admission, primarily due to malaria and infectious disease, and mortality, in these regions.^12^, ^13^ As fortified processed complementary foods enable iron to be consumed in smaller amounts throughout the day, and therefore absorbed more slowly, this form of iron administration might be a preferred alternative in these regions.^10^

Health effects of point-of-use fortification of complementary foods in children aged 6–23 months were assessed in a recent systematic review.^7^ Several trials exist on the potential beneficial and harmful effects of complementary feeding with centrally fortified foods,^14^, ^15^, ^16^, ^17^, ^18^, ^19^, ^20^, ^21^ although there is currently no up-to-date systematic review summarising potential health effects of adding micronutrients to industrially processed and widely consumed complementary food products.^22^

We aimed to assess the effects of micronutrient-fortified complementary food compared with the unfortified version of the same complementary food on beneficial or harmful dietary and health outcomes in infants and young children aged 6–23 months.

## Methods

### Search strategy and selection criteria

For this systematic review and meta-analysis, an information specialist experienced in systematic reviews (M-IM) searched the following electronic databases and trial registers from the inception of each database up to March 9, 2021, without restrictions on the language of publication: Ovid MEDLINE, Cochrane Central Register of Controlled Trials, Cumulative Index to Nursing and Allied Health Literature, Global Index Medicus (comprising African Index Medicus, Index Medicus for the Eastern Mediterranean Region, Index Medicus for the South-East Asia Region, Latin America and the Caribbean Literature on Health Science, and Western Pacific Region Index Medicus), Embase, Web of Science (comprising Science Citation Index and Emerging Citation Index), ClinicalTrials.gov, and the WHO International Clinical Trials Registry Platform. Details for all search strategies, including search terms, are available in the appendix (pp 2–4). We tried to identify other potentially eligible trials or ancillary publications by searching the reference lists of included trials and related systematic reviews, meta-analyses, and health technology assessment reports. Study authors were not contacted, and articles were translated into English if necessary.

We included randomised controlled trials with both individual and cluster randomisation and controlled clinical trials, if concurrently controlled. Included children were aged 6–23 months at the start of the intervention. We intended to include apparently healthy children from the general population, although some might have been at risk of having highly prevalent diseases (eg, malaria, HIV, diarrhoea, and undernutrition). The eligible intervention was consumption of fortified complementary products (excluding milk and milk-based formula), fortified centrally with one micronutrient or a combination of vitamins, minerals, or both. We excluded food supplements, micronutrient powders, or any other ways of home (point-of-use) fortification. The comparator was consumption of an unfortified version of the same complementary product.

Pairs of review authors (IC, RF, ÉS, and SL) independently screened titles and abstracts of every retrieved record using Covidence. Full texts of all potentially relevant records were screened for eligibility. Any disagreements were resolved through consensus or by recourse to a third author (IC or SL). All records excluded after full-text assessment are listed in the appendix (pp 5–36).

From full-text publications, we extracted data on study methods, participants, interventions, controls, outcomes, sources of funding, and potential conflict of interest statements. Data were extracted by one reviewer (IC or RF) and checked for completeness, accuracy, and consistency by a second reviewer (IC or RF).

We included abstracts and conference proceedings, but did not use them for data extraction, because they do not fulfil the CONSORT requirements.^23^ Data available as study results in trials registries were also extracted.

Two review authors (ÉS and SL) independently assessed the risk of bias of each included trial. Any disagreements were resolved by consensus. Risk of bias was evaluated with version 2 of the Cochrane risk of bias tool for randomised trials (RoB 2).^24^ Overall risk of bias was defined for each trial as the least favourable assessment across the domains of bias.

### Outcomes

The outcomes of interest reflect the actual public health need on information and were defined by the WHO guideline development group (GDG) on complementary feeding of infants and young children aged 6–23 months. The main outcomes were growth (measured by Z scores for weight for age, weight for height or length, and height or length for age, or other growth measures), stunting, wasting, nutrient adequacy or excess, anaemia, haemoglobin concentration, iron status, serum zinc concentration, and serum retinol concentration. Additional outcomes were all-cause mortality, adverse effects, mental and motor skill development, morbidity, ferritin concentration, serum or urine concentration of other vitamins or minerals, gut microbiota composition, taste preference, and displacement of other foods. We included outcomes as measured at any given timepoint.

### Data analysis

We a priori planned to undertake a meta-analysis for all outcomes for which we judged the participants, interventions, comparisons, and outcomes to be sufficiently similar to ensure a result that was clinically meaningful.

For dichotomous data, we present results as risk ratios or odds ratios (ORs) with 95% CIs. For continuous data, we use mean differences with 95% CIs for studies measuring outcomes in the same way, and standardised mean differences with 95% CIs for studies measuring outcomes in various ways.

We combined results from cluster-randomised and individually randomised studies. Where trial authors had adjusted their results for the effect of clustering, we aimed to extract the cluster-adjusted risk ratios and SE and enter the natural log of these into Review Manager (RevMan) using the generic inverse variance method, as recommended by Higgins and colleagues.^25^ Otherwise, we extracted the simple summary data for all relevant outcomes and calculated crude risk ratios and 95% CIs using RevMan. We adjusted for the effects of clustering using the approximate analysis method.^25^ This involves inflating the SE of the risk ratio using an estimate of the design effect and entering the natural logs of the adjusted risk ratios and corresponding SEs into RevMan using the generic inverse variance method. The intracluster correlation coefficient was not reported in any of the trials, so the value of 0·03 was used, as suggested by Leyrat and colleagues.^26^ A sensitivity analysis with respect to the intracluster correlation coefficient was not undertaken. We examined the potential effects of clustering using sensitivity analyses.

For outcomes with skewed data (presented as geometric means or medians), we calculated log-transformed data for all studies and did a meta-analysis on the scale of the log-transformed data. For multi-arm studies, we included the directly relevant arm only.^27^ If a study compared two possible fortified products with one non-fortified comparator, we combined groups to create a single pairwise comparison.^28^

We assessed methodological heterogeneity by examining risk of bias, and clinical heterogeneity by examining similarities and differences between studies regarding types of participants, interventions, and outcomes. We considered the size and direction of effect and used a standard χ^2^ test with a significance level of α=0·1^19^ and the *I*^2^ statistic, which quantifies inconsistency across trials, to assess the effect of heterogeneity on the meta-analysis.^29^, ^30^ We explored heterogeneity through prespecified subgroup analyses.

We used funnel plots to assess reporting bias and to investigate the relationship between effect size and SE when at least ten studies were included in a meta-analysis. The degree of funnel plot asymmetry was quantified using Egger's test.^31^ The Robvis tool was used to visualise risk of bias.

As we expected differences between studies in both the population and the intervention, we decided to combine the data using a random-effects model. We used Mantel-Haenszel weighting for dichotomous outcomes and inverse variance for continuous outcomes or in case both individually and cluster-randomised trials were included in a meta-analysis.

We planned to do subgroup analyses for the following characteristics: age groups, different types of nutrients added through fortification, different types of products fortified, duration of intervention, the country income classification (defined according to the World Bank country income classification^32^), anaemia status at the start of the intervention, and sponsor. The potential effects of clustering were examined by sensitivity analyses.

We did statistical analyses using RevMan 5 (version 5.4.1). We followed the GRADE approach to rate the certainty of evidence.^33^ The methodology and the results are reported according to the Preferred Reporting Items for Systematic reviews and Meta-Analyses (PRISMA) reporting guideline.^34^ This study is registered with PROSPERO, CRD42021245876.

### Role of the funding source

The present work serves as a background evidence review for the WHO guidelines on feeding of infants and young children aged 6–23 months. The questions guiding the review were discussed and developed, and the study protocol was approved, by the WHO GDG on complementary feeding of infants and young children aged 6–23 months. WHO and its GDG had no role in data collection, analysis, or interpretation. The WHO GDG members peer reviewed the systematic review report. The present manuscript is based on the report. WHO and its GDG did not participate in the writing of the present manuscript.

## Results

**Figure 1:**
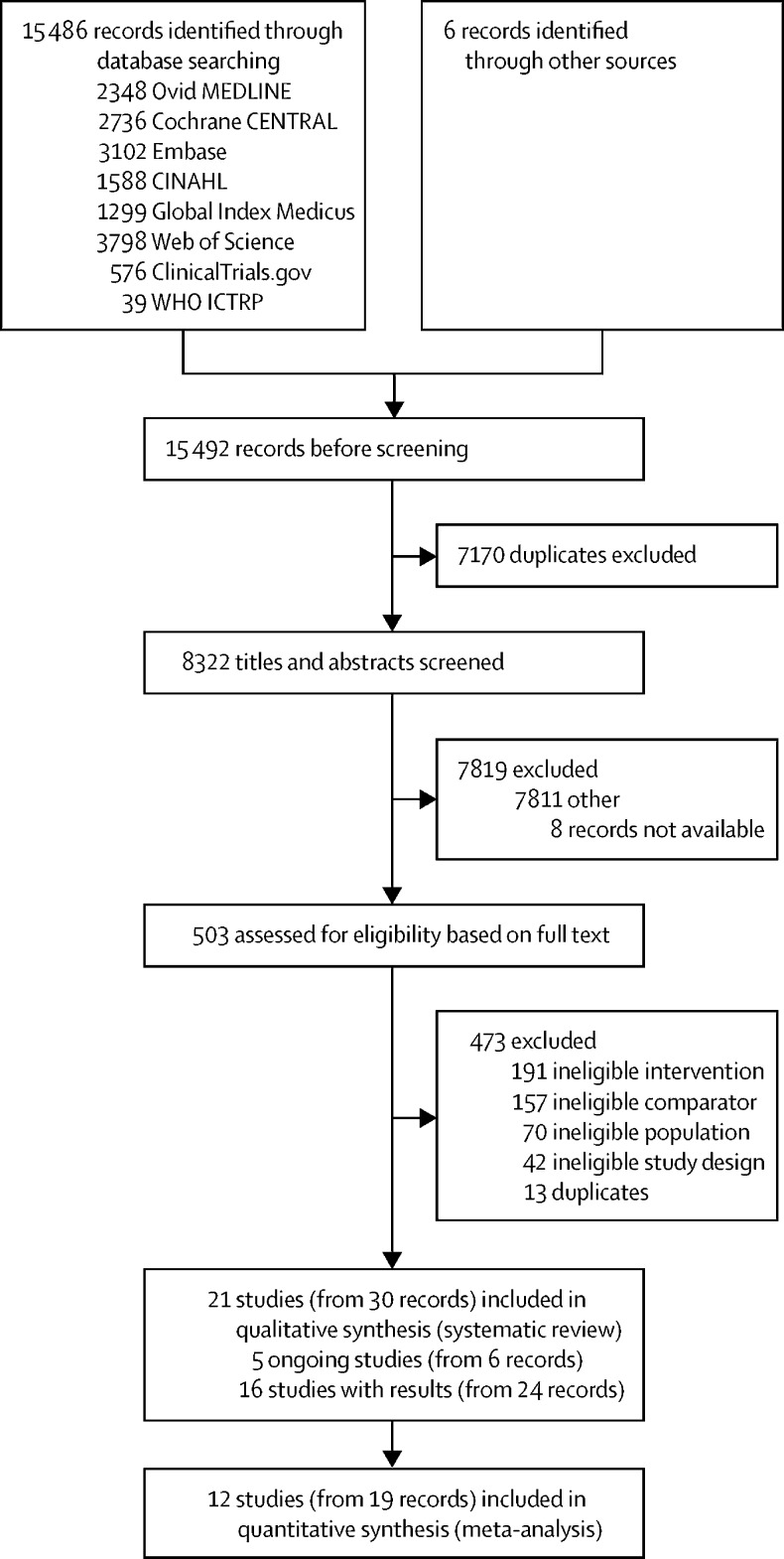
Study selection CENTRAL=Central Register of Controlled Trials. CINAHL=Cumulative Index to Nursing and Allied Health Literature. ICTRP=International Clinical Trials Registry Platform.

We retrieved 15 486 unique records through database searching and six through citation searching (figure 1). After removing duplicates, 8322 records were screened on the basis of their titles and abstracts (figure 1). We evaluated 503 full texts or records to determine their eligibility for inclusion in the review. 21 studies met our inclusion criteria (16 studies with full-text publications and five further studies that are not yet published, including key data from abstracts; appendix pp 37–41).

We included 16 studies with 6423 participants (table; appendix pp 42–56). Eight studies were randomised controlled trials randomised at the individual level,^14^, ^35^, ^37^, ^44^, ^46^, ^47^, ^48^, ^50^ one was a controlled clinical trial,^38^ and seven were cluster-randomised controlled trials.^2^, ^19^, ^36^, ^39^, ^42^, ^43^, ^45^ Most of the studies were done in upper or lower-middle-income countries. One study was done in a high-income country,^50^ and no study was done in a low-income country. Three studies were done in non-malaria-endemic areas (appendix pp 57–58).^52^

**Table:** Key characteristics of included studies

	**Country**	**Study design**	**Sample size**	**Age at start of intervention (months)**	**Fortified product**	Micronutrients added to the fortified products*	**Duration of intervention**	**Outcomes**
Palmer et al (2021)^35^	Zambia	RCT, parallel	255	9–12	Maize meal	Retinyl palmitateor β-carotene (biofortified)	90 days	Plasma retinol, vitamin A total body stores, liver retinol
Ekoe et al (2020)^36^	East Cameroon	RCT, cluster	205	18–59	Infant cereal	Iron (ferrous fumarate)	6 months	Haemoglobin, serum ferritin, serum iron, C-reactive protein, transferrin, anaemia, nutrition status, iron deficiency, iron deficiency anaemia, weight, height, weight-for-age Z scores, height or length-for-age Z scores, weight-for-height or length Z scores
Gannon et al (2019)^37^	India	RCT, cross-over	52	6–24	Crops	Biofortified crops (not further specified)	3 consecutive days	Acceptability
Huey et al (2017)^38^	India	CCT, cross-over	125	12–24	Pearl millet	Iron and zinc (biofortified)	3 consecutive days	Acceptability
Ma et al (2016),^39^ Krebs et al (2013),^40^ Sheng et al (2019)^41^	China	RCT, cluster	1465	6	Rice cereal	Iron (ferrous fumarate), zinc (zinc sulphate), vitamin B12	12 months	Weight-for-age Z scores, height or length-for-age Z scores, weight-for-height or length Z scores, serum vitamin B12, haemoglobin, body iron, anaemia, ferritin, mean corpuscular volume, mean corpuscular haemoglobin, mean corpuscular haemoglobin concentration, cognitive score, fine motor score, gross motor score
Nogueira Arcanjo et al (2012)^42^	Brazil	RCT, cluster	216	10–23	Rice	Iron (ferric pyrophosphate)	18 weeks	Haemoglobin, anaemia
Nogueira Arcanjo et al (2013)^43^	Brazil	RCT, cluster	171	10–23	Rice	Iron (ferric pyrophosphate)	18 weeks	Haemoglobin, anaemia
del Refugio Carrasco Quintero et al (2011)^44^	Mexico	RCT, parallel	395	7–24	Corn flour	Iron, zinc, vitamin A, vitamin B3, folic acid	10 months	Weight, height, nutritional status, weight-for-age Z scores, weight-for-height or length Z scores, mental and psychomotor development, haemoglobin
Bagni et al (2009)^45^	Brazil	RCT, cluster	354	12–60	Rice	Iron (bisglycine chelate)	16 weeks	Haemoglobin, anaemia
Nesamvuni et al (2005)^46^	South Africa	RCT, parallel	44	12–36	Maize meal	Vitamins A, B1, B2, B6	12 months	Weight, height, haemoglobin, haematocrit, serum retinol, serum retinol-binding protein
Faber et al (2005)^47^	South Africa	RCT, parallel	361	6–12	Porridge	β-carotene, iron (ferrous fumarate), zinc (zinc sulphate), vitamin C (sodium ascorbate), copper, selenium, vitamin B2, vitamin B6, vitamin B12, vitamin E	6 months	Motor development, weight, length, height or length-for-age Z scores, weight-for-age Z scores, weight-for-height or length Z scores, haemoglobin, serum ferritin, serum retinol, serum zinc
Schümann et al (2005)^48^	Guatemala	RCT, parallel	110	12–36	Beans	Iron (ferrous sulphate; inorganic salt) or haem iron (from bovin blood)	10 weeks	Haemoglobin, ferritin
Lartey et al (1999),^14^ Lartey et al (2000)^49^	Ghana	RCT, parallel	216	6	Cereal–legume blend	Calcium, iron, zinc, copper, magnesium, potassium, sodium, phosphorus, vitamin C, vitamin B3, vitamin B6, vitamin B2, vitamin B1, vitamin B12, folic acid, vitamin A	6 months	Plasma zinc, plasma retinol, erythrocyte vitamin B2, haemoglobin, haematocrit, plasma ferritin, plasma transferrin, plasma transferrin saturation, diarrhoea, fever, respiratory illness, dietary iron intake, zinc intake, anaemia, vitamin A intake, vitamin B2 intake, weight-for-age Z scores, height or length-for-age Z scores, weight gain, length gain, mid-upper arm circumference, head circumference, tricep skinfold thickness, subscapular skinfold thickness, mid-upper arm fat area, mid-upper arm muscle area
Bovell-Benjamin et al (1999)^50^	USA	RCT, cross-over	40	6–24	Whole-maize meal	Iron (ferrous bisglycinate)	3 subsequent sessions	Degree of liking
Liu et al (1993)^19^	China	RCT, cluster	164	6–13	Rusk	Calcium, iron, zinc, vitamin A (retinyl acetate), vitamin D3, vitamin B1, vitamin B2, vitamin B3, vitamin B12, folic acid	3 months	Weight, length, free erythrocyte porphyrin, plasma ferritin, erythrocyte glutathione reductase activation coefficient, plasma vitamin E, plasma vitamin A, haemoglobin
Gershoff et al (1977),^2^ Gershoff et al (1975)^51^	Thailand	RCT, cluster	2250	6–60	Rice	Vitamin B1 (thiamin naphthalene disulphonate), vitamin B2, vitamin A (retinol acetate), iron (ferric phosphate)	1–4 years	Length, weight, bone age, head circumference, chest circumference, arm circumference, tricep skinfold, subscapular skinfold, haemoglobin, haematocrit, morbidity, hand–wrist x-ray

Vitamin A1 is also known as retinol. Vitamin B1 is also known as thiamine. Vitamin B2 is also known as riboflavin. Vitamin B3 is also known as niacin. Vitamin B6 is also known as pyridoxine. Vitamin B12 is also known as cyanocobalamin. Vitamin C is also known as ascorbic acid. Vitamin D3 is also known as cholecalciferol. CCT=controlled clinical trial. RCT=randomised controlled trial.

* Further details on the amount of micronutrients used for fortification can be found in the appendix (pp 59–60).

Participant age ranged from six to 60 months (three studies included children aged up to 60 months). When possible, we only included data for children younger than 24 months. Sample sizes ranged from 40^50^ to 2250.^2^ Three studies had an intervention duration of three subsequent feeding sessions^50^ to 3 consecutive days.^37^, ^38^ Among studies investigating longer-term effects of fortified complementary food consumption, the intervention duration lasted between 10 weeks^48^ and 4 years.^2^, ^51^ Fortified products were cereals in most of the cases (appendix pp 59–62).

Overall, eight (50%) studies were rated with high risk of bias due to the randomisation process and carry-over effects,^38^ deviations from the intended interventions,^2^, ^45^, ^46^, ^47^, ^48^ or missing outcome data^2^, ^19^, ^46^, ^47^, ^48^, ^50^ (appendix p 63). The evidence profiles are shown in the appendix (pp 64–72).

**Figure 2:**
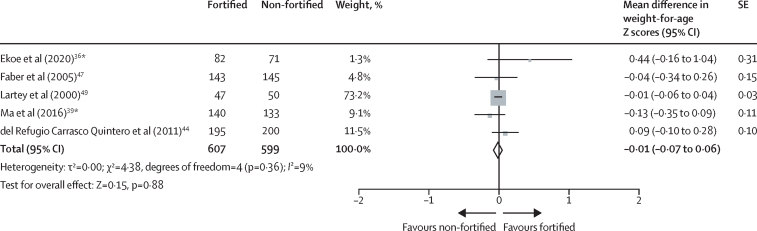
Effect of fortified versus non-fortified complementary food on weight for age (in Z scores) *Cluster-randomised controlled trial.

Five trials with an intervention duration of 6 months or longer measured growth.^36^, ^39^, ^44^, ^47^, ^49^ Available evidence showed no difference between groups in weight-for-age Z scores (mean difference −0·01, 95% CI −0·07 to 0·06; five trials; 1206 participants; moderate-certainty evidence; figure 2), weight-for-height or length Z scores (−0·05, −0·19 to 0·10; four trials; 1109 participants; moderate-certainty evidence; appendix p 82), and height or length-for-age Z scores (−0·01, −0·21 to 0·20; four trials; 811 participants; low-certainty evidence; appendix p 85).

**Figure 3:**
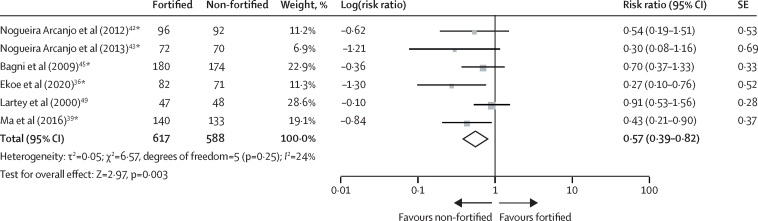
Effect of fortified versus non-fortified complementary food on anaemia prevalence *Cluster-randomised controlled trial.

Anaemia was assessed in six trials (including 1205 participants) with 4–12 months duration.^36^, ^39^, ^42^, ^43^, ^45^, ^49^ Children receiving fortified complementary food products were significantly less likely to have anaemia at follow-up than children receiving non-fortified complementary foods (risk ratio 0·57, 95% CI 0·39–0·82; moderate-certainty evidence; figure 3).

**Figure 4:**
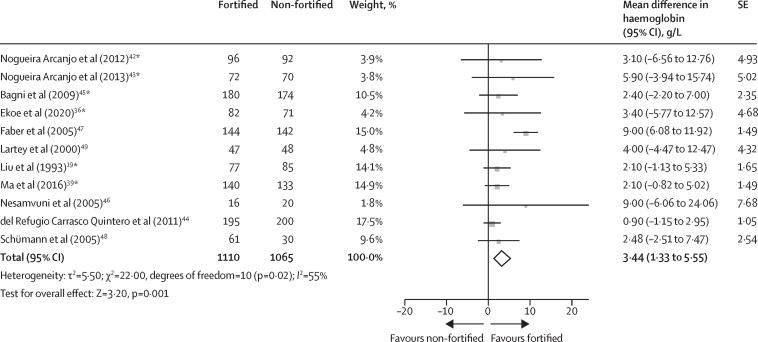
Effect of fortified versus non-fortified complementary food on haemoglobin (g/L) *Cluster-randomised controlled trial.

Compared with children receiving a non-fortified complementary food product, children consuming fortified complementary foods for 10 weeks to 12 months had higher haemoglobin concentrations at follow-up (mean difference 3·44 g/L, 95% CI 1·33–5·55; 11 trials; 2175 participants; moderate-certainty evidence; figure 4). We found no evidence of reporting bias (Egger's test p=0·37; appendix p 92).

Iron deficiency, defined as ferritin concentrations of less than 12 μg/L, was investigated in three studies with 571 participants.^36^, ^39^, ^49^ Children consuming iron-fortified complementary foods for 6 or 12 months were significantly less likely to have iron deficiency at follow-up than children consuming non-fortified complementary foods (risk ratio 0·39, 95% CI 0·21–0·75; moderate-certainty evidence; appendix p 91).

**Figure 5:**
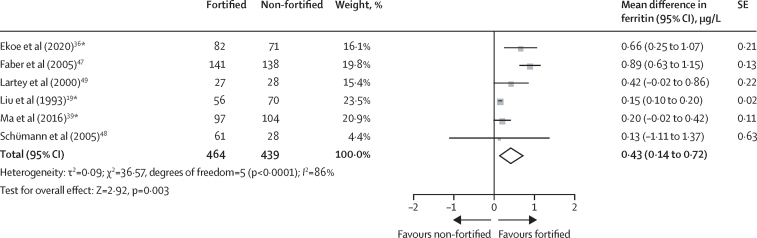
Effect of fortified versus non-fortified complementary food on iron status (ferritin concentrations in μg/L) *Cluster-randomised controlled trial.

Iron status was measured as ferritin concentration, body iron, or free erythrocyte porphyrin. A daily dose of iron added to the complementary food products via fortification varied among studies, with a daily amount of 0·36 mg,^39^ 2·5 mg,^36^ 3·66 mg,^47^ 4·6 mg,^48^ or 4·93 mg^19^ of elemental iron consumed, or with more than 10·9 mg per day or less than 21·9 mg per day.^49^ Children receiving iron-fortified complementary foods for 12 weeks to 12 months had, on average, higher ferritin concentrations at follow-up than children consuming non-fortified complementary food (mean difference 0·43 μg/L on log scale, 95% CI 0·14–0·72; six trials; 903 participants; low-certainty evidence; figure 5). One study^39^ described rice cereal fortified with iron, zinc, and vitamin B12 to be effective in increasing body iron, whereas another study^19^ found no effects of rusks fortified with iron, zinc, and calcium on free erythrocyte porphyrin (appendix pp 89–90).

Serum zinc concentration was measured in two studies, in which less than 10·26 mg per day^49^ and 6 mg per day^47^ daily doses of zinc were provided, in combination with other micronutrients. The percentage of children with low serum zinc concentrations (cutoff values defined as serum zinc <10·7 μmol/L^49^ or <9·9 μmol/L^47^) at baseline was 3·6% (among 120 participants)^49^ and 43–48% (among 289 participants).^47^ Children consumed fortified food for 6 months in both studies. These studies found no effect of the provision of a zinc-fortified complementary food on children's serum zinc concentrations (mean difference −0·13 g/dL, 95% CI −0·82 to 0·56; two trials; 333 participants; low-certainty evidence; appendix p 90).

Prevalence of children with low zinc values (defined as <10·7 μmol/L) decreased from 6·5% to 3·2% in the non-fortified, and increased from 3·3% to 10·0% in the fortified groups (appendix p 91) in one study with 61 children assessing zinc deficiency.^49^

In trials reporting on serum retinol concentrations, the percentage of children with serum retinol concentrations of less than 0·7 μmol/L at baseline were 17% for those who received fortified complementary food versus 19% for those who received non-fortified complementary food,^47^ 21·6 versus 34·5%,^14^ 0 versus 7%,^46^ 19·6 versus 34·5%,^35^ and not reported for one study.^19^ Overall, the provision of fortified complementary foods compared with non-fortified complementary food products had no effect on serum retinol concentrations (mean difference 0·03 μmol/L, 95% CI −0·02 to 0·08; five trials; 475 participants; moderate-certainty evidence; appendix p 90).

In three studies assessing vitamin A deficiency after an intervention period from 3 months to 12 months,^35^, ^46^, ^49^ there were no differences in the likelihood of having vitamin A deficiency (defined as serum retinol concentrations of <0·70 μmol/L) at follow-up between children consuming fortified and unfortified complementary foods (risk ratio 0·97, 95% CI 0·24–3·90; three trials; 257 participants; very-low-certainty evidence; appendix p 91). One study reported on nutrient adequacy and described that children in the fortified cereal–legume blend group consumed two to three times the recommended intake from zinc and vitamin A as a result of fortification.^14^

Mental skill development was assessed in two studies (508 participants), one^44^ measuring it on the first version of the Bayley Scales of Infant Development (BSID I), and the other^39^ on BSID III. Overall, children consuming fortified complementary foods had higher scores than children consuming the unfortified version of the same complementary food (mean difference 0·80, 95% CI 0·12–1·48; moderate-certainty evidence; appendix p 90). Motor skill development was reported as fine motor and gross motor score (measured on BSID III) in one study,^39^ as psychomotor score (measure on BSID I) in one study,^44^ and 25-item motor development score (BSID II) in one study.^47^ Psychomotor development was improved in children consuming fortified complementary food (mean difference 1·13, 95% CI 0·35–1·91; two trials; 661 participants; low-certainty evidence; appendix p 91). This effect was not seen in the study investigating fine and gross motor scales separately (appendix p 91).^39^

One trial with 97 children reported on morbidity,^49^ defined as number of new episodes per 100 days at risk. The number of cases of diarrhoea, acute respiratory tract infection, and fever disease episodes did not differ between children consuming fortified and non-fortified complementary foods (very-low-certainty evidence; appendix p 90).

Acceptability of fortified compared with non-fortified complementary foods was measured in two randomised controlled trials^37^, ^50^ and one controlled clinical trial^38^ with a total of 215 children. All studies evaluated acceptance on a 9-point hedonic scale (answers of toddlers interpreted by mothers), with a higher score representing better acceptance. Degree of liking did not differ significantly in any of these studies between fortified and non-fortified groups.

No studies reported data on the following prespecified outcomes: stunting and wasting, nutrient intakes above the upper limit, all-cause mortality, gut microbiota composition, or displacement of other foods.

Adverse effects were reported in one study, in which fortified complementary foods “were well tolerated and no side effects were reported in either group”.^36^

For the growth outcome, no relevant subgroup effects were detected, and cluster-randomised controlled trials were shown to have no effect on the direction of the pooled estimate in sensitivity analyses. For the outcomes of anaemia prevalence, haemoglobin concentration, and iron status, inconsistent differences were seen among age subgroups and the types of products that were fortified. Baseline anaemia status did not affect favourable effects of fortified complementary foods on post-intervention anaemia prevalence and iron status, but had effects on haemoglobin concentrations (all subgroup analyses are shown in the appendix pp 73–91). The beneficial effect of fortified complementary foods on haemoglobin concentration was no longer significant when all of the cluster-randomised controlled trials were excluded in the sensitivity analysis (mean difference 4·48 g/L, 95% CI −0·10 to 9·05; five trials;^44^, ^46^, ^47^, ^48^, ^49^ 903 participants), but were still significant in the case of iron status (0·64 μg/L on log scale, 95% CI 0·23 to 1·05; three trials;^47^, ^48^, ^49^ 423 participants).

## Discussion

To our knowledge, this is the first systematic review summarising evidence on the consumption of fortified complementary foods compared with the unfortified version of the same complementary foods in infants and children aged 6–23 months. Results showed that providing fortified complementary foods to children probably reduces anaemia by 43%. Participants who receive fortified complementary foods probably have higher haemoglobin and might have higher ferritin concentrations than those who receive non-fortified complementary foods. Fortification with the applied micronutrient composition and doses probably makes little or no difference to growth outcomes. The intervention might result in little to no effect on zinc and vitamin A concentrations; there is no available evidence of effects on other vitamin and mineral concentrations. Acceptability of fortified and non-fortified complementary products in terms of sensory characteristics might not differ. Results of this systematic review are limited to preventive purposes; effectiveness of food fortification in treating any form of malnutrition was not evaluated.

As of March 30, 2022, two of five ongoing studies had been published as full-text papers.^53^, ^54^ One of them proved to be ineligible to be included in this systematic review because it compared two different fortified food products.^53^ The other study provides additional data for the outcomes haemoglobin, ferritin, zinc, and retinol concentrations, and on growth measures,^54^ which do not change the conclusions.

WHO guidelines recommend that: “In populations where anaemia is a public health problem, point-of-use fortification of complementary foods with iron-containing micronutrient powders in infants and young children aged 6–23 months is recommended, to improve iron status and reduce anaemia”.^55^ Our systematic review and meta-analysis shows that fortified foods formulated specifically for infants and young children might be an effective alternative.

This study has several strengths. First, we used a broad search strategy in both electronic databases and trial registries without applying date or language restrictions. It is unlikely that published trials have been missed; however, unpublished or ongoing trials not registered in clinical trial registries could be missing. Second, we aimed to reduce bias wherever possible by having at least two review authors work independently on trial selection, data extraction, and risk of bias and GRADE assessments.

A limitation of this systematic review is that several prespecified outcomes were investigated in a small number of trials or no data were available at all. Second, authors of ongoing studies were not contacted. We were able to explore the potential for publication bias using funnel plots only for the outcome haemoglobin concentration. Included studies were published between 1977 and 2020. Both the foods fortified and the micronutrients used for fortification were diverse; most of the studies provided iron either alone or in combination with other micronutrients.

We checked other systematic reviews and meta-analyses and investigated their agreements and disagreements with our findings. We found two studies: one review assessed the effects of a wide range of iron-containing interventions on anaemia and iron status in infants and children younger than 3 years,^56^ and the other assessed the health effect of centrally processed micronutrient-fortified dairy products and fortified cereal in children aged 6 months to 5 years.^57^ These two reviews found that iron fortification increased haemoglobin concentrations and reduced anaemia. Developmental effects of a range of interventions, including micronutrient-fortified complementary foods, were assessed in a systematic review in infants and children aged between 6 months and 2 years,^58^ with only two included studies assessing the effects of fortified processed foods, both finding no effect on growth.

The findings of our review are generalisable to apparently healthy children in middle-income settings in Asia and Africa, although some children might have been at risk of having highly prevalent diseases such as malaria, diarrhoea, or malnutrition. In malaria-endemic regions, malaria might be an additional cause of anaemia besides iron deficiency, infections, or other nutrient deficiencies, as the malaria parasite destroys erythrocytes.^9^ In such regions, nutritional status results have to be interpreted cautiously because malaria substantially reduces haemoglobin concentrations^59^ and affects many other nutritional status indicators, including serum ferritin, serum transferrin, and plasma retinol.^60^ Therefore, biomarkers of iron and vitamin A status should be statistically adjusted for malaria and the severity of infection.^61^ Most of the included studies in our systematic review were done in malaria-endemic areas; however, none reported adjustments for malaria. Thus, the effect of malaria on our results cannot be accurately judged.

Some studies suggest that fetal iron deficiency might be detrimental to early neurodevelopment, but early iron supplementation in anaemic infants can have a positive effect on developmental scores (both cognitive and motor), regardless of a country's income status.^62^ Micronutrients as a sole intervention (ie, without an additional increase in macronutrient intake), however, seem to have no effect on growth.

Micronutrient deficiencies and their health consequences remain a global health problem, and several factors, including age of the target population, baseline anaemia status, malaria prevalence, and presence of other causes of infection or inflammation, need to be considered.^22^, ^63^ Beside products from multinational companies, locally manufactured, lower-cost fortified complementary food products are available in low-income countries; however, access to these products is still far from optimal in several settings.^64^, ^65^ WHO states: “To ensure their success and sustainability, especially in resource-poor countries, food fortification programmes should be implemented in concert with poverty reduction programmes and other agricultural, health, education and social intervention programmes that promote the consumption and utilization of adequate quantities of good quality nutritious foods among the nutritionally vulnerable.”^9^

Several research gaps have to be further explored. As provision of iron in any product that is given to infants and young children raises questions of safety, potential adverse effects have to be further explored in both malaria-endemic and non-malaria-endemic settings. Studies should investigate whether consumption of fortified complementary foods has an effect on all-cause mortality. It would be important to know what level of fortification can lead to adequate or excess nutrient intakes, affect stunting or wasting, or influence vitamin and mineral status other than iron. Further studies are required to answer the question under what circumstances the provision of fortified complementary foods to infants and young children is able to affect mental and motor skill development. Microbiota composition has not yet been investigated at all. How the consumption of such foods affects the intake of other foods, diet diversity, or breastfeeding time—ie, whether and to what extent they displace other foods—should also be determined.

Studies with both higher and lower nutrient content have to be done to determine dose–response effects. Effects of complementary food fortification should be further investigated in low-income and middle-income countries, but should also be assessed in high-income countries, and in regions where malaria is not endemic. Planned randomised controlled trials should be done with rigorous methodology and large sample sizes. Furthermore, there is a need to investigate the sustainability of different evidence-based interventions and factors that enhance or impede their implementation.^63^

In conclusion, micronutrient deficiencies and their health consequences are a global problem, and fortified complementary food products might be one effective strategy to improve the micronutrient status of young children. There is a need to enhance these targeted, evidence-based interventions to prevent micronutrient deficiencies in infants and young children aged 6–23 months.

For the **Robvis tool** see https://mcguinlu.shinyapps.io/robvis/

For **Covidence** see https://www.covidence.org/

## Data sharing

Full datasets can be obtained from the corresponding author.

## Declaration of interests

We declare no competing interests.
