# Correction to *Lancet Child Adolesc Health* 2022; 6: 533–44

**DOI:** 10.1016/S2352-4642(22)00319-4

**Published:** 2022-12

*Csölle I, Felső R, Szabó É, et al. Health outcomes associated with micronutrient-fortified complementary foods in infants and young children aged 6–23 months: a systematic review and meta-analyis.* Lancet Child Adolesc Health *2022; **6:** 533–44*—In Figure 3 of this Article, “Favours fortified” should be on the left side and “Favours non-fortified” should be on the right side of the x-axis in this forest plot. This correction has been made as of Nov 16, 2022.

