# Supplementary material for: Health outcomes associated with micronutrient-fortified complementary foods in infants and young children aged 6–23 months: a systematic review and meta-analysis
Source: Lancet Child Adolesc Health. 2022 Aug;6(8):533–44. doi: 10.1016/S2352-4642(22)00147-X (PMC9279162; doi:10.1016/S2352-4642(22)00147-X)
Supplement: Supplementary appendix [file mmc1.pdf]

THE LANCET
Child & Adolescent Health

## Supplementary appendix

This appendix formed part of the original submission and has been peer reviewed. We post it as supplied by the authors.

Supplement to: Csölle I, Felső R, Szabó É, et al. Health outcomes associated with micronutrient-fortified complementary foods in infants and young children aged 6–23 months: a systematic review and meta-analysis. *Lancet Child Adolesc Health* 2022; published online June 23. https://doi.org/10.1016/S2352-4642(22)00147-X.

# Supplementary files

## Table of contents

Supplement 1. Search strategies ....2
Supplement 2. Characteristics of excludes studies ....5
Supplement 3. Characteristics of studies awaiting classification ....37
Supplement 4.  Characteristics of included studies ....42
Supplement 5. Information about malaria in the area of the trial ....57
Supplement 6. Composition of fortified complementary foods ....59
Supplement 7. Risk of bias assessments ....63
Supplement 8. GRADE Assessment ....64
Supplement 9. ANALYSES ....73
Supplement 10. Funnel plot for the outcome haemoglobin ....92
Supplement 11. Acknowledgments ....93

## Supplement 1. Search strategies

### Medline (Ovid)

1. Food, Fortified/
2. ((complement* or supplement* or fortif* or enrich*) adj3 (food* or feed* or nutri*)).tw.
3. ((fortif* or enrich*) adj3 (cereal* or porridg* or maize or grain* or rice or wheat or corn or millet or cowpea* or soy or peanut* or pasta* or noodle* or bread* or bakery or rusk* or biscuit* or cake* or puree* or sauce* or snack* or drink* or juice*)).tw.
4. ((fortif* or enrich*) adj3 (vitamin* or multivitamin* or mineral* or micronutrient* or multimicronutrient* or nutri*)).tw.
5. or/1-4
6. exp Infant/
7. (baby or babies or infant* or toddler* or child or children* or kid or kids).tw.
8. or/6-7
9. 5 and 8
10. randomized controlled trial.pt.
11. controlled clinical trial.pt.
12. randomized.ab.
13. placebo.ab.
14. clinical trials as topic.sh.
15. randomly.ab.
16. trial.ti.
17. or/10-16
18. exp animals/ not humans.sh.
19. 17 not 18
20. 9 and 19
21. remove dupliates from 20

### Cochrane Central Register of Controlled Trials (Cochrane Register of Studies Online)

1. MESH DESCRIPTOR Food, Fortified
2. ((complement* or supplement* or fortif* or enrich*) ADJ4 (food* or feed* or nutri*)):TI,AB,KY
3. ((fortif* or enrich*) ADJ4 (cereal* or porridg* or maize or grain* or rice or wheat or corn or millet or cowpea* or soy or peanut* or pasta* or noodle* or bread* or bakery or rusk* or biscuit* or cake* or puree* or sauce* or snack* or drink* or juice*)):TI,AB,KY
4. ((fortif* or enrich*) ADJ4 (vitamin* or multivitamin* or mineral* or micronutrient* or multimicronutrient* or nutri*)):TI,AB,KY
5. #1 OR #2 OR #3 OR #4
6. MESH DESCRIPTOR Infant EXPLODE ALL TREES
7. (baby or babies or infant* or toddler* or child or children* or kid or kids):TI,AB,KY
8. #6 OR #7
9. #5 AND #8

**CINAHL (EbscoHost)**

1. MH "Food, Fortified"
2. TI ((complement* or supplement* or fortif* or enrich*) N3 (food* or feed* or nutri*)) OR AB ((complement* or supplement* or fortif* or enrich*) N3 (food* or feed* or nutri*))
3. TI ((fortif* or enrich*) N4 (cereal* or porrdig* or maize or grain* or rice or wheat or corn or millet or cowpea* or soy or peanut* or pasta* or noodle* or bread* or bakery or rusk* or biscuit* or cake* or puree* or sauce* or snack* or drink* or juice*)) OR AB ((fortif* or enrich*) N4 (cereal* or porrdig* or maize or grain* or rice or wheat or corn or millet or cowpea* or soy or peanut* or pasta* or noodle* or bread* or bakery or rusk* or biscuit* or cake* or puree* or sauce* or snack* or drink* or juice*))
4. TI ((fortif* or enrich*) N4 (vitamin* or multivitamin* or mineral* or micronutrient* or multimicronutrient* or nutri*)) OR AB ((fortif* or enrich*) N4 (vitamin* or multivitamin* or mineral* or micronutrient* or multimicronutrient* or nutri*))
5. S1 OR S2 OR S3 OR S4
6. MH "Infant+"
7. TI (baby or babies or infant* or toddler* or child or children* or kid or kids) OR AB (baby or babies or infant* or toddler* or child or children* or kid or kids)
8. S6 OR S7
9. S5 AND S8
10. MH "treatment outcomes+" OR MH "experimental studies+" or random*
11. S9 AND S10

**Web of Science (Science Citation Index Expanded and Emerging Sources Citation Index)**

1. TI=((complement* or supplement* or fortif* or enrich*) NEAR/3 (food* or feed* or nutri*)) OR AB=((complement* or supplement* or fortif* or enrich*) NEAR/3 (food* or feed* or nutri*))
2. TI=((fortif* or enrich*) NEAR/4 (cereal* or porridg* or maize or grain* or rice or wheat or corn or millet or cowpea* or soy or peanut* or pasta* or noodle* or bread* or bakery or rusk* or biscuit* or cake* or puree* or sauce* or snack* or drink* or juice*)) OR AB=((fortif* or enrich*) NEAR/4 (cereal* or porridg* or maize or grain* or rice or wheat or corn or millet or cowpea* or soy or peanut* or pasta* or noodle* or bread* or bakery or rusk* or biscuit* or cake* or puree* or sauce* or snack* or drink* or juice*))
3. TI=((fortif* or enrich*) NEAR/3 (vitamin* or multivitamin* or mineral* or micronutrient* or multimicronutrient* or nutri*)) OR AB=((fortif* or enrich*) NEAR/3 (vitamin* or multivitamin* or mineral* or micronutrient* or multimicronutrient* or nutri*))
4. #1 OR #2 OR #3
5. TI=(baby or babies or infant* or toddler* or child or children* or kid or kids) OR AB=(baby or babies or infant* or toddler* or child or children* or kid or kids)
6. #4 AND #5
7. TI=(random* OR placebo OR trial OR groups) OR AB=(random* OR placebo OR trial OR groups)
8. #6 AND #7, Indexes=SCI-EXPANDED, ESCI Timespan=All years

**Embase**

#1. 'fortified food'/exp
#2. ((complement* OR supplement* OR fortif* OR enrich*) NEAR/3 (food* OR feed* OR nutri*)):ti,ab
#3. ((fortif* OR enrich*) NEAR/3 (cereal* OR porridg* OR maize OR grain* OR rice OR wheat OR corn OR millet OR cowpea* OR soy OR peanut* OR pasta* OR noodle* OR bread* OR bakery OR rusk* OR biscuit* OR cake* OR puree* OR sauce* OR snack* OR drink* OR juice*)):ti,ab
#4. ((fortif* OR enrich*) NEAR/3 (vitamin* OR multivitamin* OR mineral* OR micronutrient* OR multimicronutrient* OR nutri*)):ti,ab
#5. #1 OR #2 OR #3 OR #4
#6. 'infant'/exp
#7. baby:ti,ab OR babies:ti,ab OR infant*:ti,ab OR toddler*:ti,ab OR child:ti,ab OR children*:ti,ab OR kid:ti,ab OR kids:ti,ab
#8. #6 OR #7
#9. #5 AND #8
#10. 'randomized controlled trial'/exp
#11. 'double blind procedure'/exp
#12. 'crossover procedure'/exp
#13. 'parallel design'/exp
#14. 'single blind procedure'/exp
#15. random*:ti,ab
#16. doubl* NEAR/1 blind*
#17. singl* NEAR/1 blind*

#18. assign*:ti,ab
#19. allocat*:ti,ab
#20. volunteer*:ti,ab
#21. placebo*:ti,ab
#22. factorial*:ti,ab
#23. crossover*:ti,ab
#24. 'cross over':ti,ab
#25. #10 OR #11 OR #12 OR #13 OR #14 OR #15 OR #16 OR #17 OR #18 OR #19 OR #20 OR #21 OR #22 OR #23 OR #24
#26. #9 AND #25

**Global Index Medicus (WHO)**

(((complement* OR supplement* OR fortif* OR enrich*) AND (food* OR feed* OR nutri*)) OR ((fortif* or enrich*) AND (cereal* OR porridg* OR maize OR grain* OR rice OR wheat OR corn OR millet OR cowpea* OR soy OR peanut* OR pasta* OR noodle* OR bread* OR bakery OR rusk* OR biscuit* OR cake* OR puree* OR sauce* OR snack* OR drink* OR juice*)) OR ((fortif* or enrich*) AND (vitamin* OR multivitamin* OR mineral* OR micronutrient* OR multimicronutrient* OR nutri*))) AND (baby OR babies OR infant* OR toddler* OR child OR children* OR kid OR kids) AND (random* OR placebo OR trial OR groups)

**ICTRP (Standard search)**

fortif* AND infant* OR
fortif* AND child* OR
fortif* AND bab* OR
food* AND complement* AND infant* OR
food* AND complement* AND child* OR
food* AND complement* AND bab* OR
food* AND enrich* AND infant* OR
food* AND enrich* AND child* OR
food* AND enrich* AND bab* OR
feed* AND complement* AND infant* OR
feed* AND complement* AND child* OR
feed* AND complement* AND bab* OR
feed* AND enrich* AND infant* OR
feed* AND enrich* AND child* OR
feed* AND enrich* AND bab*

**ClinicalTrials.gov (Expert search)**

(((fortified OR fortification OR fortificant OR enriched) AND (food OR foods OR feeding OR cereal OR cereals OR porridge OR porridges OR maize OR corn OR wheat OR rusk OR drink OR vitamin OR vitamins OR multivitamin OR multivitamins OR mineral OR micronutrient OR micronutrients OR nutrient OR nutrients OR nutrition)) OR "complementary food" OR "complementary foods" OR "food complement" OR "complementary feeding" OR "micronutrient fortified" OR "vitamin fortified" OR "supplemented food" OR "supplemented foods") AND (baby OR babies OR infant OR infants OR toddler OR toddlers OR child OR children OR kid OR kids)

## Supplement 2. Characteristics of excludes studies

| Study | Reason for exclusion |
|---|---|
| Dewey 1998 [1] | Wrong comparator |
| Aakko 2017 [2] | Wrong comparator |
| Aaron 2011 [3] | Wrong comparator |
| Aboud 2011 [4] | Wrong intervention (responsive stimilation) |
| Ackatia-Armah 2012 [5] | Duplicate |
| Ackatia-Armah 2013 [6] | Wrong comparator |
| Ackatia-Armah 2015 [7] | Wrong comparator |
| ACTRN12609000061235 [8] | Wrong comparator |
| ACTRN12620000026921 [9] | Wrong intervention (prebiotic food) |
| Agapova 2018 [10] | Wrong intervention (non-fortified food) |
| Ahmad 2019 [11] | Wrong comparator |
| Ahmad 2020 [12] | Wrong comparator |
| Ahmed 2014 [13] | Wrong intervention (non-fortified food) |
| Ahmed 2017 [14] | Wrong comparator |
| Akalu 2010 [15] | Wrong intervention (non-fortified food) |
| Alemán 2008 [16] | Wrong intervention (snack prepared with quality protein maize) |
| Amthor 2009 [17] | Wrong study design |
| Anorve-Valdez 2018 [18] | Wrong intervention (MNP) |
| Arcanjo 2019 [19] | Wrong intervention (MNP) |
| Argaw 2018 [20] | Wrong intervention (n-3 LCPUFA) |
| Ariff 2013 [21] | Wrong intervention (MNP) |
| Arsenault 2007 [22] | Wrong intervention (fortified porridge combined with liquid multivitamin supplement) |
| Arsenault 2008 [23] | Wrong intervention (fortified porridge combined with liquid multivitamin supplement) |
| Arsenault 2016 [24] | Wrong intervention (fortified porridge combined with liquid multivitamin supplement) |
| Arya 2014 [25] | Wrong intervention (Shashtikashalyadi Churna) |
| Asad 2003 [26] | Wrong intervention (zinc as supplement) |
| Associationforthe 2013 [27] | Wrong comparator |
| Awasthi 2020 [28] | Wrong comparator |
| Badau 2016 [29] | Wrong study design |
| Bagni 2009 [30] | Duplicate |
| Bajaj 2005 [31] | Wrong intervention (low energy density diet) |
| Baskaran 1999 [32] | Wrong study design |
| Bauserman 2015 [33] | Wrong intervention (caterpillar cereal) |
| Becroft 1965 [34] | Wrong intervention (non-fortified food) |
| Beinner 2010 [35] | Wrong comparator |
| Bergmann 1989 [36] | Wrong comparator |
| Bernal 2013 [37] | Wrong intervention (cereals with different carbohydrate profiles) |
| Bhandari 2016 [38] | Wrong intervention (non-fortified product) |
| Bhargava 2013 [39] | Wrong study design |
| Bishop 1996 [40] | Wrong population |
| Bisimwa 2012 [41] | Wrong comparator |

| | |
|---|---|
| Boateng 2017 [42] | Wrong comparator |
| Boateng 2018 [43] | Wrong intervention (flour with added moringa leaf powder) |
| Boateng 2019 [44] | Wrong intervention (fortified with moringa leaf powder) |
| Borg 2017 [45] | Wrong comparator |
| Borg 2018 [46] | Wrong comparator |
| Borg 2019 [47] | Wrong comparator |
| Boston 2008 [48] | Wrong population (aged > 23 months) |
| Bouhouch 2015 [49] | Wrong population (aged > 23 months) |
| Brett 2018 [50] | Wrong population (aged > 23 months) |
| Brnic 2017 [51] | Wrong intervention (cereal porridge with phytase) |
| Brown 2007 [52] | Wrong intervention (fortified porridge combined with liquid multivitamin supplement) |
| Campbell 2015 [53] | Wrong comparator |
| Campbell 2016 [54] | Wrong comparator |
| Campbell 2016 [55] | Wrong comparator |
| Campbell 2017 [56] | Wrong study design |
| Campbell 2018 [57] | Wrong comparator |
| Campbell 2020 [58] | Wrong comparator |
| Capozzi 2011 [59] | Wrong intervention (iron fortified formula) |
| Carol 2019 [60] | Wrong population (children aged > 23 months) |
| Cercamondi 2013 [61] | Wrong comparator |
| Chauhan 2019 [62] | Wrong intervention (non-fortified food) |
| Chavasit 2015 [63] | Wrong comparator |
| Chilenje Infant Growth 2010 [64] | Wrong comparator |
| Chisenga 2011 [65] | Wrong study design |
| Chomba 2015 [66] | Wrong population (aged > 23 months) |
| Choudhury 2016 [67] | Wrong intervention (MNP) |
| Christian 2015 [68] | Wrong comparator |
| Christian 2015 [69] | Wrong comparator |
| Cliffer 2017 [70] | Wrong comparator |
| Cliffer 2020 [71] | Wrong comparator |
| Cook 1997 [72] | Wrong population |
| Cornell 2010 [73] | Wrong study design |
| Cornell 2012 [74] | Wrong population (aged > 23 months) |
| CTRI/2011/12/002259 [75] | Wrong intervention (non-fortified food) |
| CTRI/2017/08/009260 [76] | Wrong comparator |
| CTRI/2017/02/007767 2017 [77] | Wrong study design |
| Cubero 2009 [78] | Wrong intervention (cereal enriched with tryptophan) |
| Cuj 2016 [79] | Wrong study design |
| Dahl 2019 [80] | Wrong intervention (non-fortified porridge) |
| Daniels 2016 [81] | Wrong intervention (baby-led approach) |
| Daniels 2017 [82] | Wrong population (women) |
| Davidsson 2000 [83] | Wrong comparator |
| Davidsson 2003 [84] | Wrong population |
| Davidsson 2009 [85] | Wrong comparator |
| de Almeida 2003 [86] | Wrong study design |
| de Almeida 2005 [87] | Wrong comparator |

| | |
|---|---|
| de Almeida 2014 [88] | Wrong intervention (fortified water) |
| Delimont 2017 [89] | Wrong comparator |
| Delimont 2017 [90] | Wrong comparator |
| Delimont 2019 [91] | Wrong comparator |
| DeOliveira 2006 [92] | Wrong intervention (diet with a bran-based cereal mixture) |
| DeOliviera 1996 [93] | Wrong intervention (fortified water) |
| dePaula 2001 [94] | Wrong comparator |
| Dewan 2009 [95] | Wrong intervention (leaf protein concentrate) |
| Dewan 2009 [96] | Wrong intervention (leaf protein concentrate) |
| Dewey 1998 [97] | Wrong comparator |
| Dewey 2004 [98] | Wrong intervention (iron supplementation) |
| Dhingra 2012 [99] | Wrong comparator |
| Dong 2013 [100] | Wrong intervention (vitamin and mineral supplements) |
| Drks 2014 [101] | Wrong comparator |
| Dube 2010 [102] | Wrong intervention (low or high meat content) |
| Duggan 2003 [103] | Wrong comparator |
| Duizer 2017 [104] | Wrong intervention (home fortifiers) |
| Dutradeoliveira 1994 [105] | Wrong population (children aged > 23 months) |
| Ekbote 2011 [106] | Wrong population |
| Emel 2006 [107] | Wrong study design |
| Ernst 2013 [108] | Wrong intervention (beef biscuits) |
| Ernst 2014 [109] | Wrong intervention (non-fortified biscuits) |
| Faber 2005 [110] | Wrong study design |
| Fatmah 2018 [111] | Wrong population (aged > 23 months) |
| Ferreira 2008 [112] | Wrong intervention (bran-based cereal mixture as supplement) |
| Fhi 2018 [113] | Wrong population (aged > 23 months) |
| Filteau 2011 [114] | Duplicate |
| Filteau 2011 [115] | Wrong comparator |
| Fink 2017 [116] | Wrong intervention (growth monitoring) |
| Finn 2017 [117] | Wrong study design |
| Fleige 2010 [118] | Wrong study design |
| Food 2018 [119] | Wrong study design |
| Friel 2013 [120] | Wrong comparator |
| Friel 2014 [121] | Wrong comparator |
| Friel 2015 [122] | Wrong comparator |
| Friel 2016 [123] | Wrong comparator |
| Fuchs 1991 [124] | Wrong intervention (milk + fortified cereal or formula) |
| Fuchs 1991 [125] | Wrong intervention (milk + fortified cereal or formula) |
| Fuchs 1993 [126] | Wrong intervention (milk + fortified cereal or formula) |
| Galpin 2007 [127] | Wrong comparator |
| Gannon 2014 [128] | Wrong population (aged > 23 months) |
| Gannon 2014 [129] | Wrong population (aged > 23 months) |
| GarcĂa-Guerra 2009 [130] | Wrong comparator |
| Gartner 2006 [131] | Wrong study design |
| Geltman 2009 [132] | Wrong intervention (MNP) |
| Gershoff 1977 [133] | Wrong study design |

| | |
|---|---|
| Ghosh 2017 [134] | Wrong intervention (MNP) |
| Ghosh 2019 [135] | Wrong comparator |
| Gibson 2011 [136] | Wrong comparator (basal fortified vs. richly fortified porridge) |
| Glinz 2015 [137] | Wrong comparator |
| Glinz 2017 [138] | Wrong comparator |
| Glinz 2017 [139] | Duplicate |
| GodomarGalindo 1989 [140] | Wrong study design |
| Gough 2020 [141] | Wrong intervention (improved water, sanitation, and hygiene) |
| Grantham-McGregor 1989 [142] | Wrong intervention (full-cream powdered milk) |
| Granthammcgregor 1993 [143] | Wrong intervention (nutritional supplementation) |
| Gunaratna 2016 [144] | Wrong study design |
| Gutierrez 1998 [145] | Wrong intervention (received coupons) |
| Hambidge 1979 [146] | Wrong population (aged > 23 months) |
| Hambidge 2013 [147] | Wrong comparator |
| Harrington 2011 [148] | Wrong comparator |
| Haschke 1988 [149] | Wrong intervention (formula) |
| HaydomLutheran 2020 [150] | Wrong population (aged > 23 months) |
| HelenKeller 2011 [151] | Wrong comparator |
| HeroInstituteforInfant 2017 [152] | Wrong intervention (infant cereal with whole grain flour) |
| Herter-Aeberli 2017 [153] | Wrong population (aged > 23 months) |
| Herter-Aeberli 2017 [154] | Duplicate |
| Hertrampf 1990 [155] | Duplicate |
| Hertrampf 1990 [156] | Wrong intervention (Haemoglobin fortified cereal) |
| Hess 2017 [157] | Wrong intervention (zinc supplementation) |
| Hi 2020 [158] | Wrong population (aged <6 months) |
| Hilmers 2002 [159] | Wrong population (aged > 23 months) |
| Hlaing 2015 [160] | Wrong comparator |
| Hoffman 2004 [161] | Wrong intervention (fortified with DHA-enriched egg yolks) |
| Hoffman 2014 [162] | Wrong intervention (fortified with DHA-enriched egg yolks) |
| HospitalClinicoUniversitariode 2017 [163] | Wrong intervention (dairy product with prebiotic) |
| Hossain 2005 [164] | Wrong intervention (amylase-rich flour) |
| Huey 2017 [165] | Duplicate |
| Huo 2013 [166] | Wrong study design |
| Hussain 2004 [167] | Wrong intervention (lysine) |
| Huybregts 2012 [168] | Wrong intervention (non-fortified food) |
| Isanaka 2008 [169] | Wrong comparator |
| Isanaka 2019 [170] | Wrong comparator |
| ISRCTN47598408 [171] | Wrong comparator |
| ISRCTN30012997 [172] | Wrong comparator |
| ISRCTN10309022 [173] | Wrong intervention (lactose-free, chickpea flour) |
| Iuel-Brockdorf 2015 [174] | Wrong intervention (lipid-based nutrient supplements, non-fortified corn-soy blended flours) |
| Iuel-Brockdorf 2016 [175] | Wrong intervention (lipid-based nutrient supplements, non-fortified corn-soy blended flours) |
| Jaeggi 2015 [176] | Wrong intervention (home-fortified maize porridge) |
| Jahari 2000 [177] | Wrong intervention (milk plus micronutrients) |
| Jalla 2002 [178] | Wrong comparator |
| Javan 2017 [179] | Wrong intervention (multivitamin / mineral supplement) |

| | |
|---|---|
| Javaid 1991 [180] | Wrong population (aged <6 months) |
| Javaid 1991 [181] | Duplicate |
| Jilcott 2010 [182] | Wrong study design |
| John 1993 [183] | Wrong intervention (gruels, without micronutrient fortification) |
| Jong-Mee 2005 [184] | Wrong intervention (fortified chewiness) |
| Kaimila 2019 [185] | Wrong intervention (supplemental legumes) |
| Kajjura 2019 [186] | Wrong comparator |
| Kajjura 2020 [187] | Wrong comparator |
| Kalavi 1996 [188] | Wrong intervention |
| Kalhoff 2020 [189] | Wrong intervention |
| Kampstra 2018 [190] | Wrong comparator |
| Karakochuk 2012 [191] | Wrong comparator |
| Kekalih 2019 [192] | Wrong comparator |
| King 2007 [193] | Wrong intervention (highdiastase malted barley) |
| Kodkany 2013 [194] | Wrong population (aged > 23 months) |
| Konyole 2013 [195] | Wrong intervention (“Winfood Classic” vs. “Winfood Lite” vs. Corn Soy Blend Plus) |
| Konyole 2017 [196] | Wrong comparator |
| Konyole 2019 [197] | Wrong comparator |
| Krebs 2006 [198] | Wrong comparator |
| Krebs 2012 [199] | Wrong comparator |
| Krebs 2012 [200] | Wrong comparator |
| Krebs 2013 [201] | Wrong comparator |
| Krebs 2013 [202] | Wrong intervention (MNP) |
| Kuusipalo 2006 [203] | Wrong comparator |
| Laboratorios 2021 [204] | Wrong intervention (probiotics) |
| Lachat 2006 [205] | Wrong intervention (processing to improve protein digestibility) |
| LaGrone 2012 [206] | Wrong comparator |
| Lakkam 2014 [207] | Wrong study design |
| Langendorf 2014 [208] | Wrong comparator |
| Langlois 2020 [209] | Wrong comparator |
| Laylo-NavarroCelestinaRaquel 2011 [210] | Wrong intervention (non-fortified food) |
| Leroy 2020 [211] | Wrong intervention (micronutrient supplements) |
| Leroy 2021 [212] | Wrong intervention (different timing and duration of feeding) |
| Libuda 2016 [213] | Wrong intervention (food with rapeseed oil or oily fish) |
| Li 2015 [214] | Wrong population (aged > 23 months) |
| Lin 2008 [215] | Wrong comparator |
| Lind 2003 [216] | Wrong intervention (phytate-reduced products) |
| Lind 2004 [217] | Wrong intervention (phytate-reduced products) |
| Lind 2019 [218] | Wrong intervention (protein-reduced food) |
| LitkowskiPe 2016 [219] | Wrong intervention (non-fortified product) |
| Lo 2011 [220] | Wrong intervention (iron-fortified porridge combined with a liquid multivitamin supplement) |
| Long 2012 [221] | Wrong study design |
| LopezdeRomana 2005 [222] | Wrong population (aged > 23 months) |
| Ly 2006 [223] | Wrong comparator |
| Macharia-Mutie 2012 [224] | Wrong intervention (porridge with amaranth or MNP) |
| Macharia-Mutie 2013 [225] | Wrong intervention (porridge with amaranth or MNP) |

| | |
|---|---|
| Macharia-Mutie 2015 [226] | Wrong intervention (porridge with amaranth or MNP) |
| Manno 2011 [227] | Wrong comparator |
| Mahalanabis 1993 [228] | Wrong intervention |
| Mallard 2014 [229] | Wrong study design |
| Mamiro 2004 [230] | Wrong intervention (processed complementary food) |
| Manary 2004 [231] | Wrong comparator |
| Mank 2011 [232] | Wrong intervention (zinc supplement) |
| Manno 2012 [233] | Wrong comparator (richly fortified vs. basal fortified porridge) |
| Marron 2015 [234] | Wrong comparator |
| MartinezMartinez 2009 [235] | Wrong study design |
| Martorell 2020 [236] | Wrong study design |
| Masuda 2019 [237] | Wrong intervention (spirulina powder ) |
| Masuda 2019 [238] | Wrong intervention (spirulina powder) |
| Matilsky 2009 [239] | Wrong comparator |
| Maust 2015 [240] | Wrong intervention (integrated management of malnutrition) |
| McDonald 2019 [241] | Wrong comparator |
| McGill 2014 [242] | Wrong comparator |
| McGill 2015 [243] | Wrong population (aged > 23 months) |
| MedecinsSansFrontieres 2012 [244] | Wrong intervention (MNP) |
| MeeksGardner 1995 [245] | Wrong intervention (milk-based supplement) |
| Menon 2007 [246] | Wrong intervention (MNP) |
| Miles 1987 [247] | Wrong population |
| Mize 1995 [248] | Wrong intervention (formula) |
| Moore 2003 [249] | Wrong intervention (fructo-oligosaccharide-supplemented infant cereal) |
| Morales 2008 [250] | Wrong comparator |
| Moursi 2003 [251] | Wrong intervention (amylase) |
| Nane 2019 [252] | Wrong comparator |
| NCT01224535 [253] | Wrong intervention (porridge enriched with amaranth) |
| NCT02162238 [254] | Wrong population (aged > 23 months) |
| NCT01790048 [255] | Wrong intervention (non-fortified food) |
| NCT04334538 [256] | Wrong comparator |
| NCT00998517 [257] | Wrong comparator |
| NCT01552512 [258] | Wrong intervention (Nutributter) |
| NCT01785680 [259] | Wrong intervention (integrated protocol) |
| NCT02053857 [260] | Wrong comparator |
| NCT02375503 [261] | Wrong population (aged > 23 months) |
| NCT00822380 [262] | Wrong comparator |
| NCT01817634 [263] | Wrong intervention (fish oil capsule) |
| NCT00890695 [264] | Wrong comparator |
| NCT00631046 [265] | Wrong intervention (fish oil) |
| NCT01593969 [266] | Wrong intervention (n-3 PUFA enriched food) |
| NCT01790542 [267] | Wrong population (aged <6 months) |
| NCT03355222 [268] | Wrong intervention (non-fortified food) |
| NCT03385590 [269] | Wrong intervention (non-fortified food) |
| NCT03597061 [270] | Wrong intervention (behavioral) |
| NCT02221063 [271] | Wrong population (aged > 23 months) |
| NCT03041103 [272] | Wrong study design |
| NCT03175003 [273] | Wrong population (aged > 23 months) |

| | |
|---|---|
| NCT02208609 [274] | Wrong intervention (maize tortillas with Amaranth) |
| NCT02142647 [275] | Wrong intervention (non-fortified food) |
| NCT04137445 [276] | Wrong intervention (non-fortified food) |
| NCT00653705 [277] | Wrong intervention (probiotic bacteria BB12) |
| NCT01282788 [278] | Wrong comparator |
| NCT03355287 [279] | Wrong intervention (iron drops) |
| NCT01184716 [280] | Wrong population (aged > 23 months) |
| NCT01097889 [281] | Wrong comparator |
| NCT01001871 [282] | Wrong intervention (MNP) |
| NCT01455636 [283] | Wrong intervention (MNP) |
| NCT02192892 [284] | Wrong comparator |
| NCT01061307 [285] | Wrong population (aged > 23 months) |
| NCT01573013 [286] | Wrong population (aged > 23 months) |
| NCT01321099 [287] | Wrong comparator |
| NCT01111864 [288] | Wrong intervention (iron and micronutrient supplement) |
| NCT01634945 [289] | Wrong comparator |
| NCT02176759 [290] | Wrong population (aged > 23 months) |
| NCT02437955 [291] | Wrong population (aged > 23 months) |
| NCT02118402 [292] | Wrong intervention (MNP) |
| NCT03894358 [293] | Wrong intervention (prebiotic mixture) |
| NCT01423162 [294] | Wrong population (aged > 23 months) |
| NCT01418898 [295] | Wrong population (aged > 23 months) |
| NCT00571948 [296] | Wrong population (aged <6 months) |
| NCT04174846 [297] | Wrong intervention (non-fortified food) |
| NCT02079961 [298] | Wrong population (aged > 23 months) |
| NCT04564222 [299] | Wrong population (aged > 23 months) |
| NCT02847962 [300] | Wrong comparator |
| NCT01724073 [301] | Wrong comparator |
| NCT02165956 [302] | Wrong intervention (cereal with prebiotics, probiotics, vegetable proteins) |
| NCT03617575 [303] | Wrong intervention (iron supplement) |
| NCT02257437 [304] | Wrong comparator |
| NCT02257762 [305] | Wrong comparator |
| NCT02078271 [306] | Wrong intervention (food-based dietary guidelines) |
| NCT03474276 [307] | Wrong comparator |
| NCT01115647 [308] | Wrong comparator |
| NCT03752762 [309] | Wrong population (aged <6 months) |
| NCT03399617 [310] | Wrong population (aged <6 months) |
| NCT00867867 [311] | Wrong intervention (iron supplementation) |
| NCT04099849 [312] | Wrong population (aged > 23 months) |
| NCT01553877 [313] | Wrong intervention (non-fortified product) |
| NCT01634009 [314] | Wrong intervention (non-fortified product) |
| NCT02185196 [315] | Wrong intervention (vitamin D supplementation) |
| NCT04099849 [316] | Wrong population (aged > 23 months) |
| NCT03258385 [317] | Wrong population (aged > 23 months) |
| NCT04015999 [318] | Wrong intervention (non-fortified food) |
| NCT03084731 [319] | Wrong intervention (non-fortified food) |
| NCT01562379 [320] | Wrong comparator |
| NCT01751009 [321] | Wrong intervention (vitamin A supplement) |
| NCT02073149 [322] | Wrong intervention (point-of-care fortification) |
| NCT02435524 [323] | Wrong population (aged > 23 months) |

| | |
|---|---|
| NCT03038633 [324] | Wrong population (aged > 23 months) |
| NCT00131222 [325] | Wrong comparator |
| NCT04250896 [326] | Wrong intervention (behavioral) |
| NCT00944398 [327] | Wrong intervention (iron-fortified porridge combined with a liquid multivitamin supplement) |
| NCT01783067 [328] | Wrong population (aged > 23 months) |
| NCT00760890 [329] | Wrong comparator |
| NCT00841061 [330] | Wrong comparator |
| NCT00098202 [331] | Wrong population (aged > 23 months) |
| NCT01898871 [332] | Wrong comparator |
| NCT03549156 [333] | Wrong comparator |
| Nestel 2004 [334] | Wrong population (aged > 23 months) |
| NCT03754543 [335] | Wrong comparator |
| NCT04483453 [336] | Wrong intervention (different feeding regimens) |
| NCT04766346 [337] | Wrong study design |
| NCT03111927 [338] | Wrong population (aged <6 months) |
| Neufeld 2019 [339] | Wrong comparator |
| NCT03181178 [340] | Wrong intervention (MNP) |
| Nicklas 2020 [341] | Wrong study design |
| Nikiema 2014 [342] | Wrong comparator |
| Nikiema 2021 [343] | Wrong intervention (non-fortified food) |
| North-WestUniversity 2016 [344] | Wrong comparator |
| Nozari 2015 [345] | Wrong population (aged > 23 months) |
| Nurhasan 2018 [346] | Wrong comparator |
| Obatolu 2003 [347] | Wrong population (aged > 23 months) |
| Oelofse 2003 [348] | Wrong comparator |
| Ordiz 2020 [349] | Wrong intervention (legume supplementation) |
| Orsango 2019 [350] | Wrong population (aged > 23 months) |
| Osendarp 2002 [351] | Wrong population |
| Ouedraogo 2010 [352] | Wrong intervention (micronutrient supplement) |
| Owino 2007 [353] | Wrong intervention (alpha-amylase) |
| Owino 2011 [354] | Wrong comparator |
| Owino 2013 [355] | Wrong comparator |
| Owino 2015 [356] | Wrong comparator |
| PACTR201604001584278 [357] | Wrong intervention (precooked maize-sorghum flour) |
| PACTR201809662822990 [358] | Wrong comparator |
| Pactr 2019 [359] | Wrong comparator |
| Palmer 2018 [360] | Wrong population (aged > 23 months) |
| Patel 2005 [361] | Wrong comparator |
| Paul 2007 [362] | Wrong intervention (non-fortified food) |
| Phu 2010 [363] | Wrong population (aged <6 months) |
| Phu 2012 [364] | Wrong population (aged <6 months) |
| Phuka 2008 [365] | Wrong comparator |
| Phuka 2009 [366] | Wrong intervention (LNS) |
| Phuka 2009 [367] | Wrong comparator |
| Picciano 1980 [368] | Wrong intervention (formula) |
| Pollitt 2002 [369] | Wrong intervention (micronutrient supplement) |

| | |
|---|---|
| Purwestri 2012 [370] | Wrong intervention (RUF-Nias biscuits) |
| Pynaert 2006 [371] | Wrong study design |
| Qasem 2017 [372] | Wrong comparator |
| Rahman 1994 [373] | Wrong intervention (amylase) |
| Rahman 1997 [374] | Wrong intervention (amylase) |
| Rahman 1997 [375] | Wrong intervention (amylase) |
| Ramirez 2013 [376] | Wrong study design |
| Ramirez-Luzuriaga 2016 [377] | Wrong intervention (Powdered fortified milk) |
| Rao 1992 [378] | Wrong intervention (sweet ready mix with amylase) |
| Rim 2008 [379] | Wrong intervention (point-of-use fortification) |
| Rivera 1991 [380] | Wrong intervention (Atole, Fresco) |
| Rivera 2002 [381] | Wrong intervention (Atole, Fresco) |
| Roberts 2017 [382] | Wrong comparator |
| Roberts 2020 [383] | Wrong comparator |
| Roediger 2020 [384] | Wrong intervention (protein quality optimized RUSF) |
| Rosado 2010 [385] | Wrong comparator |
| Ruel 1997 [386] | Wrong intervention (liquid preparation zinc) |
| Safaa 2003 [387] | Wrong study design |
| Sako 2018 [388] | Wrong study design |
| Salinas-Pielago 1998 [389] | Wrong population (aged > 23 months) |
| Samadpour 2009 [390] | Wrong intervention (MNP) |
| Sandjaja 2015 [391] | Wrong study design |
| Sarojini 1999 [392] | Wrong study design |
| Sato 2017 [393] | Wrong intervention (MNP) |
| Sayyad-Neerkorn 2015 [394] | Wrong comparator |
| Sazawal 2014 [395] | Wrong comparator |
| Scherbaum 2015 [396] | Wrong comparator |
| Schlossman 2015 [397] | Wrong intervention (non-fortified food) |
| Schlossman 2017 [398] | Wrong study design |
| Schlossman 2018 [399] | Wrong intervention (non-fortified food) |
| Schroeder 1995 [400] | Wrong intervention (highenergy, high-protein beverage) |
| Schumann 2009 [401] | Wrong intervention (foodLET: chewable, flavored multiple-micronutrient vehicle that was a hybrid of a food and a tablet) |
| Schwartz 2009 [402] | Wrong intervention (n-6 linoleic acid) |
| Seal 2008 [403] | Wrong study design |
| Shaikh 2020 [404] | Wrong comparator |
| Shamah-Levy 2008 [405] | Wrong comparator |
| Shamim 2015 [406] | Wrong comparator |
| Shen 2017 [407] | Wrong comparator |
| Shen 2017 [408] | Wrong comparator |
| Sheng 2019 [409] | Duplicate |
| Shewade 2013 [410] | Wrong intervention (non-fortified food) |
| Sigh 2018 [411] | Wrong intervention (non-fortified food) |
| Simondon 1996 [412] | Wrong population (aged > 23 months) |
| Simpore 2006 [413] | Wrong intervention (Spirulina, Misola) |
| Singh 2010 [414] | Wrong intervention (fortified cereal-milk supplement) |
| Skau 2013 [415] | Wrong comparator |

| | |
|---|---|
| Skau 2013 [416] | Wrong comparator |
| Skau 2015 [417] | Wrong comparator |
| Stephenson 2017 [418] | Wrong intervention (cowpea or common bean flour) |
| Stobaugh 2016 [419] | Wrong intervention (soy vs. way) |
| Stobaugh 2017 [420] | Wrong intervention (package of interventions) |
| Stookey 1967 [421] | Wrong population |
| Tampere 2006 [422] | Wrong comparator |
| Tampere 2008 [423] | Wrong comparator |
| Tano-Debrah 2019 [424] | Wrong study design |
| Tekale 2015 [425] | Wrong population (aged > 23 months) |
| Thakur 2016 [426] | Wrong intervention (foods with different composition) |
| Thakwalakwa 2010 [427] | Wrong intervention (corn-soy blend, Lipid-based nutrient supplements) |
| Thakwalakwa 2014 [428] | Wrong comparator |
| Tharrey 2017 [429] | Wrong intervention (behavioural) |
| TheMathileInstitutefortheAdvancementofHuman 2020 [430] | Wrong comparator |
| Tondeur 2004 [431] | Wrong intervention (lipid-based nutrient supplement, MNP) |
| Traore 2005 [432] | Wrong intervention (non-fortified food) |
| Traore 2013 [433] | Wrong intervention (processed fortified flours wih dried milk and without milk, Misola) |
| Trehan 2015 [434] | Wrong comparator |
| Tufts 2014 [435] | Wrong comparator |
| Tufts 2017 [436] | Wrong comparator |
| Tufts 2017 [437] | Wrong comparator |
| Tufts 2018 [438] | Wrong comparator |
| vanderKam 2012 [439] | Wrong comparator |
| VanderWal 2018 [440] | Wrong intervention (aloe-enriched, whey protein drink) |
| VanHoan 2009 [441] | Wrong intervention (Favina and Favilase gruels) |
| Varea 2011 [442] | Wrong study design |
| Vega 2016 [443] | Wrong intervention (food supplements: Nutrisano, Vitanino) |
| Verkaik-Kloosterman 2017 [444] | Wrong study design |
| Verna [445] | Wrong comparator |
| Villanueva 2016 [446] | Wrong study design |
| Viseshakul 1979 [447] | Duplicate |
| Vray 2018 [448] | Wrong intervention (flour with prebiotic) |
| Vuongle 2002 [449] | Wrong population (aged > 23 months) |
| Walker 1996 [450] | Wrong intervention (home-fortification) |
| Wang 2013 [451] | Wrong comparator |
| Walter 1993 [452] | Wrong population (aged <6 months) |
| Whitfield 2016 [453] | Wrong population (aged > 23 months) |
| Whitfield 2017 [454] | Wrong population (aged > 23 months) |
| Whitfield 2016 [453] | Wrong population (aged > 23 months) |
| Westcott 2011 [455] | Wrong comparator |
| Women's 2010 [456] | Wrong comparator |
| Yeung 2000 [457] | Wrong comparator |
| Ying 1956 [458] | Wrong population (aged > 23 months) |
| Yu 2013 [459] | Wrong intervention (education and supplementation) |
| Yuliarti 2017 [460] | Wrong comparator |

| | |
|---|---|
| Zakaria 2019 [461] | Wrong intervention (formulas and Moringa Oleifera Leaf Powder) |
| Zakaria 2020 [462] | Wrong intervention (Moringa Oleifera Leaf Powder) |
| Zavaleta 2011 [463] | Wrong intervention (milk with protein) |
| Zhang 2016 [464] | Wrong intervention (food supplement+health education) |
| Zhichien 1956 [465] | Wrong intervention (fortification with lysine) |
| Ziegler 2009 [466] | Wrong comparator |
| Ziegler 2011 [467] | Wrong study design |
| Ziegler 2011 [468] | Wrong comparator |
| Zyba 2019 [469] | Wrong intervention (lipid-based nutrient supplement) |

3 duplicates are not listed.

## References


1. Dewey KG, Cohen RJ, Rivera LL, Brown KH. Effects of age of introduction of complementary foods on iron status of breast-fed infants in Honduras including commentary by Garza C and Frongillo EA Jr. *American Journal of Clinical Nutrition*; **67**(5): 878-16.
2. Aakko J, Grzeskowiak L, Asukas T, et al. Lipid-based Nutrient Supplements Do Not Affect Gut Bifidobacterium Microbiota in Malawian Infants: A Randomized Trial. *J Pediatr Gastroenterol Nutr* 2017; **64**(4): 610-5.
3. Aaron GJ, Lo NB, Hess SY, et al. Acceptability of Complementary Foods and Breads Prepared from Zinc-Fortified Cereal Flours among Young Children and Adults in Senegal. *J Food Sci* 2011; **76**(1): S56-S62.
4. Aboud FE, Akhter S. A cluster-randomized evaluation of a responsive stimulation and feeding intervention in bangladesh. *Pediatrics* 2011; **127**(5): e1191-7.
5. Ackatia-Armah RS, McDonald C, Doumbia S, Brown KH. Effect of selected dietary regimens on recovery from moderate acute malnutrition in young Malian children. *FASEB journal* 2012; **26**.
6. Ackatia-Armah RS, Mc Donald CM, Doumbia S, Earhardt J, Peerson J, Brown KH. Effect of selected dietary supplements on micronutrient status during recovery from moderate acute malnutrition in young malian children. *Annals of nutrition & metabolism* 2013; **63**: 842.
7. Ackatia-Armah RS, McDonald CM, Doumbia S, Erhardt JG, Hamer DH, Brown KH. Malian children with moderate acute malnutrition who are treated with lipid-based dietary supplements have greater weight gains and recovery rates than those treated with locally produced cereal-legume products: a community-based, cluster-randomized trial. *Am J Clin Nutr* 2015; **101**(3): 632-45.
8. Actrn. Evaluation of the efficacy of different strategies to treat anemia in Mexican children. *http://wwwwhoint/trialsearch/Trial2aspx?TrialID=ACTRN12609000061235* 2009.
9. Actrn. Seeding throUgh FeediNg: nourishing the infant microbiome to support immune health. *http://wwwwhoint/trialsearch/Trial2aspx?TrialID=ACTRN12620000026921* 2020.
10. Agapova SE, Stephenson KB, Divala O, et al. Additional Common Bean in the Diet of Malawian Children Does Not Affect Linear Growth, but Reduces Intestinal Permeability. *J Nutr* 2018; **148**(2): 267-74.
11. Ahmad A, Madanijah S, Dwiriani CM, Kolopaking R. Nutrition education using food monitoring card and multi-nutrient biscuit interventions improving nutritional and iron status in undernourished children aged 6-23 months: A cluster randomized control trial in Aceh, Indonesia. *Annals of Nutrition and Metabolism* 2019; **75**(3): 316.
12. Ahmad A, Madanijah S, Dwiriani CM, Kolopaking R. Effect of Nutrition Education and Multi-Nutrient Biscuit Interventions on Nutritional and Iron Status: A Cluster Randomized Control Trial on Undernourished Children Aged 6-23 Months in Aceh, Indonesia. *J Nutr Sci Vitaminol (Tokyo)* 2020; **66**(Supplement): S380-S90.
13. Ahmed T, Choudhury N, Hossain MI, et al. Development and acceptability testing of ready-to-use supplementary food made from locally available food ingredients in Bangladesh. *BMC Pediatr* 2014; **14**: 164.
14. Ahmed T, Islam M, Choudhury N, et al. Results with Complementary Food Using Local Food Ingredients. *Nestle Nutr Inst Workshop Ser* 2017; **87**: 103-13.
15. Akalu G, Taffesse S, Gunaratna NS, et al. The effectiveness of quality protein maize in improving the nutritional status of young children in the Ethiopian highlands. *Food & Nutrition Bulletin* 2010; **31**(3): 418-30.
16. Ortega Alemán EdC, Coulson Romero AJ, Ordóñez Argueta LI, Pachón H. Efectos de la ingesta de maíz de alta calidad de proteína (QPM) versus maíz convencional en el crecimiento y la morbilidad de niños nicaragüenses desnutridos de 1 a 5 años de edad. *Arch latinoam nutr* 2008; **58**(4): 377-85.
17. Amthor RE, Cole SM, Manary MJ. The use of home-based therapy with ready-to-use therapeutic food to treat malnutrition in a rural area during a food crisis. *Journal of the American Dietetic Association* 2009; **109**(3): 464-7.
18. Anorve-Valdez G, Quezada-Sanchez AD, Mejia-Rodriguez F, Garcia-Guerra A, Neufeld LM. Fortified food supplementation in children with reduced dietary energy and micronutrients intake in Southern Mexico. *Nutr J* 2018; **17**(1): 76.
19. Arcanjo FPN, da Costa Rocha TC, Arcanjo CPC, Santos PR. Micronutrient Fortification at Child-Care Centers Reduces Anemia in Young Children. *J Diet Suppl* 2019; **16**(6): 689-98.
20. Argaw A, Wondafrash M, Bouckaert KP, et al. Effects of n-3 long-chain PUFA supplementation to lactating mothers and their breastfed children on child growth and morbidity: A 2 x 2 factorial randomized controlled trial in rural Ethiopia. *American journal of clinical nutrition* 2018; **107**(3): 454-64.
21. Ariff SS, Soofi SSB, Krebs NN, Westcott JJ, Bhatti ZZ, Bhutta ZZA. Exchangeable zn pool size and zn absorption from sprinkles and traditional foods in pakistani infants/toddlers. *Annals of Nutrition and Metabolism* 2013; **63**: 257-8.
22. Arsenault JE, Havel PJ, Lopez de Romana D, Penny ME, Van Loan MD, Brown KH. Longitudinal measures of circulating leptin and ghrelin concentrations are associated with the growth of young Peruvian children but are not affected by zinc supplementation. *Am J Clin Nutr* 2007; **86**(4): 1111-9.

23. Arsenault JE, Lopez de Romana D, Penny ME, Van Loan MD, Brown KH. Additional zinc delivered in a liquid supplement, but not in a fortified porridge, increased fat-free mass accrual among young Peruvian children with mild-to-moderate stunting. *J Nutr* 2008; **138**(1): 108-14.
24. Arsenault JE, De Romaña DL, Penny M, Brown KH. Dietary pattern with high dairy intake is associated with linear growth in Peruvian infants. *FASEB Journal* 2016; **30**.
25. Arya TU. Assessment of growth and development in infants with Shashtikshalyadi Churna as weaning food: A clinical study. *International Journal of Research in Ayurveda and Pharmacy* 2014; **5**(4): 439-43.
26. As'ad S, Yusuf I. The effects of zinc supplementation on the TNF-α profile and diarrhea in severely malnourished children of low income family. 2003.
27. Association for the S, Prevention of HA. Comparison of the Effect of Two Treatments on the Nutritional and Micronutrient Status of Malnourished Children. 2013.
28. Awasthi S, Reddy NU, Mitra M, et al. Micronutrient-fortified infant cereal improves Hb status and reduces iron-deficiency anaemia in Indian infants: an effectiveness study. *Br J Nutr* 2020; **123**(7): 780-91.
29. Badau MH, Bristone C, Igwebuike JU, Danbaba N. Production, viscosity, microbiological quality and sensory properties of complementary food blends of improved rice cultivars, soybean and sorghum malt. *Pakistan Journal of Nutrition* 2016; **15**(9): 849-56.
30. Bagni UV, Baião MR, Santos MMAdS, Luiz RR, Veiga GVd. Efeito da fortificação semanal do arroz com ferro quelato sobre a freqüência de anemia e concentração de hemoglobina em crianças de creches municipais do Rio de Janeiro, Brasil. *Cad saúde pública* 2009; **25**(2): 291-302.
31. Bajaj M, Dubey AP, Nagpal J, Singh PK, Sachdev HP. Short-term effect of oil supplementation of complementary food on total ad libitum consumption in 6- to 10-month-old breastfed Indian infants. *J Pediatr Gastroenterol Nutr* 2005; **41**(1): 61-5.
32. Baskaran V, Mahadevamma, Malleshi NG, Shankara R, Lokesh BR. Acceptability of supplementary foods based on popped cereals and legumes suitable for rural mothers and children. *Plant Food Hum Nutr* 1999; **53**(3): 237-47.
33. Bauserman M, Lokangaka A, Gado J, et al. A cluster-randomized trial determining the efficacy of caterpillar cereal as a locally available and sustainable complementary food to prevent stunting and anaemia. *Public Health Nutr* 2015; **18**(10): 1785-92.
34. Becroft T, Bailey KV. Supplementary feeding trial in New Guinea Highland infants. *J Trop Pediatr Afr Child Health* 1965; **11**(2): 28-34.
35. Beinner MA, Velasquez-Melendez G, Pessoa MC, Greiner T. Iron-fortified rice is as efficacious as supplemental iron drops in infants and young children. *J Nutr* 2010; **140**(1): 49-53.
36. Bergmann R, Bergler H, Moshoudis E, Bergmann E, Lachmann E, Bergmann KE. [Preventing iron deficiency in breast-fed infants by suitable supplementary food. A prospective, controlled study]. *Monatsschr Kinderheilkd* 1989; **137**(12): 775-9.
37. Bernal MJ, Periago MJ, Martinez R, et al. Effects of infant cereals with different carbohydrate profiles on colonic function--randomised and double-blind clinical trial in infants aged between 6 and 12 months--pilot study. *Eur J Pediatr* 2013; **172**(11): 1535-42.
38. Bhandari N, Mohan SB, Bose A, et al. Efficacy of three feeding regimens for home-based management of children with uncomplicated severe acute malnutrition: a randomised trial in India. *BMJ glob* 2016; **1**(4): e000144.
39. Bhargava A. Iron status, malaria parasite loads and food policies: evidence from sub-Saharan Africa. *Econ Hum Biol* 2013; **11**(1): 108-12.
40. Bishop WB, Laubscher I, Labadarios D, Rehder P, Louw ME, Fellingham SA. Effect of vitamin-enriched bread on the vitamin status of an isolated rural community--a controlled clinical trial. *South African medical journal = Suid-Afrikaanse tydskrif vir geneeskunde* 1996; **86**(4 Suppl): 458-62.
41. Bisimwa G, Owino VO, Bahwere P, et al. Randomized controlled trial of the effectiveness of a soybean-maize-sorghum-based ready-to-use complementary food paste on infant growth in South Kivu, Democratic Republic of Congo. *Am J Clin Nutr* 2012; **95**(5): 1157-64.
42. Boateng L OAAMS-AM. Measuring blood retinol concentrations of infants fed with complementary foods fortified with Moringa Oleifera leaf powder-a pilot study. *Annals of nutrition & metabolism* 2017; **71**: 655.
43. Boateng L, Ashley I, Ohemeng A, Asante M, Steiner-Asiedu M. Improving Blood Retinol Concentrations with Complementary Foods Fortified with Moringa oleifera Leaf Powder - A Pilot Study. *Yale J Biol Med* 2018; **91**(2): 83-94.
44. Boateng L, Quarpong W, Ohemeng A, Asante M, Steiner-Asiedu M. Effect of complementary foods fortified with Moringa oleifera leaf powder on hemoglobin concentration and growth of infants in the Eastern Region of Ghana. *Food sci* 2019; **7**(1): 302-11.
45. Borg B SDMSGMCCLABJWF. Efficacy of a locally-produced multiple micronutrient-fortified ready-to-use supplementary food (RUSF) for children under two years in Cambodia. *Annals of nutrition & metabolism* 2017; **71**: 329.

46. Borg B, Mihrshahi S, Griffin M, et al. Randomised controlled trial to test the effectiveness of a locally-produced ready-to-use supplementary food (RUSF) in preventing growth faltering and improving micronutrient status for children under two years in Cambodia: a study protocol. *Nutr J* 2018; **17**(1): 39.
47. Borg B, Mihrshahi S, Griffin M, et al. Acceptability of locally-produced Ready-to-Use Supplementary Food (RUSF) for children under two years in Cambodia: A cluster randomised trial. *Matern Child Nutr* 2019; **15**(3): e12780.
48. Boston U. A Randomized Controlled Trial Testing The Effect Of A Multi-Nutrient Fortified Juice. 2008.
49. Bouhouch RR, Fadeli SE, Sedki A, et al. Reducing Lead Burden and Improving Iron Status with Iron Fortification Using NaFeEDTA: A Double- Blind, Randomized, Placebo- Controlled Intervention Trial (RCT) in Lead-exposed Moroccan Children. 2015.
50. Brett NR, Lavery P, Agellon S, et al. Vitamin D Status and Immune Health Outcomes in a Cross-Sectional Study and a Randomized Trial of Healthy Young Children. *Nutrients* 2018; **10**(6): 27.
51. Brnić M, Hurrell RF, Songré-Ouattara LT, et al. Effect of phytase on zinc absorption from a millet-based porridge fed to young Burkinabe children. *European Journal of Clinical Nutrition* 2017; **71**(1): 137-41.
52. Brown KH, Lopez de Romana D, Arsenault JE, Peerson JM, Penny ME. Comparison of the effects of zinc delivered in a fortified food or a liquid supplement on the growth, morbidity, and plasma zinc concentrations of young Peruvian children. *Am J Clin Nutr* 2007; **85**(2): 538-47.
53. Campbell R, Hurley K, Shamim AA, et al. Children receiving complementary food supplements (CFS) have higher nutrient intakes from home foods in jivita-4 trial Rural Bangladesh. *FASEB journal* 2015; **29**(1 Meeting Abstracts).
54. Campbell R SKSSAHMSWLCP. An enteropathy score predicts subsequent length better than lactulose mannitol (L:M) ratio alone in children enrolled in a community-based randomized trial of complementary food supplements in rural Bangladesh. *FASEB journal* 2016; **30**.
55. Campbell RK, Hurley KM, Shamim AA, et al. Effect of complementary food supplementation on breastfeeding and home diet in rural Bangladeshi children. *Am J Clin Nutr* 2016; **104**(5): 1450-8.
56. Campbell RK, Schulze KJ, Shaikh S, et al. Biomarkers of Environmental Enteric Dysfunction Among Children in Rural Bangladesh. *J Pediatr Gastroenterol Nutr* 2017; **65**(1): 40-6.
57. Campbell RK, Hurley KM, Shamim AA, et al. Complementary Food Supplements Increase Dietary Nutrient Adequacy and Do Not Replace Home Food Consumption in Children 6-18 Months Old in a Randomized Controlled Trial in Rural Bangladesh. *J Nutr* 2018; **148**(9): 1484-92.
58. Campbell RK, Shaikh S, Schulze K, et al. Micronutrient and Inflammation Status Following One Year of Complementary Food Supplementation in 18-Month-Old Rural Bangladeshi Children: A Randomized Controlled Trial. *Nutrients* 2020; **12**(5): 18.
59. Capozzi L, Russo R, Bertocco F, Ferrara D, Ferrara M. Effect on haematological and anthropometric parameters of iron supplementation in the first 2 years of life. Risks and benefits. *Hematol* 2011; **16**(5): 261-4.
60. Carol H, Brac, Marywood U, Nutrition I, University of S. Iron-fortified Lentils to Improve Iron (Fe) Status in Bangladesh. 2019.
61. Cercamondi CI, Egli IM, Mitchikpe E, et al. Iron bioavailability from a lipid-based complementary food fortificant mixed with millet porridge can be optimized by adding phytase and ascorbic acid but not by using a mixture of ferrous sulfate and sodium iron EDTA. *J Nutr* 2013; **143**(8): 1233-9.
62. Chauhan HH, Javadekar BB, Jayswal AV, Thakkar PA, Parmar NT. Non-milk based local therapeutic feed plus home-based diet as compared to home-based diet alone for nutritional rehabilitation of severe acute malnutrition following discharge: A randomised clinical trial. *Journal of Clinical and Diagnostic Research* 2019; **13**(2): SC05-SC8.
63. Chavasit V, Porasuphatana S, Suthutvoravut U, Zeder C, Hurrell R. Iron bioavailability in 8-24-month-old Thai children from a micronutrient-fortified quick-cooking rice containing ferric ammonium citrate or a mixture of ferrous sulphate and ferric sodium ethylenediaminetetraacetic acid. *Matern Child Nutr* 2015; **11**: 179-87.
64. Chilenje Infant Growth N, Infection Study T. Micronutrient fortification to improve growth and health of maternally HIV-unexposed and exposed Zambian infants: a randomised controlled trial. *PLoS ONE* 2010; **5**(6): e11165.
65. Chisenga M SJBKKLFS. Determinants of infant feeding choices by Zambian mothers: A mixed quantitative and qualitative study. *Maternal & child nutrition* 2011; **7**(2): 148-59.
66. Chomba E, Westcott CM, Westcott JE, et al. Zinc absorption from biofortified maize meets the requirements of young rural Zambian children. *J Nutr* 2015; **145**(3): 514-9.
67. Choudhury N, Bromage S, Alam MA, et al. Intervention study shows suboptimal growth among children receiving a food supplement for five months in a slum in Bangladesh. *Acta Paediatr* 2016; **105**(10): e464-73.

68. Christian P, Shaikh S, Shamim AA, et al. Effect of Fortified Complementary Food Supplementation on Child Growth in Rural Bangladesh - a Cluster-Randomized Trial. *Faseb J* 2015; **29**: 1.
69. Christian P, Shaikh S, Shamim AA, et al. Effect of fortified complementary food supplementation on child growth in rural Bangladesh: a cluster-randomized trial. *Int J Epidemiol* 2015; **44**(6): 1862-76.
70. Cliffer I, Langlois B, Suri D, et al. Design and baseline characteristics of a study comparing four supplementary foods in the prevention of stunting and wasting among children 6-23 months in Burkina Faso. *FASEB Journal* 2017; **31**(1).
71. Cliffer IR, Nikiema L, Langlois BK, et al. Cost-Effectiveness of 4 Specialized Nutritious Foods in the Prevention of Stunting and Wasting in Children Aged 6-23 Months in Burkina Faso: A Geographically Randomized Trial. *Curr* 2020; **4**(2): nzaa006.
72. Cook JD, Reddy MB, Burri J, Juillerat MA, Hurrell RF. The influence of different cereal grains on iron absorption from infant cereal foods. *American Journal of Clinical Nutrition* 1997; **65**(4): 964-9.
73. Cornell U, Haitian Group for the Study of Kaposi's S, Opportunistic, Weill Medical College of Cornell U, University of V. Evaluation of an Infant Feeding Intervention for HIV-exposed Haitian Infants. 2010.
74. Cornell U, University SWs. Feeding Trial of Bio-fortified Pearl Millet. 2012.
75. Ctri. Study to see how good Ready to use therapeutic food is in treating uncomplicated severe acute malnourished under-five children in community settings. *http://wwwwhoint/trialsearch/Trial2aspx?TrialID=CTRI/2011/12/002259* 2011.
76. Ctri. To check how intake of iron rich baby food can help in increasing the level of iron in blood and reduce the risk of iron deficiency. 2017.
77. Ctri. Dharavi Nutrition Project. *http://wwwwhoint/trialsearch/Trial2aspx?TrialID=CTRI/2017/08/009260* 2017.
78. Cubero J, Chanclon B, Sanchez S, Rivero M, Rodriguez AB, Barriga C. Improving the quality of infant sleep through the inclusion at supper of cereals enriched with tryptophan, adenosine-5'-phosphate, and uridine-5'-phosphate. *Nutr Neurosci* 2009; **12**(6): 272-80.
79. Cuj M, Mazariegos M, Fischer E, Román AV. Aceptabilidad y uso en el hogar de un alimento complementario listo para consumir en el área rural de Guatemala. *Rev cient (Guatem)* 2016; **26**(1): [60]-[70].
80. Dahl W, Lungu E. P145 Feasibility and Acceptability of a Soy-Fiber-Maize Blend Complementary Food for Children Aged 6-36 Months in Malawi...Society for Nutrition Education and Behavior, 52nd Annual Conference, Nutrition Education: Rooted in Food, July 27-30, 2019, Orlando. *Journal of nutrition education and behavior* 2019; **51**: S97.
81. Daniels L TRWWSMFLAWBJTBJHJJSSHA. Impact of a baby-led approach to complementary feeding on iron intake and status at 12 months of age: A randomised controlled trial. *European journal of pediatrics* 2016; **175**(11): 1542.
82. Daniels L TRWGRSSSHJJWSMWBJTBJHA. Impact of a baby-led approach to complementary feeding on iron status at 12 months of age: A randomised controlled trial. *Nutrients* 2017; **9**(3).
83. Davidsson L, Kastenmayer P, Szajewska H, Hurrell RF, Barclay D. Iron bioavailability in infants from an infant cereal fortified with ferric pyrophosphate or ferrous fumarate. *Am J Clin Nutr* 2000; **71**(6): 1597-602.
84. Davidsson L, Adou P, Zeder C, Walczyk T, Hurrell R. The effect of retinyl palmitate added to iron-fortified maize porridge on erythrocyte incorporation of iron in African children with vitamin A deficiency. *British Journal of Nutrition* 2003; **90**(2): 337-43.
85. Davidsson L, Sarker SA, Jamil KA, Sultana S, Hurrell R. Regular consumption of a complementary food fortified with ascorbic acid and ferrous fumarate or ferric pyrophosphate is as useful as ferrous sulfate in maintaining hemoglobin concentrations >105 g/L in young Bangladeshi children. *Am J Clin Nutr* 2009; **89**(6): 1815-20.
86. de Almeida CAN, Crott GCl, Ricco RG, Del Ciampo LA, Dutra-de-Oliveira JE, Cantolini A. Control of iron-deficiency anaemia in Brazilian preschool children using iron-fortified orange juice. *Nutrition Research* 2003; **23**(1): 27-33.
87. de Almeida CA, Dutra-De-Oliveira JE, Crott GC, et al. Effect of fortification of drinking water with iron plus ascorbic acid or with ascorbic acid alone on hemoglobin values and anthropometric indicators in preschool children in day-care centers in Southeast Brazil. *Food Nutr Bull* 2005; **26**(3): 259-65.
88. de Almeida CA, De Mello ED, Ramos AP, Joao CA, Joao CR, Dutra-de-Oliveira JE. Assessment of drinking water fortification with iron plus ascorbic Acid or ascorbic Acid alone in daycare centers as a strategy to control iron-deficiency anemia and iron deficiency: a randomized blind clinical study. *J Trop Pediatr* 2014; **60**(1): 40-6.
89. Delimont NM, Alavi S, Lindshield B. New formulations for fortified-blended foods: The mffapp Tanzania efficacy trial. *FASEB Journal* 2017; **31**(1).
90. Delimont NM, Chanadang S, Joseph MV, et al. The MFFAPP Tanzania Efficacy Study Protocol: Newly Formulated, Extruded, Fortified Blended Foods for Food Aid. *Curr* 2017; **1**(5): e000315.

91. Delimont NM, Vahl CI, Kayanda R, et al. Complementary Feeding of Sorghum-Based and Corn-Based Fortified Blended Foods Results in Similar Iron, Vitamin A, and Anthropometric Outcomes in the MFFAPP Tanzania Efficacy Study. *Current Developments in Nutrition* 2019; **3**(6).
92. De Oliveira SMS, Costa M, Rivera MAA, et al. [Impact of a dietary supplement on the nutritional status of preschool children enrolled in day care centers]. *Revista de Nutricao* 2006; **19**(2): 169-76.
93. de Oliveira JE, Scheid MM, Desai ID, Marchini S. Iron fortification of domestic drinking water to prevent anemia among low socioeconomic families in Brazil. *Int J Food Sci Nutr* 1996; **47**(3): 213-9.
94. de Paula RA, Fisberg M. The use of sugar fortified with iron tris-glycinate chelate in the prevention of iron deficiency anemia in preschool children. *Arch Latinoam Nutr* 2001; **51**(1 Suppl 1): 54-9.
95. Dewan P, Kaur IR, Faridi MM, Agarwal KN. Cytokine response to dietary rehabilitation with curd (Indian dahi) & leaf protein concentrate in malnourished children. *The Indian journal of medical research* 2009; **130**(1): 31-6.
96. Dewan P, Kaur IR, Faridi MMA, Agarwal KN. Cytokine response to dietary rehabilitation with curd (Indian dahi) & leaf protein concentrate in malnourished children. 2009.
97. Dewey KG, Cohen RJ, Rivera LL, Brown KH. Effects of age of introduction of complementary foods on iron status of breast-fed infants in Honduras. *Am J Clin Nutr* 1998; **67**(5): 878-84.
98. Dewey KG, Cohen RJ, Brown KH. Exclusive breast-feeding for 6 months, with iron supplementation, maintains adequate micronutrient status among term, low-birthweight, breast-fed infants in Honduras. *J Nutr* 2004; **134**(5): 1091-8.
99. Dhingra U, Pradhan A, Menon VP, et al. Evaluating mode of delivery of iron or iron and zinc on iron status and iron stress markers. *Archives of disease in childhood* 2012; **97**: A299.
100. Dong C, Ge P, Ren X, et al. Prospective study on the effectiveness of complementary food supplements on improving status of elder infants and young children in the areas affected by Wenchuan earthquake. *PLoS ONE* 2013; **8**(9): e72711.
101. Drks. Effectiveness of Locally Produced Peanut/Milk spread and Newly Developed Ready-to-Use Foods (RUF) for Malnourished Children in Nias Island, Indonesia? http://wwwwhoint/trialsearch/Trial2aspx?TrialID=DRKS00006174 2014.
102. Dube K, Schwartz J, Mueller MJ, Kalhoff H, Kersting M. Complementary food with low (8%) or high (12%) meat content as source of dietary iron: a double-blinded randomized controlled trial. *Eur J Nutr* 2010; **49**(1): 11-8.
103. Duggan C, Penny ME, Hibberd P, et al. Oligofructose-supplemented infant cereal: 2 randomized, blinded, community-based trials in Peruvian infants. *Am J Clin Nutr* 2003; **77**(4): 937-42.
104. Duizer LM, Diana A, Rathomi HS, et al. An Acceptability Trial of Desiccated Beef Liver and Meat Powder as Potential Fortifiers of Complementary Diets of Young Children in Indonesia. *J Food Sci* 2017; **82**(9): 2206-12.
105. Dutradeoliveira JE, Ferreira JB, Vasconcellos VP, Marchini JS. DRINKING-WATER AS AN IRON CARRIER TO CONTROL ANEMIA IN PRESCHOOL-CHILDREN IN A DAY-CARE-CENTER. *J Am Coll Nutr* 1994; **13**(2): 198-202.
106. Ekbote VH, Khadilkar AV, Chiplonkar SA, Hanumante NM, Khadilkar VV, Mughal MZ. A pilot randomized controlled trial of oral calcium and vitamin D supplementation using fortified laddoos in underprivileged Indian toddlers. *Eur J Clin Nutr* 2011; **65**(4): 440-6.
107. Emel K, Sm. Ziauddin H, Melody C T, Shabina R, Noor Ahmad K, Stanley H Z. Home fortification with sprinkles [TM] to reduce childhood anaemia: lessons learned in North West Frontier Province Pakistan. 2006. p. 35-40.
108. Ernst J, Ettyang G, Neumann C. High nutrition biscuits as a supplement to increase animal protein in diets of HIV-infected kenyan women and their children. *Annals of Nutrition and Metabolism* 2013; **63**: 144.
109. Ernst J, Ettyang G, Neumann CG. High-nutrition biscuits to increase animal protein in diets of HIV-infected Kenyan women and their children: a study in progress. *Food Nutr Bull* 2014; **35**(4 Suppl): S198-204.
110. Faber M. Complementary foods consumed by 6-12-month-old rural infants in South Africa are inadequate in micronutrients. *Public Health Nutrition* 2005; **8**(4): 373-81.
111. Fatmah. MOCAF TEMPEH DATES BISCUIT FOR THE IMPROVEMENT NUTRITIONAL STATUS OF UNDERWEIGHT CHILDREN. *Int J GEOMATE* 2018; **15**(49): 155-61.
112. Ferreira HdS, Cavalcante SdA, Cabral Júnior CR, Paffer ATd. Efeitos do consumo da multimistura sobre o estado nutricional: ensaio comunitário envolvendo crianças de uma favela da periferia de Maceió, Alagoas, Brasil. *Rev bras saúde matern infant* 2008; **8**(3): 309-18.
113. Fhi, International Food Policy Research I, Project Concern International I. Evaluation of Integrating MIYCN Interventions in Existing CGPP of PCI. 2018.
114. Filteau S, Baisley K, Chisenga M, Kasonka L, Gibson RS. Provision of micronutrient-fortified food from 6 months of age does not permit HIV-exposed, uninfected Zambian children to catch up in growth to HIV-

unexposed children: a randomised controlled trial. *Journal of acquired immune deficiency syndromes (1999)* 2011; **56**(2): 166-75.
115. Filteau S, Baisley K, Chisenga M, Kasonka L, Gibson RS, Team CS. Provision of micronutrient-fortified food from 6 months of age does not permit HIV-exposed uninfected Zambian children to catch up in growth to HIV-unexposed children: a randomized controlled trial. *J Acquir Immune Defic Syndr* 2011; **56**(2): 166-75.
116. Fink G, Levenson R, Tembo S, Rockers PC. Home- and community-based growth monitoring to reduce early life growth faltering: an open-label, cluster-randomized controlled trial. *Am J Clin Nutr* 2017; **106**(4): 1070-7.
117. Finn K, Callen C, Bhatia J, Reidy K, Bechard LJ, Carvalho R. Importance of Dietary Sources of Iron in Infants and Toddlers: Lessons from the FITS Study. *Nutrients* 2017; **9**(7): 9.
118. Fleige LE, Sahyoun NR, Murphy SP. A New Simulation Model Estimates Micronutrient Levels to Include in Fortified Blended Foods Used in Food Aid Programs. *J Nutr* 2010; **140**(2): 355-65.
119. Food U, Nutrition S, Westat, University of California LA, University of California B. Feeding My Baby - A National WIC Study. 2018.
120. Friel JK, Qasem W, Cai C, et al. Iron and complementary feeding of breast-fed infants. *Free radical biology & medicine* 2013; **65**: S108-S9.
121. Friel J, Qasem W, Hossain Z, Jorgensen S. Iron and complementary feeding of breast-fed infants. *FASEB Journal* 2014; **28**(1).
122. Friel J, Azad E, Azad M, Qasem W, Cai C, Kafipour E. Iron and complementary feeding of breastfed infants: Impact on gut inflammation and microbiota. *FASEB journal* 2015; **29**(1 Meeting Abstracts).
123. Friel J. Effect of iron rich foods on gut oxidative status and microbiota of the newborn. *Free radical biology & medicine* 2016; **100**: S191.
124. Fuchs GJ, DeWier M, Hutchinson SW, et al. Impact of whole cow's milk with iron-fortified cereal or formula on dietary intake and iron status of infants 6-12 months of age. *American journal of clinical nutrition* 1991; **53**(5): P-18.
125. Fuchs George J, DeWier M, Hutchinson S, Doucet H, Suskind Robert M. Iron intake, occult blood loss, and iron status in infants 6-12 months fed formula or WCM + Fe-fortified cereal [abstract]. *Pediatric research* 1991; **29 Suppl**: 119A.
126. Fuchs GJ, Farris RP, DeWier M, et al. Iron status and intake of older infants fed formula vs cow milk with cereal. *Am J Clin Nutr* 1993; **58**(3): 343-8.
127. Galpin L, Thakwalakwa C, Phuka J, et al. Breast milk intake is not reduced more by the introduction of energy dense complementary food than by typical infant porridge. *J Nutr* 2007; **137**(7): 1828-33.
128. Gannon B, Kaliwile C, Arscott S, et al. Biofortified orange maize is as efficacious as a vitamin A supplement in Zambian children even on the background of high liver reserves of vitamin A. *FASEB journal* 2014; **28**(1 SUPPL. 1).
129. Gannon B, Kaliwile C, Arscott SA, et al. Biofortified orange maize is as efficacious as a vitamin A supplement in Zambian children even in the presence of high liver reserves of vitamin A: a community-based, randomized placebo-controlled trial. *Am J Clin Nutr* 2014; **100**(6): 1541-50.
130. García-Guerra A, Rivera-Dommarco J, Neufeld L, Domínguez-Islas CP. Effect of three supplements with equal micronutrient content on serum zinc concentrations in Mexican children. *The FASEB Journal* 2009; **23**(S1).
131. Gartner A, Maire B, Traissac P, Kameli Y, Delpeuch F. Determinants of nutrition improvement in a large-scale urban project: a follow-up study of children participating in the Senegal Community Nutrition Project. *Public Health Nutr* 2006; **9**(8): 982-90.
132. Geltman PL, Hironaka LK, Mehta SD, et al. Iron supplementation of low-income infants: a randomized clinical trial of adherence with ferrous fumarate sprinkles versus ferrous sulfate drops. *J Pediatr* 2009; **154**(5): 738-43.
133. Gershoff SN, McGandy RB, Suttapreyasri D, et al. Nutrition studies in Thailand. II. Effects of fortification of rice with lysine, threonine, thiamin, riboflavin, vitamin A, and iron on preschool children. *AMJCLINNUTR* 1977; **30**(7): 1185-95.
134. Ghosh S, Strutt N, Otoo GE, et al. Effect of the provision of a macro- and micro-nutrient fortified complementary food supplement on nutritional status of Ghanaian infants. *FASEB Journal* 2017; **31**(1).
135. Ghosh SA, Strutt NR, Otoo GE, et al. A macro- and micronutrient-fortified complementary food supplement reduced acute infection, improved haemoglobin and showed a dose-response effect in improving linear growth: a 12-month cluster randomised trial. *J* 2019; **8**: e22.
136. Gibson RS, Kafwembe E, Mwanza S, et al. A micronutrient-fortified food enhances iron and selenium status of Zambian infants but has limited efficacy on zinc. *Journal of Nutrition* 2011; **141**(5): 935-43.

137. Glinz D, Hurrell RF, Ouattara M, et al. The effect of iron-fortified complementary food and intermittent preventive treatment of malaria on anaemia in 12- to 36-month-old children: a cluster-randomised controlled trial. *Malar J* 2015; **14**: 347.
138. Glinz D, Wegmuller R, Ouattara M, et al. Iron Fortified Complementary Foods Containing a Mixture of Sodium Iron EDTA with Either Ferrous Fumarate or Ferric Pyrophosphate Reduce Iron Deficiency Anemia in 12-to 36-Month-Old Children in a Malaria Endemic Setting: A Secondary Analysis of a Cluster-R. *Nutrients* 2017; **9**(7): 15.
139. Glinz D, Wegmüller R, Ouattara M, et al. Iron Fortified Complementary Foods Containing a Mixture of Sodium Iron EDTA with Either Ferrous Fumarate or Ferric Pyrophosphate Reduce Iron Deficiency Anemia in 12- to 36-Month-Old Children in a Malaria Endemic Setting: A Secondary Analysis of a Cluster. *Nutrients* 2017; **9**(7): 759.
140. Godomar Galindo R. Mejoramiento de la olla familiar para alimentar a los niños en edad de lactancia. *Rev chil nutr* 1989; **17**(1,supl): 77-82.
141. Gough EK, Moulton LH, Mutasa K, et al. Effects of improved water, sanitation, and hygiene and improved complementary feeding on environmental enteric dysfunction in children in rural Zimbabwe: A cluster-randomized controlled trial. *PLoS Negl Trop Dis* 2020; **14**(2): e0007963.
142. Grantham-McGregor S, Powell C, Walker S. Nutritional supplements, stunting, and child development. *Lancet* 1989; **2**(8666): 809-10.
143. Granthammcgregor SM, Walker SP, Himes JH, Powell CA. THE EFFECT OF NUTRITIONAL SUPPLEMENTATION AND STUNTING ON MORBIDITY IN YOUNG-CHILDREN - THE JAMAICAN STUDY. *Trans Roy Soc Trop Med Hyg* 1993; **87**(1): 109-13.
144. Gunaratna NS, Bosha T, Belayneh D, Fekadu T, De Groote H. Women's and children's acceptance of biofortified quality protein maize for complementary feeding in rural Ethiopia. *J Sci Food Agric* 2016; **96**(10): 3439-45.
145. Gutierrez MR, Bettiol H, Barbieri MA. Avaliação de um programa de suplementação alimentar. *Rev panam salud pública* 1998; **4**(1): 32-9.
146. Hambidge KM, Chavez MN, Brown RM, Walravens PA. Zinc nutritional status of young middle-income children and effects of consuming zinc-fortified breakfast cereals. *Am J Clin Nutr* 1979; **32**(12): 2532-9.
147. Hambidge KM, Sheng X, Westcott J, et al. Growth of poor rural Chinese children fed meat as a daily complementary food from 6-18 months of age. *FASEB journal* 2013; **27**.
148. Harrington M, Hotz C, Zeder C, et al. A comparison of the bioavailability of ferrous fumarate and ferrous sulfate in non-anemic Mexican women and children consuming a sweetened maize and milk drink. *Eur J Clin Nutr* 2011; **65**(1): 20-5.
149. Haschke F, Pietschnig B, Vanura H, et al. Iron intake and iron nutritional status of infants fed iron-fortified beikost with meat. *Am J Clin Nutr* 1988; **47**(1): 108-12.
150. Haydom Lutheran H, University of T, Tanzania F, Drugs A. Quadruple Fortified Salt Clinical Trial. 2020.
151. Helen Keller I, University of B, University of California D, Unicef, United Nations World Food P. Management of Children With Moderate Acute Malnutrition in Mali. 2011.
152. Hero Institute for Infant N, Universidad de M, Hospital Universitario Virgen de la A, Quantum E. Acceptability, Tolerance, Satiety and Prebiotic Effect of a New Infant Cereal. 2017.
153. Herter-Aeberli I, Eliancy K, Rathon Y, Loechl CU, Marhone Pierre J, Zimmermann MB. In Haitian women and preschool children, iron absorption from wheat flour-based meals fortified with sodium iron EDTA is higher than that from meals fortified with ferrous fumarate, and is not affected by Helicobacter pylori infection in children. *Br J Nutr* 2017; **118**(4): 273-9.
154. Herter-Aeberli I EKRYLCMJZMB. In Haitian women and children, iron absorption from wheat flour fortified with NaFeEDTA is higher than from flour fortified with ferrous fumarate and is not affected by H. pylori infection. *Annals of nutrition & metabolism* 2017; **71**: 1075.
155. Hertrampf E, Olivares M, Pizarro F, et al. HEMOGLOBIN FORTIFIED CEREAL - A SOURCE OF AVAILABLE IRON TO BREAST-FED INFANTS. *Eur J Clin Nutr* 1990; **44**(11): 793-8.
156. Hertrampf E, Olivares M, Pizarro F, et al. Haemoglobin fortified cereal: a source of available iron to breast-fed infants. *Eur J Clin Nutr* 1990; **44**(11): 793-8.
157. Hess SY, Peerson JM, Becquey E, et al. Differing growth responses to nutritional supplements in neighboring health districts of Burkina Faso are likely due to benefits of small-quantity lipid-based nutrient supplements (LNS). *PLoS ONE* 2017; **12**(8): e0181770.
158. Hi PPG, Co. Vertrieb KG, Biofortis Mérieux N. The Combiotic-Study. 2020.
159. Hilmers DC, Mushi A, Griffin IJ, Allen LH, Hicks PD, Abrams SA. A trial of a multi-nutrient fortified beverage in Botswana children. *Pediatr Res* 2002; **51**(4): 210A-A.

160. Hlaing LM, Fahmida U, Htet MK, Firmansyah A, Utomo B, Gibson RS. Iron Supplementation with or without Optimized Complementary Feeding Recommendations: Effect on Micronutrient Status and Growth of 1-2 Year Old Myanmar Children. 2015.
161. Hoffman DR, Theuer RC, Castaneda YS, et al. Maturation of visual acuity is accelerated in breast-fed term infants fed baby food containing DHA-enriched egg yolk. *J Nutr* 2004; **134**(9): 2307-13.
162. Hoffman DR, Garfield S, Morale SE, et al. Visual and neural development of breast-fed infants receiving docosahexaenoic acid (DHA)-enriched baby food: A randomized clinical trial. *Invest Ophthalmol Vis Sci* 2004; **45**: U158-U.
163. Hospital Clinico Universitario de S, Centre for the Development of Industrial T. Effect of a Kefir Beverage on Immunity and Lipid Profile. 2017.
164. Hossain MI, Wahed MA, Ahmed S. Increased food intake after the addition of amylase-rich flour to supplementary food for malnourished children in rural communities of Bangladesh. *Food Nutr Bull* 2005; **26**(4): 323-9.
165. Huey SL, Venkatramanan S, Udipi SA, et al. Acceptability of Iron- and Zinc-Biofortified Pearl Millet (ICTP-8203)-Based Complementary Foods among Children in an Urban Slum of Mumbai, India. *Front Nutr* 2017; **4**: 10.
166. Huo J, Sun J, Chen J. Iron-fortified soy sauce in China-an assessment of 10 years of policy and business development. *Annals of Nutrition and Metabolism* 2013; **63**: 91.
167. Hussain T, Abbas S, Khan MA, Scrimshaw NS. Lysine fortification of wheat flour improves selected indices of the nutritional status of predominantly cereal-eating families in Pakistan. *Food Nutr Bull* 2004; **25**(2): 114-22.
168. Huybregts L, Houngbe F, Salpeteur C, et al. The effect of adding ready-to-use supplementary food to a general food distribution on child nutritional status and morbidity: a cluster-randomized controlled trial. *PLoS Med* 2012; **9**(9): e1001313.
169. Isanaka S, Nombella N, Djibo A, et al. EFFECT OF READY-TO-USE-THERAPEUTIC FOOD SUPPLEMENTATION ON THE NUTRITIONAL STATUS, MORTALITY AND MORBIDITY OF CHILDREN 6 TO 60 MONTHS IN NIGER: A CLUSTER RANDOMIZED TRIAL. *Am J Trop Med Hyg* 2008; **79**(6): 359-.
170. Isanaka S, Barnhart DA, McDonald CM, et al. Cost-effectiveness of community-based screening and treatment of moderate acute malnutrition in Mali. *BMJ Glob Health* 2019; **4**(2): 10.
171. Isrctn. A comparison of diets to treat moderate childhood malnutrition. *http://wwwwhoint/trialsearch/Trial2aspx?TrialID=ISRCTN47598408* 2008.
172. Isrctn. The WinFood Intervention Study: the effect of improved complementary foods on nutrition and health among infants in Western Kenya. *http://wwwwhoint/trialsearch/Trial2aspx?TrialID=ISRCTN30012997* 2012.
173. Isrctn. Evaluating the effectiveness of a legume-enriched nutritional intervention, in treatment of severe undernutrition in children. *http://wwwwhoint/trialsearch/Trial2aspx?TrialID=ISRCTN10309022* 2018.
174. Iuel-Brockdorf AS, Dræbel TA, Fabiansen C, et al. Acceptability of new formulations of corn-soy blends and lipid-based nutrient supplements in Province du Passoré, Burkina Faso. *Appetite* 2015; **91**: 278-86.
175. Iuel-Brockdorf AS, Draebel TA, Ritz C, et al. Evaluation of the acceptability of improved supplementary foods for the treatment of moderate acute malnutrition in Burkina Faso using a mixed method approach. *Appetite* 2016; **99**: 34-45.
176. Jaeggi T, Kortman GA, Moretti D, et al. Iron fortification adversely affects the gut microbiome, increases pathogen abundance and induces intestinal inflammation in Kenyan infants. *Gut* 2015; **64**(5): 731-42.
177. Jahari AB, Haas J, Husaini MA, Pollitt E. Effects of an energy and micronutrient supplement on skeletal maturation in undernourished children in Indonesia. *European journal of clinical nutrition* 2000; **54 Suppl 2**: S74-9.
178. Jalla S, Westcott J, Steirn M, Miller LV, Bell M, Krebs NF. Zinc absorption and exchangeable zinc pool sizes in breast-fed infants fed meat or cereal as first complementary food. *J Pediatr Gastroenterol Nutr* 2002; **34**(1): 35-41.
179. Javan R, Kooshki A, Afzalaghaee M, Aldaghi M, Yousefi M. Effectiveness of supplementary blended flour based on chickpea and cereals for the treatment of infants with moderate acute malnutrition in Iran: A randomized clinical trial. *Electron Physician* 2017; **9**(12): 6078-86.
180. Javaid N, Haschke F, Pietschnig B, et al. Interactions between infections, malnutrition and iron nutritional status in Pakistani infants. A longitudinal study. *Acta Paediatr Scand Suppl* 1991; **374**: 141-50.
181. Javaid N HFPBSEHCSAGPSIHRSMC. Interactions between infections, malnutrition and ironnutritional status in Pakistani infants. *Acta Paediatrica Scandinavica, Supplement* 1991; **80**(374): 141-50.
182. Jilcott SB, Ickes SB, Ammerman AS, Myhre JA. Iterative design, implementation and evaluation of a supplemental feeding program for underweight children ages 6-59 months in Western Uganda. *Maternal and child health journal* 2010; **14**(2): 299-306.

183. John C, Gopaldas T. Evaluation of the impact on growth of a controlled 6-month feeding trial on children (6-24 months) fed a complementary feed of a high energy-low bulk gruel versus a high energy-high bulk gruel in addition to their habitual home diet. *J Trop Pediatr* 1993; **39**(1): 16-22.
184. Jong-Mee LEE, Hea-Jin P. Effects of Supplementary Diet on Iron Status and Development in Infants. *The Korean Journal of Nutrition* 2005: 226-31.
185. Kaimila Y, Pitman RT, Divala O, et al. Development of Acute Malnutrition Despite Nutritional Supplementation in Malawi. *J Pediatr Gastroenterol Nutr* 2019; **68**(5): 734-7.
186. Kajjura RB, Veldman FJ, Kassier SM. Effect of a novel supplementary porridge on the nutritional status of infants and young children diagnosed with moderate acute malnutrition in Uganda: a cluster randomised control trial. *J Hum Nutr Diet* 2019; **32**(3): 295-302.
187. Kajjura RB, Veldman FJ, Kassier SM. Maternal perceptions and barriers experienced during the management of moderately malnourished children in northern Uganda. *Maternal and Child Nutrition* 2020.
188. Kalavi FN, Muroki NM, Omwega AM, Mwadime RK. Effect of tempe-yellow maize porridge and milk-yellow maize porridge on growth rate, diarrhoea and duration of rehabilitation of malnourished children. *East Afr Med J* 1996; **73**(7): 427-31.
189. Kalhoff H, Mesch CM, Stimming M, et al. Effects of LC-PUFA supply via complementary food on infant development-a food based intervention (RCT) embedded in a total diet concept. *Eur J Clin Nutr* 2020; **74**(5): 682-90.
190. Kampstra NA, Hoan NV, Koenders D, et al. Energy and nutrient intake increased by 47-67% when amylase was added to fortified blended foodsa study among 12-to 35-month-old Burkinabe children. *Matern Child Nutr* 2018; **14**(1): 12.
191. Karakochuk C, van den Briel T, Stephens D, Zlotkin S. Treatment of moderate acute malnutrition with ready-to-use supplementary food results in higher overall recovery rates compared with a corn-soya blend in children in southern Ethiopia: an operations research trial. *Am J Clin Nutr* 2012; **96**(4): 911-6.
192. Kekalih A, Anak Agung Sagung IO, Fahmida U, Ermayani E, Mansyur M. A multicentre randomized controlled trial of food supplement intervention for wasting children in Indonesia-study protocol. *BMC Public Health* 2019; **19**(1): 305.
193. King S PADUJOGT. The impact of high diastase malted barley flour on weight and height of malnourished children in Panama. *Journal of hunger & environmental nutrition* 2007; **1**(4): 23-35.
194. Kodkany BS, Bellad RM, Mahantshetti NS, et al. Biofortification of pearl millet with iron and zinc in a randomized controlled trial increases absorption of these minerals above physiologic requirements in young children. *J Nutr* 2013; **143**(9): 1489-93.
195. Konyole S, Kinyuru J, Owuor B, et al. Acceptability of complementary foods with indigenous animal source foods and corn soy blend plus among young children/mothers in Kenya. *Annals of Nutrition and Metabolism* 2013; **63**: 1729.
196. Konyole S ASKJOBEBWJMKFFHRNOV. Effect on lean mass, linear growth and iron status of improved animal source foods and micronutrients fortified complementary foods among kenyan young children: A randomized controlled trial. *Annals of nutrition & metabolism* 2017; **71**: 496-7.
197. Konyole SO, Omollo SA, Kinyuru JN, et al. Effect of locally produced complementary foods on fat-free mass, linear growth, and iron status among Kenyan infants: A randomized controlled trial. *Matern Child Nutr* 2019; **15**(4): e12836.
198. Krebs NF, Westcott JE, Butler N, Robinson C, Bell M, Hambidge KM. Meat as a first complementary food for breastfed infants: feasibility and impact on zinc intake and status. *J Pediatr Gastroenterol Nutr* 2006; **42**(2): 207-14.
199. Krebs NF, Sherlock L, Robertson CE, et al. Enteric Microbiome (EMB) of breastfed infants (BFI) on complementary feeding (CF) regimens with different iron (Fe) exposure. *FASEB journal* 2012; **26**.
200. Krebs NF, Westcott JE, Culbertson DL, Sian L, Miller LV, Hambidge KM. Comparison of complementary feeding strategies to meet zinc requirements of older breastfed infants. *Am J Clin Nutr* 2012; **96**(1): 30-5.
201. Krebs NF, Sherlock LG, Westcott J, et al. Effects of different complementary feeding regimens on iron status and enteric microbiota in breastfed infants. *J Pediatr* 2013; **163**(2): 416-23.
202. Krebs NF, Hambidge KM, Ikemeri JE, et al. Zinc (Zn) absorption from SprinklesTM is not affected by iron (Fe) in Kenyan infants in malaria endemic area. *FASEB journal* 2013; **27**.
203. Kuusipalo H, Maleta K, Briend A, Manary M, Ashorn P. Growth and change in blood haemoglobin concentration among underweight Malawian infants receiving fortified spreads for 12 weeks: a preliminary trial. *J Pediatr Gastroenterol Nutr* 2006; **43**(4): 525-32.
204. Laboratorios O, Ministerio de Ciencia e Innovación S. Whole Grain, Gluten-containing Cereal and PROBIOtics to Evaluate Digestive TOLERAance and Immuno-inflammatory Response. 2021.
205. Lachat CK, Van Camp JH, Mamiro PS, et al. Processing of complementary food does not increase hair zinc levels and growth of infants in Kilosa district, rural Tanzania. *Br J Nutr* 2006; **95**(1): 174-80.

206. LaGrone LN, Trehan I, Meuli GJ, et al. A novel fortified blended flour, corn-soy blend "plus-plus," is not inferior to lipid-based ready-to-use supplementary foods for the treatment of moderate acute malnutrition in Malawian children. *Am J Clin Nutr* 2012; **95**(1): 212-9.
207. Lakkam M, Wager S, Wise PH, Wein LM. Quantifying and exploiting the age dependence in the effect of supplementary food for child undernutrition. *PLoS ONE* 2014; **9**(6): e99632.
208. Langendorf C, Roederer T, de Pee S, et al. Preventing acute malnutrition among young children in crises: a prospective intervention study in Niger. *PLoS medicine* 2014; **11**(9): e1001714.
209. Langlois BK, Cliffer IR, Nikiema L, et al. Factors that May Influence the Effectiveness of 4 Specialized Nutritious Foods in the Prevention of Stunting and Wasting in Children Aged 6-23 Months in Burkina Faso. *Curr Dev Nutr* 2020; **4**(2): 11.
210. Laylo-Navarro Celestina Raquel B, Limos Elizabeth M, Martinez Elizabeth G. A randomized controlled trial on the efficacy and safety of a modified ready to use therapeutic food among malnourished children. *Acta Medica Philippina* 2011: 29-33.
211. Leroy JL, D KO, Bliznashka L, Ruel M. Tubaramure, a Food-Assisted Maternal and Child Health and Nutrition Program in Burundi, Increased Household Food Security and Energy and Micronutrient Consumption, and Maternal and Child Dietary Diversity: A Cluster-Randomized Controlled Trial. *J Nutr* 2020; **150**(4): 945-57.
212. Leroy JL, Olney DK, Nduwabike N, Ruel MT. Tubaramure, a Food-Assisted Integrated Health and Nutrition Program, Reduces Child Wasting in Burundi: A Cluster-Randomized Controlled Intervention Trial. *J Nutr* 2021; **151**(1): 197-205.
213. Libuda L, Mesch CM, Stimming M, et al. Fatty acid supply with complementary foods and LC-PUFA status in healthy infants: results of a randomised controlled trial. *Eur J Nutr* 2016; **55**(4): 1633-44.
214. Li M, Wu J, Ren T, et al. Effect of NaFeEDTA-fortified soy sauce on zinc absorption in children. *Food Funct* 2015; **6**(3): 788-92.
215. Lin CA, Manary MJ, Maleta K, Briend A, Ashorn P. An energy-dense complementary food is associated with a modest increase in weight gain when compared with a fortified porridge in Malawian children aged 6-18 months. *J Nutr* 2008; **138**(3): 593-8.
216. Lind T, Lonnerdal B, Persson LA, Stenlund H, Tennefors C, Hernell O. Effects of weaning cereals with different phytate contents on hemoglobin, iron stores, and serum zinc: a randomized intervention in infants from 6 to 12 mo of age. *Am J Clin Nutr* 2003; **78**(1): 168-75.
217. Lind T, Hernell O, Lonnerdal B, Stenlund H, Domellof M, Persson LA. Dietary iron intake is positively associated with hemoglobin concentration during infancy but not during the second year of life. *J Nutr* 2004; **134**(5): 1064-70.
218. Lind T, Johansson U, Ohlund I, et al. Study protocol: optimized complementary feeding study (OTIS): a randomized controlled trial of the impact of a protein-reduced complementary diet based on Nordic foods. *BMC Public Health* 2019; **19**(1): 134.
219. Litkowski Pe SHCTIMMJ. The addition of whey permeate to ready-to use supplementary food improves recovery from moderate acute malnutrition. *Annals of global health* 2016; **82**(3): 393.
220. Lo NB, Aaron GJ, Hess SY, et al. Plasma zinc concentration responds to short-term zinc supplementation, but not zinc fortification, in young children in Senegal1,2. *Am J Clin Nutr* 2011; **93**(6): 1348-55.
221. Long SE, Murphy KE, Davis WC, Wood LJ. Methods and reference materials for measuring iodine status. *Planta Medica* 2012; **78**(5).
222. Lopez de Romana D, Salazar M, Hambidge KM, et al. Longitudinal measurements of zinc absorption in Peruvian children consuming wheat products fortified with iron only or iron and 1 of 2 amounts of zinc. *Am J Clin Nutr* 2005; **81**(3): 637-47.
223. Ly CT, Diallo A, Simondon F, Simondon KB. Early short-term infant food supplementation, maternal weight loss and duration of breast-feeding: a randomised controlled trial in rural Senegal. *Eur J Clin Nutr* 2006; **60**(2): 265-71.
224. Macharia-Mutie CW, Moretti D, Van den Briel N, et al. Maize porridge enriched with a micronutrient powder containing low-dose iron as NaFeEDTA but not amaranth grain flour reduces anemia and iron deficiency in Kenyan preschool children. *J Nutr* 2012; **142**(9): 1756-63.
225. Macharia-Mutie CW, Omusundi AM, Mwai JM, Mwangi AM, Brouwer ID. Simulation of the effect of maize porridge fortified with grain amaranth or micronutrient powder containing NaFeEDTA on iron intake and status in Kenyan children. *Public Health Nutr* 2013; **16**(9): 1605-13.
226. Macharia-Mutie C, Mwangi A, Brouwer I. Efficacy of Amaranth Grain Flour or Multi-micronutrient Fortified Maize Porridge on Iron of Kenyan Pre-school Children: A Randomized, Controlled Intervention. 2015.
227. Manno D, Siame J, Larke N, Baisley K, Kasonka L, Filteau S. Effect of multiple micronutrient-fortified food on mild morbidity and clinical symptoms in Zambian infants: results from a randomised controlled trial. *Eur J Clin Nutr* 2011; **65**(10): 1163-6.

228. Mahalanabis D, Faruque AS, Wahed MA. Energy dense porridge liquified by amylase of germinated wheat: use in infants with diarrhoea. *Acta Paediatr* 1993; **82**(6-7): 603-4.
229. Mallard SR, Houghton LA, Filteau S, et al. Dietary diversity at 6 months of age is associated with subsequent growth and mediates the effect of maternal education on infant growth in urban Zambia. *J Nutr* 2014; **144**(11): 1818-25.
230. Mamiro PS, Kolsteren PW, van Camp JH, Roberfroid DA, Tatala S, Opsomer AS. Processed complementary food does not improve growth or hemoglobin status of rural tanzanian infants from 6-12 months of age in Kilosa district, Tanzania. *J Nutr* 2004; **134**(5): 1084-90.
231. Manary MJ, Ndkeha MJ, Ashorn P, Maleta K, Briend A. Home based therapy for severe malnutrition with ready-to-use food. *Arch Dis Child* 2004; **89**(6): 557-61.
232. Mank T, Veenemans J, Olomi R, Wielders J, Savelkoul H, Verhoef H. Protection against diarrhoea associated with asymptomatic giardiasis is lost with multi-nutrient supplementation: A prospective study among rural Tanzanian children. *Tropical Medicine and International Health* 2011; **16**: 256.
233. Manno D, Kowa PK, Bwalya HK, et al. Rich micronutrient fortification of locally produced infant food does not improve mental and motor development of Zambian infants: a randomised controlled trial. *Br J Nutr* 2012; **107**(4): 556-66.
234. Marron B, Green J, Jayson L, et al. Comparison of four different supplementary foods in the treatment of moderate acute malnutrition (MAM) in children under five in sierra leone. *FASEB journal* 2015; **29**(1 Meeting Abstracts).
235. Martínez Martínez ÓA. Complementos nutricionales y capitalhumano: un análisis desde los beneficiarios al nutrisano y nutrivida del programa oportunidades de México. *Rev gerenc políticas salud* 2009; **8**(17): 140-54.
236. Martorell R. Complementary Food Supplementation Helps Build Fat-Free Mass, a Little Anyway. *Journal of Nutrition* 2020; **150**(7): 1676-7.
237. Masuda K, Chitundu M. Multiple micronutrient supplementation using spirulina platensis and infant growth, morbidity, and motor development: Evidence from a randomized trial in Zambia. *PLoS ONE* 2019; **14**(2): e0211693.
238. Masuda K, Chitundu M. Multiple Micronutrient Supplementation Using Spirulina platensis during the First 1000 Days is Positively Associated with Development in Children under Five Years: A Follow up of A Randomized Trial in Zambia. *Nutrients* 2019; **11**(4): 29.
239. Matilsky DK, Maleta K, Castleman T, Manary MJ. Supplementary feeding with fortified spreads results in higher recovery rates than with a corn/soy blend in moderately wasted children. *J Nutr* 2009; **139**(4): 773-8.
240. Maust A, Koroma AS, Abla C, et al. Severe and Moderate Acute Malnutrition Can Be Successfully Managed with an Integrated Protocol in Sierra Leone. *J Nutr* 2015; **145**(11): 2604-9.
241. McDonald CM, Ackatia-Armah RS, Doumbia S, Kupka R, Duggan CP, Brown KH. Percent Fat Mass Increases with Recovery, But Does Not Vary According to Dietary Therapy in Young Malian Children Treated for Moderate Acute Malnutrition. *J Nutr* 2019; **149**(6): 1089-96.
242. McGill U, Micronutrient I, Ethiopian H, Nutrition Research I. The Effects of Iodized Salt on Cognitive Development in Ethiopia. 2014.
243. McGill U. Fortified Cheese and Yogurt Products and Vitamin D Status in Young Children? Phase 2. 2015.
244. Medecins Sans Frontieres S. Effectiveness of Nutritional Supplementation in Preventing Malnutrition in Children With Infection in Karamoja, Uganda. 2012.
245. Meeks Gardner J, Grantham-McGregor SM, Chang SM, Himes JH, Powell CA. Activity and behavioral development in stunted and nonstunted children and response to nutritional supplementation. *Child Dev* 1995; **66**(6): 1785-97.
246. Menon P, Ruel MT, Loechl CU, et al. Micronutrient Sprinkles reduce anemia among 9- to 24-mo-old children when delivered through an integrated health and nutrition program in rural Haiti. *J Nutr* 2007; **137**(4): 1023-30.
247. Miles CW, Bodwell CE, Morris E, et al. Long-term consumption of beef extended with soy protein by men, women and children: I. Study design, nutrient intakes, and serum zinc levels. *Plant Foods Hum Nutr* 1987; **37**(4): 341-59.
248. Mize CE, Uauy R, Kramer R, Benser M, Allen S, Grundy SM. Lipoprotein-cholesterol responses in healthy infants fed defined diets from ages 1 to 12 months: comparison of diets predominant in oleic acid versus linoleic acid, with parallel observations in infants fed a human milk-based diet. *J Lipid Res* 1995; **36**(6): 1178-87.
249. Moore N, Chao C, Yang L, Storm H, Oliva-Hemker M, Saavedra JM. Effects of fructo-oligosaccharide-supplemented infant cereal: a double-blind, randomized trial. *British Journal of Nutrition* 2003; **90**(3): 581-7.

250. Morales J, Vargas F, Cassis L, Sanchez E, Villalpando S. Sensorial evaluation of nutritional supplements (PROGRESA) enriched with 3 different forms of iron in a rural Mexican community. *J Food Sci* 2008; **73**(1): S1-5.
251. Moursi M, Mbemba M, Trèche S, Mbemba F. Does the consumption of amylase-containing gruels impact on the energy intake and growth of Congolese infants? *Public Health Nutrition* 2003; **6**(3): 249-57.
252. Nane D, Hatloy A, Tadesse E, Lindtjorn B. Research protocol local ingredients-based supplementary food as an alternative to corn-soya blends plus for treating moderate acute malnutrition among children aged 6 to 59 months: a randomized controlled non-inferiority trial in Wolaita. *BMC Public Health* 2019; **19**(1): 1689.
253. Wageningen U, University of N, Nestlé F, Nevin Scrimshaw International Nutrition F. Improving Iron Status of Children: Potential of Amaranth. 2011.
254. Wageningen U, Zurich ETH, Maseno U. Assessment of Zinc Intake From Enriched Water and Other Dietary Sources in Kisumu, Kenya. 2014.
255. Washington University School of M. Whey Permeate Study. 2015.
256. Washington University School of M, Project Peanut Butter SL, Ministry of H, Sanitation GoSL. Effect of an Alternative RUTF on Intestinal Permeability in Children With Severe Acute Malnutrition. 2021.
257. Washington University School of M. Effectiveness Comparison of Three Supplementary Foods in the Treatment of Moderate Acute Malnutrition. 2011.
258. Washington University School of M, University of California D, World B, United Nations World Food P. Nutributter Programming to Prevent Undernutrition: an Evaluation. 2013.
259. Washington University School of M. Moderate Acute Malnutrition (MAM) and Severe Acute Malnutrition (SAM) in Sierra Leone. 2013.
260. Washington University School of M, University of M. Pilot Study of PUFA-optimized RUTF for Severe Acute Malnutrition. 2014.
261. United States Army Research Institute of Environmental M, United States Department of D. Efficacy of a Once Daily Calcium and Vitamin D Fortified Food Product to Improve Bone Microarchitecture. 2015.
262. Universidad Autonoma de Q, Procter, Gamble. Evaluation of the Efficacy of Different Strategies to Treat Anemia in Mexican Children. 2003.
263. University G, Jimma U, Programme VIUC, Nutrition Tiers M, Nutricia Research F. ω3 LCPUFAs for Healthy Growth and Development of Infants and Young Children in Southwest Ethiopia. 2016.
264. University of O. Effectiveness of Supplementary Feeding During Infection Among Moderately Malnourished Children. 2009.
265. University of C, Axellus. Essential Fatty Acids During Complementary Feeding. 2009.
266. University of O, Program KE-WTCR. A Trial of n-3 PUFA-Enriched Ready to Use Therapeutic Food for Childhood Severe Malnutrition. 2013.
267. University of M. Assessment of Complementary Feeding of Canadian Infants. 2014.
268. University of S, Hawassa U. Using Eggshell Calcium to Mitigate Fluorosis in Ethiopia. 2018.
269. University of F, Michigan State U. Feasibility and Acceptability of a Soy-fiber-maize Complementary Food in Malawi. 2018.
270. University of C, Children's Hospital Medical Center C. Healthy Start to Feeding Intervention. 2020.
271. University of British C, Helen Keller I, Grand Challenges C, International Development Research Centre C, Canadian Institutes of Health R. Thiamin Fortified Fish Sauce as a Means of Combating Infantile Beriberi in Rural Cambodia. 2015.
272. University of California D. Acceptability and Tolerance of a Protein and Micronutrient Fortified Food. 2017.
273. University of California D. Assessment of a New Food Product in Metabolically at Risk Children. 2018.
274. University of Colorado D, International Atomic Energy A, Universidad Francisco M. Zinc Bioavailability From Amaranth-enriched Maize Tortillas. 2015.
275. University of Colorado D, Colorado C, Translational Sciences I, et al. Effect of Protein From Complementary Foods on Infant Growth, Body Composition and Gut Health. 2018.
276. University of Colorado D. The Effects of Early Complementary Feeding on Growth, Neurodevelopment, Sleep and Gut Health. 2022.
277. University of North Carolina CH, General M. The Effect of Yogurt Containing BB12 on Children's Health and Child Care Absenteeism. 2008.
278. University of North Carolina CH, Kinshasa School of Public H, University of California D, Bill, Melinda Gates F, Thrasher Research F. Efficacy of Caterpillar Cereal for Complementary Feeding in the Democratic Republic of Congo. 2012.
279. Swiss Federal Institute of T, Addis Ababa U, HarvestPlus. Iron Status in Infants in Ethiopia. 2019.
280. Technical University of D. The Effect of Vitamin D Fortification of Bread and Milk in Danish Families. 2011.

281. The Hospital for Sick C. Evaluating the Relative Effectiveness of Two Feeding Interventions for the Treatment of Moderate Acute Malnutrition. 2009.
282. The Hospital for Sick C. Treatment of Iron Deficiency Anemia in Malaria Endemic Ghana. 2010.
283. The Hospital for Sick C. Preventing Linear Growth Faltering Among Low Birth Weight Infants in Bangladesh. 2011.
284. Specialties DSMF, United Nations World Food P. Acceptability Among Children and Caregivers of Amylase Porridges. 2014.
285. Swiss Federal Institute of T, Mahidol U. An Efficacy Trial of Iron, Zinc and Vitamin A Fortified Rice in Children in Satun, Thailand. 2010.
286. Swiss Federal Institute of T. Iron Fortification Trail Using NaFeEDTA in Iron Deficient Lead-exposed Children. 2012.
287. Swiss Federal Institute of T, Université dA-C. Iron Absorption From Complementary Food Fortificants (CFFs) and Acceptability of CFFs by Beninese Children. 2012.
288. Swiss Federal Institute of T, University of K, University of N. Changes in Microbiota and Iron Status After Iron Fortification of Complementary Foods. 2012.
289. Swiss Federal Institute of T, Swiss T, Public Health I, Swiss National Science F. Efficacy of Iron Fortified Complementary Food and IPT of Malaria in Young Children in Côte d'Ivoire. 2013.
290. Swiss Federal Institute of T. Iron Absorption From Rice Fortified With Ferric Pyrophosphate. 2014.
291. Swiss Federal Institute of T, Cheikh Anta Diop University S. Fe Absorption in Mother and Child Pairs From Wheat Fortified With Iron With and Without Phenolic Containing Beverages. 2015.
292. Swiss Federal Institute of T, Dsm Nutritional Products I. Iron and Prebiotics Fortification in Kenyan Infants. 2015.
293. Swiss Federal Institute of T, Danone Nutricia R. Iron Absorption From a Wheat-based Instant Cereal:Gut and Stable Isotope Studies in Kenyan Infants. 2020.
294. PepsiCo Global R, Food, Nutrition Research Institute P. Iron Bioavailability of Fortified Oat Drink. 2011.
295. PepsiCo Global R, PepsiCo P. Nutrient Fortified Oat Drink. 2011.
296. Research Institute of Child Nutrition D. Modification in Complementary Food Composition to Improve the Status of Iron and Fatty Acids in Infants. 2007.
297. Aga Khan U, United Nations World Food P, People's Primary Healthcare Initiative S. Comparison of Treatment of SAM in Children 6-59 Months With RUTF and RUSF in Umerkot, Sindh, Pakistan. 2021.
298. Agnes L, Gret, Cellule de Lutte contre la M, Universite Gaston B, International Food Policy Research I. Distribution of Fortified Yoghurt in Senegal to Decrease Children's Anemia and Improve Milk Supply. 2014.
299. Food SRCf, Nutrition, Universitas P, et al. Sustainable Intervention of Supplementation to Improve Kid's Growth Study. 2023.
300. Kansas State U, Project Concern International T, National Institute for Medical Research T, United States Department of Agriculture Foreign Agricultural S. Newly Formulated, Extruded Fortified-blended Foods for Food Aid: the MFFAPP Tanzania Efficacy Study. 2016.
301. Institut de Recherche pour le D, Faso IDB, Hopital Yalgado Ouedraogo Burkina F. Regular Consumption of Leafy Vegetable Sauces and Micro-nutrient Status of Young Children in Burkina Faso. 2013.
302. Laboratorios O. Effect of a New Infant Cereal on Weight Gain. 2012.
303. Swiss Federal Institute of T. The Effect of Lactoferrin and Dosing Regimen on Iron Absorption From a Maize-based Porridge in Kenyan Infants. 2019.
304. Institut de Recherche pour le Developpement C, Unicef, Department of Fisheries Post-harvest T, Quality C. Acceptability of a Multiple Micronutrient-Fortified Lipid-Based Nutrient Supplement for Children Under Two in Cambodia. 2015.
305. Institut de Recherche pour le Developpement C, Unicef, Department of Fisheries Post-harvest T, Quality C. Efficacy of a Multiple Micronutrient-Fortified Lipid-Based Nutrient Supplement for Children Under Two in Cambodia. 2016.
306. Indonesia U, Nestlé F. Food-Based Intervention and Psychosocial Stimulation to Improve Growth & Development of < 24 Month Indonesian Children. 2011.
307. Institut P, Action Contre la F, Centre de Recherches Médicales et Sanitaires CN, Institut Pasteur de M, Institut Pasteur de B, Institut Pasteur de D. Comparing Several Strategies to Manage Moderate Acute Malnutrition Among Children From 6 to 24 Months Old. 2018.
308. Institute of Tropical Medicine B, Institut de Recherche en Sciences de la Sante BF, Ministry of Health BF, University G. Treating Moderate Malnutrition in 6-24 Months Old Children. 2011.
309. Inter-American Development B, Hospital Infantil de Mexico Federico G, Servicios de Salud de N, The PepsiCo F. SPOON:Sustained Program for Improving Nutrition - Mexico. 2020.
310. Inter-American Development B, The PepsiCo F, Program J-JPR, Fundazúcar. SPOON: Sustained Program for Improving Nutrition - Guatemala. 2021.

311. International Centre for Diarrhoeal Disease Research B, Nutrition Third World B, Nestlé F. Ferrous Fumarate and Ferric Pyrophosphate as Food Fortificants in Developing Countries. 2008.
312. International Centre for Diarrhoeal Disease Research B. Zinc Absorption From Zinc Biofortified Rice in Bangladeshi Children. 2020.
313. International Centre for Diarrhoeal Disease Research B. Development and Acceptability Testing of Ready-to-use-complementary Food Supplement (RUCFS) for Children in Bangladesh. 2012.
314. International Centre for Diarrhoeal Disease Research B. Soy-Ready to Use Therapeutic Food (RUTF) in Severely Malnourished Children. 2016.
315. International Centre for Diarrhoeal Disease Research B, Centers for Disease C, Prevention. Vitamin-D Supplementation: Impact on Severe Pneumonia Among Under-five Children. 2017.
316. International Centre for Diarrhoeal Disease Research B, HarvestPlus, International Atomic Energy A. Zinc Absorption From Zinc Biofortified Rice. 2011.
317. International Centre for Diarrhoeal Disease Research B, University of California D. Vitamin B12 Supplementation to Improve B12 Status and Child Development. 2020.
318. International Centre for Diarrhoeal Disease Research B, Washington University School of M. Community-based Clinical Trial With Microbiota-directed Complementary Foods (MDCFs) Made of Locally Available Food Ingredients for the Management of Children With Primary Moderate Acute Malnutrition. 2021.
319. International Centre for Diarrhoeal Disease Research B, Washington University School of M. 'Pre-Proof of Concept (Pre-POC)' Clinical Trials to Optimize Lead Microbiota-directed Complementary Food (MDCF) Prototypes for Their Ability to Repair Microbiota Immaturity and Establish Their Organoleptic Acceptability. 2021.
320. Johns Hopkins Bloomberg School of Public H, International Centre for Diarrhoeal Disease Research B, United Nations World Food P, Ltd DSM. Complementary Food Supplements for Reducing Childhood Undernutrition. 2014.
321. Kintampo Health Research Centre G, University of Wisconsin M. Effects of Fortified Complementary Foods on Vitamin A Status and Body Pool Size in Ghanaian Infants. 2010.
322. London School of H, Tropical M, Maseno University MK. Safe and Efficacious Iron for Children in Kenya. 2014.
323. London School of H, Tropical M, Fhi, Centre M, Africsanté. Alive & Thrive Evaluation in Burkina Faso. 2017.
324. Milton SHMC. A Trial to Evaluate the Safety and Tolerability of a Novel Medical Food for Management of Iron Deficiency Anemia. 2015.
325. Tampere U, Academy of F, Foundation for Paediatric Research F. Trial to Alleviate Malnutrition With Fortified Spread Given as a Food Supplement to Underweight Infants.
326. Mexican National Institute of Public H, Unicef. mHealth for Prevention of Childhood Obesity in Mexico. 2020.
327. University of California D, Helen Keller I, Cheikh Anta Diop University S, Global Alliance for Improved N. Impact of Targeted Zinc Fortification Programs on Plasma Zinc Concentration. 2009.
328. University of Colorado D, Jawaharlal Nehru Medical C, HarvestPlus, International Atomic Energy A. Zinc and Iron Bioavailability From Biofortified Pearl Millet. 2012.
329. National Institutes of H, Eunice Kennedy Shriver National Institute of Child H, Human D. Iron and the Breast-Fed Infant: Iron Status and Two Regimens of Iron Supplementation. 2005.
330. National Institutes of H, Eunice Kennedy Shriver National Institute of Child H, Human D. Cereals as a Source of Iron for Breastfed Infants. 2006.
331. Nct. Randomized Placebo-Controlled Trial (RPCT) of Maize/Zinc in Guatemala. *https://clinicaltrialsgov/show/NCT00098202* 2004.
332. Nct. Effectiveness of Nutritional Products to Treat Moderate Acute Malnutrition. *https://clinicaltrialsgov/show/NCT01898871* 2013.
333. Nct. Comparison of a Two Ready-to-use Supplementary Foods of Differing Protein Quality for the Treatment of MAM. *https://clinicaltrialsgov/show/NCT03549156* 2018.
334. Nestel P, Nalubola R, Sivakaneshan R, Wickramasinghe AR, Atukorala S, Wickramanayake T. The use of iron-fortified wheat flour to reduce anemia among the estate population in Sri Lanka. *Int J Vitam Nutr Res* 2004; **74**(1): 35-51.
335. Nestlé, Swiss Federal Institute of T, University of Malawi College of M. Baby Iron Bioavailability Study. 2019.
336. Nestlé. Effect of Nutritional Intervention on Metabolic Response in Infants. 2021.
337. Nestlé. Nutritional Supplementation in Children Aged 1-3 Years Experiencing Growth Concerns. 2021.
338. Nestlé. Infant Nutrition and Brain Development. 2022.

339. Neufeld LM, Garcia-Guerra A, Quezada AD, et al. A Fortified Food Can Be Replaced by Micronutrient Supplements for Distribution in a Mexican Social Protection Program Based on Results of a Cluster-Randomized Trial and Costing Analysis. *J Nutr* 2019; **149**(Suppl 1): 2302S-9S.
340. Nevin Scrimshaw International Nutrition F, University of G, University of Cape C, Ajinomoto Usa INC, Ghana Health S. Effect of a Complementary Food Supplement on Growth and Morbidity of Ghanaian Infants. 2015.
341. Nicklas TA, O'Neil CE, Fulgoni VL. Nutrient intake, introduction of baby cereals and other complementary foods in the diets of infants and toddlers from birth to 23 months of age. *AIMS Public Health* 2020; **7**(1): 123-47.
342. Nikiema L, Huybregts L, Kolsteren P, et al. Treating moderate acute malnutrition in first-line health services: an effectiveness cluster-randomized trial in Burkina Faso. *Am J Clin Nutr* 2014; **100**(1): 241-9.
343. Nikiema V, Kangas ST, Salpeteur C, et al. Adequacy of Nutrient Intakes of Severely and Acutely Malnourished Children Treated with Different Doses of Ready-To-Use Therapeutic Food in Burkina Faso. *J Nutr* 2021; **11**: 11.
344. North-West University SA, Global Alliance for Improved N, Ltd DSM, Unilever R. Randomized Controlled Trial in South Africa Comparing the Efficacy of Complementary Food Products on Child Growth. 2016.
345. Nozari A, Motamedifar M, Seifi N, Hatamizargaran Z, Ranjbar MA. The Effect of Iranian Customary Used Probiotic Yogurt on the Children's Salivary Cariogenic Microflora. *J Dent (Shiraz)* 2015; **16**(2): 81-6.
346. Nurhasan M, Roos N, Skau JK, et al. Effect of complementary food with small amounts of freshwater fish on whole blood n-3 fatty acids in Cambodian infants age 6-15 months. *Prostaglandins Leukot Essent Fatty Acids* 2018; **135**: 92-101.
347. Obatolu VA. Growth pattern of infants fed with a mixture of extruded malted maize and cowpea. *Nutrition* 2003; **19**(2): 174-8.
348. Oelofse A, Van Raaij JMA, Benade AJS, Dhansay MA, Tolboom JJM, Hautvast J. The effect of a micronutrient-fortified complementary food on micronutrient status, growth and development of 6-to 12-month-old disadvantaged urban South African infants. *Int J Food Sci Nutr* 2003; **54**(5): 399-407.
349. Ordiz MI, Janssen S, Humphrey G, et al. The effect of legume supplementation on the gut microbiota in rural Malawian infants aged 6 to 12 months. *Am J Clin Nutr* 2020; **111**(4): 884-92.
350. Orsango AZ, Loha E, Lindtjorn B, Engebretsen IMS. The efficacy of processed amaranth on anemia prevalence and hemoglobin level in preschool children in South Ethiopia: A randomised controlled trial. *Transactions of the Royal Society of Tropical Medicine and Hygiene* 2019; **113**: S213-S4.
351. Osendarp SJ, Santosham M, Black RE, Wahed MA, van Raaij JM, Fuchs GJ. Effect of zinc supplementation between 1 and 6 mo of life on growth and morbidity of Bangladeshi infants in urban slums. *The American journal of clinical nutrition* 2002; **76**(6): 1401-8.
352. Ouedraogo HZ, Traore T, Zeba AN, Dramaix-Wilmet M, Hennart P, Donnen P. Effect of an improved local ingredient-based complementary food fortified or not with iron and selected multiple micronutrients on Hb concentration. *Public Health Nutr* 2010; **13**(11): 1923-30.
353. Owino VO, Kasonka LM, Sinkala MM, et al. Fortified complementary foods with or without alpha-amylase treatment increase hemoglobin but do not reduce breast milk intake of 9-mo-old Zambian infants. *Am J Clin Nutr* 2007; **86**(4): 1094-103.
354. Owino VO, Bahwere P, Bisimwa G, Mwangi CM, Collins S. Breast-milk intake of 9-10-mo-old rural infants given a ready-to-use complementary food in South Kivu, Democratic Republic of Congo. *Am J Clin Nutr* 2011; **93**(6): 1300-4.
355. Owino V, Kasonka L, Wells J, et al. Breast milk intake of 9-month old zambian infants given fortified complementary foods. *Annals of nutrition & metabolism* 2013; **63**: 554.
356. Owino VO, Skau J, Omollo S, et al. WinFood data from Kenya and Cambodia: constraints on field procedures. *Food Nutr Bull* 2015; **36**(1 Suppl): S41-6.
357. Pactr. DEVELOPMENT AND UTILIZATION OF AMARANTH-SORGHUM GRAINS PRODUCT TO ENHANCE THE NUTRITIONAL STATUS OF MODERATELY MALNOURISHED CHILDREN IN THIKA DISTRICT. *http://wwwwhoint/trialsearch/Trial2aspx?TrialID=PACTR201604001584278* 2016.
358. Pactr. Moderate acute malnutrition treatment. *http://wwwwhoint/trialsearch/Trial2aspx?TrialID=PACTR201809662822990* 2018.
359. Pactr. Efficacy of a strategy combining the promotion of responsive feeding with the daily consumption of a fortified cereal-based blend to reduce anemia, micronutrient deficiencies and stunting in infants from Amparafaravola District, Madagascar. *http://wwwwhoint/trialsearch/Trial2aspx?TrialID=PACTR201906819960554* 2019.
360. Palmer AC, Craft NE, Schulze KJ, et al. Impact of biofortified maize consumption on serum carotenoid concentrations in Zambian children. *Eur J Clin Nutr* 2018; **72**(2): 301-3.

361. Patel MP, Sandige HL, Ndekha MJ, Briend A, Ashorn P, Manary MJ. Supplemental feeding with ready-to-use therapeutic food in Malawian children at risk of malnutrition. *J Health Popul Nutr* 2005; **23**(4): 351-7.
362. Paul KH, Dickin KL, Ali NS, Stoltzfus RJ. Acceptability by mothers and children of two fortified, processed complementary foods for use in an upcoming efficacy trial in Tanzania. *Faseb J* 2007; **21**(5): A677-A.
363. Phu PV, Hoan NV, Salvignol B, et al. Complementary foods fortified with micronutrients prevent iron deficiency and anemia in Vietnamese infants. *J Nutr* 2010; **140**(12): 2241-7.
364. Phu PV, Hoan NV, Salvignol B, et al. A six-month intervention with two different types of micronutrient-fortified complementary foods had distinct short- and long-term effects on linear and ponderal growth of vietnamese infants. *Journal of nutrition* 2012; **142**(9): 1735-40.
365. Phuka JC, Maleta K, Thakwalakwa C, et al. Complementary feeding with fortified spread and incidence of severe stunting in 6- to 18-month-old rural Malawians. *Arch Pediatr Adolesc Med* 2008; **162**(7): 619-26.
366. Phuka JC, Maleta K, Thakwalakwa C, et al. Postintervention growth of Malawian children who received 12-mo dietary complementation with a lipid-based nutrient supplement or maize-soy flour. *Am J Clin Nutr* 2009; **89**(1): 382-90.
367. Phuka J, Thakwalakwa C, Maleta K, et al. Supplementary feeding with fortified spread among moderately underweight 6-18-month-old rural Malawian children. *Matern Child Nutr* 2009; **5**(2): 159-70.
368. Picciano MF, Deering RH. The influence of feeding regimens on iron status during infancy. *Am J Clin Nutr* 1980; **33**(4): 746-53.
369. Pollitt E, Jahari A, Husaini M, Kariger P, Saco-Pollitt C. Developmental trajectories of poorly nourished toddlers that received a micronutrient supplement with and without energy. *J Nutr* 2002; **132**(9): 2617-25.
370. Purwestri RC, Scherbaum V, Inayati DA, et al. Supplementary feeding with locally-produced Ready-to-Use Food (RUF) for mildly wasted children on Nias Island, Indonesia: comparison of daily and weekly program outcomes. *Asia Pac J Clin Nutr* 2012; **21**(3): 374-9.
371. Pynaert I, Armah C, Fairweather-Tait S, Kolsteren P, van Camp J, De Henauw S. Iron solubility compared with in vitro digestion-Caco-2 cell culture method for the assessment of iron bioavailability in a processed and unprocessed complementary food for Tanzanian infants (6-12 months). *Br J Nutr* 2006; **95**(4): 721-6.
372. Qasem W, Azad MB, Hossain Z, et al. Assessment of complementary feeding of Canadian infants: effects on microbiome & oxidative stress, a randomized controlled trial. *BMC Pediatr* 2017; **17**(1): 54.
373. Rahman MM, Islam MA, Mahalanabis D, Biswas E, Majid N, Wahed MA. Intake from an energy-dense porridge liquefied by amylase of germinated wheat: a controlled trial in severely malnourished children during convalescence from diarrhoea. *Eur J Clin Nutr* 1994; **48**(1): 46-53.
374. Rahman MM, Mahalanabis D, Ali M, Mazumder RN, Wahed MA, Fuchs GJ. Absorption of macronutrients and nitrogen balance in children with dysentery fed an amylase-treated energy-dense porridge. *Acta Paediatr* 1997; **86**(12): 1312-6.
375. Rahman MM, Mitra AK, Mahalanabis D, Wahed MA, Khatun M, Majid N. Absorption of nutrients from an energy-dense diet liquefied with amylase from germinated wheat in infants with acute diarrhea. *Journal of pediatric gastroenterology and nutrition* 1997; **24**(2): 119-23.
376. Ramirez MJ, Gonzalez De Cossio T, Rodriguez S, Unar M, Rivera J. Impact evaluation of the nutritional support program (pal by its spanish acronym) on food diversity and consumption of iron-rich or iron-fortified foods in 6-23 month-old children. *Annals of Nutrition and Metabolism* 2013; **63**: 1028.
377. Ramirez-Luzuriaga MJ, Unar-Munguia M, Rodriguez-Ramirez S, Rivera JA, Gonzalez de Cosio T. A Food Transfer Program without a Formal Education Component Modifies Complementary Feeding Practices in Poor Rural Mexican Communities. *J Nutr* 2016; **146**(1): 107-13.
378. Rao DH, Sarma KV, Kumar S, Reddy CG, Roa NP. Acceptability trials with ready to eat foods in a rural area. *Indian Pediatr* 1992.
379. Rim H, Kim S, Sim B, et al. Effect of iron fortification of nursery complementary food on iron status of infants in the DPRKorea. *Asia Pacific journal of clinical nutrition* 2008; **17**(2): 264-9.
380. Rivera JA, Habicht JP, Robson DS. Effect of supplementary feeding on recovery from mild to moderate wasting in preschool children. *Am J Clin Nutr* 1991; **54**(1): 62-8.
381. Rivera JA, Habicht JP. Effect of supplementary feeding on the prevention of mild-to-moderate wasting in conditions of endemic malnutrition in Guatemala. *Bull World Health Organ* 2002; **80**(12): 926-32.
382. Roberts SB, Franceschini MA, Krauss A, et al. A Pilot Randomized Controlled Trial of a New Supplementary Food Designed to Enhance Cognitive Performance during Prevention and Treatment of Malnutrition in Childhood. *Curr* 2017; **1**(11).
383. Roberts SB, Franceschini MA, Silver RE, et al. Effects of food supplementation on cognitive function, cerebral blood flow, and nutritional status in young children at risk of undernutrition: randomized controlled trial. *Bmj* 2020; **370**: m2397.

384. Roediger R, Callaghan-Gillespie M, Blackman JK, Kohlmann K, Maleta KM, Manary M. COMPARISON OF TWO READY-TO-USESUPPLEMENTARY FOODS (RUSF) OF DIFFERING PROTEIN QUALITY FOR THE TREATMENT OF MODERATE ACUTE MALNUTRITION (MAM) IN MALAWI: A RANDOMISED, DOUBLE-BLINDED, CLINICAL EFFECTIVENESS TRIAL. *Gastroenterology* 2020; **158**(6): S-993.
385. Rosado JL, Gonzalez KE, Caamano Mdel C, Garcia OP, Preciado R, Odio M. Efficacy of different strategies to treat anemia in children: a randomized clinical trial. *Nutr J* 2010; **9**: 40.
386. Ruel MT, Rivera JA, Santizo MC, Lonnerdal B, Brown KH. Impact of zinc supplementation on morbidity from diarrhea and respiratory infections among rural Guatemalan children. *Pediatrics* 1997; **99**(6): 808-13.
387. Safaa T Z, Salwa M ES, Fawzi A ES. Role of iron zinc supplementation in the growth retardation and iron deficiency anaemia in Egyptian children. 2003. p. 183-7.
388. Sako B, Leerlooijer JN, Lelisa A, et al. Exploring barriers and enablers for scaling up a community-based grain bank intervention for improved infant and young child feeding in Ethiopia: A qualitative process evaluation. *Matern Child Nutr* 2018; **14**(2): 11.
389. Salinas-Pielago JE, Vega-Dienstmaier JM, Rojas-Oblitas M. [Effect of biscuits fortified with haem iron on the intellectual status of pre-school children]. *Rev Neurol* 1998; **27**(157): 400-4.
390. Samadpour K, Marks GC, Mohammad R, Long K. RANDOMIZED COMPARISON OF TWO TYPES OF MULTIPLE MICRONUTRIENT SUPPLEMENTS (Sprinkles, Foodlets) WITH RECOMMENDED SUPPLEMENT (Drops) IN IRANIAN INFANTS. *Ann Nutr Metab* 2009; **55**: 168-.
391. Sandjaja S, Jus, at I, et al. Fortifying Cooking Oil with Vitamin A in Two Rural Districts of Indonesia: Impact on Vitamin A Status of Mothers and Children. 2015.
392. Sarojini G, Nirmala G, Geetha R. Introduction of red palm oil into the 'ready to eat' used supplementary feeding programme through ICDS. *Indian J Public Health* 1999; **43**(4): 125-31.
393. Sato W FCMHSDOGT-DKGS. A macro and micronutrient fortified complementary food supplement enhances plasma branched-chain amino acid levels in Ghanaian infants. *Annals of nutrition & metabolism* 2017; **71**: 481.
394. Sayyad-Neerkorn J LCRTDSMAAW-MLMMLHSDPSGRF. Preventive effects of long-term supplementation with 2 nutritious food supplements in young children in Niger. *Journal of nutrition* 2015; **145**(11): 2596-603.
395. Sazawal S, Dhingra P, Dhingra U, et al. Compliance with home-based fortification strategies for delivery of iron and zinc: its effect on haematological and growth markers among 6-24 months old children in north India. *J Health Popul Nutr* 2014; **32**(2): 217-26.
396. Scherbaum V, Purwestri RC, Stuetz W, et al. Locally produced cereal/nut/legume-based biscuits versus peanut/milk-based spread for treatment of moderately to mildly wasted children in daily programmes on Nias Island, Indonesia: an issue of acceptance and compliance? *Asia Pac J Clin Nutr* 2015; **24**(1): 152-61.
397. Schlossman N, Batra P, Balan E, et al. The effectiveness of two ready to use supplementary foods (RUSFs) differing in dairy protein content on growth and nutritional status of young children: A pilot study in preschools in guinea-bissau. *FASEB Journal* 2015; **29**(1).
398. Schlossman N, Brown C, Batra P, et al. A Randomized Controlled Trial of Two Ready-to-Use Supplementary Foods Demonstrates Benefit of the Higher Dairy Supplement for Reduced Wasting in Mothers, and Differential Impact in Infants and Children Associated With Maternal Supplement Response. *Food Nutr Bull* 2017; **38**(3): 275-90.
399. Schlossman N. Higher Levels of Dairy Result in Improved Physical Outcomes: A Synthesis of 3 Randomized Controlled Trials in Guinea-Bissau Comparing Supplements with Different Levels of Dairy Ingredients Among Children 6 to 59 Months, 5 to 19 Year Olds, and Mothers in P. *Food Nutr Bull* 2018; **39**(2_suppl): S35-S44.
400. Schroeder DG, Martorell R, Rivera JA, Ruel MT, Habicht JP. Age differences in the impact of nutritional supplementation on growth. *J Nutr* 1995; **125**(4 Suppl): 1051S-9S.
401. Schumann K, Longfils P, Monchy D, von Xylander S, Weinheimer H, Solomons NW. Efficacy and safety of twice-weekly administration of three RDAs of iron and folic acid with and without complement of 14 essential micronutrients at one or two RDAs: a placebo-controlled intervention trial in anemic Cambodian infants 6 to 24 months of ag. *Eur J Clin Nutr* 2009; **63**(3): 355-68.
402. Schwartz J, Dube K, Sichert-Hellert W, et al. Modification of dietary polyunsaturated fatty acids via complementary food enhances n-3 long-chain polyunsaturated fatty acid synthesis in healthy infants: a double blinded randomised controlled trial. *Arch Dis Child* 2009; **94**(11): 876-82.
403. Seal A, Kafwembe E, Kassim IAR, et al. Maize meal fortification is associated with improved vitamin a and iron status in adolescents and reduced childhood anaemia in a food aid-dependent refugee population. *Public health nutrition* 2008; **11**(7): 720-8.

404. Shaikh S, Campbell RK, Mehra S, et al. Supplementation with Fortified Lipid-Based and Blended Complementary Foods has Variable Impact on Body Composition Among Rural Bangladeshi Children: A Cluster-Randomized Controlled Trial. *J Nutr* 2020; **150**(7): 1924-32.
405. Shamah-Levy T, Villalpando S, Rivera-Dommarco JA, Mundo-Rosas V, Cuevas-Nasu L, Jimenez-Aguilar A. Ferrous gluconate and ferrous sulfate added to a complementary food distributed by the Mexican nutrition program Oportunidades have a comparable efficacy to reduce iron deficiency in toddlers. *J Pediatr Gastroenterol Nutr* 2008; **47**(5): 660-6.
406. Shamim A, Hanif AAM, Merrill RD, et al. Preferred Delivery Method and Acceptability of Wheat-Soy Blend (WSB++) as a Daily Complementary Food Supplement in Northwest Bangladesh. *Ecol Food Nutr* 2015; **54**(1): 74-92.
407. Shen Y CISDVSWPRB. Research methods used to determine cost-effectiveness of a supplementary feeding trial to prevent child undernutrition in Burkina Faso. *Annals of nutrition & metabolism* 2017; **71**: 455-6.
408. Shen YK, Griswold S, Suri D, Vosti SA, Rogers B. Costing methods for a cluster-randomized cost-effectiveness trial comparing the performance of four supplementary foods in treating Sierra Leonean children with moderate acute malnutrition (MAM). *FASEB Journal* 2017; **31**(1).
409. Sheng X, Wang J, Li F, Ouyang F, Ma J. Effects of dietary intervention on vitamin B<sub>12</sub> status and cognitive level of 18-month-old toddlers in high-poverty areas: a cluster-randomized controlled trial. *BMC Pediatr* 2019; **19**(1): 334.
410. Shewade HD, Patro BK, Bharti B, Soundappan K, Kaur A, Taneja N. Effectiveness of indigenous ready-to-use therapeutic food in community-based management of uncomplicated severe acute malnutrition: a randomized controlled trial from India. *J Trop Pediatr* 2013; **59**(5): 393-8.
411. Sigh S, Roos N, Sok D, et al. Development and Acceptability of Locally Made Fish-Based, Ready-to-Use Products for the Prevention and Treatment of Malnutrition in Cambodia. *Food Nutr Bull* 2018; **39**(3): 420-34.
412. Simondon KB, Gartner A, Berger J, et al. Effect of early, short-term supplementation on weight and linear growth of 4-7-mo-old infants in developing countries: a four-country randomized trial. *Am J Clin Nutr* 1996; **64**(4): 537-45.
413. Simpore J, Kabore F, Zongo F, et al. Nutrition rehabilitation of undernourished children utilizing Spiruline and Misola. *Nutr J* 2006; **5**: 3.
414. Singh AS, Kang G, Ramachandran A, Sarkar R, Peter P, Bose A. Locally made ready to use therapeutic food for treatment of malnutrition a randomized controlled trial. *Indian Pediatr* 2010; **47**(8): 679-86.
415. Skau JKH, Bunthang T, Chamnan C, et al. Effects of animal-source foods and micronutrientfortification complementary foods on body composition, linear growth, iron status-the WinFood project in Cambodia. *Tropical medicine & international health* 2013; **18**: 87.
416. Skau JKH, Chamnan C, Touch B, et al. Effect of animal-source foods and micronutrient-fortification complementary foods on body composition, linear growth, iron status-the winfood project in cambodia. *Annals of nutrition & metabolism* 2013; **63**: 163-4.
417. Skau JK, Touch B, Chhoun C, et al. Effects of animal source food and micronutrient fortification in complementary food products on body composition, iron status, and linear growth: a randomized trial in Cambodia. *Am J Clin Nutr* 2015; **101**(4): 742-51.
418. Stephenson KB, Agapova SE, Divala O, et al. Complementary feeding with cowpea reduces growth faltering in rural Malawian infants: a blind, randomized controlled clinical trial. *Am J Clin Nutr* 2017; **106**(6): 1500-7.
419. Stobaugh HC, Ryan KN, Kennedy JA, et al. Including whey protein and whey permeate in ready-to-use supplementary food improves recovery rates in children with moderate acute malnutrition: a randomized, double-blind clinical trial. *Am J Clin Nutr* 2016; **103**(3): 926-33.
420. Stobaugh HC, Bollinger LB, Adams SE, et al. Effect of a package of health and nutrition services on sustained recovery in children after moderate acute malnutrition and factors related to sustaining recovery: a cluster-randomized trial. *Am J Clin Nutr* 2017; **106**(2): 657-66.
421. Stookey GK, Carroll RA, Muhler JC. The clinical effectiveness of phosphate-enriched breakfast cereals on the incidence of dental caries in children: results after 2 years. *J Am Dent Assoc* 1967; **74**(4): 752-8.
422. Tampere U, Academy of F, Foundation for Paediatric Research F. Trial to Test the Growth-Promoting Effect of Fortified Spreads When Used as Complementary Food for Infants. 2006.
423. Tampere U. Effectiveness Trial on Alleviation of Infant Malnutrition With Fortified Spread or Maize-Soy Flour Food Supplements. 2008.
424. Tano-Debrah K, Saalia FK, Ghosh S, Hara M. Development and Sensory Shelf-Life Testing of KOKO Plus: A Food Supplement for Improving the Nutritional Profiles of Traditional Complementary Foods. *Food and nutrition bulletin* 2019; **40**(3): 340-56.
425. Tekale N, Kulkarni S. Designing of Nutritional Snacks to Address Micronutrient Deficiency among the Malnourished Tribal Children below Age Five in Western Part of India. 2015.

426. Thakur HS, Gottapu GS, Kadali SP, Kulkarni B, Mamidi RS. Effect of Nutrition Supplementation in Children Living with HIV at ART Centre. *Indian J Pediatr* 2016; **83**(3): 232-7.
427. Thakwalakwa C, Ashorn P, Phuka J, et al. A lipid-based nutrient supplement but not corn-soy blend modestly increases weight gain among 6- to 18-month-old moderately underweight children in rural Malawi. *J Nutr* 2010; **140**(11): 2008-13.
428. Thakwalakwa C, Phiri A, Rollins N, Heikens GT, Barnell EK, Manary M. Growth and HIV-free survival of HIV-exposed infants in Malawi: a randomized trial of two complementary feeding interventions in the context of maternal antiretroviral therapy. *J Acquir Immune Defic Syndr* 2014; **66**(2): 181-7.
429. Tharrey M, Olaya GA, Fewtrell M, Ferguson E. Adaptation of New Colombian Food-based Complementary Feeding Recommendations Using Linear Programming. *J Pediatr Gastroenterol Nutr* 2017; **65**(6): 667-72.
430. The Mathile Institute for the Advancement of Human N, Fundación Salvadoreña para la Salud y el Desarrollo H, Humanitas Global D, Ministry of Health ES. Comparison of Two Nutrition Interventions in Young Children in El Salvador. 2020.
431. Tondeur MC, Schauer CS, Christofides AL, et al. Determination of iron absorption from intrinsically labeled microencapsulated ferrous fumarate (sprinkles) in infants with different iron and hematologic status by using a dual-stable-isotope method. *Am J Clin Nutr* 2004; **80**(5): 1436-44.
432. Traore T, Vieu MC, Alfred TS, Serge T. Effects of the duration of the habituation period on energy intakes from low and high energy density gruels by Burkinabe infants living in free conditions. *Appetite* 2005; **45**(3): 279-86.
433. Traore T, Mouquet-Rivier C, Kabore C, Bruyeron O. Efficacy of different fortified gruels on the recovery from moderate acute malnutrition in burkinabe 6-23-month-old children. *Annals of nutrition & metabolism* 2013; **63**: 1024-5.
434. Trehan I, Banerjee S, Murray E, et al. Extending supplementary feeding for children younger than 5 years with moderate acute malnutrition leads to lower relapse rates. *J Pediatr Gastroenterol Nutr* 2015; **60**(4): 544-9.
435. Tufts U, Washington University School of M, Project Peanut B, United Nations World Food P, United States Agency for International D. Comparison of Four Different Supplementary Foods in the Treatment of Moderate Acute Malnutrition (MAM) in Children in Sierra Leone: a Cluster-randomised, Controlled Clinical Effectiveness Trial. 2014.
436. Tufts U, Massachusetts General H, Global F, Nutrition I. Locally Prepared Supplement to Support Growth and Brain Health. 2017.
437. Tufts U, United States Agency for International D, Acdi/Voca, Save the C, Institut de Recherche en Sciences de la Sante BF. Effectiveness and Cost-Effectiveness of Four Formulations of Food Supplements for the Prevention of Wasting and Stunting in Burkina Faso. 2017.
438. Tufts U, Washington University School of M, Project Peanut B, et al. Comparison of Four Different Supplementary Foods in the Treatment of Moderate Acute Malnutrition. 2018.
439. van der Kam S, Swarthout T, Niragira O, et al. Ready-to-use therapeutic food for catch-up growth in children after an episode of Plasmodium falciparum malaria: an open randomised controlled trial. *PLoS ONE* 2012; **7**(4): e35006.
440. Vander Wal MC. Evaluation of Antihelminth Treatment and an Aloe Enriched Whey Protein Drink on Measures of HIV Enteropathy and Immune Activation in HIV+ Children in Addis Ababa. *Dissertation/ Thesis* 2018: 1-.
441. Van Hoan N, Van Phu P, Salvignol B, Berger J, Treche S. Effect of the consumption of high energy dense and fortified gruels on energy and nutrient intakes of 6-10-month-old Vietnamese infants. *Appetite* 2009; **53**(2): 233-40.
442. Varea A, Malpeli A, Etchegoyen G, et al. Short-term evaluation of the impact of a food program on the micronutrient nutritional status of Argentinean children under the age of six. *Biological trace element research* 2011; **143**(3): 1337-48.
443. Vega NIV, Levy TS, Pineda EBG, Nasu LC, Gomez-Humaran IM. Adherence to the consumption of food supplements of PROSPERA program, in the decrease of the prevalence of anemia in children under three years old in the state of San Luis Potosi, Mexico. *Nutr Hosp* 2016; **33**(4): 782-9.
444. Verkaik-Kloosterman J, Beukers MH, Jansen-van der Vliet M, Ocké MC. Vitamin D intake of Dutch infants from the combination of (fortified) foods, infant formula, and dietary supplements. *European Journal of Nutrition* 2017; **56**(2): 581-90.
445. Verna M, Corradi M, Fantoni S, et al. Parma pap project: A ready to use therapeutic food for moderately malnourished children in sierra leone. *Archives of disease in childhood* 2012; **97**: A301.
446. Villanueva LM, Norton SA, Palacios AM, Reinhart GA. Acceptability of a fortified atole among guatemalan children who attend six casa Del niño day care centers, and the effects on linear growth and hemoglobin status. *FASEB Journal* 2016; **30**.

447. Viseshakul D, Premwatana P, Kewsiri D. FIELD TRIAL OF SUPPLEMENTARY FEEDING IN HILL-TRIBE CHILDREN (1-4 YEARS OLD) - ONE YEAR FOLLOW-UP-STUDY. *Journal of the Medical Association of Thailand* 1979; **62**(4): 190-9.
448. Vray M, Hedible BG, Adam P, et al. A multicenter, randomized controlled comparison of three renutrition strategies for the management of moderate acute malnutrition among children aged from 6 to 24 months (the MALINEA project). *Trials* 2018; **19**(1): 666.
449. Vuong le T, Dueker SR, Murphy SP. Plasma beta-carotene and retinol concentrations of children increase after a 30-d supplementation with the fruit Momordica cochinchinensis (gac). *Am J Clin Nutr* 2002; **75**(5): 872-9.
450. Walker SP, Grantham-McGregor SM, Himes JH, Powell CA, Chang SM. Early childhood supplementation does not benefit the long-term growth of stunted children in Jamaica. *J Nutr* 1996; **126**(12): 3017-24.
451. Wang RJ, Trehan I, LaGrone LN, et al. Investigation of food acceptability and feeding practices for lipid nutrient supplements and blended flours used to treat moderate malnutrition. *J Nutr Educ Behav* 2013; **45**(3): 258-63.
452. Walter TD, P. R.; Pizarro, F.; Velozo, L.; Pena, G.; Bartholmey, S. J.; Hertrampf, E.; Olivares, M.; Letelier, A.; Arredondo, M. Effectiveness of iron-fortified infant cereal in prevention of iron deficiency anemia. *Pediatrics* 1993; **91**(5): 976-82.
453. Whitfield KC, Karakochuk CD, Kroeun H, et al. Consumption of novel thiamin-fortified fish sauce improves the thiamin status of rural cambodian women of childbearing age and their children <5 years. *FASEB Journal Conference: Experimental Biology 2016, EB San Diego, CA United States Conference Start: 20160402 Conference End: 20160406 Conference Publication: (varpagings)* 2016; **30**(no pagination).
454. Whitfield KC, Karakochuk CD, Kroeun H, et al. Household Consumption of Thiamin-Fortified Fish Sauce Increases Erythrocyte Thiamin Concentrations among Rural Cambodian Women and Their Children Younger Than 5 Years of Age: A Randomized Controlled Efficacy Trial. *J Pediatr* 2017; **181**: 242-7.e2.
455. Westcott JE, Culbertson D, Lei S, Hambidge KM, Post K, Krebs NF. Complementary food (CF) choices are critical to meet physiologic requirements for zinc (Zn). *FASEB journal* 2011; **25**.
456. Women's NGNf, Children's H, Kinshasa School of Public H, et al. First Bites: Complementary Feeding - A Global Network Cluster Randomized Controlled Trial. 2010.
457. Yeung GS, Zlotkin SH. Efficacy of meat and iron-fortified commercial cereal to prevent iron depletion in cow milk-fed infants 6 to 12 months of age: a randomized controlled trial. *Can J Public Health* 2000; **91**(4): 263-7.
458. Ying C, Dongsheng LIU, Jiguo BAI, et al. THE RELATIONSHIP BETWEEN THE DIET AND THE GROWTH OF INFANTS DURING THE WEANING PERIOD (6-18 MONTHS). *Acta Nutrimenta Sinica* 1956; (6).
459. Yu D, Ma G, Joop V, Wanda B, Fang Z, Zhao L. The effects on children's growth, hemoglobin concentration, and anemia in 3 nutrition interventions in poor rural, China. *Annals of nutrition & metabolism* 2013; **63**: 611.
460. Yuliarti K, Honoris E, Sjarif D. Chicken liver compared to fortified rice cereal as a first complementary food for breastfed infants: Effectivity on zinc intake and status. *Journal of Pediatric Gastroenterology and Nutrition* 2017; **64**: 809.
461. Zakaria, Rauf S, Salim A. Development of instant powder formula with addition of moringa oleifera as a complementary food for infants aged 6-12 months. *Annals of Nutrition and Metabolism* 2019; **75**(3): 172.
462. Zakaria, Rauf S, Salim A, Rahman N, Bohari. Development of instant powder with the addition of Moringa oleifera leaf powder as complementary food for infants 6-12 months old. *Systematic Reviews in Pharmacy* 2020; **11**(7): 61-4.
463. Zavaleta N, Kvistgaard AS, Graverholt G, et al. Efficacy of an MFGM-enriched complementary food in diarrhea, anemia, and micronutrient status in infants. *J Pediatr Gastroenterol Nutr* 2011; **53**(5): 561-8.
464. Zhang Y, Wu Q, Wang W, et al. Effectiveness of complementary food supplements and dietary counselling on anaemia and stunting in children aged 6-23 months in poor areas of Qinghai Province, China: a controlled interventional study. *BMJ Open* 2016; **6**(10): e011234.
465. Zhichien HE. THE INFLUENCE OF WEANING FOOD ON THE GROWTH OF INFANTS WHILE BREAST-FEEDING. *Acta Nutrimenta Sinica* 1956; (6).
466. Ziegler EE, Nelson SE, Jeter JM. Iron status of breastfed infants is improved equally by medicinal iron and iron-fortified cereal. *Am J Clin Nutr* 2009; **90**(1): 76-87.
467. Ziegler EE, Nelson SE, Jeter JM. Iron supplementation of breastfed infants. *Nutr Rev* 2011; **69**: S71-S7.
468. Ziegler EE, Fomon SJ, Nelson SE, Jeter JM, Theuer RC. Dry cereals fortified with electrolytic iron or ferrous fumarate are equally effective in breast-fed infants. *J Nutr* 2011; **141**(2): 243-8.
469. Zyba SJ, Wegmuller R, Woodhouse LR, et al. Effect of exogenous phytase added to small-quantity lipid-based nutrient supplements (SQ-LNS) on the fractional and total absorption of zinc from a millet-based

porridge consumed with SQ-LNS in young Gambian children: a randomized controlled trial. *Am J Clin Nutr* 2019; **110**(6): 1465-75.

## Supplement 3. Characteristics of studies awaiting classification

a) **registered trials without publication**

| Study identifier | NCT03573570 [1] |
|---|---|
| **Study title:** | Reducing anaemia through food fortification at scale<br>**Acronym: –** |
| **Official title:** | Reducing Anemia Through Food Fortification at Scale |
| **Methods:** | **Type of trial:** interventional<br>**Allocation:** randomised<br>**Intervention model:** parallel assignment<br>**Masking:** none<br>**Primary purpose:** treatment |
| **Participants:** | **Age:** 6 months to 5 years<br>**Enrollment:** 0<br>**Inclusion criteria:** 6 months to 5 years<br>**Exclusion criteria:** none |
| **Interventions** | **Intervention(s):** Rice will be fortified using Fortified Rice Kernels (FRKs) containing iron, zinc, vitamin A and vitamins B1, B3, B6, B9 and B12<br>**Comparator(s):** regular rice |
| **Starting date** | **Trial start date: –**<br>**Trial completion date: –**<br>**Status:** Withdrawn (The Government of Tamil Nadu decided not to proceed with implementation of fortified rice through the Public Distribution (per the original study protocol)) |
| **Contact information** | **Responsible party/principal investigator:** Norman G Miller, Stanford University |
| **Stated purpose of study** | **Quote: "**This trial proposes to address anemia and other micronutrient deficiencies by providing micronutrient fortified rice through the Public Distribution System (PDS) of Tamil Nadu in a manner that requires no change in behaviour by end-user households and that can feasibly be conducted on a large scale **"** |
| **Note** | Recruitment Status: Withdrawn (The Government of Tamil Nadu decided not to proceed with implementation of fortified rice through the Public Distribution (per the original study protocol))<br>First Posted: June 29, 2018<br>Last Update Posted: March 18, 2021 |

| Study identifier | NCT02532816 [2] |
|---|---|
| **Study title:** | Nutrient-dense complementary foods on catch-up growth and nutritional status of stunting children<br>**Acronym: –** |
| **Official title:** | The Effect of Higher Nutrient-Dense Complementary Foods on Catch-up Growth and Nutritional Status of Stunting Children in Dompu District, Indonesia |
| **Methods:** | **Type of trial: interventional**<br>**Allocation: randomised**<br>**Intervention model: not clear**<br>**Masking:** Quadruple (Participant, Care Provider, Investigator, Outcomes Assessor)<br>**Primary purpose: treatment** |
| **Participants:** | **Age: 12-23 months**<br>**Enrollment: actual 217**<br>**Inclusion criteria:**<br>• identified as stunting (having HAZ ≤ -2SD of the WHO Growth Standard 2006)<br>• no clinical evidence of any acute infectious disease or other diseases or morbidity condition that could interfere with the intake of study diets<br>• parents and children residence in the study area.<br>• parental concent obtained<br>**Exclusion criteria:**<br>• presence of oedema, severe illness warranting hospitalization on the enrolment day such as persistent diarrhea and other disease which may influence feeding practices and nutrient absorption<br>• conncurrent participation in another clinical trial<br>• severe anemia with hemoglobine concentration < 7.0 g/dL |
| **Interventions** | **Intervention(s):**<br>• Optimized Complementary Feeding Recommendation (CFR) + fortified biscuit I (ferrous fumarate 83.5 mg, zinc oxide 50.95 mg, Calcium carbonate 3104.05 mg, Thiamine Mononitrate1.85 mg, Nicotinic Acid 30.45 mg, Pyridoxine Hydrocloride 2.90 mg, Pteroyl monoglutamic acid 764.90 mcg, Cyanocobalamin 0.95 mcg, Retinol Palmitate (dry) 742.50 mcgRE)<br>• CFR + fortified biscuit II (ferrous fumarate 32.6 mg, zinc oxide 3.78 mg, Calcium carbonate 874.12 mg, Thiamine Mononitrate1.25 mg, Nicotinic Acid 17.95 mg, Pyridoxine Hydrocloride 1.10 mg, Pteroyl monoglutamic acid 329.90 mcg, Cyanocobalamin 0.55 mcg)<br>**Comparator(s):**<br>• CFR + non-fortified biscuit |
| **Starting date** | **Trial start date:** April 2016<br>**Trial completion date:** July 2016 |
| **Contact information** | **Responsible party/principal investigator:** Duma O Fransisca and Umi Fahmida, SEAMEO Regional Centre for Food and Nutrition |
| **Stated purpose of study** | **Quote: "t**o determine and compare the effect of higher nutrient-dense complementary foods and standard nutrient dense complementary foods on the catch-up growth and nutritional status of stunting children aged 12-23 months old in Indonesia**"** |
| **Note** | Recruitment Status: Unknown<br>Verified August 2015 by Duma Octavia Fransisca, MSc, SEAMEO Regional Centre for Food and Nutrition.<br>Recruitment status was: Active, not recruiting<br>First Posted: August 26, 2015<br>Last Update Posted: August 26, 2015 |

b) **Published protocols**

| Study | Mehta 2017 [3] |
|---|---|
| **Methods** | **Study design:** randomised controlled trial |
| **Participants** | **Inclusion criteria:**<br>• 12–18 months old<br>• Hemoglobin ≥9 g/dL<br>• living in urban slums of Mumbai<br>**Exclusion criteria:**<br>• Age <12 months, 0 days or >18 months<br>• Hemoglobin < 9 g/dL and/or hemoglobinopathy<br>• severe malnutrition (marasmus, marasmic kwashiorkor, kwashiorkor, weight-for-height z-score < -3)<br>• Diagnosis: Prior: HIV/AIDS or Tuberculosis, or Current: HIV/AIDS, malaria, Dengue fever, Tuberculosis >1-day hospitalization<br>• Children without caretaker<br>• no migrating from the slum for 4 weeks<br>• Prior/current consumption: iron/zinc supplements in the past 1 year<br>• no dietary allergies<br>**Age at the start of the intervention:** 12-18 month<br>**Country where trial was performed:** India |
| **Interventions** | **Intervention(s):**<br>• FeZnPM (iron- and zinc-biofortified pearl millet)<br>**Comparator(s):**<br>• CtrlPM (conventional pearl millet)<br>**Duration of intervention:** 9 months<br>**Duration of follow-up:** 9 months<br>**Run-in period:** –<br>**Number of study centres: -** |
| **Outcomes** | **Outcomes listed in the protocol:**<br>Hb, serum ferritin, serum transferrin receptor and plasma zinc, growth, immune function, cognitive function |
| **Identification** | **Trial identifier:** NCT02233764, REF/2014/10/007731, CTRI/2015/11/006376<br>**Trial terminated early:** no |
| **Publication details** | **Language of publication:** English<br>**Funding:** HarvestPlus (2014H8302)<br>**Publication status:** published protocol in peer-reviewed journal |
| **Stated aim for study** | **Quote: "**This study aims to investigate the effect of the consumption of foods prepared with iron- and zinc-biofortified pearl millet (FeZn-PM) by children on biomarkers of iron and zinc status, growth, and immune function" |
| **Note** | |

c) **Conference abstracts**

| Study | Hays 2019 [4 5] |
|---|---|
| **Methods** | **Study design:** cluster-randomized, double-blind, controlled study |
| **Participants** | **Inclusion criteria:**<br>aged 6-18 months<br>**Exclusion criteria: –**<br>**Setting:** La Nkwantanang Municipality of the Greater Accra Region, Ghana<br>**Age at the start of the intervention (age subgroups):** 6-18 months<br>**Country where trial was performed:** Ghana |
| **Interventions** | **Intervention(s):** micronutrient-fortified infant cereal with iron (3.75 mg iron as ferrous fumarate / 50 g cereal<br>**Comparator(s):** the same cereal without iron<br>**Duration of intervention:** 6 months<br>**Duration of follow-up:** 8 months |
| **Outcomes** | **Reported outcomes in the abstract:** Hb, weight, height, mid-upper arm circumference, usual dietary intake |
| **Identification** | **Trial identifier:** PACTR201906885776793<br>**Trial terminated early:** no |
| **Publication details** | **Language of publication:** English<br>**Funding**: Nestlé Company Limited (Ghana) in collaboration with the University of Ghana School of Biological Sciences<br>**Publication status:** conference abstract |
| **Stated aim for study** | **Quote:** "to assess the effect of a micronutrient-fortified complementary food on hemoglobin, anemia prevalence, and growth of infants in the La Nkwantanang Municipality of the Greater Accra Region, Ghana." |
| **Note** | |

| Study | Krebs 2011 [6] |
|---|---|
| Methods | **Study design:** randomized study |
| Participants | **Inclusion criteria:**<br>~ 6 mo of ag<br>**Exclusion criteria: –**<br>**Setting: –**<br>**Age at the start of the intervention (age subgroups):** ~ 6 months<br>**Country where trial was performed: –** |
| Interventions | **Intervention(s):**<br>infant cereal with Zn fortification<br>**Comparator(s):**<br>infant cereal without Zn fortification<br>**Duration of intervention:** –<br>**Duration of follow-up:** 3-4 months |
| Outcomes | **Reported outcomes in the abstract:** Exchangeable zinc (Zn) pool (EZP) size, Diet Zn, daily absorbed Zn, and plasma Zn |
| Identification | **Trial identifier:** –<br>**Trial terminated early:** – |
| Publication details | **Language of publication:** English<br>**Funding**: –<br>**Publication status:** conference abstract |
| Stated aim for study | **Quote:** "Using stable isotope methods, we measured EZP size at 9-10 mo of age in healthy breastfed infants (n=37) who had a wide range of habitual dietary Zn resulting from random assignment at ~ 6 mo of age to 1 of 3 complementary feeding groups: infant cereal, with and without Zn fortification, or meats." |
| Note | |

## Full text not available

- Araya 1994 [7]
- Arya 2000 [8]
- Cros 1966 [9]
- OrganizaciónPanamericanadelaSalud 1989 [10]
- Viseshakul 1979 [11]
- Viteri 1981 [12]
- Zhao 2004 [13]
- Zlotkin 2000 [14]

**References**


1. Stanford U, Government of Tamil N, Global Innovation F, et al. Reducing Anemia Through Food Fortification at Scale, 2020.
2. Food SRCf, Nutrition, Nestlé F, et al. Nutrient-Dense Complementary Foods on Catch-up Growth and Nutritional Status of Stunting Children, 2016.
3. Mehta S, Finkelstein JL, Venkatramanan S, et al. Effect of iron and zinc-biofortified pearl millet consumption on growth and immune competence in children aged 12-18 months in India: study protocol for a randomised controlled trial. *BMJ Open* 2017;7(11):e017631. doi: https://dx.doi.org/10.1136/bmjopen-2017-017631
4. Hays N, Vuvor F, Paul S, et al. Effect of an Iron-Fortified Complementary Food on Anemia and Nutritional Status of Infants Aged 6-18 Months in Ghana. *Nutrition 2019* 2019
5. Pactr. Efficacy of an iron fortified complementary food in improving the nutritional status of children aged 6-18 months in the La Nkwantanang Municipality of the Greater Accra Region. *http://wwwwhoint/trialsearch/Trial2aspx?TrialID=PACTR201906885776793* 2019
6. Krebs NF, Westcott JE, Culbertson D, et al. Exchangeable zinc (Zn) pool (EZP) size, but not plasma Zn, reflects absorbed Zn in breastfed infants on different complementary foods. *FASEB journal* 2011;25
7. Araya L H. Importancia de la fortificación de alimentos en la salud de la población chilena. *Rev chil nutr* 1994;22(3):137-43.
8. Arya V. Calcium and the treatment of nutritional rickets. 2000
9. Cros J, Schartner J. [Trial food supplementation for children of Diack canton (Senegal)]. *Bull Soc Med Afr Noire Lang Fr* 1966;11(2):193-7.
10. Organización Panamericana de la Salud. Programa de Alimentación y N, Chile. Instituto de Nutrición y Tecnología de los A. Estudio sobre intervenciónes alimentario-nutricionales para poblaciones de bajos ingresos en Latinoamerica y el Caribe: Resumen del proyecto colaborativo OPS/INTA, 1989:139-39.
11. Viseshakul D, Premwatana P, Kewsiri D. A field trial of supplementary feeding in hilltribe children (1-4 years old): one year follow-up study. *J Med Assoc Thai* 1979;62(4):190-200.
12. Viteri FE, Alvarez E, Bulux J, et al. Iron fortification in developing countries. *Prog Clin Biol Res* 1981;77:345-54.
13. Zhao X, Lu Q, Wang S, et al. [Efficiency of NaFeEDTA fortified soy sauce on anemia prevention]. *Wei Sheng Yen Chiu* 2004;33(2):202-4.
14. Zlotkin SH, Yeung GS, Colleta F, et al. Fractional zinc absorption from Zn-fortified cereals in infants and young children. *Pediatric research* 2000;47(4):237A.

## Supplement 4. Characteristics of included studies

| **Study (Covidence identifier)** | **Palmer 2021** (#4720) [1 2] |
|---|---|
| **Methods** | **Study design:** parallel randomized control trial<br>**Unit of randomisation:** individual<br>**Blinding:** blinding (colour-coded containers) not mentioned single-blind or double blind<br>**Number of study arms:** 3 arms |
| **Participants** | **Location/Setting**: Mkushi District in the Central Province of Zambia<br>**Country where trial was performed:** Zambia<br>**Sample size:** 255<br>**Dropouts/withdrawals:** Lost to follow up: 18 (refused), 7 (moved), and incomplete biospecimen: 69 infants<br>**Sex**: both male and female<br>**Inclusion criteria:**<br>Infants:<br>• healthy<br>• singleton infants<br>• hemoglobin concentration ≥ 7.0 g/dL<br>• received a vitamin A capsule (105 µmol) at 6mo of age<br><br>Mothers:<br>• age 18-45 years<br>• Hemoglobin concentration ≥8.0 g/dL<br>• free from chronic health conditions (i.e., any issue requiring regular medical visits)<br>• brestfeeding and planning to continue through ≥12 mo postpartum<br>• not currently pregnant<br>• not planning to relocate<br><br>**Exclusion criteria:**<br><br>Infant(s):<br>• not receiving the 105 µmol dose of vitamin A at ∼6 months<br>• or intent to move from the study area<br>Mother(s):<br>• pregnancy<br>• not currently breastfeeding or planning to cease breastfeeding prior to the infant's first birthday<br>Both (Mother/infants pairs):<br>• chronic health condition in the mother or infant<br>• severe anemia in the mother (Hb < 8.0 g/dL) or infant (Hb < 7.0 g/dL)<br>**Health status:** healthy infants<br>**Ongoing treatment:** no treatment<br>**Anaemic status:** mixed (Defined as hemoglobin <12 g/dL for women and <10 g/dL for infants)<br>**Age range at start of intervention:** 9-12 months<br>**Mean age: no data** |
| **Interventions** | **Intervention(s):**<br>• retinyl palmitate–fortified white maize (FM) (n=85); serving size (287 g dry weight/d for women; 50 g dry weight for infants; 2 meals/day, for 6d/wk for 90 d<br>• biofortified orange maize (BM) (n=85); serving size (287 g dry weight/d for women; 50 g dry weight for infants; 2 meals/day, for 6d/wk for 90 d)<br><br>**Comparator(s):**<br>• conventional low-carotenoid white maize (CM) (n=85); serving size (287 g dry weight/d for women; 50 g dry weight for infants; 2 meals/day, for 6d/wk for 90 d)<br><br>– white maizes (for the WM and FM) purchased from the same harvest season<br>**Duration of intervention:** 90-d<br>**Duration of follow-up:** 90 days<br>**Run-in period:** –<br>**Number of study centres:** 1 |
| **Outcomes** | **Reported outcomes in full text of publication:** plasma retinol**,** total body stores (TBS), liver retinol concentration<br>**Primary outcomes:**<br>Total body vitamin A stores of infants measured by retinol isotope dilution<br>**Secondary outcomes:**<br>Breast milk retinol concentrations of women measured by high performance liquid chromatography<br>Plasma retinol concentrations of women measured by high performance liquid chromatography<br>Pupillary responsiveness of women measured by portable field dark adaptometer<br>**Timing of outcome assessment:** baseline and endline |
| **Identification** | **Trial identifier:** NCT02804490<br>**Trial terminated early:** no |
| **Publication details** | **Language of publication:** English |

| | |
|---|---|
| | **Funding:** HarvestPlus (www.HarvestPlus.org). In-kind support was provided by DSM Nutritional Products, Inc. (fortificant) and Bioanalyt GmbH (iCheck Fluoro and consumables). Additional funding was provided by the Sight and Life Global Nutrition Research Institute at Johns Hopkins University, with support from the Christian Blind Mission.<br>**Conflict of interest: „**The authors report no conflicts of interest”<br>**Publication status:** full article in peer-reviewed journal |
| **Stated aim for study** | **Quote: “**To determine whether biofortified or industrially fortified maize consumption by Zambian women and their breastfeeding infants could improve milk retinol concentration and infant TBS.” |
| **Note** | **Study start date:** March 2016<br>**Study end date:** June, 2017 |

| **Study (Covidence identifier)** | **Ekoe 2020** [3 4]<br>(# 5741) |
|---|---|
| **Methods** | **Study design:** cluster randomised trial<br>**Unit of randomisation:** cluster (30 villages)<br>**Blinding:** double-blind<br>**Number of study arms:** 2 arms |
| **Participants** | **Location/Setting**: Salapoumbé<br>**Country where trial was performed:** (East) Cameroon<br>**Sample size:** 205<br>**Dropouts/withdrawals:** 52 (14 moved away, 13 absent, 25 refused blood collection)<br>**Sex:** both male and female children included<br>**Inclusion criteria:**<br>• Apparent good health<br>• 18 to 59 months<br>• Haemoglobin rate ranging 7 to 11 g/dl<br>**Exclusion criteria:**<br>• iron supplementation in progress<br>• Clinical presentation of severe malnutrition (e.g., bilateral pitting oedema<br>• Diagnosis of any chronic infection (tuberculosis, HIV);<br>• Severe acute infection (e.g., severe malaria, pneumonia, meningitis);<br>• Blood transfusion < 3 months prior to enrollment;<br>• Allergy/intolerance to the cow's milk and/or to the gluten<br>**Health status: „**apparent good health”<br>**Ongoing treatment:** no data<br>**Anaemic status:** anaemic („anemic (hemoglobin 7–11 g/dl) but otherwise healthy children”)<br>**Age range at start of intervention:** 18 to 59 months<br>**Mean age:**<br>Age (months) Iron-fortified IC group (N = 106): 32.1 ± 10.9;<br>Age (months) Control IC group (N = 99): 36.1 ± 10.8 |
| **Interventions** | **Intervention(s):**<br>• Iron fortified infant cereal (IC):<br>two 50 g servings/day IC with 7.5 mg of ferrous fumarate<br>providing 3.75 mg iron/serving; (n = 106)<br>**Comparator(s):**<br>• Control IC: infant cereal<br>two 50 g servings/day IC<br>Control IC group (N = 99**)**<br>**Duration of intervention:** 6 months<br>**Duration of follow-up:** 6 months<br>**Run-in period:** no<br>**Number of study centres:** 1 |
| **Outcomes** | **Reported outcomes in full text of publication:**<br>Haemoglobin rate/level, Se ferritin, Se iron, CRP, transferrin, frequencies of anaemia, nutrititon status, iron deficiency, iron deficiency anaemia, weight, height, weight-for-age *z*-scores, height-for-age, weight-for-height *z*-score<br>**Primary outcomes:** seven parameters:<br>hemoglobin, serum ferritin adjusted to CRP, serum iron, transferrin saturation, prevalence of anemia, iron deficiency, iron deficiency anemia.<br>**Secondary outcomes:** changes in weight, height, and other anthropometric z-scores<br>**Timing of outcome assessment: baseline, 3 months, 6 months** |
| **Identification** | **Trial identifier:** PACTR201802003069111<br>**Trial terminated early:** no |
| **Publication details** | **Language of publication:** English<br>**Funding:** Nestlé Nutrition Institute of Africa; Helen Keller Foundation for Research and Education; Cameroon Ministry of Public Health; UNICEF; National Statistics Institute<br>**Conflict of interest**: „NPH is employed by Société des Produits Nestlé SA. No other author has any conflict of interest to report”<br>**Publication status:** full article in peer-reviewed journal |
| **Stated aim for study** | **Quote: “**To evaluate the efficacy of iron fortified wheat flour for the correction and the prevention of iron deficiency anaemia among 18-59 months old children in Salapoumbé in Cameroon.” |
| **Note** | **Study start date:** February 2017 |

| | |
|---|---|
| | **Study end date:** August 2017 |

| **Study (Covidence identifier)** | **Gannon 2019** [5] (#6180) |
|---|---|
| **Methods** | **Study design:** cross-over randomised controlled trial<br>**Unit of randomisation:** individual (mother infant pairs)<br>**Blinding:** no data<br>**Number of study arms:** 2 arms |
| **Participants** | **Location/Setting**: „near Madanapalle, Andrhra Pradesh"<br>**Country where trial was performed:** India<br>**Sample size:** 52 children-mother pairs<br>**Dropouts/withdrawals:** „Twelve participant pairs attended for less than 6 days. Of the remaining, median (Q1, Q3) daily attendance compliance was 62.8% (44.1%, 83.5%)."<br>**Sex:** both male and female children included<br>**Inclusion criteria:** age: 6- to 24-months old<br>**Exclusion criteria:**<br>• dietary allergies<br>• currently diagnosed with malaria or dengue<br>• ever diagnosed with HIV or tuberculosis, or severe malnutrition (ie, weight-for-length Z-score [WLZ] < 3) determined using World Health Organization (WHO) field tables<br>**Health status:** no data<br>**Ongoing treatment:** no data<br>**Anaemic status:** no data<br>**Age range at start of intervention:** 6-24 months<br>**Mean age:** 14.3 (5.6) months |
| **Interventions** | **Intervention(s):**<br>• Multiple biofortified food crops, three times per day, six days per week, median daily intake was 75 g, (n = 52)<br>**Comparator(s):**<br>• Commercially available non-fortified food crops, three times per day, six days per week, median daily intake was 75 g, (n = 52)<br>**Duration of intervention:** 3 days<br>**Duration of follow-up:** 3 days<br>**Run-in period:** –<br>**Number of study centres:** 2 feeding centers |
| **Outcomes** | **Reported outcomes in full text of publication:** acceptability<br>**Primary outcomes:** not defined<br>**Secondary outcomes:** –<br>**Timing of outcome assessment: after each feeding** |
| **Identification** | **Trial identifier:** NCT02648893; IRB #: 1508005782<br>**Trial terminated early:** no |
| **Publication details** | **Language of publication:** English<br>**Funding:** non-commercial (HarvestPlus, grant number #2015H8336 awarded to Cornell University)<br>**Conflict of interest**: „The author(s) declared the following potential conflicts of interest with respect to the research, authorship, and/ or publication of this article: S.M. is an unpaid board member for a diagnostic startup focused on developing point-of-care assays for nutritional status informed by his research as a faculty member at Cornell University."<br>**Publication status:** full article in peer-reviewed journal |
| **Stated aim for study** | **Quote: "**To determine whether biofortified or industrially fortified maize consumption by Zambian women and their breastfeeding infants could improve milk retinol concentration and infant TBS." |
| **Note** | this paper reports short-term results of a longer, ongoing study<br>**Study start date:** December 2017<br>**Study end date:** April 2018 |

| **Study (Covidence identifier)** | **Huey 2018** [6] (#3636/1) |
|---|---|
| **Methods** | **Study design:** cross-over, controlled clinical trial<br>**Unit of allocation:** individual<br>**Blinding:** no data<br>**Number of study arms:** 2 arms |
| **Participants** | **Location/Setting**: a feeding center within a large slum known locally as Nehru Nagar, in Vile Parle, a suburb in Mumbai |

| | |
|---|---|
| | **Country where trial was performed:** India<br>**Sample size:** 125<br>**Dropouts/withdrawals:** no information<br>**Sex:** both male and female children included<br>**Inclusion criteria:** –<br>**Exclusion criteria:** –<br>**Age at the start of the intervention:** 12-24 month<br>**Country where trial was performed:** India |
| **Interventions** | **Intervention(s):**<br>• FeZnPM (iron- and zinc-biofortified pearl millet); 18 types of different recipies (n = 125**)**<br>**Comparator(s):**<br>• CtrlPM (conventional pearl millet) (n = 125)<br>**Duration of intervention:** 3 days<br>**Duration of follow-up:** 3 days<br>**Run-in period:** yes, 3 days<br>**Number of study centres:** 1 feeding center |
| **Outcomes** | **Reported outcomes in full text of publication:**<br>acceptability |
| **Identification** | **Trial identifier:** –<br>**Trial terminated early:** no |
| **Publication details** | **Language of publication:** English<br>**Funding:** non-commercial **(**HarvestPlus; 2014H8302)<br>**Conflict of interest**: „SM is an unpaid board member for a diagnostic start up focused on developing point-of-care assays for nutritional status informed by his research as a faculty member at Cornell University. All other authors declare that the research was conducted in the absence of any commercial or financial relationships that could be construed as a potential conflict of interest<br>**Publication status:** full article in peer-reviewed journal |
| **Stated aim for study** | **Quote: "**The main objective of this study was to formulate and test the acceptability (in terms of volume consumed and sensory characteristics) of new pearl millet-based palatable complementary food products for weaning infants. The food products with highest acceptability would be ideal candidates for a randomized controlled trial testing the efficacy of biofortified pearl millet for improving iron status in infants and young children" |
| **Note** | This is an acute study. Based on the results a longer-term study is planned (Mehta 2017)<br>**Study start date:** January 2015<br>**Study end date:** December 2015 |

| **Study (Covidence identifier)** | **Ma 2016** (Sheng 2019; Krebs 2013) [7-10]<br>(#3906) |
|---|---|
| **Methods** | **Study design:** cluster randomized, non-masked, controlled efficacy intervention trial ("this study was a cross-sectional sub-sample nested within a larger intervention")<br>**Unit of randomisation:** cluster (60 villages clustered, 9 districts in Xichou County)<br>**Blinding:** nonmasked<br>**Number of study arms:** 3 arms |
| **Participants** | **Location/Setting**: Xichou county in Yunnan province<br>**Country where trial was performed:** China<br>**Sample size:** 1465 (954 allocated to the arms relevant to this systematic review)<br>**Dropouts/withdrawals:**149 (123 moved away, 22 refused to participate, 2 died, 2 were visited out of the range of the scheduled age)<br>**Sex:** both male and female children included<br>**Inclusion criteria:**<br>• healthy singleton infants between 3–5 months of age<br>• born between 37 to 42 weeks gestational age<br>• born with birth weight >2 000 g<br>• with no metabolic or physical problems<br>• lack of acute or chronic illness<br>• being exclusively breastfed<br>**Exclusion criteria:** –<br>**Health status:** healthy infants<br>**Ongoing treatment:** no data<br>**Anaemic status:** mixed<br>**Age range at start of intervention:** 6 months<br>**Mean age:** – |
| **Interventions** | **Intervention(s):**<br>• fortified infant rice cereal (commercial infant rice cereals (Nestle), fortified with iron, zinc and vitamin B12); 20g/day (n=419)<br>• red meat (50g/day) (n=461)<br>**Comparator(s):**<br>• local [10]infant rice cereal (from a mixture of glutinous rice flour, white granulated sugar and honey), 20g/day (n= 436) |

| | |
|---|---|
| | **Duration of intervention:** 12 months<br>**Duration of follow-up:** 12 months<br>**Run-in period:** 1-3 months after enrolment (no intervention)<br>**Number of study centres:** 1 |
| **Outcomes** | **Reported outcomes in full text of publication:**<br>WAZ, LAZ, WLZ, serum B12, Hb, body iron, ferritin, B12 concentration, MCV, MCH, MCHC, cognitive score, fine motor score, gross motor score, anaemia<br>**Primary outcomes: based on clinicaltrial.com:**<br>Linear Growth [Time Frame: 6-18 mos of age]<br>**Secondary outcomes: based on clinicaltrial.com:**<br>Morbidity [Time Frame: 6-18 mos of age]<br>Cognitive development [Time Frame: 0-18 mo of age]<br>Zn absorption [Time Frame: 9 and 18 mos of age]<br>**Timing of outcome assessment:** 6, 12 and 18 months were measured antropometric data, venous blood samples and cognitive scale and the fine motor and gross motor subtests were collected at the end of intervention. |
| **Identification** | **Trial identifier:** NCT0072610<br>**Trial terminated early:** no |
| **Publication details** | **Language of publication:** English<br>**Funding:** non-commercial (National Natural Science Foundation of China and Thrasher Foundation)<br>**Conflict of interest:** "The authors declare that they have no conflicts of interest"<br>**Publication status:** full article in peer-reviewed journal |
| **Stated aim for study** | **Quote: "**Our objective was to compare iron status at 18 months and growth from 6 to 18 months in rural poor toddlers fed 3 different complementary foods." |
| **Note** | **Study start date:** March 2009<br>**Study end date:** December 2011 |

| **Study (Covidence identifier)** | **Arcanjo, 2012** [11]<br>(#2936) |
|---|---|
| **Methods** | **Study design:** cluster-randomised trial<br>**Unit of randomisation:** cluster (2 day-care centers)<br>**Blinding:** double-blind, pacebo-controlled<br>**Number of study arms:** 2 arms |
| **Participants** | **Location/Setting**: City of Morrinhos—Ceara<br>**Country where trial was performed:** Brazil<br>**Sample size:** 216<br>**Dropouts/withdrawals:** 10 (3 left center, 5 absentee, 2 non-compliant)<br>**Sex:** both male and female children included<br>• Center A: 60:40 (male:female)<br>• Center B: 54:44 (male:female)<br>**Inclusion criteria:**<br>• age: 10-23 months<br>**Exclusion criteria:**<br>• infants already taking iron supplements<br>**Health status:** no data<br>**Ongoing treatment:** no data<br>**Anaemic status:** mixed (Anemia prevalence in the study population was estimated at 40%)<br>**Age range at start of intervention:** 10-23 months<br>**Mean age:**<br>• Center A: 16.4 (4.77)<br>Center B: 15.8 (4.27) |
| **Interventions** | **Intervention(s):**<br>• fortified rice (Ultrarice), containing 56.4 mg elemental iron (micronized ferric pyrophosphate/ 50 g portion (n=100)<br>**Comparator(s):**<br>• standard rice (n=98)<br>**Duration of intervention:** 18 weeks<br>**Duration of follow-up:** 18 weeks<br>**Run-in period:** no<br>**Number of study centres: 1** |
| **Outcomes** | **Reported outcomes in full text of publication:** Hb concentration, anaemia prevalence<br>**Primary outcomes:** hemoglobin values (before and after intervention), anemia (Hb < 110 g/L)<br>**Secondary outcomes:** –<br>**Timing of outcome assessment:** before and after trail |
| **Identification** | **Trial identifier:** –<br>**Trial terminated early:** no |
| **Publication details** | **Language of publication:** English<br>**Funding:** non-commercial (Santa Casa de Misericórdia de Sobral Hospital- Research Initiative Grant)<br>**Conflict of interest:** no data<br>**Publication status:** full article in peer-reviewed journal |

| | |
|---|---|
| **Stated aim for study** | **Quote:** **"**„to evaluate the impact of iron-fortified rice (Ultrarice) weekly on hemoglobin and anemia levels compared with standard rice (control)" |
| **Note** | **Study start date:** August 2010<br>**Study end date:** December 2010 |

| **Study (Covidence identifier)** | **Arcanjo 2013** [12]<br>(#2937) |
|---|---|
| **Methods** | **Study design:** cluster-randomised, controlled trial<br>**Unit of randomisation:** cluster (two day-care center)<br>**Blinding:** no data<br>**Number of study arms:** 2 arms |
| **Participants** | **Location/Setting**: City of Sobral<br>**Country where trial was performed:** Brazil<br>**Sample size:** 171<br>**Dropouts/withdrawals:** 7 (1 left center, 5 absentee, 1 non-compliant)<br>**Sex:**<br>both male and female children included<br>• Center A: 39:35 (male:female)<br>• Center B: 34:41 (male:female)<br>**Inclusion criteria:**<br>• 10 to 23 months<br>• written parental consent<br>**Exclusion criteria:**<br>• Infants' parents who refused to participate<br>• infants already using iron supplementation<br>**Health status:** no data<br>**Ongoing treatment:** no data<br>**Anaemic status:** mixed<br>**Age range at start of intervention:** 10-23 months<br>**Mean age:**<br>• Center A: 17.8 (2.85)<br>Center B (18.0 (2.97) |
| **Interventions** | **Intervention(s):**<br>**Center A**: fortified rice (Ultrarice), containing 56.4 mg elemental iron (micronized ferric pyrophosphate/ 50 g portion (n =74 at baseline)<br>once weekly<br>**Comparator(s):**<br>• Center B: standard (household) rice (n =75 at baseline)<br>**Duration of intervention:** 18 weeks<br>**Duration of follow-up:** 18 weeks<br>**Run-in period:** no<br>**Number of study centres: 1** |
| **Outcomes** | **Reported outcomes in full text of publication:** hemoglobin values, anaemia prevalance (Hb < 110 g/L)<br>**Primary outcomes:** hemoglobin values, anaemia prevalance (Hb < 110 g/L)<br>**Secondary outcomes: –**<br>**Timing of outcome assessment: baseline and endpoint** |
| **Identification** | **Trial identifier: –**<br>**Trial terminated early:** no |
| **Publication details** | **Language of publication:** English<br>**Funding:** non-commercial (Federal University of Ceara - Research Initiative Grant)<br>**Conflict of interest:** no data<br>**Publication status:** full article in peer-reviewed journal |
| **Stated aim for study** | **Quote:** „to evaluate the impact of iron-fortified rice (Ultrarice) weekly on hemoglobin and anemia levels compared with standard rice (control)" |
| **Note** | **Study start date:** August 2010<br>**Study end date:** December 2010 |

| **Study (Covidence identifier)** | **Quintero 2011** [13]<br>(# 2309) |
|---|---|
| **Methods** | **Study design:** parallel, randomised controlled trial<br>**Unit of randomisation:** individual<br>**Blinding:** double-blind<br>**Number of study arms:** 2 |
| **Participants** | **Location/Setting**: the State of Mexico<br>**Country where trial was performed:** Mexico<br>**Sample size:** 395 infants and pre-schoolers<br>**Dropouts/withdrawals:** 5 cases at the end of the study<br>**Sex:** both male and female children included |

| | |
|---|---|
| | **Inclusion criteria:**<br>• 7 to 24 months<br>• no neurological diseases<br>• written informed consent<br>• municipality of residence<br>• condition of indigenismo<br>**Exclusion criteria:** –<br>**Health status: „**it was necessary that families resided in municipalities before mentioned, who did not have neurological diseases"<br>**Ongoing treatment:** no data<br>**Anaemic status:** no data<br>**Age range at start of intervention**: 7-24 monts<br>**Mean age:** 16 months |
| **Interventions** | **Intervention(s):**<br>• enriched maize flour:<br>**(**100 grams: 1.5 g of soybean meal (3%), 42.4 mg of iron, 33.3 mg of zinc, 120 mcg of vitamin A, 6,5 mg of niacin, 548 mcg of folic acid (n = 195)<br>**Comparator(s):**<br>• Control group: corn flour without fortification (n =200)<br>**Duration of intervention:** 10 months<br>**Duration of follow-up:** 10 months<br>**Run-in period:** no<br>**Number of study centres:** 14 |
| **Outcomes** | **Reported outcomes in full text of publication**: weight, height**,** nutritional status, weight for age z-score, weight for height z-score, mental and psychomotor development, blood haemoglobin levels<br>**Primary outcomes:** not defined<br>**Secondary outcomes:** not defined<br>**Timing of outcome assessment:** before and after trail |
| **Identification** | **Trial identifier:** –<br>**Trial terminated early:** no |
| **Publication details** | **Language of publication:** Spanish<br>**Funding:** commercial **(**DICONSA; "formerly CONASUPO, which is a majority state-owned company belonging to the Secretariat of Social Development of Mexico")<br>**Conflict of interest:** no data<br>**Publication status:** full article in peer-reviewed journal |
| **Stated aim for study** | **Quote:** "To evaluate the effect of the consumption of a corn flour enriched with 3% soy, vitamins and minerals, on the growth and development of infants and preschool children." |
| **Note** | **Study start date:** no data<br>**Study end date:** no data |

| **Study (Covidence identifier)** | **Bagni, 2009** [14 15]<br>(#5201) |
|---|---|
| **Methods** | **Study design:** cluster-randomised trial<br>**Unit of randomisation:** cluster (daycare centres)<br>**Blinding:** double-blind<br>**Number of study arms:** 2 |
| **Participants** | **Location/Setting**: Rio de Janeiro<br>**Country where trial was performed:** Brazil<br>**Sample size:** 354<br>**Dropouts/withdrawals: intervention group:** loss of 57 children (22.4%), loss of 8.6%, control group: loss of 65 children (25.5%), loss of 8.4%,<br>**Sex:** both male and female children included<br>**Inclusion criteria:**<br>• all preschoolers in the established age group attending day care centres at baseline<br>• had written consent from those responsible<br>**Exclusion criteria:** sickle cell anaemia, purpura<br>**Health status:** no data<br>**Ongoing treatment:** no data<br>**Anaemic status:** mixed ("Anemia was defined as hemoglobin <11.0g / dL"; "In the IG, the frequency of anemia was 39.1%, and in the CG, it was 44.7%")<br>**Age range at start of intervention:** 12-60 months<br>**Mean age:** no data |
| **Interventions** | **Intervention(s):**<br>• white rice fortified with Iron Bisglycine Chelate, rice was held once a week at lunchtime (90g) (n= 180)<br>**Comparator(s):**<br>• rice with placebo once a week (n = 174)<br>**Duration of intervention:** 16 weeks<br>**Duration of follow-up:** 16 weeks<br>**Run-in period:** no |

| | |
|---|---|
| | **Number of study centres:** – |
| **Outcomes** | **Reported outcomes in full text of publication:** frequency of anaemia, haemoglobin<br>**Primary outcomes:** frequency of anaemia, haemoglobin<br>**Secondary outcomes:** –<br>**Timing of outcome assessment:** before and after trail |
| **Identification** | **Trial identifier:** NCT00727545<br>**Trial terminated early:** no |
| **Publication details** | **Language of publication:** Portuguese<br>**Funding:** non-commercial (Universidade Federal do Rio de Janeiro)<br>**Conflict of interest:** no data<br>**Publication status:** full article in peer-reviewed journal |
| **Stated aim for study** | **Quote:** "to evaluate the effect of weekly rice fortification with iron on the frequency of anaemia and haemoglobin concentration in children from public daycare centres in the city of Rio de Janeiro" |
| **Note** | **Study start date:** March 2006<br>**Study end date:** December 2006 |

| **Study (Covidence identifier)** | **Nesamvuni 2005** [16]<br>(#3052) |
|---|---|
| **Methods** | **Study design:** randomised, parallel, intervention trail<br>**Unit of randomisation:** children and their household or families<br>**Blinding:** single-blind<br>**Number of study arms:** 2 |
| **Participants** | **Location/Setting**: Oukasie, Brits, in the North West Province<br>**Country where trial was performed:** South Africa<br>**Sample size:** 44 of children randomly assigned<br>**Dropouts/withdrawals:** 8 lost to follow-up<br>**Sex:** both male and female children included<br>**Inclusion criteria:**<br>• 1 –3-year-old children at the crèches and the well-baby clinic<br>• who had weight-for-age or height-for-age below the 5th percentile of the National Centre for Health Statistics (NCHS) reference (undernourished)<br>**Exclusion criteria:**<br>• any physical or mental disability (not on disability grant),<br>• severe forms of undernutrition (marasmus and kwashiorkor),<br>• children of mothers who recently relocated to the area<br>**Health status:** undernourished (had weight-for-age or height-for-age below the 5th percentile)<br>**Ongoing treatment**: no data<br>**Anaemic status:** no data<br>**Age range at start of intervention:** 1-3 years old<br>**Mean age:** no data |
| **Interventions** | **Intervention(s):**<br>• to 150 g of raw maize meal, 1700 IU vitamin A, 0.61 mg thiamine, 0.62 mg riboflavin and 0.56 mg pyridoxine were added (25 and 50 kg (depending on usual monthly consumption) of maize meal flour was provided to the families per month to replace all maize meal consumed by these households) (n =16)<br>**Comparator(s):**<br>• unfortified maize (n =20)<br>**Duration of intervention:** 12 months<br>**Duration of follow-up:** 12 months<br>**Run-in period:** no<br>**Number of study centres:** - |
| **Outcomes** | **Reported outcomes in full text of publication:** weight, height, haemoglobin, haematocrit, serum retinol and serum retinol-binding protein<br>**Primary outcomes:** haemoglobin, haematocrit, serum retinal and serum retinol-binding protein<br>**Secondary outcomes:** weight, height<br>**Timing of outcome assessment:** before and after trail |
| **Identification** | **Trial identifier:** –<br>**Trial terminated early:** no |
| **Publication details** | **Language of publication:** English<br>**Funding:** non-commercial (grants from the National Research Foundation, Potchefstroom University for Christian Higher Education) and commercial (Hoffman La Roche (Switzerland), Roche Vitamin and Fine Chemicals and a gift of maize from Maizecor)<br>**Conflict of interest:** no data<br>**Publication status:** full article in peer-reviewed journal |
| **Stated aim for study** | **Quote: "**To evaluate the effectiveness of a vitamin-fortified maize meal to improve the nutritional status of 1–3-year-old malnourished African children" |
| **Note** | **Study start date:** no data<br>**Study end date:** no data |

| Study (Covidence identifier) | Faber 2005 [17] (#6626) |
|---|---|
| Methods | **Study design:** parallel randomized controlled trial<br>**Unit of randomisation:** individual<br>**Blinding:** double-blind<br>**Number of study arms:** 2 |
| Participants | **Location/Setting**: The Valley of a Thousand Hills in KwaZulu-Natal province<br>**Country where trial was performed:** South Africa<br>**Sample size:** 361 infants<br>**Dropouts/withdrawals:** 72 lost to follow-up<br>**Sex:** both male and female children included<br>**Inclusion criteria:**<br>• aged 6 –12 mo<br>**Exclusion criteria:**<br>• their parent or legal guardian did not sign the consent form,<br>• birth weight <2500 g<br>• a baseline blood sample was not obtained,<br>• haemoglobin concentration <80 g/L<br>**Health status:** not defined<br>**Ongoing treatment:** no data<br>**Anaemic status:** mixed<br>**Age range at start of intervention:** 6-12 months<br>**Mean age:** 8.9 months |
| Interventions | **Intervention(s):**<br>• milled maize meal was fortified to supply 3 mg ß-carotene, 11 mg iron (ferrous fumarate), and 3 mg zinc (zinc sulfate) per 40 g dry product; ascorbic acid (sodium ascorbate) was added (56mg/40 g dry product); 110 µg copper, 10 µg selenium, 0.4 mg riboflavin, 0.15 mg vitamin B-6, 0.25 µg vitamin B-12, and 2.5 mg vitamin E per 40 g dry product; 2 sachets/d was recommended, consumed as either 1 or 2 meals (n =144)<br>**Comparator(s):**<br>• same porridge, but without the added micronutrients; 2 sachets/d was recommended, consumed as either 1 or 2 meals (n = 145)<br>**Duration of intervention:** 6 months<br>**Duration of follow-up:** 6 months<br>**Run-in period:** no<br>**Number of study centres:** 1 |
| Outcomes | **Reported outcomes in full text of publication:** motor development, weight, length, length-for-age, weight-for-age, and weight-for length—which were expressed as z scores, Hb concentration, serum ferritin, serum retinol, serum zinc<br>**Primary outcomes:** Hemoglobin concentration, serum ferritin concentration, serum retinol concentration, serum zinc concentration, motor development<br>**Secondary outcomes:** weight, length, length-for-age, weight-for-age, and weight-for length—which were expressed as z scores, stunting<br>**Timing of outcome assessment:** before and after trial |
| Identification | **Trial identifier:** –<br>**Trial terminated early:** no |
| Publication details | **Language of publication:** English<br>**Funding:** non-commercial (Thrasher Research Fund and the Community-based Health Programme of The Valley Trust and commercial (Tiger Food Brands Limited donated the fortified-porridge product)<br>**Conflict of interest**: None of the authors had any personal or financial conflict of interest.<br>**Publication status:** full article in peer-reviewed journal |
| Stated aim for study | **Quote:** „We assessed whether the fortified porridge could reduce anaemia and improve the micronutrient status and motor development of infants." |
| Note | **Study start date:** February 2002<br>**Study end date:** March 2003 |

| Study (Covidence identifier) | Schumann 2005 [18] (#1790) |
|---|---|
| Methods | **Study design:** parallel, randomised, controlled trial<br>**Unit of randomisation:** individual<br>**Blinding:** double-masked<br>**Number of study arms:** 3 |
| Participants | **Location/Setting**: Ciudad Peronia<br>**Country where trial was performed:** Guatemala<br>**Sample size:** 110 number of children randomly assigned<br>**Dropouts/withdrawals:** 13 lost to follow-up<br>**Sex:** both male and female children included<br>**Inclusion criteria:** |

| | |
|---|---|
| | • 12 to 36 months age<br>• a high susceptibility to anaemia (Hb value was in the range of 100 to 115 g $l^{-1}$)<br>• give informed consent<br>• abide by the dietary instructions involved in the 5-day-per-week bean administration<br>**Exclusion criteria:**<br>• recent use of vitamin or mineral preparations containing iron<br>• recent surgery<br>• diagnosed chronic gastric or intestinal diseases, or chronic infections<br>• used supplements during the intervention<br>• at any point the parents made the decision to withdraw them from the study<br>• moved from the house of residence and re-localisation was not possible<br>• child with Hb concentration of <115 g/l<br>**Health status:** an age group with high susceptibility to anaemia)<br>**Ongoing treatment:** no data<br>**Anaemic status:** anaemic (“moderately anaemic”; “high susceptibility toa naemia”; [Hb value was in the range of 100 to 115 g/l])<br>**Age range at start of intervention:** 12 to 36 months age<br>**Mean age:** 20,9 months |
| **Interventions** | **Intervention(s):**<br>• $FeSO_4$ (inorganic salt) fortified black bean paste; 156-g cans for 5 days of a week with 31.2 mg of fortification iron (n=37)<br>• Haem-fortified (from bovine blood) black bean paste; 35.0 mg Fe/can for 5 days of a week (n=36)<br>**Comparator(s):**<br>• basic black bean paste for 5 days of a week (n =37)<br>**Duration of intervention:** 10 weeks<br>**Duration of follow-up:** 10 weeks<br>**Run-in period:** no<br>**Number of study centres:** 1 |
| **Outcomes** | **Reported outcomes in full text of publication:** Hb concentration, Ferritin concentration<br>**Primary outcomes:** Hb, Ferritin<br>**Secondary outcomes:** –<br>**Timing of outcome assessment:** baseline, 5 and 10 weeks |
| **Identification** | **Trial identifier:** –<br>**Trial terminated early:** no |
| **Publication details** | **Language of publication:** English<br>**Funding:** no data<br>**Conflict of interest:** no data<br>**Publication status:** full article in peer-reviewed journal |
| **Stated aim for study** | **Quote:** „Haem iron as a fortificant was compared with FeSO4 and a placebo treatment for the restoration of Hb and the incrementing of circulating ferritin as an index of iron stores" „The goal was to produce the same iron fortification that, with the intrinsic iron content, would total approximately 35.0 mg Fe per can in haem iron-fortified as well as in inorganic iron-fortified beans." |
| **Note** | **Study start date:** no data<br>**Study end date:** no data |

| **Study (Covidence identifier)** | **Lartey, 2000** [19 20]<br>(#4145) |
|---|---|
| **Methods** | **Study design:** randomized, controlled trial<br>**Unit of randomisation:** individual<br>**Blinding:** no data<br>**Number of study arms:** 4 |
| **Participants** | **Location/Setting**: Techiman (district capital is located about 400 km north of Accra)<br>**Country where trial was performed:** Ghana<br>**Sample size:** 216 infants<br>**Dropouts/withdrawals:** 18 during the intervention (7 because child's mother left the area, 1 because the father refused participation, 4 because the infant rejected the project food (2 for WM, 1 for WF, and 1 for KF), 4 because the mother did not feed the project food (2 for WF and 2 for KF), and the death of infant (n = 1).<br>**Sex:** both male and female children included<br>**Inclusion criteria:**<br>• breast-fed<br>• no health complication<br>• birth weight ≥2.5 kg<br>• no congenital abnormalities<br>• assigned a Maternal and Child Health card<br>• the child's mother was not planning to travel or move out of study area during the study period<br>**Exclusion criteria:** –<br>**Health status:** healthy<br>**Ongoing treatment:** no data<br>**Anaemic status:** mixed |

| | |
|---|---|
| | **Age range at start of intervention:** 6 months<br>**Mean age:** 6 months |
| **Interventions** | **Intervention(s):**<br>• Weanimix plus vitamins and minerals (Iron, zinc, calcium, vitamin A, riboflavin) (WM), (High and low refer to 2 formulations of Weanimix with vitamins and minerals added: "high" for infants consuming ≤60 g/d and "low" for infants consuming > 60 g/d of the food.) (n=47)<br>• Weanimix plus fish (smoked anchovy) powder (WF), (n=48)<br>• koko (fermented maize dough) plus fish powder (KF) (n= 45)<br>**Comparator(s):**<br>• Weanimix (75% maize (corn), 15% soybeans, and 10% groundnuts (peanuts) (n = 50)<br>**Duration of intervention:** 6 months<br>**Duration of follow-up:** 6 months<br>**Run-in period:** no<br>**Number of study centres:** 1 |
| **Outcomes** | **Reported outcomes in full text of publication:**<br>plasma zinc, plasma retinol, erythrocyte riboflavin, haemoglobin, haematocrit, plasma ferritin, plasma transferrin saturation, plasma transferrin, breastfeeding, iron intake, zinc intake, Vitamin A intake, riboflavin intake, weight-for-age z-score, length-for-age z score, weight gain, length gain, midupper arm circumference, head circumference, triceps skinfold thickness, subscapular skinfold thickness, midupper arm fat area, midupper arm muscle area, haemoglobin, haematocrit, plasma ferritin saturation, plasma ferritin, diarrhea, fever, respiratory illness, dietary intake (weighed food and beverages, energy intake, nutrient intake), anaemia<br>**Primary outcomes:** not defined<br>**Secondary outcomes:** not defined<br>**Timing of outcome assessment:** monthly anthropometric and morbidity data; baseline and after treatment blood draw |
| **Identification** | **Trial identifier:** –<br>**Trial terminated early:** no |
| **Publication details** | **Language of publication:** English<br>**Funding:** commercial (Roche Vitamins and Fine Chemicals) and non-commercial (Nestlé Foundation, a Rockefeller Foundation African Dissertation Internship Award, a Fulbright Scholarship)<br>**Conflicts of interest:** no data<br>**Publication status:** full article in peer-reviewed journal |
| **Stated aim for study** | **Quote: "**to compare the growth and micronutrient (iron, zinc, riboflavin, and vitamin A) status of Ghanaian infants 6–12 mo of age fed Weanimix (W) or 1 of 3 other improved complementary foods"; "This study describes the factors associated with hemoglobin and plasma ferritin, zinc and retinol concentrations and erythrocyte riboflavin status among 208 Ghanaian infants who participated in a complementary feeding intervention trial from 6 to 12 mo of age." |
| **Note** | **Study start date:** November 1994<br>**Study end date:** April 1995 |

| **Study (Covidence identifier)** | **Bovell-Benjamin 1999** [21]<br>(#7084) |
|---|---|
| **Methods** | **Study design:** cross-over randomized controlled trial<br>**Unit of randomisation:** individual (mother-toddler pairs)<br>**Blinding:** double-blind<br>**Number of study arms:** 3 |
| **Participants** | **Location/Setting**: -<br>**Country where trial was performed:** USA<br>**Sample size:** 40 mothers-toddlers pairs<br>**Dropouts/withdrawals:** 2 mothers-toddlers pairs (because they did not test all three porridges at one of the three test sessions)<br>**Sex:** both male and female toddlers included with their mothers<br>**Inclusion criteria:**<br>• eating infant cereals<br>• able to eat from a spoon<br>• not allergic to milk or maize<br>**Exclusion criteria:**<br>• not sample the porridge<br>**Health status:** no data<br>**Ongoing treatment:** no data<br>**Anaemic status:** no data<br>**Age range at start of intervention:** 6-24 months<br>**Mean age:** 13,5 ± 4,8 months |
| **Interventions** | **Intervention(s):** |

| | |
|---|---|
| | • whole maize fortified with ferrous bisglycinate (30 mg iron/kg) in their home, at the toddlers' regular mealtime and largest meal of the day. They recieved in 65 ml cup, but mothers were free to feed toddlers additional porridge (n = 38)<br>• maize fortified with ferrous bisglycinate and containing the antioxidant butylated hydroxyanisole (50 ppm) in their home, at the toddlers' regular mealtime and largest meal of the day. They received in 65 ml cup, but mothers were free to feed toddlers additional porridge. (n= 38)<br>**Comparator(s):**<br>• unfortified whole maize in their home, at the toddlers' regular mealtime and largest meal of the day. They received in 65 ml cup, but mothers were free to feed toddlers additional porridge (n = 38)<br>**Duration of intervention:** 3 subsequent sessions<br>**Duration of follow-up:** 3 subsequent sessions<br>**Run-in period:** no<br>**Number of study centres:** 1 |
| **Outcomes** | **Reported outcomes in full text of publication:** DOL (degree of liking) among porridges<br>**Primary outcomes:** not defined<br>**Secondary outcomes:** –<br>**Timing of outcome assessment:** After each sample was tested, mothers indicated the toddlers' degree of liking of the sample |
| **Identification** | **Trial identifier:** –<br>**Trial terminated early:** no |
| **Publication details** | **Language of publication:** English<br>**Funding:** Albion Laboratories, Clearfield, CT<br>**Conflict of interest**: no data<br>**Publication status:** full article in peer-reviewed journal |
| **Stated aim for study** | **Quote:** **"**Our ultimate goal is to use BIS as an iron fortificant in infants' and children's cereals in developing countries to reduce the high prevalence of ID and IDA." |
| **Note** | **Study start date:** no data<br>**Study end date:** no data |

| **Study (Covidence identifier)** | **Liu 1993** [22]<br>(#4438) |
|---|---|
| **Methods** | **Study design:** cluster randomized trial<br>**Unit of randomisation:** cluster<br>**Blinding:** N/A<br>**Number of study arms: 2** |
| **Participants** | **Location/Setting**: Mi-yun rural area near Beijing<br>**Country where trial was performed:** China<br>**Sample size:** 164<br>**Dropouts/withdrawals:** N/A no data<br>**Sex:** both male and female children included<br>**Inclusion criteria:**<br>• healthy full-term infants.<br>• born without complication<br>• born with birth weights > 2.5 kg.<br>• aged 6-13 mo<br>• enrolled from 33 villages of the Mi-yun rural area near Beijing<br>**Exclusion criteria:**<br>• not consumed all the rusks<br>• hemoglobin concentrations <100 g/L<br>**Health status:** No clinical deficiency signs attributable to micronutrient deficiencies were observed in any of the children**.**<br>**Ongoing treatment:** no data<br>**Anaemic status:** mixed ("Of the children in the present study, only 15% were anemic at the outset (hemoglobin < 1 10 g/L)")<br>**Age range at start of intervention**: 6-13 mo<br>**Mean age:** T: 9,63 mo; C: 9,86 mo |
| **Interventions** | **Intervention(s):**<br>• Calcium (300 mg/day), ferric ammonium citrate (5 mg/day), zinc (3 mg/day), vitamin A (224ug/day), cholecalciferol (4 ug/day), thiamine (0.15 ug/day), riboflavin (0.2 mg/day), niacin (2.5 mg/day), cyanocobalamin (0.3 ug/day), folic acid (25 ug/day) fortified rusk (17 g) daily supply (n =77)<br>**Comparator(s):**<br>• unfortified rusks daily supply (n =87)<br>**Duration of intervention:** 3 months<br>**Duration of follow-up:** 3 months<br>**Run-in period:** no<br>**Number of study centres:** 1 |
| **Outcomes** | **Reported outcomes in full text of publication:** Weight, length, free erythrocyte porphyrin in red cells, plasma ferritin, eryihrocyte glutathione reductase activation coefficient, plasma vitamin E and plasma retinol, hemoglobin concentration<br>**Primary outcomes:** not defined |

| | |
|---|---|
| | **Secondary outcomes:** not defined<br>**Timing of outcome assessment:** before trail, after trail |
| **Identification** | **Trial identifier: -**<br>**Trial terminated early:** no |
| **Publication details** | **Language of publication:** English<br>**Funding:** Supported in part by a grant from the United Kingdom Department of Trade and Industry.<br>**Conflict of interest:** no data<br>**Publication status:** full article in peer-reviewed journal |
| **Stated aim for study** | **Quote: "**A micronutrient-fortified rusk for weanling children was tested in a rural area near Beijing." "The purpose of the study was to investigate the efficacy of the micronutrients addition not of the rusk per se." |
| **Note** | **Study start date:** February 1990<br>**Study end date:** June 1990 |

| **Study (Covidence identifier)** | **Gershoff 1977** (Gershoff 1975) [23 24]<br>(#6115) |
|---|---|
| **Methods** | **Study design:** parallel cluster randomized trial<br>**Unit of randomisation:** cluster<br>**Blinding:** no data<br>**Number of study arms:** 5 |
| **Participants** | **Location/Setting**: province of Chiang Mai<br>**Country where trial was performed:** Thailand<br>**Sample size:** 1265 children at the start; 2250 at the end (<2 years: 357)<br>**Dropouts/withdrawals:** no data<br>**Sex:** both male and female children included<br>**Inclusion criteria: -**<br>**Exclusion criteria:** "villages not selected where there is iodine deficiency area"<br>**Health status:** no data<br>**Ongoing treatment:** no data<br>**Anaemic status**: no data<br>**Age range at the start of intervention:** 6 months to 5 years<br>**Mean age:** no data |
| **Interventions** | **Intervention(s):** daily received<br>• RFG1: rice fortification grain 1 contains 0.0873% thiamine naphthalene disulfonate (equivalent to 0.05% thiamine nitrate), 0.0.4% retinol (added starting in August of 1972), 0.0815% of retinol acetate, and 0.8% $FePO_4$ $4H_2O$ (0.2% iron), lysine HCI and 10% L-threonine. (n= no data)<br>• RFG2: rice fortification grain 1 contains 0.0873% thiamine naphthalene disulfonate (equivalent to 0.05% thiamine nitrate), 0.0.4% retinol (added starting in August of 1972), 0.0815% of retinol acetate, and 0.8% $FePO_4$ $4H_2O$ (0.2% iron), no amino acids (n= no data)<br>**Comparator(s):**<br>• RFG3: Rice fortification grains (daily received) (n= no data)<br>**Duration of intervention:** 4 years<br>**Duration of follow-up:** 4 years<br>**Run-in period:** no<br>**Number of study centres:** 29 |
| **Outcomes** | **Reported outcomes in full text of publication:**<br>• data from anthropometric measurements (length, weight, bone age, head circumference, chest circumference, arm circumference, triceps skinfold, subscapular skinfold)<br>• haemoglobin, haematocrit<br>• morbidity data<br>• handwrist x-ray<br>**Primary outcomes:** not defined<br>**Secondary outcomes:** not defined<br>• Timing of outcome assessment: Two physical examinations and haemoglobin and haematocrit values were conducted every year. Morbidity data were collected on each child every 15 days for up to 3 years. |
| **Identification** | **Trial identifier:** –<br>**Trial terminated early:** no |
| **Publication details** | **Language of publication:** English<br>**Funding:** Supported in part by Contract AID/CSD-3291 from the United States Agency for International Development and the Fund for Research and Teaching, Department of Nutrition, Harvard School of Public Health.<br>**Publication status:** full article in peer-reviewed journal<br>**Conflict of interest**: no data |
| **Stated aim for study** | **Quote: "**a large-scale field study in villages of northern Thailand designed to measure the health benefits to be derived from the fortification of rice with lysine, threonine, thiamine, riboflavin, vitamin A and iron" |
| **Note** | **Study start date:** January 1971<br>**Study end date:** July 1975 |

**References**


1. Palmer AC, Jobarteh ML, Chipili M, et al. Biofortified and fortified maize consumption reduces prevalence of low milk retinol, but does not increase vitamin A stores of breastfeeding Zambian infants with adequate reserves: a randomized controlled trial. *Am J Clin Nutr* 2021 doi: 10.1093/ajcn/nqaa429
2. Johns Hopkins Bloomberg School of Public H, University of California D, Newcastle U, et al. Efficacy of Biofortified Maize to Improve Maternal and Infant Vitamin A Status, 2017.
3. Ekoe T, Bianpambe OI, Nguefack F, et al. Efficacy of an iron-fortified infant cereal to reduce the risk of iron deficiency anemia in young children in East Cameroon. *Food sci* 2020;8(7):3566-77. doi: https://dx.doi.org/10.1002/fsn3.1639
4. Pactr. Efficacy of an iron fortified wheat flour for the correction and the prevention of iron deficiency anaemia of children in Salapoumbe. *http://wwwwhoint/trialsearch/Trial2aspx?TrialID=PACTR201802003069111* 2018
5. Gannon BM, Thakker V, Bonam VS, et al. A Randomized Crossover Study to Evaluate Recipe Acceptability in Breastfeeding Mothers and Young Children in India Targeted for a Multiple Biofortified Food Crop Intervention. *Food Nutr Bull* 2019;40(4):460-70. doi: https://dx.doi.org/10.1177/0379572119855588
6. Huey SL, Venkatramanan S, Udipi SA, et al. Acceptability of Iron- and Zinc-Biofortified Pearl Millet (ICTP-8203)-Based Complementary Foods among Children in an Urban Slum of Mumbai, India (vol 4, 39, 2017). *Front Nutr* 2018;5:2. doi: 10.3389/fnut.2018.00092
7. Ma J, Sun Q, Liu J, et al. The Effect of Iron Fortification on Iron (Fe) Status and Inflammation: A Randomized Controlled Trial. *PLoS ONE* 2016;11(12):e0167458. doi: https://dx.doi.org/10.1371/journal.pone.0167458
8. Sheng XY, Wang JL, Li F, et al. Effects of dietary intervention on vitamin B-12 status and cognitive level of 18-month-old toddlers in high-poverty areas: a cluster-randomized controlled trial. *BMC Pediatr* 2019;19(1):9. doi: 10.1186/s12887-019-1716-z
9. Krebs NF, Sheng XY, Westcott JE, et al. Higher cognitive and gross motor scores in Chinese toddlers randomized to meat compared to either micro-nutrient fortified or unfortified infant cereal as first complementary food. *Faseb J* 2013;27:1.
10. University of Colorado D, Shanghai Jiao Tong University School of M, Xi-Chou W, et al. Development and Health of Rural Chinese Children Fed Meat as a Daily Complementary Food From 6-18 Mos of Age, 2012.
11. Nogueira Arcanjo FP, Santos PR, Arcanjo CP, et al. Use of iron-fortified rice reduces anemia in infants. *J Trop Pediatr* 2012;58(6):475-80. doi: https://dx.doi.org/10.1093/tropej/fms021
12. Nogueira Arcanjo FP, Roberto Santos P, Madeiro Leite AJ, et al. Rice fortified with iron given weekly increases hemoglobin levels and reduces anemia in infants: a community intervention trial. *Int J Vitam Nutr Res* 2013;83(1):59-66. doi: https://dx.doi.org/10.1024/0300-9831/a000145
13. Quintero MDC, Hernandez LO, Villasana AC, et al. IMPACT OF CONSUMPTION FO CORN FLOUR WITH LOW LEVEL ENRICHMENT IN CHILDREN OF RURAL ZONES. *Nutr Hosp* 2011;26(5):1097-104. doi: 10.3305/nh.2011.26.5.5127
14. Bagni UV, Baiao MR, Santos MM, et al. [Effect of weekly rice fortification with iron on anemia prevalence and hemoglobin concentration among children attending public daycare centers in Rio de Janeiro, Brazil]. *Cad Saude Publica* 2009;25(2):291-302.
15. Universidade Federal do Rio de J. Effect of Rice Fortification With Iron on Anemia Among Children.
16. Nesamvuni AE, Vorster HH, Margetts BM, et al. Fortification of maize meal improved the nutritional status of 1-3-year-old African children. *Public Health Nutrition* 2005;8(5):461-67. doi: 10.1079/phn2005782
17. Faber M, Kvalsvig JD, Lombard CJ, et al. Effect of a fortified maize-meal porridge on anemia, micronutrient status, and motor development of infants. *Am J Clin Nutr* 2005;82(5):1032-9.
18. Schumann K, Romero-Abal ME, Maurer A, et al. Haematological response to haem iron or ferrous sulphate mixed with refried black beans in moderately anaemic Guatemalan pre-school children. *Public Health Nutr* 2005;8(6):572-81.
19. Lartey A, Manu A, Brown KH, et al. Predictors of micronutrient status among six- to twelve-month-old breast-fed Ghanaian infants. *J Nutr* 2000;130(2):199-207.
20. Lartey A, Manu A, Brown KH, et al. A randomized, community-based trial of the effects of improved, centrally processed complementary foods on growth and micronutrients status of Ghanaian infants from 6 to 12 mo of age Weanimix, a cereal-legume blend. *American Journal of Clinical Nutrition* 1999;70(3):391-404. doi: 10.1093/ajcn/70.3.391
21. Bovell-Benjamin AC, Allen LH, Guinard JX. Toddlers' acceptance of whole maize meal porridge fortified with Ferrous Bisglycinate. *Food Qual Prefer* 1999;10(2):123-28. doi: 10.1016/s0950-3293(98)00058-5
22. Liu DS, Bates CJ, Yin TA, et al. Nutritional efficacy of a fortified weaning rusk in a rural area near Beijing. *Am J Clin Nutr* 1993;57(4):506-11. doi: 10.1093/ajcn/57.4.50610.1093/ajcn/57.4.506.

23. Gershoff SN, McGandy RB, Suttapreyasri D, et al. Nutrition studied in Thailand. II. Effects of fortification of rice with lysine, threonine, thiamin, riboflavin, vitamin A, and iron on preschool children. *Am J Clin Nutr* 1977;30(7):1185-95.
24. Gershoff SN, McGandy RB, Suttapreyasri D, et al. Amino acid fortification of rice studies in Thailand. I. Background and baseline data. *The American journal of clinical nutrition* 1975;28(2):170-82. doi: 10.1093/ajcn/28.2.170

## Supplement 5. Information about malaria in the area of the trial

| Study | Country | Malaria presence in the region mentioned in the manuscript | Children with malaria included | Malaria endemic country * |
|---|---|---|---|---|
| **Palmer 2021**[1] | Zambia | yes | yes | yes |
| **Lartey 2000** [2 3] | Ghana | yes | yes | yes |
| **Ma 2016** [4] | China | no | - | no |
| **Arcanjo 2012** [5] | Brazil | no | - | yes |
| **Schumann 2005** [6] | Guatemala | yes | yes | yes |
| **Huey 2018** [7] | India | no | - | yes |
| **Faber 2005** [8] | South Africa | no | - | yes |
| **Liu 1993** [9] | China | no | - | no |
| **Nesamvuni 2005** [10] | South Africa | no | - | yes |
| **Bovell-Benjamin 1999** [11] | USA | no | - | no |
| **Gershoff 1977** [12] | Thailand | no | - | yes |
| **Gannon 2019** [13] | India | yes | no | yes |
| **Arcanjo 2013** [14] | Brazil | no | - | yes |
| **Bagni 2009** [15] | Brazil | no | - | yes |
| **Ekoe 2020** [16] | (East) Cameroon | yes | yes** | yes |
| **Quintero 2011** [17] | Mexico | no | - | yes |

*based on: Word Malaria Report, 10 years of Global Progress & Challenges, 2020
****** severe malaria cases were excluded

**References**

1. Palmer AC, Jobarteh ML, Chipili M, et al. Biofortified and fortified maize consumption reduces prevalence of low milk retinol, but does not increase vitamin A stores of breastfeeding Zambian infants with adequate reserves: a randomized controlled trial. *Am J Clin Nutr* 2021 doi: 10.1093/ajcn/nqaa429
2. Lartey A, Manu A, Brown KH, et al. A randomized, community-based trial of the effects of improved, centrally processed complementary foods on growth and micronutrients status of Ghanaian infants from 6 to 12 mo of age Weanimix, a cereal-legume blend. *American Journal of Clinical Nutrition* 1999;70(3):391-404. doi: 10.1093/ajcn/70.3.391
3. Lartey A, Manu A, Brown KH, et al. Predictors of micronutrient status among six- to twelve-month-old breast-fed Ghanaian infants. *J Nutr* 2000;130(2):199-207.
4. Ma J, Sun Q, Liu J, et al. The Effect of Iron Fortification on Iron (Fe) Status and Inflammation: A Randomized Controlled Trial. *PLoS ONE* 2016;11(12):e0167458. doi: https://dx.doi.org/10.1371/journal.pone.0167458
5. Nogueira Arcanjo FP, Santos PR, Arcanjo CP, et al. Use of iron-fortified rice reduces anemia in infants. *J Trop Pediatr* 2012;58(6):475-80. doi: https://dx.doi.org/10.1093/tropej/fms021
6. Schumann K, Romero-Abal ME, Maurer A, et al. Haematological response to haem iron or ferrous sulphate mixed with refried black beans in moderately anaemic Guatemalan pre-school children. *Public Health Nutr* 2005;8(6):572-81.
7. Huey SL, Venkatramanan S, Udipi SA, et al. Acceptability of Iron- and Zinc-Biofortified Pearl Millet (ICTP-8203)-Based Complementary Foods among Children in an Urban Slum of Mumbai, India (vol 4, 39, 2017). *Front Nutr* 2018;5:2. doi: 10.3389/fnut.2018.00092
8. Faber M, Kvalsvig JD, Lombard CJ, et al. Effect of a fortified maize-meal porridge on anemia, micronutrient status, and motor development of infants. *Am J Clin Nutr* 2005;82(5):1032-9.
9. Liu DS, Bates CJ, Yin TA, et al. Nutritional efficacy of a fortified weaning rusk in a rural area near Beijing. *Am J Clin Nutr* 1993;57(4):506-11. doi: 10.1093/ajcn/57.4.50610.1093/ajcn/57.4.506.
10. Nesamvuni AE, Vorster HH, Margetts BM, et al. Fortification of maize meal improved the nutritional status of 1-3-year-old African children. *Public Health Nutrition* 2005;8(5):461-67. doi: 10.1079/phn2005782
11. Bovell-Benjamin AC, Allen LH, Guinard JX. Toddlers' acceptance of whole maize meal porridge fortified with Ferrous Bisglycinate. *Food Qual Prefer* 1999;10(2):123-28. doi: 10.1016/s0950-3293(98)00058-5
12. Gershoff SN, McGandy RB, Suttapreyasri D, et al. Nutrition studied in Thailand. II. Effects of fortification of rice with lysine, threonine, thiamin, riboflavin, vitamin A, and iron on preschool children. *Am J Clin Nutr* 1977;30(7):1185-95.
13. Gannon BM, Thakker V, Bonam VS, et al. A Randomized Crossover Study to Evaluate Recipe Acceptability in Breastfeeding Mothers and Young Children in India Targeted for a Multiple Biofortified Food Crop Intervention. *Food Nutr Bull* 2019;40(4):460-70. doi: https://dx.doi.org/10.1177/0379572119855588
14. Nogueira Arcanjo FP, Roberto Santos P, Madeiro Leite AJ, et al. Rice fortified with iron given weekly increases hemoglobin levels and reduces anemia in infants: a community intervention trial. *Int J Vitam Nutr Res* 2013;83(1):59-66. doi: https://dx.doi.org/10.1024/0300-9831/a000145
15. Bagni UV, Baiao MR, Santos MM, et al. [Effect of weekly rice fortification with iron on anemia prevalence and hemoglobin concentration among children attending public daycare centers in Rio de Janeiro, Brazil]. *Cad Saude Publica* 2009;25(2):291-302.
16. Ekoe T, Bianpambe OI, Nguefack F, et al. Efficacy of an iron-fortified infant cereal to reduce the risk of iron deficiency anemia in young children in East Cameroon. *Food sci* 2020;8(7):3566-77. doi: https://dx.doi.org/10.1002/fsn3.1639
17. Quintero MDC, Hernandez LO, Villasana AC, et al. IMPACT OF CONSUMPTION FO CORN FLOUR WITH LOW LEVEL ENRICHMENT IN CHILDREN OF RURAL ZONES. *Nutr Hosp* 2011;26(5):1097-104. doi: 10.3305/nh.2011.26.5.5127

## Supplement 6. Composition of fortified complementary foods

| | | Composition of the non-fortified complementary food | | | |
|---|---|---|---|---|---|
| **Study number #** | **Type of comple-mentary food** | **Total Energy** | **Macronutrients (Protein, Fat Carbohydrate)** | **Micronutrients** | **Micronutrient(s) added to the fortified products** |
| **Palmer 2021** [1,2] | Maize meal | Not specified | Not specified | Not specified | Retinyl palmitate ~55 µg RE/d<br>or<br>provitamin A carotenoid-biofortified |
| **Gannon 2019** [3] | Crops | Not specified | Not specified | Not specified | Biofortified crops (not further specified) |
| **Ma 2016** [4-7] | Rice cereal | 80 kcal/day<br>(20 g/ day) | Not specified | Iron (0.04 mg/20g) | Iron (ferrous fumarate; 1.10 mg/20 g); zinc (zinc sulfate; amount not specified); vitamin B12 (amount not specified) |
| **Ekoe 2020** [8,9] | Cereal | 420 kcal/100g of cereal/day | Protein 14.5g; Fat 10 g; Carbo-hydrate 68 g/ 100g | Sodium 135 mg, Calcium 450 mg, Zinc 5 mg, Vitamin A 1,300 IU, Vitamin D 180 IU, Vitamin C 50 mg, Vitamin $B_1$ (thiamin) 0.6 mg/100g | Iron (ferrus fumarate; 7.5 mg/100g) |
| **Arcanjo 2013** [10] | Rice | Not specified<br>(~50g/day) | Not specified | Not specified | 56.4 mg ferric pyrophosphate/ 50 g portion |
| **Arcanjo 2012** [11] | Rice | Not specified | Not specified | Not specified | 56.4 mg ferric pyrophosphate/ 50 g portion |
| **Huey 2018** [12,13] | Pearl millet | Not specified | Not specified | Iron (21.24 ppm); zinc (19.34 ppm) | Iron (additional 61.5 ppm); Zinc (additional 14.83 ppm) |
| **Quintero 2011** [14] | Corn flour | Not specified<br>(20 kg flour/family/month) | Not specified | Iron 3.90 mg; zinc 2.00 mg; retinol 0.50 mcg; niacin 1.30mg/100 g flour | 1.5 g of soybean meal, iron 42.4mg, zink 33.3 mg, retinol 120 mcg, niacin 6.5 mg, 548mcg folic acid/ 100 g flour |
| **Bagni, 2009** [15,16] | Rice | Not specified<br><br>(80 g rice /once a week) | Not specified | Not specified | Iron (3.78 mg/once a week) |
| **Nesamvuni 2005** [17] | Maize meal | Not specified | Not specified | Not specified | 1700 IU vitamin A, 0.61 mg thiamine, 0.62 mg riboflavin, 0.56 mg pyridoxine/150g raw maize meal |
| **Faber 2005** [18] | Porridge | Dry product: 617 KJ/ day<br>(40 g /day) | Not specified | Not specified | 3mg ß-carotene; 11 mg iron (ferrous fumarate); 3 mg zinc (zinc sulfate); 56 mg ascorbic acid (sodium ascorbate); 110 µg copper; 10 µg selenium; 0.4 mg riboflavin; 0.15 mg vitamin B6; 0.25 µg vitamin B12; 2.5 mg vitaminE /40 g dry product |
| **Schümann 2005** [19] | Beans | Not specified<br>(5 cans/week) | Not specified | Iron (3.1 mg/can) | Iron ($FeSO_4$) 32.5 mg/can (20 mg/ 100 g beans)<br>or |

| | | | | | |
|---|---|---|---|---|---|
| | | | | | haem iron (from bovin blood) 34.0 mg/can (1.33 g haem powder/ 100 g beans) |
| **Lartey, 2000** [20 21] | Cereal-legume blend | 4350 kcal/ 1000 g dry weight | Protein 150 g (14E%); Fat 114 g (24E%)/1000 g | Calcium 530 mg, Iron 56 mg, Zinc mg 28, Copper 4 mg, Magnesium 1400 mg, Potassium 5660 mg, Sodium 30 mg, Phosphorus 2920 mg, Ascorbic acid 1 mg, Niacin 39 mg, Pyridoxine 3.5 mg, Riboflavin 0.4 mg, Thiamine 4.8 mg, Vitamin B12 0 µg, Folic acid 670 µg, Vitamin A 360 RE | <u>for infants consuming ≤60 g/d (content/1000 g dry weight)</u>: Calcium 17360 mg, Iron 366 mg, Zinc mg 171, Copper 25 mg, Magnesium 1400 mg, Potassium 18960 mg, Sodium 30 mg, Phosphorus 17900 mg, Ascorbic acid 781 mg, Niacin 259 mg, Pyridoxine 31.3 mg, Riboflavin 19.5 mg, Thiamine 22.1 mg, Vitamin B12 70 µg Folic acid 5470 µg, Vitamin A 18360 RE<br><u>for infants consuming >60 g/d of the food (content/ 1000 g dry weight</u>: Calcium 8950 mg, Iron 183 mg, Zinc mg 86, Copper 13 mg, Magnesium 1400 mg, Potassium 12310 mg, Sodium 30 mg, Phosphorus 9400 mg, Ascorbic acid 391 mg, Niacin 149 mg, Pyridoxine 17.4 mg, Riboflavin 9.8 mg, Thiamine 13.5 mg, Vitamin B12 35 µg, Folic acid 3070 µg, Vitamin A 9360 RE |
| **Bovell-Benjamin 1999** [22] | whole maize meal | Not specified | Not specified | Not specified | Ferrous bisglycinate 30 mg/kg maize meal |
| **Liu 1993** [23] | Rusk | 9.20 KJ/g rusk<br><br>155 KJ/17 g (1 portion rusk) | Protein: 0.06 g, Fat: 0.08 g, Carbohydrate (sugar): 0.31 g/ 1 g<br><br>Protein 1.0 g, Fat 1.4 g, Carbohydrate (sugar) 5.2 g/ portion (17 g) | Not specified | <u>Per gram</u>: Calcium 17.60 mg, Iron 0.29 mg, zinc 0.18 mg, vitamin A 13.20 µg (RE (provided as retinyl acetate), cholecalciferol 0.22 µg, thiamin 0.009 µg, riboflavin 0.012 mg, niacin 0.147 mg, cyanocobalamin 0.018 µg, folic acid 1.47 µg<br><br><u>Per rusk</u>: Calcium 300 mg, Iron 5.0 mg, zinc 3 mg, vitamin A 224 µg (RE (provided as retinyl acetate), cholecalciferol 4 µg, thiamin 0.15 µg, riboflavin 0.2 mg, niacin 2.5 mg, cyanocobalamin 0.3 µg, folic acid 25.0 µg |
| **Gershoff 1977** [24 25] | Rice | Not specified | Not specified | Not specified | Artifical rice grain conatained 0.0873% thiamin naphthalene disulfonate (equivalent to 0.05% thiamin nitrate), 0.04% riboflavin, 0.0815% retinol acetate, 0.8% $FePO_4*4H_2O$ (0.2% iron), no amino acid (RFG2) |

**References**

1. Palmer AC, Jobarteh ML, Chipili M, et al. Biofortified and fortified maize consumption reduces prevalence of low milk retinol, but does not increase vitamin A stores of breastfeeding Zambian infants with adequate reserves: a randomized controlled trial. *Am J Clin Nutr* 2021 doi: 10.1093/ajcn/nqaa429
2. Johns Hopkins Bloomberg School of Public H, University of California D, Newcastle U, et al. Efficacy of Biofortified Maize to Improve Maternal and Infant Vitamin A Status, 2017.
3. Gannon BM, Thakker V, Bonam VS, et al. A Randomized Crossover Study to Evaluate Recipe Acceptability in Breastfeeding Mothers and Young Children in India Targeted for a Multiple Biofortified Food Crop Intervention. *Food Nutr Bull* 2019;40(4):460-70. doi: https://dx.doi.org/10.1177/0379572119855588
4. Ma J, Sun Q, Liu J, et al. The Effect of Iron Fortification on Iron (Fe) Status and Inflammation: A Randomized Controlled Trial. *PLoS ONE* 2016;11(12):e0167458. doi: https://dx.doi.org/10.1371/journal.pone.0167458
5. Sheng XY, Wang JL, Li F, et al. Effects of dietary intervention on vitamin B-12 status and cognitive level of 18-month-old toddlers in high-poverty areas: a cluster-randomized controlled trial. *BMC Pediatr* 2019;19(1):9. doi: 10.1186/s12887-019-1716-z
6. Krebs NF, Sheng XY, Westcott JE, et al. Higher cognitive and gross motor scores in Chinese toddlers randomized to meat compared to either micro-nutrient fortified or unfortified infant cereal as first complementary food. *Faseb J* 2013;27:1.
7. University of Colorado D, Shanghai Jiao Tong University School of M, Xi-Chou W, et al. Development and Health of Rural Chinese Children Fed Meat as a Daily Complementary Food From 6-18 Mos of Age, 2012.
8. Ekoe T, Bianpambe OI, Nguefack F, et al. Efficacy of an iron-fortified infant cereal to reduce the risk of iron deficiency anemia in young children in East Cameroon. *Food sci* 2020;8(7):3566-77. doi: https://dx.doi.org/10.1002/fsn3.1639
9. Pactr. Efficacy of an iron fortified wheat flour for the correction and the prevention of iron deficiency anaemia of children in Salapoumbe. *http://wwwwhoint/trialsearch/Trial2aspx?TrialID=PACTR201802003069111* 2018
10. Nogueira Arcanjo FP, Roberto Santos P, Madeiro Leite AJ, et al. Rice fortified with iron given weekly increases hemoglobin levels and reduces anemia in infants: a community intervention trial. *Int J Vitam Nutr Res* 2013;83(1):59-66. doi: https://dx.doi.org/10.1024/0300-9831/a000145
11. Nogueira Arcanjo FP, Santos PR, Arcanjo CP, et al. Use of iron-fortified rice reduces anemia in infants. *J Trop Pediatr* 2012;58(6):475-80. doi: https://dx.doi.org/10.1093/tropej/fms021
12. Mehta S, Finkelstein JL, Venkatramanan S, et al. Effect of iron and zinc-biofortified pearl millet consumption on growth and immune competence in children aged 12-18 months in India: study protocol for a randomised controlled trial. *BMJ Open* 2017;7(11):e017631. doi: https://dx.doi.org/10.1136/bmjopen-2017-017631
13. Huey SL, Venkatramanan S, Udipi SA, et al. Acceptability of Iron- and Zinc-Biofortified Pearl Millet (ICTP-8203)-Based Complementary Foods among Children in an Urban Slum of Mumbai, India (vol 4, 39, 2017). *Front Nutr* 2018;5:2. doi: 10.3389/fnut.2018.00092
14. Quintero MDC, Hernandez LO, Villasana AC, et al. IMPACT OF CONSUMPTION FO CORN FLOUR WITH LOW LEVEL ENRICHMENT IN CHILDREN OF RURAL ZONES. *Nutr Hosp* 2011;26(5):1097-104. doi: 10.3305/nh.2011.26.5.5127
15. Bagni UV, Baiao MR, Santos MM, et al. [Effect of weekly rice fortification with iron on anemia prevalence and hemoglobin concentration among children attending public daycare centers in Rio de Janeiro, Brazil]. *Cad Saude Publica* 2009;25(2):291-302.
16. NCT00727545. Effect of Rice Fortification With Iron on Anemia Among Children. https://clinicaltrials.gov/ct2/show/NCT00727545.
17. Nesamvuni AE, Vorster HH, Margetts BM, et al. Fortification of maize meal improved the nutritional status of 1-3-year-old African children. *Public Health Nutrition* 2005;8(5):461-67. doi: 10.1079/phn2005782
18. Faber M, Kvalsvig JD, Lombard CJ, et al. Effect of a fortified maize-meal porridge on anemia, micronutrient status, and motor development of infants. *Am J Clin Nutr* 2005;82(5):1032-9.
19. Schumann K, Romero-Abal ME, Maurer A, et al. Haematological response to haem iron or ferrous sulphate mixed with refried black beans in moderately anaemic Guatemalan pre-school children. *Public Health Nutr* 2005;8(6):572-81.
20. Lartey A, Manu A, Brown KH, et al. Predictors of micronutrient status among six- to twelve-month-old breast-fed Ghanaian infants. *J Nutr* 2000;130(2):199-207.
21. Lartey A, Manu A, Brown KH, et al. A randomized, community-based trial of the effects of improved, centrally processed complementary foods on growth and micronutrients status of Ghanaian infants from 6 to 12 mo of age Weanimix, a cereal-legume blend. *American Journal of Clinical Nutrition* 1999;70(3):391-404. doi: 10.1093/ajcn/70.3.391
22. Bovell-Benjamin AC, Allen LH, Guinard JX. Toddlers' acceptance of whole maize meal porridge fortified with Ferrous Bisglycinate. *Food Qual Prefer* 1999;10(2):123-28. doi: 10.1016/s0950-3293(98)00058-5

23. Liu DS, Bates CJ, Yin TA, et al. Nutritional efficacy of a fortified weaning rusk in a rural area near Beijing. *Am J Clin Nutr* 1993;57(4):506-11. doi: 10.1093/ajcn/57.4.50610.1093/ajcn/57.4.506.
24. Gershoff SN, McGandy RB, Suttapreyasri D, et al. Nutrition studied in Thailand. II. Effects of fortification of rice with lysine, threonine, thiamin, riboflavin, vitamin A, and iron on preschool children. *Am J Clin Nutr* 1977;30(7):1185-95.
25. Gershoff SN, McGandy RB, Suttapreyasri D, et al. Amino acid fortification of rice studies in Thailand. I. Background and baseline data. *The American journal of clinical nutrition* 1975;28(2):170-82. doi: 10.1093/ajcn/28.2.170

## Supplement 7. Risk of bias assessments

**Risk of bias graph: review authors' judgements about each risk of bias item presented as percentages across all included trials.**

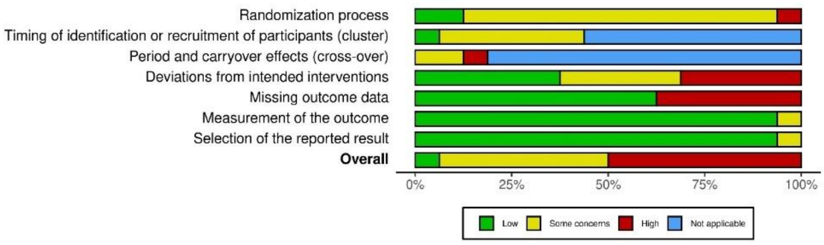


**Risk of bias summary: review authors' judgements about each risk of bias item for each included trial**

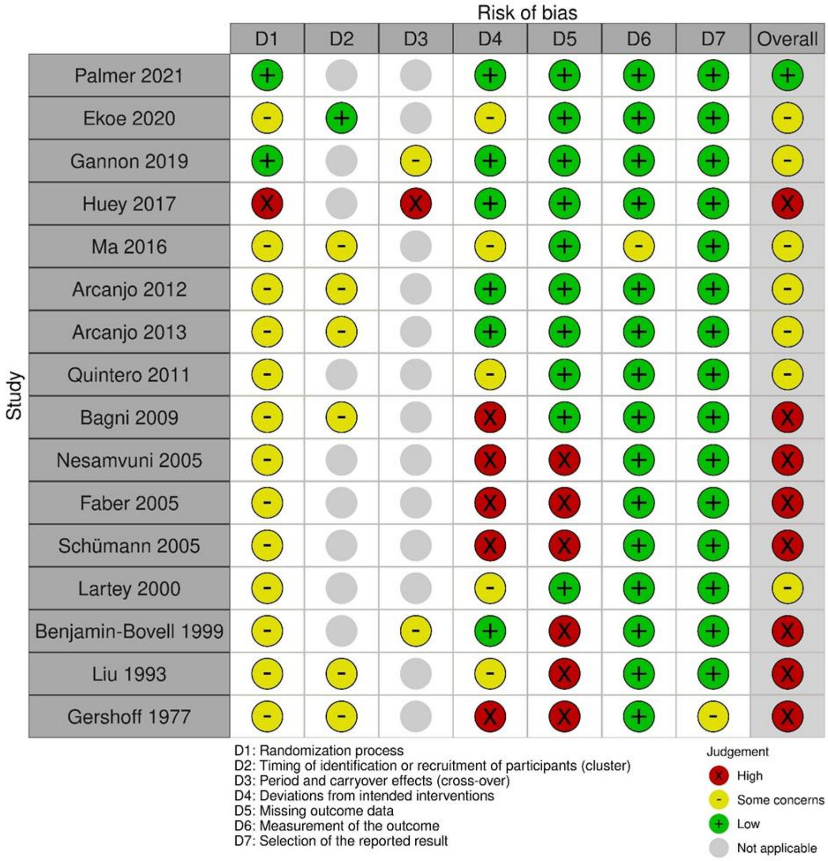

## Supplement 8. GRADE Assessment

| Certainty assessment | | | | | | | № of patients | | Effect | | Certainty | Importance |
|---|---|---|---|---|---|---|---|---|---|---|---|---|
| № of studies | Study design | Risk of bias | Inconsistency | Indirectness | Imprecision | Other considerations | Fortified complementary food | Non-fortified complementary food | Relative (95% CI) | Absolute (95% CI) | | |
| **Anaemia (follow-up: 3 to 12 months)** | | | | | | | | | | | | |
| 6[1,2,3,4,5,6] | randomised trials | serious[a] | not serious | not serious | not serious[b] | none | 51/617 (8.3%) | 90/588 (15.3%) | **RR 0.57** (0.39 to 0.82) | **66 fewer per 1 000** (from 93 fewer to 28 fewer) | ⊕⊕⊕◯ Moderate | CRITICAL |
| **Haemoglobin (follow-up: 3 to 12 months; assessed with: g/L)** | | | | | | | | | | | | |
| 11[1,2,3,4,6,7,8,9,10,11,12] | randomised trials | serious[c] | not serious[d] | not serious | not serious[e] | none | 1110 | 1065 | - | MD **3.43 g/L higher** (1.34 higher to 5.52 higher) | ⊕⊕⊕◯ Moderate | CRITICAL |
| **Weight-for-age (follow-up: 6 to 12 months; assessed with: z-scores)** | | | | | | | | | | | | |
| 5[1,4,8,11,12] | randomised trials | serious[f] | not serious | not serious | not serious[e] | none | 607 | 599 | - | MD **0.01 z-score lower** (0.07 lower to 0.06 higher) | ⊕⊕⊕◯ Moderate | CRITICAL |
| **Weight-for-length (follow-up: 6 to 12 months; assessed with: z-scores)** | | | | | | | | | | | | |

| Certainty assessment | | | | | | | № of patients | | Effect | | Certainty | Importance |
|---|---|---|---|---|---|---|---|---|---|---|---|---|
| № of studies | Study design | Risk of bias | Inconsistency | Indirectness | Imprecision | Other considerations | Fortified complementary food | Non-fortified complementary food | Relative (95% CI) | Absolute (95% CI) | | |
| 4[1,4,8,12] | randomised trials | serious[g] | not serious | not serious | not serious[e] | none | 560 | 549 | - | MD **0.05 z-score lower** (0.19 lower to 0.1 higher) | ⊕⊕⊕◯ Moderate | CRITICAL |
| **Length-for-age (follow-up: 6 to 12 months; assessed with: z-scores)** | | | | | | | | | | | | |
| 4[1,4,5,12] | randomised trials | serious[g] | serious[h] | not serious | not serious[e] | none | 412 | 399 | - | MD **0.01 z-score lower** (0.21 lower to 0.2 higher) | ⊕⊕◯◯ Low | CRITICAL |
| **Iron status (follow-up: 3 to 12 months; assessed with: ferritin concentrations in ug/L)** | | | | | | | | | | | | |
| 6[1,4,5,7,10,12] | randomised trials | serious[i] | serious[j] | not serious | not serious[e] | none | 464 | 439 | - | MD **0.43 ug/L higher** (0.14 higher to 0.72 higher) | ⊕⊕◯◯ Low | CRITICAL |
| **Iron status (follow-up: 12 months; assessed with: body iron in mg/kg)** | | | | | | | | | | | | |

| Certainty assessment | | | | | | | № of patients | | Effect | | Certainty | Importance |
|---|---|---|---|---|---|---|---|---|---|---|---|---|
| № of studies | Study design | Risk of bias | Inconsistency | Indirectness | Imprecision | Other considerations | Fortified complementary food | Non-fortified complementary food | Relative (95% CI) | Absolute (95% CI) | | |
| 1[4] | randomised trials | serious[k] | not serious[l] | not serious | serious[m] | none | 97 | 104 | - | MD **1.47 mg/kg higher** (0.63 higher to 2.31 higher) | ⊕⊕◯◯ Low | CRITICAL |
| **Iron status (follow-up: 3 months; assessed with: free erythrocythe porphyrin in µg/L)** | | | | | | | | | | | | |
| 1[10] | randomised trials | very serious[n] | not serious[l] | not serious | serious[m] | none | 69 | 78 | - | MD **30 higher** (26.06 lower to 86.06 higher) | ⊕◯◯◯ Very low | CRITICAL |
| **Serum retinol (follow-up: 3 to 12 months; assessed with: µmol/L)** | | | | | | | | | | | | |
| 5[5,9,10,12,13] | randomised trials | serious[o] | not serious | not serious | not serious[e] | none | 225 | 250 | - | MD **0.03 umol/L higher** (0.02 lower to 0.08 higher) | ⊕⊕⊕◯ Moderate | CRITICAL |
| **Serum zinc (follow-up: 6 months; assessed with: g/dL)** | | | | | | | | | | | | |

| Certainty assessment | | | | | | | № of patients | | Effect | | Certainty | Importance |
|---|---|---|---|---|---|---|---|---|---|---|---|---|
| № of studies | Study design | Risk of bias | Inconsistency | Indirectness | Imprecision | Other considerations | Fortified complementary food | Non-fortified complementary food | Relative (95% CI) | Absolute (95% CI) | | |
| 2[5,12] | randomised trials | serious[p] | not serious | not serious | serious[m] | none | 170 | 163 | - | MD **0.13 lower** (0.82 lower to 0.56 higher) | ⊕⊕◯◯ Low | CRITICAL |
| **Diarrhoea (follow-up: 6 months; assessed with: number of new episoded/ 100 days at risk)** | | | | | | | | | | | | |
| 1[5] | randomised trials | serious[k] | not serious[l] | not serious | very serious[q] | none | 47 | 50 | - | MD **0.6 higher** (2.16 lower to 3.36 higher) | ⊕◯◯◯ Very low | IMPORTANT |
| **Acute respiratory tract diseases (follow-up: 6 months; assessed with: number of new episoded/ 100 days at risk)** | | | | | | | | | | | | |
| 1[5] | randomised trials | serious[k] | not serious[l] | not serious | very serious[q] | none | 47 | 50 | - | MD **0.3 higher** (0.38 lower to 0.98 higher) | ⊕◯◯◯ Very low | IMPORTANT |
| **Fever diseases (follow-up: 6 months; assessed with: number of new episoded/ 100 days at risk)** | | | | | | | | | | | | |
| 1[5] | randomised trials | serious[k] | not serious[l] | not serious | very serious[q] | none | 47 | 50 | - | MD **0.1 higher** (1.21 lower to 1.41 higher) | ⊕◯◯◯ Very low | IMPORTANT |
| **Mental skill development (follow-up: 10 to 12 months; assessed with: BSID I-III)** | | | | | | | | | | | | |

| Certainty assessment | | | | | | | № of patients | | Effect | | Certainty | Importance |
|---|---|---|---|---|---|---|---|---|---|---|---|---|
| № of studies | Study design | Risk of bias | Inconsistency | Indirectness | Imprecision | Other considerations | Fortified complementary food | Non-fortified complementary food | Relative (95% CI) | Absolute (95% CI) | | |
| 2[4,8] | randomised trials | serious[r] | not serious | not serious | not serious[e] | none | 250 | 258 | - | MD **0.8 higher** (0.12 higher to 1.48 higher) | ⊕⊕⊕◯ Moderate | IMPORTANT |
| **Fine motor score (follow-up: 12 months; assessed with: BSID III)** | | | | | | | | | | | | |
| 1[4] | randomised trials | serious[k] | not serious[l] | not serious | serious[m] | none | 55 | 58 | - | MD **0** (0.4 lower to 0.4 higher) | ⊕⊕◯◯ Low | IMPORTANT |
| **Gross motor score (follow-up: 12 months; assessed with: BSID III)** | | | | | | | | | | | | |
| 1[4] | randomised trials | serious[k] | not serious[l] | not serious | serious[m] | none | 55 | 58 | - | MD **0.2 lower** (0.62 lower to 0.22 higher) | ⊕⊕◯◯ Low | IMPORTANT |
| **Psychomotor development (follow-up: 6 to 10 months; assessed with: BSID I-III)** | | | | | | | | | | | | |
| 2[8,12] | randomised trials | serious[p] | not serious | not serious | not serious[e] | none | 323 | 338 | - | MD **1.13 higher** (0.35 higher to 1.91 higher) | ⊕⊕⊕◯ Moderate | IMPORTANT |

| Certainty assessment | | | | | | | № of patients | | Effect | | Certainty | Importance |
|---|---|---|---|---|---|---|---|---|---|---|---|---|
| № of studies | Study design | Risk of bias | Inconsistency | Indirectness | Imprecision | Other considerations | Fortified complementary food | Non-fortified complementary food | Relative (95% CI) | Absolute (95% CI) | | |
| **Acceptability (follow-up: 3 days; assessed with: 9-point hedonic scale)** | | | | | | | | | | | | |
| 3[14,15,16] | randomised + non-randomised trials | very serious[s] | not serious | not serious | serious[m] | none | Acceptability of fortified as compared to unfortified complementary food was measured in three acute studies with a total of 215 children. All described that there were no significant differences between the ratings of children allocated to the two groups | | | | ⊕◯◯◯ Very low | IMPORTANT |
| **Iron deficiency (follow-up: 6 to 12 months)** | | | | | | | | | | | | |
| 3[1,4,5] | randomised trials | serious[t] | not serious | not serious | not serious[u] | none | 48/274 (17.5%) | 120/297 (40.4%) | **RR 0.39** (0.21 to 0.75) | **246 fewer per 1 000** (from 319 fewer to 101 fewer) | ⊕⊕⊕◯ Moderate | CRITICAL |
| **Vitamin A deficiency (follow-up: 3 to 12 months)** | | | | | | | | | | | | |
| 3[5,9,13] | randomised trials | very serious[v] | serious[w] | not serious | serious[x] | none | 39/148 (26.4%) | 30/109 (27.5%) | **RR 0.97** (0.24 to 3.90) | **8 fewer per 1 000** (from 209 fewer to 798 more) | ⊕◯◯◯ Very low | CRITICAL |
| **Zinc deficiency (follow-up: 6 months)** | | | | | | | | | | | | |

| Certainty assessment | | | | | | | № of patients | | Effect | | Certainty | Importance |
|---|---|---|---|---|---|---|---|---|---|---|---|---|
| № of studies | Study design | Risk of bias | Inconsistency | Indirectness | Imprecision | Other considerations | Fortified complementary food | Non-fortified complementary food | Relative (95% CI) | Absolute (95% CI) | | |
| 1[5] | randomised trials | serious[y] | not serious[l] | not serious | very serious[z] | none | 3/30 (10.0%) | 1/31 (3.2%) | **RR 3.10** (0.34 to 28.17) | **68 more per 1 000** (from 21 fewer to 876 more) | ⨁◯◯◯ Very low | CRITICAL |

**CI:** confidence interval; **MD:** mean difference; **RR:** risk ratio

***Explanations***

a. Downgraded by one level for risk of bias (RoB) since 1 out of 6 studies was rated with a high RoB, and none of the included studies was rated with a low RoB.

b. Not downgraded for imprecision. Although the number of events was low (<400), the outcome was a common event (occurred >1/100), and there were 6 studies with a median sample size of 170 children included. The 95% confidence interval (CI) for the pooled estimate is narrow and is consistent with benefit.

c. Downgraded by one level for RoB since 5 out of 11 studies were rated with a high RoB, and none of the included studies was rated with a low RoB.

d. Not downgraded for inconsistency although $I^2$ was 55% (driven by the study of Faber et al. 2005), since 95% CI overlaps mainly between studies. In all sub-group analyses heterogeneity was present only in those sub-groups which contained the study Faber et al. 2005, while no heterogeneity was observed in other sub-groups.

e. Not downgraded for imprecision since number of participants was >400.

f. Downgraded by one level for RoB since 1 out of 5 studies were rated with a high RoB, and none of the included studies was rated with a low RoB.

g. Downgraded by one level for RoB since 1 out of 4 studies were rated with a high RoB, and none was rated as low RoB.

h. Downgraded by one level for inconsistency since $I^2$ was 68%, p-value for heterogeneity was 0.02, point estimates and 95% CI did not overlap between studies. Sub-group analyses did not fully explain heterogeneity.

i. Downgraded by one level for RoB since 3 out of 6 studies were rated with a high RoB, and none was rated as low RoB.

j. Downgraded by one level for inconsistency since $I^2$ was 86%, p-value for heterogeneity was <0.001, point estimates and 95% CI did not overlap between studies. Sub-group analyses did not fully explain heterogeneity.

k. Downgraded by one level for RoB since the included study was rated with some concerns of RoB.

l. This is a single study so inconsistency cannot be judged.

m. Downgraded by one level for imprecision since total sample size was low (<400).

n. Downgraded by two levels for RoB since the included study was rated with a high RoB.

o. Downgraded by one level for RoB since 3 out of 5 studies were rated with a high RoB, and 1 study was rated as low RoB.

p. Downgraded by one level since 1 out of 2 included studies was rated with a high RoB, and none of the included studies was rated with a low RoB.

q. Downgraded by two levels for imprecision since sample size was very low (<100).

r. Downgraded by one level for RoB since both included studies were rated with some concerns for RoB.

s. Downgraded by two levels for RoB since 2 out of 3 included studies was rated with high RoB, and none of the included studies was rated with a low RoB.

t. Downgraded by one level for RoB since all included studies were rated with some concerns for RoB.

u. Not downgraded for imprecision. Although the number of included studies is low (n=3), studies had an intermediate sample size with a median of 239 participants, and the outcome was a common event (occurred >1/100).

v. Downgraded by two levels for RoB, as for this outcome 1 out of the 3 included studies was rated with high RoB, and none of the included studies was rated with a low RoB. There were large baseline between-group differences in the number of vitamin A deficient participants in two studies (Nesamvuni 2005: 7 out of 16 in the experimental and 0 out of 20 in the control group; Palmer 2021: 10 out of 51 in the biofortified, 11 out 52 in the fortified and 18 out of 58 in the control group)

w. Downgraded by one level for inconsistency as point estimates did vary widely, 95% CI did not overlap between studies, the direction of effect was not consistent. and the magnitude of heterogeneity was high ($I^2$ was 74%, p-value for heterogeneity was 0.02). Due to the low number of studies subgroup analyses were not possible.

x. Downgraded by one level for imprecision since total sample size was low (<400).

y. Downgraded by one level for RoB since the included study was rated with some concerns of RoB.

z. Downgraded by two levels for imprecision since results are derived from one study, where total sample size was very low (n<100).

**References**

1.Ekoe, T., Bianpambe, O. I., Nguefack, F., Pondi, D. M., Kana-Sop, M. M., Hays, N. P., Medoua, G., Koki, P. N. Efficacy of an iron-fortified infant cereal to reduce the risk of iron deficiency anemia in young children in East Cameroon. Food sci; 2020.

2.Nogueira Arcanjo, F. P., Roberto Santos, P., Madeiro Leite, A. J., Bastos Mota, F. S., Duarte Segall, S. Rice fortified with iron given weekly increases hemoglobin levels and reduces anemia in infants: a community intervention trial. Int J Vitam Nutr Res; 2013.

3.Nogueira Arcanjo, F. P., Santos, P. R., Arcanjo, C. P., Amancio, O. M., Braga, J. A. Use of iron-fortified rice reduces anemia in infants. J Trop Pediatr; 2012.

4.Ma, J., Sun, Q., Liu, J., Hu, Y., Liu, S., Zhang, J., Sheng, X., Hambidge, K. M. The Effect of Iron Fortification on Iron (Fe) Status and Inflammation: A Randomized Controlled Trial. PLoS ONE; 2016.

5.Lartey, A., Manu, A., Brown, K. H., Peerson, J. M., Dewey, K. G. A randomized, community-based trial of the effects of improved, centrally processed complementary foods on growth and micronutrients status of Ghanaian infants from 6 to 12 mo of age Weanimix, a cereal-legume blend. American Journal of Clinical Nutrition; 1999.

6.Bagni, U. V., Baiao, M. R., Santos, M. M., Luiz, R. R., Veiga, G. V. [Effect of weekly rice fortification with iron on anemia prevalence and hemoglobin concentration among children attending public daycare centers in Rio de Janeiro, Brazil]. Cad Saude Publica; 2009.

7.Schumann, K., Romero-Abal, M. E., Maurer, A., Luck, T., Beard, J., Murray-Kolb, L., Bulux, J., Mena, I., Solomons, N. W. Haematological response to haem iron or ferrous sulphate mixed with refried black beans in moderately anaemic Guatemalan pre-school children. Public Health Nutr; 2005.

8.Quintero, M. D. C., Hernandez, L. O., Villasana, A. C., Amaro, J. A. R., Soto, N. G., Arenas, J. A., Solano, J. A. L. IMPACT OF CONSUMPTION FO CORN FLOUR WITH LOW LEVEL ENRICHMENT IN CHILDREN OF RURAL ZONES. Nutr. Hosp.; 2011.

9.Nesamvuni, A. E., Vorster, H. H., Margetts, B. M., Kruger, A., Nesamvuni, Alufheli E., Vorster, Hester H., Margetts, Barrie M., Kruger, Annamarie. Fortification of maize meal improved the nutritional status of 1-3-year-old African children. Public Health Nutrition; 2005.

10.Liu, D. S., Bates, C. J., Yin, T. A., Wang, X. B., Lu, C. Q. Nutritional efficacy of a fortified weaning rusk in a rural area near Beijing. Am J Clin Nutr; 1993.

11.Lartey, A., Manu, A., Brown, K. H., Dewey, K. G. Predictors of micronutrient status among six- to twelve-month-old breast-fed Ghanaian infants. J Nutr; 2000.

12.Faber, M., Kvalsvig, J. D., Lombard, C. J., Benade, A. J. Effect of a fortified maize-meal porridge on anemia, micronutrient status, and motor development of infants. Am J Clin Nutr; 2005.

13.Palmer, A. C., Jobarteh, M. L., Chipili, M., Greene, M. D., Oxley, A., Lietz, G., Mwanza, R., Haskell, M. J. Biofortified and fortified maize consumption reduces prevalence of low milk retinol, but does not increase vitamin A stores of breastfeeding Zambian infants with adequate reserves: a randomized controlled trial. Am J Clin Nutr; 2021.

14.Huey, S. L., Venkatramanan, S., Udipi, S. A., Finkelstein, J. L., Ghugre, P., Haas, J. D., Thakker, V., Thorat, A., Salvi, A., Kurpad, A. V., Mehta, S. Acceptability of Iron- and Zinc-Biofortified Pearl Millet (ICTP-8203)-Based Complementary Foods among Children in an Urban Slum of Mumbai, India (vol 4, 39, 2017). Front. Nutr.; 2018.

15.Gannon, B. M., Thakker, V., Bonam, V. S., Haas, J. D., Bonam, W., Finkelstein, J. L., Udipi, S. A., Mehta, S. A Randomized Crossover Study to Evaluate Recipe Acceptability in Breastfeeding Mothers and Young Children in India Targeted for a Multiple Biofortified Food Crop Intervention. Food Nutr Bull; 2019.

16.Bovell-Benjamin, A. C., Allen, L. H., Guinard, J. X. Toddlers' acceptance of whole maize meal porridge fortified with Ferrous Bisglycinate. Food. Qual. Prefer.; 1999.

# Supplement 9. ANALYSES

## Analysis 1.1 Fortified versus non-fortified complementary food. Outcome: Anaemia

*see Figure 2 in manuscript*

## Analysis 1.2 Fortified versus non-fortified complementary food. Outcome: Anaemia by age at the start of the intervention

| Study or Subgroup | log[Risk Ratio] | SE | Fortified Total | Non-fortified Total | Weight | Risk Ratio IV, Random, 95% CI |
|---|---|---|---|---|---|---|
| **1.2.1 6 to 11 months** | | | | | | |
| Lartey 2000 | -0.0967 | 0.2756 | 47 | 48 | 28.6% | 0.91 [0.53, 1.56] |
| Ma 2016 (C) | -0.8398 | 0.3742 | 140 | 133 | 19.1% | 0.43 [0.21, 0.90] |
| **Subtotal (95% CI)** | | | **187** | **181** | **47.7%** | **0.65 [0.32, 1.35]** |
| Heterogeneity: $Tau^2 = 0.17$; $Chi^2 = 2.56$, df = 1 (P = 0.11); $I^2 = 61\%$ | | | | | | |
| Test for overall effect: Z = 1.15 (P = 0.25) | | | | | | |
| **1.2.2 12 to 23 months** | | | | | | |
| Arcanjo 2012 (C) | -0.6179 | 0.5253 | 96 | 92 | 11.2% | 0.54 [0.19, 1.51] |
| Arcanjo 2013 (C) | -1.2068 | 0.6915 | 72 | 70 | 6.9% | 0.30 [0.08, 1.16] |
| **Subtotal (95% CI)** | | | **168** | **162** | **18.1%** | **0.43 [0.19, 0.99]** |
| Heterogeneity: $Tau^2 = 0.00$; $Chi^2 = 0.46$, df = 1 (P = 0.50); $I^2 = 0\%$ | | | | | | |
| Test for overall effect: Z = 1.99 (P = 0.05) | | | | | | |
| **1.2.3 Mean age >23 months** | | | | | | |
| Bagni 2009 (C) | -0.3567 | 0.3284 | 180 | 174 | 22.9% | 0.70 [0.37, 1.33] |
| Ekoe 2020 (C) | -1.2967 | 0.5217 | 82 | 71 | 11.3% | 0.27 [0.10, 0.76] |
| **Subtotal (95% CI)** | | | **262** | **245** | **34.2%** | **0.48 [0.19, 1.18]** |
| Heterogeneity: $Tau^2 = 0.25$; $Chi^2 = 2.33$, df = 1 (P = 0.13); $I^2 = 57\%$ | | | | | | |
| Test for overall effect: Z = 1.60 (P = 0.11) | | | | | | |
| **Total (95% CI)** | | | **617** | **588** | **100.0%** | **0.57 [0.39, 0.82]** |
| Heterogeneity: $Tau^2 = 0.05$; $Chi^2 = 6.57$, df = 5 (P = 0.25); $I^2 = 24\%$ | | | | | | |
| Test for overall effect: Z = 2.97 (P = 0.003) | | | | | | |
| Test for subgroup differences: $Chi^2 = 0.60$, df = 2 (P = 0.74), $I^2 = 0\%$ | | | | | | |

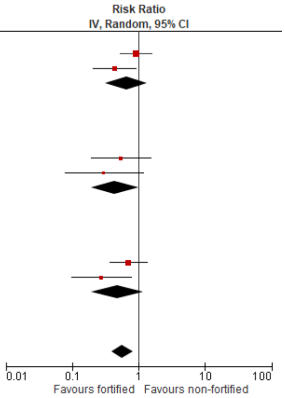


## Analysis 1.3 Fortified versus non-fortified complementary food. Outcome: Anaemia by types of nutrients added through fortification

| Study or Subgroup | log[Risk Ratio] | SE | Fortified Total | Non-fortified Total | Weight | Risk Ratio IV, Random, 95% CI |
|---|---|---|---|---|---|---|
| **1.3.1 Iron** | | | | | | |
| Arcanjo 2012 (C) | -0.6179 | 0.5253 | 96 | 92 | 11.2% | 0.54 [0.19, 1.51] |
| Arcanjo 2013 (C) | -1.2068 | 0.6915 | 72 | 70 | 6.9% | 0.30 [0.08, 1.16] |
| Bagni 2009 (C) | -0.3567 | 0.3284 | 180 | 174 | 22.9% | 0.70 [0.37, 1.33] |
| Ekoe 2020 (C) | -1.2967 | 0.5217 | 82 | 71 | 11.3% | 0.27 [0.10, 0.76] |
| **Subtotal (95% CI)** | | | **430** | **407** | **52.3%** | **0.50 [0.32, 0.79]** |
| Heterogeneity: $Tau^2 = 0.00$; $Chi^2 = 2.96$, df = 3 (P = 0.40); $I^2 = 0\%$ | | | | | | |
| Test for overall effect: Z = 2.97 (P = 0.003) | | | | | | |
| **1.3.2 Iron, zinc, vitamin B12** | | | | | | |
| Ma 2016 (C) | -0.8398 | 0.3742 | 140 | 133 | 19.1% | 0.43 [0.21, 0.90] |
| **Subtotal (95% CI)** | | | **140** | **133** | **19.1%** | **0.43 [0.21, 0.90]** |
| Heterogeneity: Not applicable | | | | | | |
| Test for overall effect: Z = 2.24 (P = 0.02) | | | | | | |
| **1.3.3 Multivitamins and minerals** | | | | | | |
| Lartey 2000 | -0.0967 | 0.2756 | 47 | 48 | 28.6% | 0.91 [0.53, 1.56] |
| **Subtotal (95% CI)** | | | **47** | **48** | **28.6%** | **0.91 [0.53, 1.56]** |
| Heterogeneity: Not applicable | | | | | | |
| Test for overall effect: Z = 0.35 (P = 0.73) | | | | | | |
| **Total (95% CI)** | | | **617** | **588** | **100.0%** | **0.57 [0.39, 0.82]** |
| Heterogeneity: $Tau^2 = 0.05$; $Chi^2 = 6.57$, df = 5 (P = 0.25); $I^2 = 24\%$ | | | | | | |
| Test for overall effect: Z = 2.97 (P = 0.003) | | | | | | |
| Test for subgroup differences: $Chi^2 = 3.61$, df = 2 (P = 0.16), $I^2 = 44.7\%$ | | | | | | |

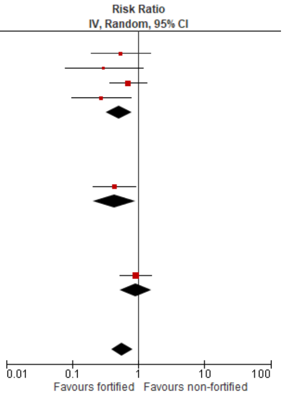

## Analysis 1.4 Fortified versus non-fortified complementary food. Outcome: Anaemia by types of products fortified

| Study or Subgroup | log[Risk Ratio] | SE | Fortified Total | Non-fortified Total | Weight | Risk Ratio IV, Random, 95% CI |
|---|---|---|---|---|---|---|
| **1.4.1 Wheat** | | | | | | |
| Ekoe 2020 (C) | -1.2967 | 0.5217 | 82 | 71 | 11.3% | 0.27 [0.10, 0.76] |
| **Subtotal (95% CI)** | | | **82** | **71** | **11.3%** | **0.27 [0.10, 0.76]** |
| Heterogeneity: Not applicable | | | | | | |
| Test for overall effect: Z = 2.49 (P = 0.01) | | | | | | |
| **1.4.2 Rice** | | | | | | |
| Arcanjo 2012 (C) | -0.6179 | 0.5253 | 96 | 92 | 11.2% | 0.54 [0.19, 1.51] |
| Arcanjo 2013 (C) | -1.2068 | 0.6915 | 72 | 70 | 6.9% | 0.30 [0.08, 1.16] |
| Bagni 2009 (C) | -0.3567 | 0.3284 | 180 | 174 | 22.9% | 0.70 [0.37, 1.33] |
| Ma 2016 (C) | -0.8398 | 0.3742 | 140 | 133 | 19.1% | 0.43 [0.21, 0.90] |
| **Subtotal (95% CI)** | | | **488** | **469** | **60.1%** | **0.53 [0.35, 0.80]** |
| Heterogeneity: Tau² = 0.00; Chi² = 1.70, df = 3 (P = 0.64); I² = 0% | | | | | | |
| Test for overall effect: Z = 2.99 (P = 0.003) | | | | | | |
| **1.4.3 Cereal-legume blend** | | | | | | |
| Lartey 2000 | -0.0967 | 0.2756 | 47 | 48 | 28.6% | 0.91 [0.53, 1.56] |
| **Subtotal (95% CI)** | | | **47** | **48** | **28.6%** | **0.91 [0.53, 1.56]** |
| Heterogeneity: Not applicable | | | | | | |
| Test for overall effect: Z = 0.35 (P = 0.73) | | | | | | |
| **Total (95% CI)** | | | **617** | **588** | **100.0%** | **0.57 [0.39, 0.82]** |
| Heterogeneity: Tau² = 0.05; Chi² = 6.57, df = 5 (P = 0.25); I² = 24% | | | | | | |
| Test for overall effect: Z = 2.97 (P = 0.003) | | | | | | |
| Test for subgroup differences: Chi² = 4.87, df = 2 (P = 0.09), I² = 58.9% | | | | | | |

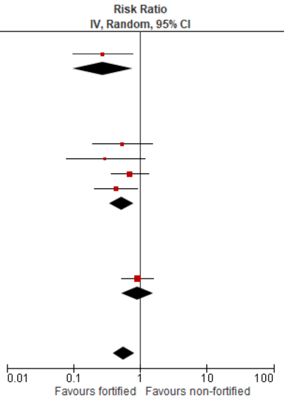


## Analysis 1.5 Fortified versus non-fortified complementary food. Outcome: Anaemia by duration of intervention

| Study or Subgroup | log[Risk Ratio] | SE | Fortified Total | Non-fortified Total | Weight | Risk Ratio IV, Random, 95% CI |
|---|---|---|---|---|---|---|
| **1.5.1 Less than 6 months** | | | | | | |
| Arcanjo 2012 (C) | -0.6179 | 0.5253 | 96 | 92 | 11.2% | 0.54 [0.19, 1.51] |
| Arcanjo 2013 (C) | -1.2068 | 0.6915 | 72 | 70 | 6.9% | 0.30 [0.08, 1.16] |
| **Subtotal (95% CI)** | | | **168** | **162** | **18.1%** | **0.43 [0.19, 0.99]** |
| Heterogeneity: Tau² = 0.00; Chi² = 0.46, df = 1 (P = 0.50); I² = 0% | | | | | | |
| Test for overall effect: Z = 1.99 (P = 0.05) | | | | | | |
| **1.5.2 Six months or more** | | | | | | |
| Bagni 2009 (C) | -0.3567 | 0.3284 | 180 | 174 | 22.9% | 0.70 [0.37, 1.33] |
| Ekoe 2020 (C) | -1.2967 | 0.5217 | 82 | 71 | 11.3% | 0.27 [0.10, 0.76] |
| Lartey 2000 | -0.0967 | 0.2756 | 47 | 48 | 28.6% | 0.91 [0.53, 1.56] |
| Ma 2016 (C) | -0.8398 | 0.3742 | 140 | 133 | 19.1% | 0.43 [0.21, 0.90] |
| **Subtotal (95% CI)** | | | **449** | **426** | **81.9%** | **0.59 [0.36, 0.94]** |
| Heterogeneity: Tau² = 0.10; Chi² = 5.44, df = 3 (P = 0.14); I² = 45% | | | | | | |
| Test for overall effect: Z = 2.21 (P = 0.03) | | | | | | |
| **Total (95% CI)** | | | **617** | **588** | **100.0%** | **0.57 [0.39, 0.82]** |
| Heterogeneity: Tau² = 0.05; Chi² = 6.57, df = 5 (P = 0.25); I² = 24% | | | | | | |
| Test for overall effect: Z = 2.97 (P = 0.003) | | | | | | |
| Test for subgroup differences: Chi² = 0.38, df = 1 (P = 0.54), I² = 0% | | | | | | |

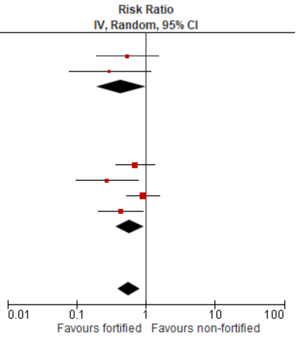


## Analysis 1.6 Fortified versus non-fortified complementary food. Outcome: Anaemia by baseline anaemia status

| Study or Subgroup | log[Risk Ratio] | SE | Fortified Total | Non-fortified Total | Weight | Risk Ratio IV, Random, 95% CI |
|---|---|---|---|---|---|---|
| **1.6.1 Anaemic** | | | | | | |
| Ekoe 2020 (C) | -1.2967 | 0.5217 | 82 | 71 | 11.3% | 0.27 [0.10, 0.76] |
| **Subtotal (95% CI)** | | | **82** | **71** | **11.3%** | **0.27 [0.10, 0.76]** |
| Heterogeneity: Not applicable | | | | | | |
| Test for overall effect: Z = 2.49 (P = 0.01) | | | | | | |
| **1.6.2 Non-anaemic** | | | | | | |
| **Subtotal (95% CI)** | | | **0** | **0** | | **Not estimable** |
| Heterogeneity: Not applicable | | | | | | |
| Test for overall effect: Not applicable | | | | | | |
| **1.6.3 Mixed** | | | | | | |
| Arcanjo 2012 (C) | -0.6179 | 0.5253 | 96 | 92 | 11.2% | 0.54 [0.19, 1.51] |
| Arcanjo 2013 (C) | -1.2068 | 0.6915 | 72 | 70 | 6.9% | 0.30 [0.08, 1.16] |
| Bagni 2009 (C) | -0.3567 | 0.3284 | 180 | 174 | 22.9% | 0.70 [0.37, 1.33] |
| Lartey 2000 | -0.0967 | 0.2756 | 47 | 48 | 28.6% | 0.91 [0.53, 1.56] |
| Ma 2016 (C) | -0.8398 | 0.3742 | 140 | 133 | 19.1% | 0.43 [0.21, 0.90] |
| **Subtotal (95% CI)** | | | **535** | **517** | **88.7%** | **0.64 [0.46, 0.90]** |
| Heterogeneity: Tau² = 0.00; Chi² = 4.10, df = 4 (P = 0.39); I² = 2% | | | | | | |
| Test for overall effect: Z = 2.56 (P = 0.01) | | | | | | |
| **Total (95% CI)** | | | **617** | **588** | **100.0%** | **0.57 [0.39, 0.82]** |
| Heterogeneity: Tau² = 0.05; Chi² = 6.57, df = 5 (P = 0.25); I² = 24% | | | | | | |
| Test for overall effect: Z = 2.97 (P = 0.003) | | | | | | |
| Test for subgroup differences: Chi² = 2.44, df = 1 (P = 0.12), I² = 59.0% | | | | | | |

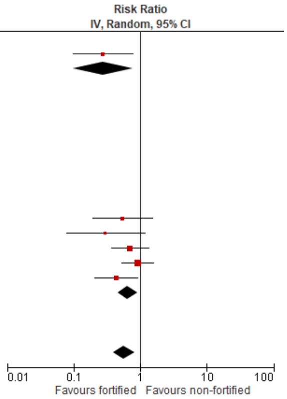

## Analysis 1.7 Fortified versus non-fortified complementary food. Outcome: Anaemia by country income classification

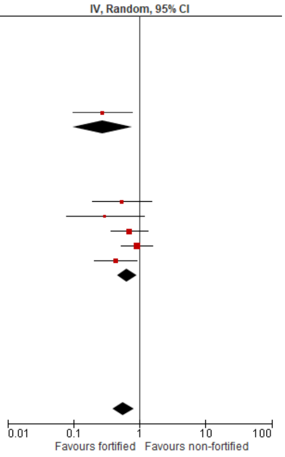

| Study or Subgroup | log[Risk Ratio] | SE | Fortified Total | Non-fortified Total | Weight | Risk Ratio IV, Random, 95% CI |
|---|---|---|---|---|---|---|
| **1.7.1 High income** | | | | | | |
| **Subtotal (95% CI)** | | | **0** | **0** | | **Not estimable** |
| Heterogeneity: Not applicable | | | | | | |
| Test for overall effect: Not applicable | | | | | | |
| **1.7.2 Upper middle income** | | | | | | |
| Ekoe 2020 (C) | -1.2967 | 0.5217 | 82 | 71 | 11.3% | 0.27 [0.10, 0.76] |
| **Subtotal (95% CI)** | | | **82** | **71** | **11.3%** | **0.27 [0.10, 0.76]** |
| Heterogeneity: Not applicable | | | | | | |
| Test for overall effect: Z = 2.49 (P = 0.01) | | | | | | |
| **1.7.3 Lower middle income** | | | | | | |
| Arcanjo 2012 (C) | -0.6179 | 0.5253 | 96 | 92 | 11.2% | 0.54 [0.19, 1.51] |
| Arcanjo 2013 (C) | -1.2068 | 0.6915 | 72 | 70 | 6.9% | 0.30 [0.08, 1.16] |
| Bagni 2009 (C) | -0.3567 | 0.3284 | 180 | 174 | 22.9% | 0.70 [0.37, 1.33] |
| Lartey 2000 | -0.0967 | 0.2756 | 47 | 48 | 28.6% | 0.91 [0.53, 1.56] |
| Ma 2016 (C) | -0.8398 | 0.3742 | 140 | 133 | 19.1% | 0.43 [0.21, 0.90] |
| **Subtotal (95% CI)** | | | **535** | **517** | **88.7%** | **0.64 [0.46, 0.90]** |
| Heterogeneity: Tau² = 0.00; Chi² = 4.10, df = 4 (P = 0.39); I² = 2% | | | | | | |
| Test for overall effect: Z = 2.56 (P = 0.01) | | | | | | |
| **1.7.4 Low income** | | | | | | |
| **Subtotal (95% CI)** | | | **0** | **0** | | **Not estimable** |
| Heterogeneity: Not applicable | | | | | | |
| Test for overall effect: Not applicable | | | | | | |
| **Total (95% CI)** | | | **617** | **588** | **100.0%** | **0.57 [0.39, 0.82]** |
| Heterogeneity: Tau² = 0.05; Chi² = 6.57, df = 5 (P = 0.25); I² = 24% | | | | | | |
| Test for overall effect: Z = 2.97 (P = 0.003) | | | | | | |
| Test for subgroup differences: Chi² = 2.44, df = 1 (P = 0.12), I² = 59.0% | | | | | | |



## Analysis 1.8 Fortified versus non-fortified complementary food. Outcome: Anaemia by study funding

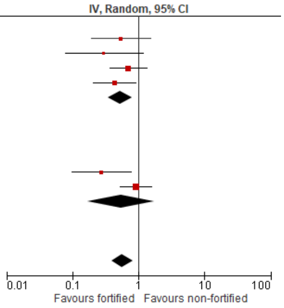

| Study or Subgroup | log[Risk Ratio] | SE | Fortified Total | Non-fortified Total | Weight | Risk Ratio IV, Random, 95% CI |
|---|---|---|---|---|---|---|
| **1.8.1 Non-commercial** | | | | | | |
| Arcanjo 2012 (C) | -0.6179 | 0.5253 | 96 | 92 | 11.2% | 0.54 [0.19, 1.51] |
| Arcanjo 2013 (C) | -1.2068 | 0.6915 | 72 | 70 | 6.9% | 0.30 [0.08, 1.16] |
| Bagni 2009 (C) | -0.3567 | 0.3284 | 180 | 174 | 22.9% | 0.70 [0.37, 1.33] |
| Ma 2016 (C) | -0.8398 | 0.3742 | 140 | 133 | 19.1% | 0.43 [0.21, 0.90] |
| **Subtotal (95% CI)** | | | **488** | **469** | **60.1%** | **0.53 [0.35, 0.80]** |
| Heterogeneity: Tau² = 0.00; Chi² = 1.70, df = 3 (P = 0.64); I² = 0% | | | | | | |
| Test for overall effect: Z = 2.99 (P = 0.003) | | | | | | |
| **1.8.2 Commercial (partly or fully)** | | | | | | |
| Ekoe 2020 (C) | -1.2967 | 0.5217 | 82 | 71 | 11.3% | 0.27 [0.10, 0.76] |
| Lartey 2000 | -0.0967 | 0.2756 | 47 | 48 | 28.6% | 0.91 [0.53, 1.56] |
| **Subtotal (95% CI)** | | | **129** | **119** | **39.9%** | **0.54 [0.17, 1.73]** |
| Heterogeneity: Tau² = 0.55; Chi² = 4.14, df = 1 (P = 0.04); I² = 76% | | | | | | |
| Test for overall effect: Z = 1.03 (P = 0.30) | | | | | | |
| **Total (95% CI)** | | | **617** | **588** | **100.0%** | **0.57 [0.39, 0.82]** |
| Heterogeneity: Tau² = 0.05; Chi² = 6.57, df = 5 (P = 0.25); I² = 24% | | | | | | |
| Test for overall effect: Z = 2.97 (P = 0.003) | | | | | | |
| Test for subgroup differences: Chi² = 0.00, df = 1 (P = 0.97), I² = 0% | | | | | | |



## 1.9 Fortified versus non-fortified complementary food. Outcome: Haemoglobin (g/L)

*see Figure 3 in manuscript*

## 1.10 Fortified versus non-fortified complementary food. Outcome: Haemoglobin by age at the start of the intervention

| Study or Subgroup | Mean Difference | SE | Fortified Total | Non-fortified Total | Weight | Mean Difference IV, Random, 95% CI |
|---|---|---|---|---|---|---|
| **1.10.1 6 to 11 months** | | | | | | |
| Faber 2005 | 9 | 1.4902 | 144 | 142 | 15.0% | 9.00 [6.08, 11.92] |
| Lartey 2000 | 4 | 4.3191 | 47 | 48 | 4.8% | 4.00 [-4.47, 12.47] |
| Liu 1993 (C) | 2.1 | 1.6469 | 77 | 85 | 14.1% | 2.10 [-1.13, 5.33] |
| Ma 2016 (C) | 2.1 | 1.4917 | 140 | 133 | 14.9% | 2.10 [-0.82, 5.02] |
| **Subtotal (95% CI)** | | | **408** | **408** | **48.7%** | **4.37 [0.39, 8.35]** |
| Heterogeneity: Tau² = 11.90; Chi² = 13.84, df = 3 (P = 0.003); I² = 78% | | | | | | |
| Test for overall effect: Z = 2.15 (P = 0.03) | | | | | | |
| **1.10.2 12 to 23 months** | | | | | | |
| Arcanjo 2012 (C) | 3.1 | 4.9266 | 96 | 92 | 3.9% | 3.10 [-6.56, 12.76] |
| Arcanjo 2013 (C) | 5.9 | 5.0181 | 72 | 70 | 3.8% | 5.90 [-3.94, 15.74] |
| Nesamvuni 2005 | 9 | 7.6844 | 16 | 20 | 1.8% | 9.00 [-6.06, 24.06] |
| Quintero 2011 | 0.9 | 1.0466 | 195 | 200 | 17.5% | 0.90 [-1.15, 2.95] |
| Schumann 2005 | 2.48 | 2.5441 | 61 | 30 | 9.6% | 2.48 [-2.51, 7.47] |
| **Subtotal (95% CI)** | | | **440** | **412** | **36.6%** | **1.48 [-0.34, 3.29]** |
| Heterogeneity: Tau² = 0.00; Chi² = 2.30, df = 4 (P = 0.68); I² = 0% | | | | | | |
| Test for overall effect: Z = 1.59 (P = 0.11) | | | | | | |
| **1.10.3 Mean age > 23 months** | | | | | | |
| Bagni 2009 (C) | 2.4 | 2.3479 | 180 | 174 | 10.5% | 2.40 [-2.20, 7.00] |
| Ekoe 2020 (C) | 3.4 | 4.6812 | 82 | 71 | 4.2% | 3.40 [-5.77, 12.57] |
| **Subtotal (95% CI)** | | | **262** | **245** | **14.7%** | **2.60 [-1.51, 6.71]** |
| Heterogeneity: Tau² = 0.00; Chi² = 0.04, df = 1 (P = 0.85); I² = 0% | | | | | | |
| Test for overall effect: Z = 1.24 (P = 0.22) | | | | | | |
| **Total (95% CI)** | | | **1110** | **1065** | **100.0%** | **3.44 [1.33, 5.55]** |
| Heterogeneity: Tau² = 5.50; Chi² = 22.00, df = 10 (P = 0.02); I² = 55% | | | | | | |
| Test for overall effect: Z = 3.20 (P = 0.001) | | | | | | |
| Test for subgroup differences: Chi² = 1.76, df = 2 (P = 0.42), I² = 0% | | | | | | |

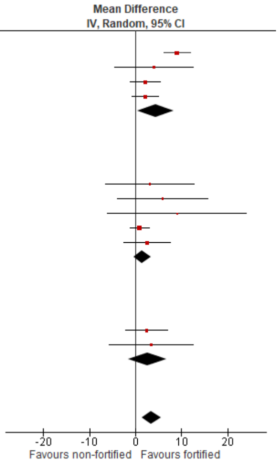


## 1.11 Fortified versus non-fortified complementary food. Outcome: Haemoglobin by types of nutrients added through fortification

| Study or Subgroup | Mean Difference | SE | Fortified Total | Non-fortified Total | Weight | Mean Difference IV, Random, 95% CI |
|---|---|---|---|---|---|---|
| **1.11.1 Iron** | | | | | | |
| Arcanjo 2012 (C) | 3.1 | 4.9266 | 96 | 92 | 3.9% | 3.10 [-6.56, 12.76] |
| Arcanjo 2013 (C) | 5.9 | 5.0181 | 72 | 70 | 3.8% | 5.90 [-3.94, 15.74] |
| Bagni 2009 (C) | 2.4 | 2.3479 | 180 | 174 | 10.5% | 2.40 [-2.20, 7.00] |
| Ekoe 2020 (C) | 3.4 | 4.6812 | 82 | 71 | 4.2% | 3.40 [-5.77, 12.57] |
| Schumann 2005 | 2.48 | 2.5441 | 61 | 30 | 9.6% | 2.48 [-2.51, 7.47] |
| **Subtotal (95% CI)** | | | **491** | **437** | **32.0%** | **2.89 [0.01, 5.77]** |
| Heterogeneity: Tau² = 0.00; Chi² = 0.44, df = 4 (P = 0.98); I² = 0% | | | | | | |
| Test for overall effect: Z = 1.96 (P = 0.05) | | | | | | |
| **1.11.4 Iron, zinc, vitamin B12** | | | | | | |
| Ma 2016 (C) | 2.1 | 1.4917 | 140 | 133 | 14.9% | 2.10 [-0.82, 5.02] |
| **Subtotal (95% CI)** | | | **140** | **133** | **14.9%** | **2.10 [-0.82, 5.02]** |
| Heterogeneity: Not applicable | | | | | | |
| Test for overall effect: Z = 1.41 (P = 0.16) | | | | | | |
| **1.11.6 Iron, zinc, vitamin A, niacin, folic acid** | | | | | | |
| Quintero 2011 | 0.9 | 1.0466 | 195 | 200 | 17.5% | 0.90 [-1.15, 2.95] |
| **Subtotal (95% CI)** | | | **195** | **200** | **17.5%** | **0.90 [-1.15, 2.95]** |
| Heterogeneity: Not applicable | | | | | | |
| Test for overall effect: Z = 0.86 (P = 0.39) | | | | | | |
| **1.11.7 Vitamin A, thiamine, riboflavin, pyridoxine** | | | | | | |
| Nesamvuni 2005 | 9 | 7.6844 | 16 | 20 | 1.8% | 9.00 [-6.06, 24.06] |
| **Subtotal (95% CI)** | | | **16** | **20** | **1.8%** | **9.00 [-6.06, 24.06]** |
| Heterogeneity: Not applicable | | | | | | |
| Test for overall effect: Z = 1.17 (P = 0.24) | | | | | | |
| **1.11.8 Multivitamins and minerals** | | | | | | |
| Faber 2005 | 9 | 1.4902 | 144 | 142 | 15.0% | 9.00 [6.08, 11.92] |
| Lartey 2000 | 4 | 4.3191 | 47 | 48 | 4.8% | 4.00 [-4.47, 12.47] |
| Liu 1993 (C) | 2.1 | 1.6469 | 77 | 85 | 14.1% | 2.10 [-1.13, 5.33] |
| **Subtotal (95% CI)** | | | **268** | **275** | **33.8%** | **5.26 [-0.07, 10.59]** |
| Heterogeneity: Tau² = 16.28; Chi² = 9.83, df = 2 (P = 0.007); I² = 80% | | | | | | |
| Test for overall effect: Z = 1.93 (P = 0.05) | | | | | | |
| **Total (95% CI)** | | | **1110** | **1065** | **100.0%** | **3.44 [1.33, 5.55]** |
| Heterogeneity: Tau² = 5.50; Chi² = 22.00, df = 10 (P = 0.02); I² = 55% | | | | | | |
| Test for overall effect: Z = 3.20 (P = 0.001) | | | | | | |
| Test for subgroup differences: Chi² = 3.74, df = 4 (P = 0.44), I² = 0% | | | | | | |

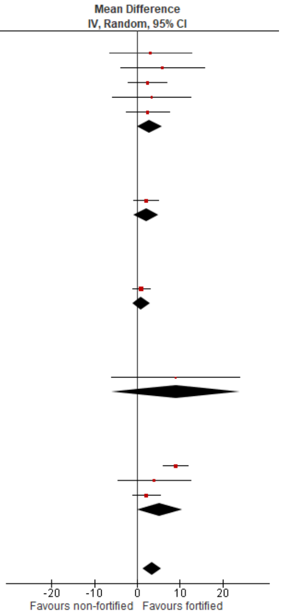

## 1.12 Fortified versus non-fortified complementary food. Outcome: Haemoglobin by types of products fortified

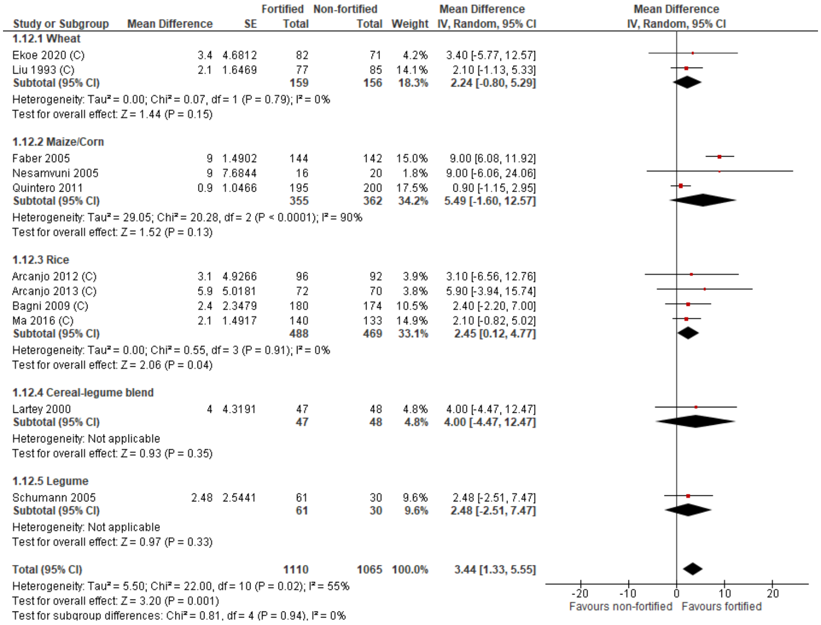


## 1.13 Fortified versus non-fortified complementary food. Outcome: Haemoglobin by duration of intervention

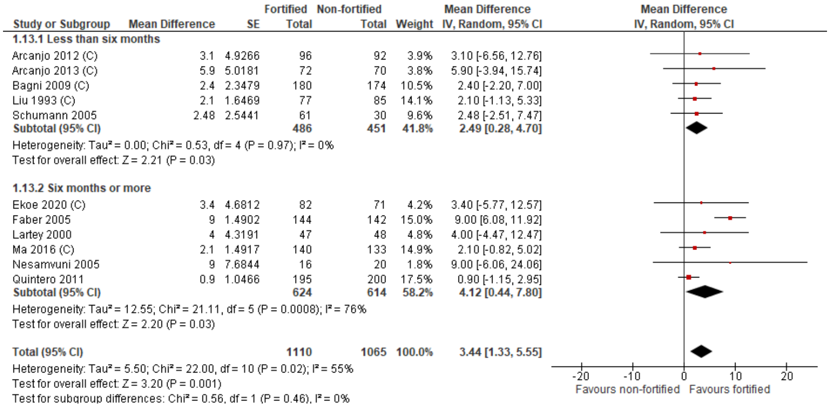

## 1.14 Fortified versus non-fortified complementary food. Outcome: Haemoglobin by baseline anaemia status

| Study or Subgroup | Mean Difference | SE | Fortified Total | Non-fortified Total | Weight | Mean Difference IV, Random, 95% CI |
|---|---|---|---|---|---|---|
| **1.14.1 Anaemic** | | | | | | |
| Ekoe 2020 (C) | 3.4 | 4.6812 | 82 | 71 | 4.2% | 3.40 [-5.77, 12.57] |
| Schumann 2005 | 2.48 | 2.5441 | 61 | 30 | 9.6% | 2.48 [-2.51, 7.47] |
| **Subtotal (95% CI)** | | | **143** | **101** | **13.9%** | **2.69 [-1.69, 7.07]** |
| Heterogeneity: Tau$^2$ = 0.00; Chi$^2$ = 0.03, df = 1 (P = 0.86); I$^2$ = 0% | | | | | | |
| Test for overall effect: Z = 1.20 (P = 0.23) | | | | | | |
| **1.14.2 Non-anaemic** | | | | | | |
| **Subtotal (95% CI)** | | | **0** | **0** | | **Not estimable** |
| Heterogeneity: Not applicable | | | | | | |
| Test for overall effect: Not applicable | | | | | | |
| **1.14.3 Mixed/ Unknown** | | | | | | |
| Arcanjo 2012 (C) | 3.1 | 4.9266 | 96 | 92 | 3.9% | 3.10 [-6.56, 12.76] |
| Arcanjo 2013 (C) | 5.9 | 5.0181 | 72 | 70 | 3.8% | 5.90 [-3.94, 15.74] |
| Bagni 2009 (C) | 2.4 | 2.3479 | 180 | 174 | 10.5% | 2.40 [-2.20, 7.00] |
| Faber 2005 | 9 | 1.4902 | 144 | 142 | 15.0% | 9.00 [6.08, 11.92] |
| Lartey 2000 | 4 | 4.3191 | 47 | 48 | 4.8% | 4.00 [-4.47, 12.47] |
| Liu 1993 (C) | 2.1 | 1.6469 | 77 | 85 | 14.1% | 2.10 [-1.13, 5.33] |
| Ma 2016 (C) | 2.1 | 1.4917 | 140 | 133 | 14.9% | 2.10 [-0.82, 5.02] |
| Nesamvuni 2005 | 9 | 7.6844 | 16 | 20 | 1.8% | 9.00 [-6.06, 24.06] |
| Quintero 2011 | 0.9 | 1.0466 | 195 | 200 | 17.5% | 0.90 [-1.15, 2.95] |
| **Subtotal (95% CI)** | | | **967** | **964** | **86.1%** | **3.59 [1.13, 6.06]** |
| Heterogeneity: Tau$^2$ = 7.14; Chi$^2$ = 21.94, df = 8 (P = 0.005); I$^2$ = 64% | | | | | | |
| Test for overall effect: Z = 2.85 (P = 0.004) | | | | | | |
| **Total (95% CI)** | | | **1110** | **1065** | **100.0%** | **3.44 [1.33, 5.55]** |
| Heterogeneity: Tau$^2$ = 5.50; Chi$^2$ = 22.00, df = 10 (P = 0.02); I$^2$ = 55% | | | | | | |
| Test for overall effect: Z = 3.20 (P = 0.001) | | | | | | |
| Test for subgroup differences: Chi$^2$ = 0.12, df = 1 (P = 0.73), I$^2$ = 0% | | | | | | |

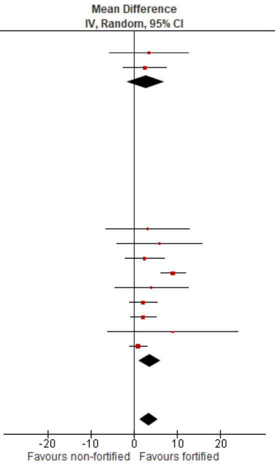


## 1.15 Fortified versus non-fortified complementary food. Outcome: Haemoglobin by country income classification

| Study or Subgroup | Mean Difference | SE | Fortified Total | Non-fortified Total | Weight | Mean Difference IV, Random, 95% CI |
|---|---|---|---|---|---|---|
| **1.15.1 High income** | | | | | | |
| **Subtotal (95% CI)** | | | **0** | **0** | | **Not estimable** |
| Heterogeneity: Not applicable | | | | | | |
| Test for overall effect: Not applicable | | | | | | |
| **1.15.2 Upper middle income** | | | | | | |
| Arcanjo 2012 (C) | 3.1 | 4.9266 | 96 | 92 | 3.9% | 3.10 [-6.56, 12.76] |
| Arcanjo 2013 (C) | 5.9 | 5.0181 | 72 | 70 | 3.8% | 5.90 [-3.94, 15.74] |
| Bagni 2009 (C) | 2.4 | 2.3479 | 180 | 174 | 10.5% | 2.40 [-2.20, 7.00] |
| Faber 2005 | 9 | 1.4902 | 144 | 142 | 15.0% | 9.00 [6.08, 11.92] |
| Liu 1993 (C) | 2.1 | 1.6469 | 77 | 85 | 14.1% | 2.10 [-1.13, 5.33] |
| Ma 2016 (C) | 2.1 | 1.4917 | 140 | 133 | 14.9% | 2.10 [-0.82, 5.02] |
| Nesamvuni 2005 | 9 | 7.6844 | 16 | 20 | 1.8% | 9.00 [-6.06, 24.06] |
| Quintero 2011 | 0.9 | 1.0466 | 195 | 200 | 17.5% | 0.90 [-1.15, 2.95] |
| Schumann 2005 | 2.48 | 2.5441 | 61 | 30 | 9.6% | 2.48 [-2.51, 7.47] |
| **Subtotal (95% CI)** | | | **981** | **946** | **91.0%** | **3.44 [1.08, 5.79]** |
| Heterogeneity: Tau$^2$ = 6.75; Chi$^2$ = 21.95, df = 8 (P = 0.005); I$^2$ = 64% | | | | | | |
| Test for overall effect: Z = 2.86 (P = 0.004) | | | | | | |
| **1.15.3 Lower middle income** | | | | | | |
| Ekoe 2020 (C) | 3.4 | 4.6812 | 82 | 71 | 4.2% | 3.40 [-5.77, 12.57] |
| Lartey 2000 | 4 | 4.3191 | 47 | 48 | 4.8% | 4.00 [-4.47, 12.47] |
| **Subtotal (95% CI)** | | | **129** | **119** | **9.0%** | **3.72 [-2.50, 9.95]** |
| Heterogeneity: Tau$^2$ = 0.00; Chi$^2$ = 0.01, df = 1 (P = 0.92); I$^2$ = 0% | | | | | | |
| Test for overall effect: Z = 1.17 (P = 0.24) | | | | | | |
| **1.15.4 Low income** | | | | | | |
| **Subtotal (95% CI)** | | | **0** | **0** | | **Not estimable** |
| Heterogeneity: Not applicable | | | | | | |
| Test for overall effect: Not applicable | | | | | | |
| **Total (95% CI)** | | | **1110** | **1065** | **100.0%** | **3.44 [1.33, 5.55]** |
| Heterogeneity: Tau$^2$ = 5.50; Chi$^2$ = 22.00, df = 10 (P = 0.02); I$^2$ = 55% | | | | | | |
| Test for overall effect: Z = 3.20 (P = 0.001) | | | | | | |
| Test for subgroup differences: Chi$^2$ = 0.01, df = 1 (P = 0.93), I$^2$ = 0% | | | | | | |

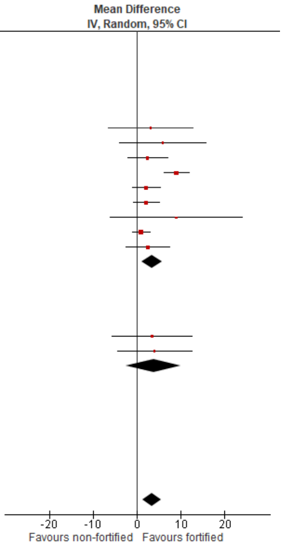

## 1.16 Fortified versus non-fortified complementary food. Outcome: Haemoglobin by study funding

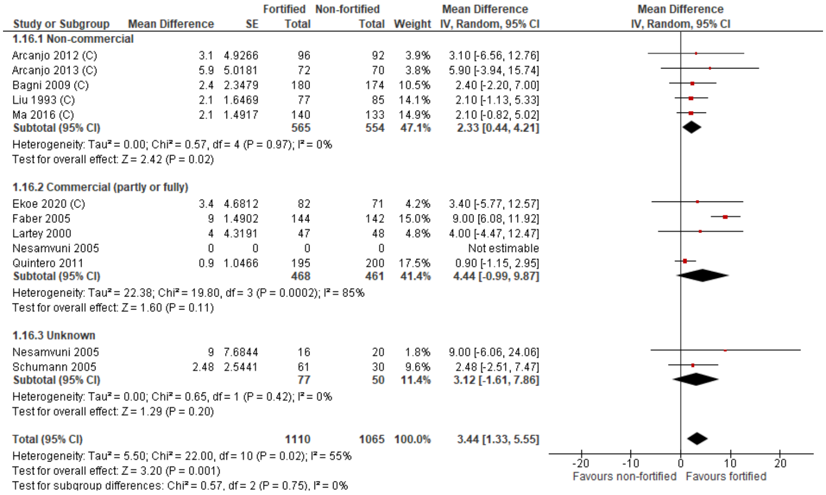


| Study or Subgroup | Mean Difference | SE | Fortified Total | Non-fortified Total | Weight | Mean Difference IV, Random, 95% CI |
|---|---|---|---|---|---|---|
| **1.16.1 Non-commercial** | | | | | | |
| Arcanjo 2012 (C) | 3.1 | 4.9266 | 96 | 92 | 3.9% | 3.10 [-6.56, 12.76] |
| Arcanjo 2013 (C) | 5.9 | 5.0181 | 72 | 70 | 3.8% | 5.90 [-3.94, 15.74] |
| Bagni 2009 (C) | 2.4 | 2.3479 | 180 | 174 | 10.5% | 2.40 [-2.20, 7.00] |
| Liu 1993 (C) | 2.1 | 1.6469 | 77 | 85 | 14.1% | 2.10 [-1.13, 5.33] |
| Ma 2016 (C) | 2.1 | 1.4917 | 140 | 133 | 14.9% | 2.10 [-0.82, 5.02] |
| **Subtotal (95% CI)** | | | **565** | **554** | **47.1%** | **2.33 [0.44, 4.21]** |
| Heterogeneity: $Tau^2 = 0.00$; $Chi^2 = 0.57$, df = 4 (P = 0.97); $I^2 = 0\%$ | | | | | | |
| Test for overall effect: Z = 2.42 (P = 0.02) | | | | | | |
| **1.16.2 Commercial (partly or fully)** | | | | | | |
| Ekoe 2020 (C) | 3.4 | 4.6812 | 82 | 71 | 4.2% | 3.40 [-5.77, 12.57] |
| Faber 2005 | 9 | 1.4902 | 144 | 142 | 15.0% | 9.00 [6.08, 11.92] |
| Lartey 2000 | 4 | 4.3191 | 47 | 48 | 4.8% | 4.00 [-4.47, 12.47] |
| Nesamvuni 2005 | 0 | 0 | 0 | 0 | | Not estimable |
| Quintero 2011 | 0.9 | 1.0466 | 195 | 200 | 17.5% | 0.90 [-1.15, 2.95] |
| **Subtotal (95% CI)** | | | **468** | **461** | **41.4%** | **4.44 [-0.99, 9.87]** |
| Heterogeneity: $Tau^2 = 22.38$; $Chi^2 = 19.80$, df = 3 (P = 0.0002); $I^2 = 85\%$ | | | | | | |
| Test for overall effect: Z = 1.60 (P = 0.11) | | | | | | |
| **1.16.3 Unknown** | | | | | | |
| Nesamvuni 2005 | 9 | 7.6844 | 16 | 20 | 1.8% | 9.00 [-6.06, 24.06] |
| Schumann 2005 | 2.48 | 2.5441 | 61 | 30 | 9.6% | 2.48 [-2.51, 7.47] |
| **Subtotal (95% CI)** | | | **77** | **50** | **11.4%** | **3.12 [-1.61, 7.86]** |
| Heterogeneity: $Tau^2 = 0.00$; $Chi^2 = 0.65$, df = 1 (P = 0.42); $I^2 = 0\%$ | | | | | | |
| Test for overall effect: Z = 1.29 (P = 0.20) | | | | | | |
| **Total (95% CI)** | | | **1110** | **1065** | **100.0%** | **3.44 [1.33, 5.55]** |
| Heterogeneity: $Tau^2 = 5.50$; $Chi^2 = 22.00$, df = 10 (P = 0.02); $I^2 = 55\%$ | | | | | | |
| Test for overall effect: Z = 3.20 (P = 0.001) | | | | | | |
| Test for subgroup differences: $Chi^2 = 0.57$, df = 2 (P = 0.75), $I^2 = 0\%$ | | | | | | |

## 1.17 Fortified versus non-fortified complementary food. Outcome: Weight-for-age (in z-scores)

*see Figure 1 in manuscript*

**1.18 Fortified versus non-fortified complementary food. Outcome: Weight-for-age (in z-scores) by age at the start of the intervention**

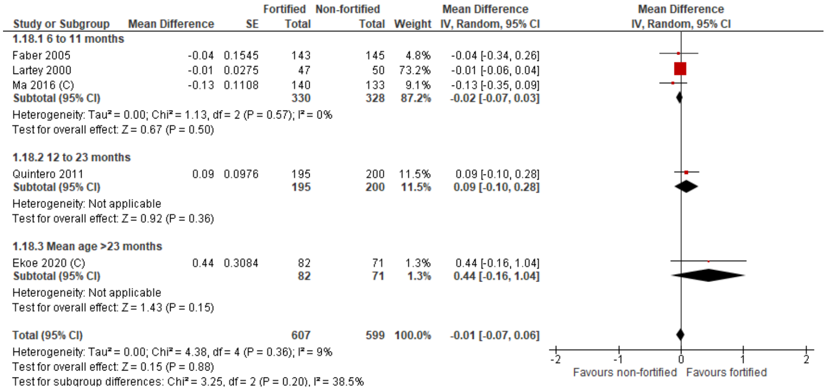


**1.19 Fortified versus non-fortified complementary food. Outcome: Weight-for-age (in z-scores) by types of nutrients added through fortification**

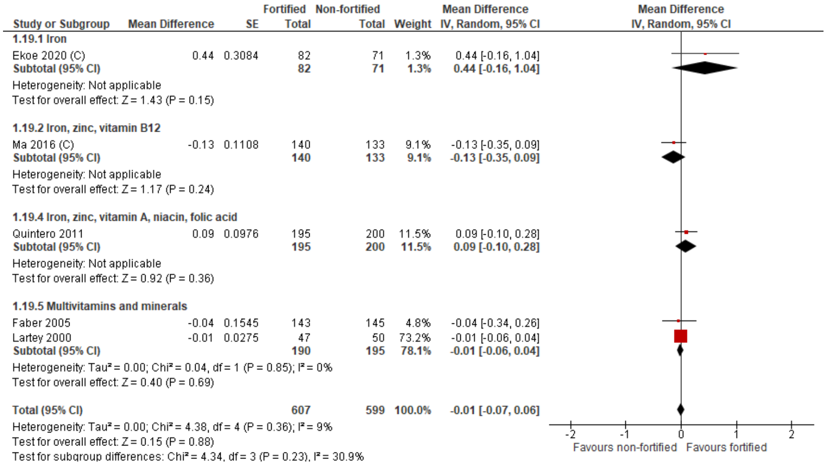

**1.20 Fortified versus non-fortified complementary food. Outcome: Weight-for-age (in z-scores) by types of products fortified**

| Study or Subgroup | Mean Difference | SE | Fortified Total | Non-fortified Total | Weight | Mean Difference IV, Random, 95% CI |
|---|---|---|---|---|---|---|
| **1.20.1 Wheat** | | | | | | |
| Ekoe 2020 (C) | 0.44 | 0.3084 | 82 | 71 | 1.3% | 0.44 [-0.16, 1.04] |
| **Subtotal (95% CI)** | | | **82** | **71** | **1.3%** | **0.44 [-0.16, 1.04]** |
| Heterogeneity: Not applicable | | | | | | |
| Test for overall effect: Z = 1.43 (P = 0.15) | | | | | | |
| **1.20.2 Maize/Corn** | | | | | | |
| Faber 2005 | -0.04 | 0.1545 | 143 | 145 | 4.8% | -0.04 [-0.34, 0.26] |
| Quintero 2011 | 0.09 | 0.0976 | 195 | 200 | 11.5% | 0.09 [-0.10, 0.28] |
| **Subtotal (95% CI)** | | | **338** | **345** | **16.4%** | **0.05 [-0.11, 0.21]** |
| Heterogeneity: Tau² = 0.00; Chi² = 0.51, df = 1 (P = 0.48); I² = 0% | | | | | | |
| Test for overall effect: Z = 0.64 (P = 0.52) | | | | | | |
| **1.20.3 Rice** | | | | | | |
| Ma 2016 (C) | -0.13 | 0.1108 | 140 | 133 | 9.1% | -0.13 [-0.35, 0.09] |
| **Subtotal (95% CI)** | | | **140** | **133** | **9.1%** | **-0.13 [-0.35, 0.09]** |
| Heterogeneity: Not applicable | | | | | | |
| Test for overall effect: Z = 1.17 (P = 0.24) | | | | | | |
| **1.20.4 Cereal-legume blend** | | | | | | |
| Lartey 2000 | -0.01 | 0.0275 | 47 | 50 | 73.2% | -0.01 [-0.06, 0.04] |
| **Subtotal (95% CI)** | | | **47** | **50** | **73.2%** | **-0.01 [-0.06, 0.04]** |
| Heterogeneity: Not applicable | | | | | | |
| Test for overall effect: Z = 0.36 (P = 0.72) | | | | | | |
| **Total (95% CI)** | | | **607** | **599** | **100.0%** | **-0.01 [-0.07, 0.06]** |
| Heterogeneity: Tau² = 0.00; Chi² = 4.38, df = 4 (P = 0.36); I² = 9% | | | | | | |
| Test for overall effect: Z = 0.15 (P = 0.88) | | | | | | |
| Test for subgroup differences: Chi² = 3.87, df = 3 (P = 0.28), I² = 22.5% | | | | | | |

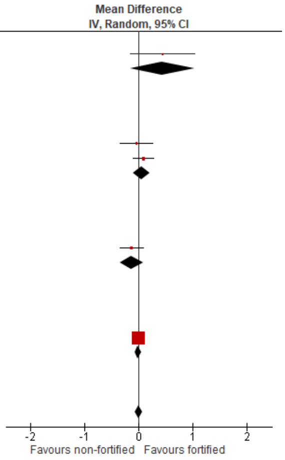


**1.21 Fortified versus non-fortified complementary food. Outcome: Weight-for-age (in z-scores) by duration of intervention**

| Study or Subgroup | Mean Difference | SE | Fortified Total | Non-fortified Total | Weight | Mean Difference IV, Random, 95% CI |
|---|---|---|---|---|---|---|
| **1.21.1 Less than six months** | | | | | | |
| **Subtotal (95% CI)** | | | **0** | **0** | | **Not estimable** |
| Heterogeneity: Not applicable | | | | | | |
| Test for overall effect: Not applicable | | | | | | |
| **1.21.2 Six months or more** | | | | | | |
| Ekoe 2020 (C) | 0.44 | 0.3084 | 82 | 71 | 1.3% | 0.44 [-0.16, 1.04] |
| Faber 2005 | -0.04 | 0.1545 | 143 | 145 | 4.8% | -0.04 [-0.34, 0.26] |
| Lartey 2000 | -0.01 | 0.0275 | 47 | 50 | 73.2% | -0.01 [-0.06, 0.04] |
| Ma 2016 (C) | -0.13 | 0.1108 | 140 | 133 | 9.1% | -0.13 [-0.35, 0.09] |
| Quintero 2011 | 0.09 | 0.0976 | 195 | 200 | 11.5% | 0.09 [-0.10, 0.28] |
| **Subtotal (95% CI)** | | | **607** | **599** | **100.0%** | **-0.01 [-0.07, 0.06]** |
| Heterogeneity: Tau² = 0.00; Chi² = 4.38, df = 4 (P = 0.36); I² = 9% | | | | | | |
| Test for overall effect: Z = 0.15 (P = 0.88) | | | | | | |
| **Total (95% CI)** | | | **607** | **599** | **100.0%** | **-0.01 [-0.07, 0.06]** |
| Heterogeneity: Tau² = 0.00; Chi² = 4.38, df = 4 (P = 0.36); I² = 9% | | | | | | |
| Test for overall effect: Z = 0.15 (P = 0.88) | | | | | | |
| Test for subgroup differences: Not applicable | | | | | | |

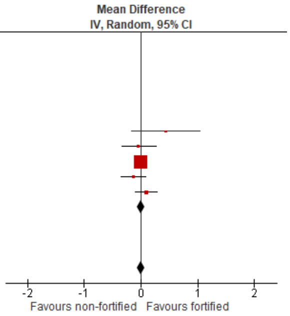


**1.22 Fortified versus non-fortified complementary food. Outcome: Weight-for-age (in z-scores) by baseline anaemia status**

| Study or Subgroup | Mean Difference | SE | Fortified Total | Non-fortified Total | Weight | Mean Difference IV, Random, 95% CI |
|---|---|---|---|---|---|---|
| **1.22.1 Anaemic** | | | | | | |
| Ekoe 2020 (C) | 0.44 | 0.3084 | 82 | 71 | 1.3% | 0.44 [-0.16, 1.04] |
| **Subtotal (95% CI)** | | | **82** | **71** | **1.3%** | **0.44 [-0.16, 1.04]** |
| Heterogeneity: Not applicable | | | | | | |
| Test for overall effect: Z = 1.43 (P = 0.15) | | | | | | |
| **1.22.2 Non-anaemic** | | | | | | |
| **Subtotal (95% CI)** | | | **0** | **0** | | **Not estimable** |
| Heterogeneity: Not applicable | | | | | | |
| Test for overall effect: Not applicable | | | | | | |
| **1.22.3 Mixed/ Unknown** | | | | | | |
| Faber 2005 | -0.04 | 0.1545 | 143 | 145 | 4.8% | -0.04 [-0.34, 0.26] |
| Lartey 2000 | -0.01 | 0.0275 | 47 | 50 | 73.2% | -0.01 [-0.06, 0.04] |
| Ma 2016 (C) | -0.13 | 0.1108 | 140 | 133 | 9.1% | -0.13 [-0.35, 0.09] |
| Quintero 2011 | 0.09 | 0.0976 | 195 | 200 | 11.5% | 0.09 [-0.10, 0.28] |
| **Subtotal (95% CI)** | | | **525** | **528** | **98.7%** | **-0.01 [-0.06, 0.04]** |
| Heterogeneity: Tau² = 0.00; Chi² = 2.26, df = 3 (P = 0.52); I² = 0% | | | | | | |
| Test for overall effect: Z = 0.41 (P = 0.68) | | | | | | |
| **Total (95% CI)** | | | **607** | **599** | **100.0%** | **-0.01 [-0.07, 0.06]** |
| Heterogeneity: Tau² = 0.00; Chi² = 4.38, df = 4 (P = 0.36); I² = 9% | | | | | | |
| Test for overall effect: Z = 0.15 (P = 0.88) | | | | | | |
| Test for subgroup differences: Chi² = 2.12, df = 1 (P = 0.15), I² = 52.8% | | | | | | |

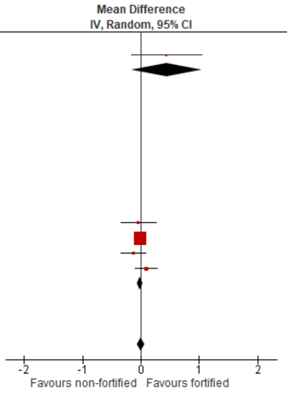

## 1.23 Fortified versus non-fortified complementary food. Outcome: Weight-for-age (in z-scores) by country income classification

| Study or Subgroup | Mean Difference | SE | Fortified Total | Non-fortified Total | Weight | Mean Difference IV, Random, 95% CI |
|---|---|---|---|---|---|---|
| **1.23.1 High income** | | | | | | |
| **Subtotal (95% CI)** | | | **0** | **0** | | **Not estimable** |
| Heterogeneity: Not applicable | | | | | | |
| Test for overall effect: Not applicable | | | | | | |
| **1.23.2 Upper middle income** | | | | | | |
| Faber 2005 | -0.04 | 0.1545 | 143 | 145 | 4.8% | -0.04 [-0.34, 0.26] |
| Ma 2016 (C) | -0.13 | 0.1108 | 140 | 133 | 9.1% | -0.13 [-0.35, 0.09] |
| Quintero 2011 | 0.09 | 0.0976 | 195 | 200 | 11.5% | 0.09 [-0.10, 0.28] |
| **Subtotal (95% CI)** | | | **478** | **478** | **25.5%** | **-0.01 [-0.15, 0.12]** |
| Heterogeneity: Tau² = 0.00; Chi² = 2.26, df = 2 (P = 0.32); I² = 11% | | | | | | |
| Test for overall effect: Z = 0.20 (P = 0.84) | | | | | | |
| **1.23.3 Lower middle income** | | | | | | |
| Ekoe 2020 (C) | 0.44 | 0.3084 | 82 | 71 | 1.3% | 0.44 [-0.16, 1.04] |
| Lartey 2000 | -0.01 | 0.0275 | 47 | 50 | 73.2% | -0.01 [-0.06, 0.04] |
| **Subtotal (95% CI)** | | | **129** | **121** | **74.5%** | **0.11 [-0.28, 0.50]** |
| Heterogeneity: Tau² = 0.05; Chi² = 2.11, df = 1 (P = 0.15); I² = 53% | | | | | | |
| Test for overall effect: Z = 0.55 (P = 0.58) | | | | | | |
| **1.23.4 Low income** | | | | | | |
| **Subtotal (95% CI)** | | | **0** | **0** | | **Not estimable** |
| Heterogeneity: Not applicable | | | | | | |
| Test for overall effect: Not applicable | | | | | | |
| **Total (95% CI)** | | | **607** | **599** | **100.0%** | **-0.01 [-0.07, 0.06]** |

Heterogeneity: Tau² = 0.00; Chi² = 4.38, df = 4 (P = 0.36); I² = 9%
Test for overall effect: Z = 0.15 (P = 0.88)
Test for subgroup differences: Chi² = 0.35, df = 1 (P = 0.56), I² = 0%

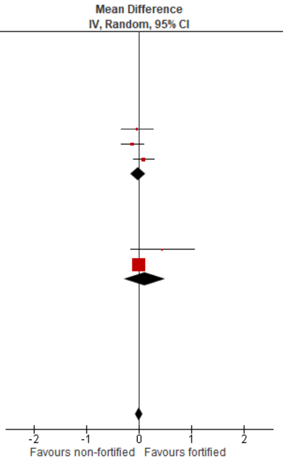


## 1.24 Fortified versus non-fortified complementary food. Outcome: Weight-for-age (in z-scores) by study funding

| Study or Subgroup | Mean Difference | SE | Fortified Total | Non-fortified Total | Weight | Mean Difference IV, Random, 95% CI |
|---|---|---|---|---|---|---|
| **1.24.1 Non-commercial** | | | | | | |
| Ma 2016 (C) | -0.13 | 0.1108 | 140 | 133 | 9.1% | -0.13 [-0.35, 0.09] |
| **Subtotal (95% CI)** | | | **140** | **133** | **9.1%** | **-0.13 [-0.35, 0.09]** |
| Heterogeneity: Not applicable | | | | | | |
| Test for overall effect: Z = 1.17 (P = 0.24) | | | | | | |
| **1.24.2 Commercial (partly or fully)** | | | | | | |
| Ekoe 2020 (C) | 0.44 | 0.3084 | 82 | 71 | 1.3% | 0.44 [-0.16, 1.04] |
| Faber 2005 | -0.04 | 0.1545 | 143 | 145 | 4.8% | -0.04 [-0.34, 0.26] |
| Lartey 2000 | -0.01 | 0.0275 | 47 | 50 | 73.2% | -0.01 [-0.06, 0.04] |
| Quintero 2011 | 0.09 | 0.0976 | 195 | 200 | 11.5% | 0.09 [-0.10, 0.28] |
| **Subtotal (95% CI)** | | | **467** | **466** | **90.9%** | **0.00 [-0.06, 0.06]** |
| Heterogeneity: Tau² = 0.00; Chi² = 3.08, df = 3 (P = 0.38); I² = 3% | | | | | | |
| Test for overall effect: Z = 0.08 (P = 0.94) | | | | | | |
| **Total (95% CI)** | | | **607** | **599** | **100.0%** | **-0.01 [-0.07, 0.06]** |

Heterogeneity: Tau² = 0.00; Chi² = 4.38, df = 4 (P = 0.36); I² = 9%
Test for overall effect: Z = 0.15 (P = 0.88)
Test for subgroup differences: Chi² = 1.33, df = 1 (P = 0.25), I² = 24.7%

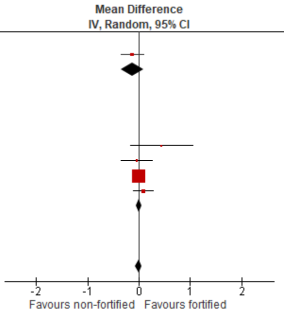


## 1.25 Fortified versus non-fortified complementary food. Outcome: Weight-for-length (in z-scores)

| Study or Subgroup | Mean Difference | SE | Fortified Total | Non-fortified Total | Weight | Mean Difference IV, Random, 95% CI |
|---|---|---|---|---|---|---|
| Ekoe 2020 (C) | -0.22 | 0.2367 | 82 | 71 | 8.9% | -0.22 [-0.68, 0.24] |
| Faber 2005 | -0.17 | 0.1403 | 143 | 145 | 21.6% | -0.17 [-0.44, 0.10] |
| Ma 2016 (C) | -0.09 | 0.104 | 140 | 133 | 32.9% | -0.09 [-0.29, 0.11] |
| Quintero 2011 | 0.11 | 0.0956 | 195 | 200 | 36.5% | 0.11 [-0.08, 0.30] |
| **Total (95% CI)** | | | **560** | **549** | **100.0%** | **-0.05 [-0.19, 0.10]** |

Heterogeneity: Tau² = 0.01; Chi² = 4.12, df = 3 (P = 0.25); I² = 27%
Test for overall effect: Z = 0.61 (P = 0.54)

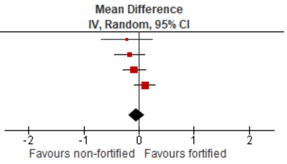

### 1.26 Fortified versus non-fortified complementary food. Outcome: Weight-for-length (in z-scores) by age at the start of the intervention

| Study or Subgroup | Mean Difference | SE | Fortified Total | Non-fortified Total | Weight | Mean Difference IV, Random, 95% CI |
|---|---|---|---|---|---|---|
| **1.26.1 6 to 11 months** | | | | | | |
| Faber 2005 | -0.17 | 0.1403 | 143 | 145 | 21.6% | -0.17 [-0.44, 0.10] |
| Ma 2016 (C) | -0.09 | 0.104 | 140 | 133 | 32.9% | -0.09 [-0.29, 0.11] |
| **Subtotal (95% CI)** | | | **283** | **278** | **54.5%** | **-0.12 [-0.28, 0.05]** |
| Heterogeneity: Tau² = 0.00; Chi² = 0.21, df = 1 (P = 0.65); I² = 0% | | | | | | |
| Test for overall effect: Z = 1.42 (P = 0.16) | | | | | | |
| **1.26.2 12 to 23 months** | | | | | | |
| Quintero 2011 | 0.11 | 0.0956 | 195 | 200 | 36.5% | 0.11 [-0.08, 0.30] |
| **Subtotal (95% CI)** | | | **195** | **200** | **36.5%** | **0.11 [-0.08, 0.30]** |
| Heterogeneity: Not applicable | | | | | | |
| Test for overall effect: Z = 1.15 (P = 0.25) | | | | | | |
| **1.26.3 Mean age >23 months** | | | | | | |
| Ekoe 2020 (C) | -0.22 | 0.2367 | 82 | 71 | 8.9% | -0.22 [-0.68, 0.24] |
| **Subtotal (95% CI)** | | | **82** | **71** | **8.9%** | **-0.22 [-0.68, 0.24]** |
| Heterogeneity: Not applicable | | | | | | |
| Test for overall effect: Z = 0.93 (P = 0.35) | | | | | | |
| **Total (95% CI)** | | | **560** | **549** | **100.0%** | **-0.05 [-0.19, 0.10]** |
| Heterogeneity: Tau² = 0.01; Chi² = 4.12, df = 3 (P = 0.25); I² = 27% | | | | | | |
| Test for overall effect: Z = 0.61 (P = 0.54) | | | | | | |
| Test for subgroup differences: Chi² = 3.91, df = 2 (P = 0.14), I² = 48.8% | | | | | | |

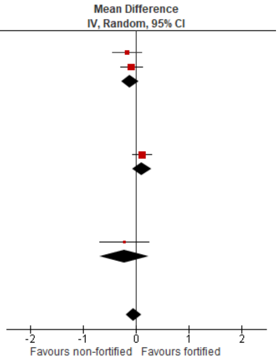


### 1.27 Fortified versus non-fortified complementary food. Outcome: Weight-for-length (in z-scores) by types of nutrients added through fortification

| Study or Subgroup | Mean Difference | SE | Fortified Total | Non-fortified Total | Weight | Mean Difference IV, Random, 95% CI |
|---|---|---|---|---|---|---|
| **1.27.1 Iron** | | | | | | |
| Ekoe 2020 (C) | -0.22 | 0.2367 | 82 | 71 | 8.9% | -0.22 [-0.68, 0.24] |
| **Subtotal (95% CI)** | | | **82** | **71** | **8.9%** | **-0.22 [-0.68, 0.24]** |
| Heterogeneity: Not applicable | | | | | | |
| Test for overall effect: Z = 0.93 (P = 0.35) | | | | | | |
| **1.27.2 Iron, zinc, vitamin B12** | | | | | | |
| Ma 2016 (C) | -0.09 | 0.104 | 140 | 133 | 32.9% | -0.09 [-0.29, 0.11] |
| **Subtotal (95% CI)** | | | **140** | **133** | **32.9%** | **-0.09 [-0.29, 0.11]** |
| Heterogeneity: Not applicable | | | | | | |
| Test for overall effect: Z = 0.87 (P = 0.39) | | | | | | |
| **1.27.3 Iron, zinc, vitamin A, niacin, folic acid** | | | | | | |
| Quintero 2011 | 0.11 | 0.0956 | 195 | 200 | 36.5% | 0.11 [-0.08, 0.30] |
| **Subtotal (95% CI)** | | | **195** | **200** | **36.5%** | **0.11 [-0.08, 0.30]** |
| Heterogeneity: Not applicable | | | | | | |
| Test for overall effect: Z = 1.15 (P = 0.25) | | | | | | |
| **1.27.4 Multivitamins and minerals** | | | | | | |
| Faber 2005 | -0.17 | 0.1403 | 143 | 145 | 21.6% | -0.17 [-0.44, 0.10] |
| **Subtotal (95% CI)** | | | **143** | **145** | **21.6%** | **-0.17 [-0.44, 0.10]** |
| Heterogeneity: Not applicable | | | | | | |
| Test for overall effect: Z = 1.21 (P = 0.23) | | | | | | |
| **Total (95% CI)** | | | **560** | **549** | **100.0%** | **-0.05 [-0.19, 0.10]** |
| Heterogeneity: Tau² = 0.01; Chi² = 4.12, df = 3 (P = 0.25); I² = 27% | | | | | | |
| Test for overall effect: Z = 0.61 (P = 0.54) | | | | | | |
| Test for subgroup differences: Chi² = 4.12, df = 3 (P = 0.25), I² = 27.1% | | | | | | |

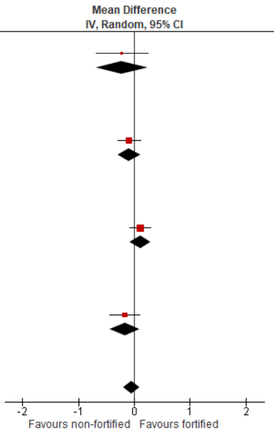


### 1.28 Fortified versus non-fortified complementary food. Outcome: Weight-for-length (in z-scores) by types of products fortified

| Study or Subgroup | Mean Difference | SE | Fortified Total | Non-fortified Total | Weight | Mean Difference IV, Random, 95% CI |
|---|---|---|---|---|---|---|
| **1.28.1 Wheat** | | | | | | |
| Ekoe 2020 (C) | -0.22 | 0.2367 | 82 | 71 | 8.9% | -0.22 [-0.68, 0.24] |
| **Subtotal (95% CI)** | | | **82** | **71** | **8.9%** | **-0.22 [-0.68, 0.24]** |
| Heterogeneity: Not applicable | | | | | | |
| Test for overall effect: Z = 0.93 (P = 0.35) | | | | | | |
| **1.28.2 Maize/Corn** | | | | | | |
| Faber 2005 | -0.17 | 0.1403 | 143 | 145 | 21.6% | -0.17 [-0.44, 0.10] |
| Quintero 2011 | 0.11 | 0.0956 | 195 | 200 | 36.5% | 0.11 [-0.08, 0.30] |
| **Subtotal (95% CI)** | | | **338** | **345** | **58.1%** | **-0.01 [-0.28, 0.26]** |
| Heterogeneity: Tau² = 0.02; Chi² = 2.72, df = 1 (P = 0.10); I² = 63% | | | | | | |
| Test for overall effect: Z = 0.08 (P = 0.94) | | | | | | |
| **1.28.3 Rice** | | | | | | |
| Ma 2016 (C) | -0.09 | 0.104 | 140 | 133 | 32.9% | -0.09 [-0.29, 0.11] |
| **Subtotal (95% CI)** | | | **140** | **133** | **32.9%** | **-0.09 [-0.29, 0.11]** |
| Heterogeneity: Not applicable | | | | | | |
| Test for overall effect: Z = 0.87 (P = 0.39) | | | | | | |
| **Total (95% CI)** | | | **560** | **549** | **100.0%** | **-0.05 [-0.19, 0.10]** |
| Heterogeneity: Tau² = 0.01; Chi² = 4.12, df = 3 (P = 0.25); I² = 27% | | | | | | |
| Test for overall effect: Z = 0.61 (P = 0.54) | | | | | | |
| Test for subgroup differences: Chi² = 0.61, df = 2 (P = 0.74), I² = 0% | | | | | | |

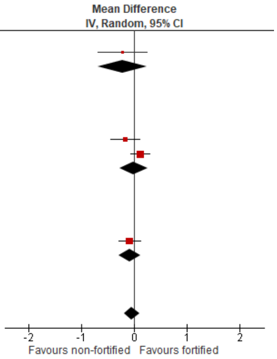

## 1.29 Fortified versus non-fortified complementary food. Outcome: Weight-for-length (in z-scores) by duration of intervention

| Study or Subgroup | Mean Difference | SE | Fortified Total | Non-fortified Total | Weight | Mean Difference IV, Random, 95% CI |
|---|---|---|---|---|---|---|
| **1.29.1 Less than six months** | | | | | | |
| **Subtotal (95% CI)** | | | **0** | **0** | | **Not estimable** |
| Heterogeneity: Not applicable | | | | | | |
| Test for overall effect: Not applicable | | | | | | |
| **1.29.2 Six months or more** | | | | | | |
| Ekoe 2020 (C) | -0.22 | 0.2367 | 82 | 71 | 8.9% | -0.22 [-0.68, 0.24] |
| Faber 2005 | -0.17 | 0.1403 | 143 | 145 | 21.6% | -0.17 [-0.44, 0.10] |
| Ma 2016 (C) | -0.09 | 0.104 | 140 | 133 | 32.9% | -0.09 [-0.29, 0.11] |
| Quintero 2011 | 0.11 | 0.0956 | 195 | 200 | 36.5% | 0.11 [-0.08, 0.30] |
| **Subtotal (95% CI)** | | | **560** | **549** | **100.0%** | **-0.05 [-0.19, 0.10]** |
| Heterogeneity: Tau² = 0.01; Chi² = 4.12, df = 3 (P = 0.25); I² = 27% | | | | | | |
| Test for overall effect: Z = 0.61 (P = 0.54) | | | | | | |
| **Total (95% CI)** | | | **560** | **549** | **100.0%** | **-0.05 [-0.19, 0.10]** |
| Heterogeneity: Tau² = 0.01; Chi² = 4.12, df = 3 (P = 0.25); I² = 27% | | | | | | |
| Test for overall effect: Z = 0.61 (P = 0.54) | | | | | | |
| Test for subgroup differences: Not applicable | | | | | | |

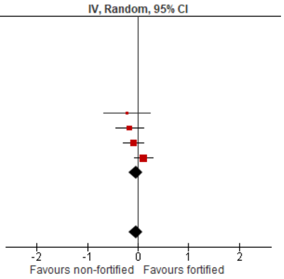


## 1.30 Fortified versus non-fortified complementary food. Outcome: Weight-for-length (in z-scores) by baseline anaemia status

| Study or Subgroup | Mean Difference | SE | Fortified Total | Non-fortified Total | Weight | Mean Difference IV, Random, 95% CI |
|---|---|---|---|---|---|---|
| **1.30.1 Anaemic** | | | | | | |
| Ekoe 2020 (C) | -0.22 | 0.2367 | 82 | 71 | 8.9% | -0.22 [-0.68, 0.24] |
| **Subtotal (95% CI)** | | | **82** | **71** | **8.9%** | **-0.22 [-0.68, 0.24]** |
| Heterogeneity: Not applicable | | | | | | |
| Test for overall effect: Z = 0.93 (P = 0.35) | | | | | | |
| **1.30.2 Non-anaemic** | | | | | | |
| **Subtotal (95% CI)** | | | **0** | **0** | | **Not estimable** |
| Heterogeneity: Not applicable | | | | | | |
| Test for overall effect: Not applicable | | | | | | |
| **1.30.3 Mixed/ Unknown** | | | | | | |
| Faber 2005 | -0.17 | 0.1403 | 143 | 145 | 21.6% | -0.17 [-0.44, 0.10] |
| Ma 2016 (C) | -0.09 | 0.104 | 140 | 133 | 32.9% | -0.09 [-0.29, 0.11] |
| Quintero 2011 | 0.11 | 0.0956 | 195 | 200 | 36.5% | 0.11 [-0.08, 0.30] |
| **Subtotal (95% CI)** | | | **478** | **478** | **91.1%** | **-0.03 [-0.20, 0.13]** |
| Heterogeneity: Tau² = 0.01; Chi² = 3.45, df = 2 (P = 0.18); I² = 42% | | | | | | |
| Test for overall effect: Z = 0.37 (P = 0.71) | | | | | | |
| **Total (95% CI)** | | | **560** | **549** | **100.0%** | **-0.05 [-0.19, 0.10]** |
| Heterogeneity: Tau² = 0.01; Chi² = 4.12, df = 3 (P = 0.25); I² = 27% | | | | | | |
| Test for overall effect: Z = 0.61 (P = 0.54) | | | | | | |
| Test for subgroup differences: Chi² = 0.56, df = 1 (P = 0.45), I² = 0% | | | | | | |

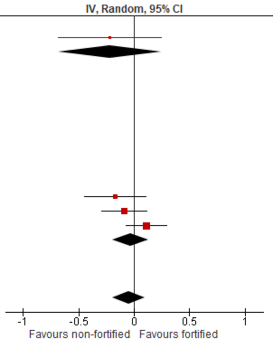


## 1.31 Fortified versus non-fortified complementary food. Outcome: Weight-for-length (in z-scores) by country income classification

| Study or Subgroup | Mean Difference | SE | Fortified Total | Non-fortified Total | Weight | Mean Difference IV, Random, 95% CI |
|---|---|---|---|---|---|---|
| **1.31.1 High income** | | | | | | |
| **Subtotal (95% CI)** | | | **0** | **0** | | **Not estimable** |
| Heterogeneity: Not applicable | | | | | | |
| Test for overall effect: Not applicable | | | | | | |
| **1.31.2 Upper middle income** | | | | | | |
| Faber 2005 | -0.17 | 0.1403 | 143 | 145 | 21.6% | -0.17 [-0.44, 0.10] |
| Ma 2016 (C) | -0.09 | 0.104 | 140 | 133 | 32.9% | -0.09 [-0.29, 0.11] |
| Quintero 2011 | 0.11 | 0.0956 | 195 | 200 | 36.5% | 0.11 [-0.08, 0.30] |
| **Subtotal (95% CI)** | | | **478** | **478** | **91.1%** | **-0.03 [-0.20, 0.13]** |
| Heterogeneity: Tau² = 0.01; Chi² = 3.45, df = 2 (P = 0.18); I² = 42% | | | | | | |
| Test for overall effect: Z = 0.37 (P = 0.71) | | | | | | |
| **1.31.3 Lower middle income** | | | | | | |
| Ekoe 2020 (C) | -0.22 | 0.2367 | 82 | 71 | 8.9% | -0.22 [-0.68, 0.24] |
| **Subtotal (95% CI)** | | | **82** | **71** | **8.9%** | **-0.22 [-0.68, 0.24]** |
| Heterogeneity: Not applicable | | | | | | |
| Test for overall effect: Z = 0.93 (P = 0.35) | | | | | | |
| **1.31.4 Low income** | | | | | | |
| **Subtotal (95% CI)** | | | **0** | **0** | | **Not estimable** |
| Heterogeneity: Not applicable | | | | | | |
| Test for overall effect: Not applicable | | | | | | |
| **Total (95% CI)** | | | **560** | **549** | **100.0%** | **-0.05 [-0.19, 0.10]** |
| Heterogeneity: Tau² = 0.01; Chi² = 4.12, df = 3 (P = 0.25); I² = 27% | | | | | | |
| Test for overall effect: Z = 0.61 (P = 0.54) | | | | | | |
| Test for subgroup differences: Chi² = 0.56, df = 1 (P = 0.45), I² = 0% | | | | | | |

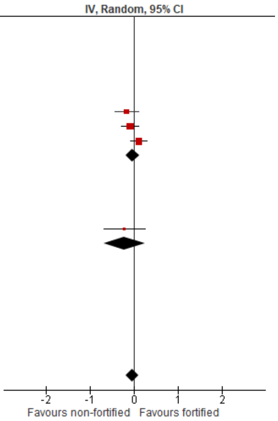

## 1.32 Fortified versus non-fortified complementary food. Outcome: Weight-for-length (in z-scores) by study funding

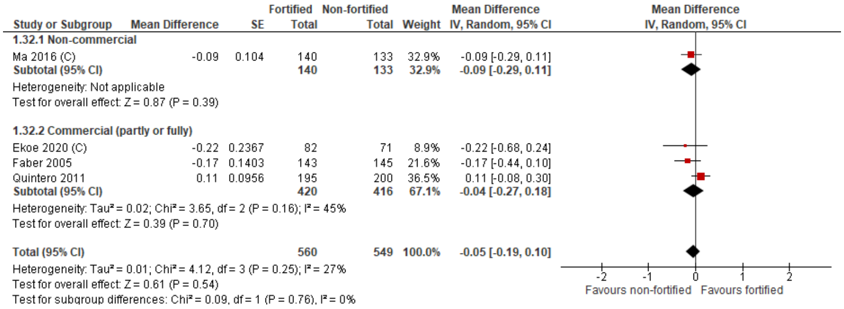


## 1.33 Fortified versus non-fortified complementary food. Outcome: Length-for-age (in z-scores)

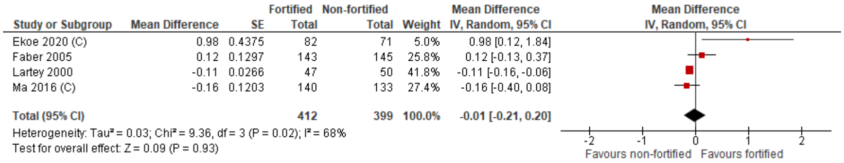


## 1.34 Fortified versus non-fortified complementary food. Outcome: Length-for-age (in z-scores) by age at the start of the intervention

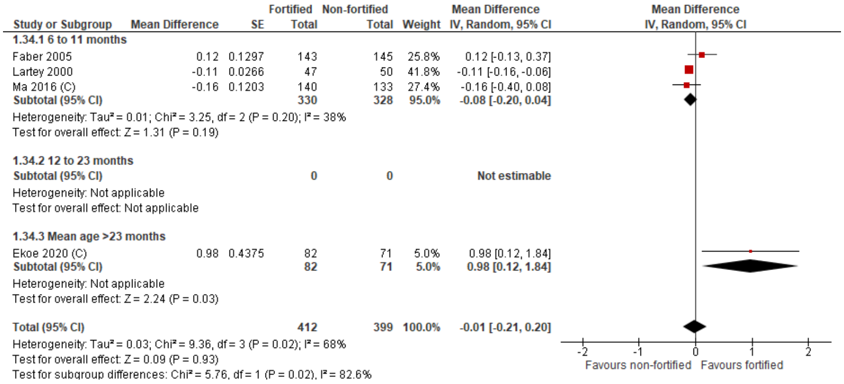


## 1.35 Fortified versus non-fortified complementary food. Outcome: Length-for-age (in z-scores) by types of nutrients added through fortification

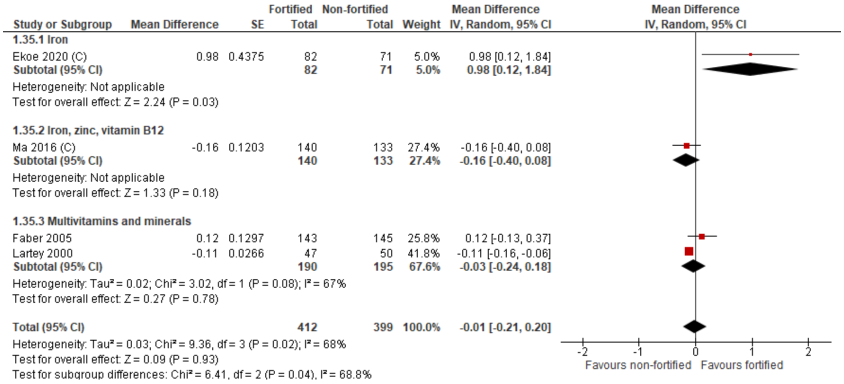

### 1.36 Fortified versus non-fortified complementary food. Outcome: Length-for-age (in z-scores) by types of products fortified

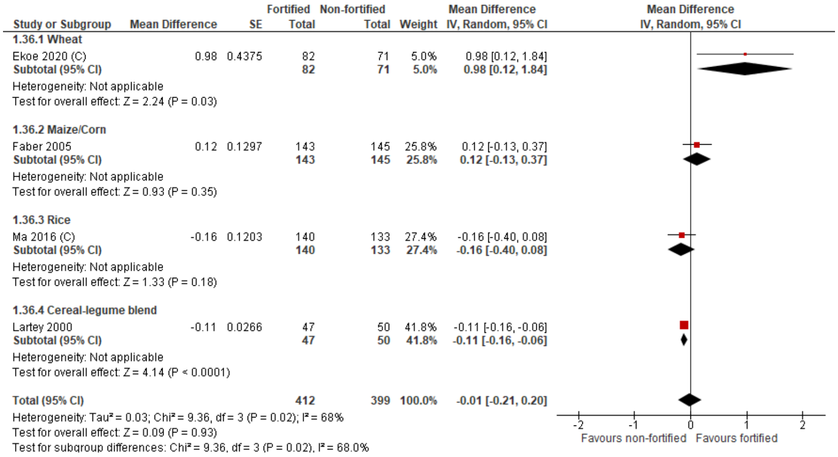


### 1.37 Fortified versus non-fortified complementary food. Outcome: Length-for-age (in z-scores) by duration of intervention

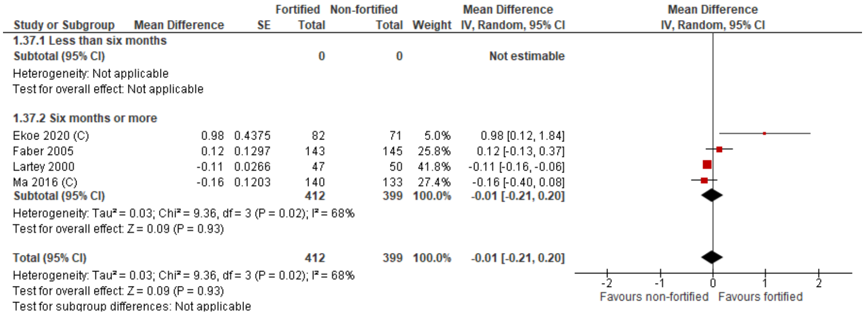


### 1.38 Fortified versus non-fortified complementary food. Outcome: Length-for-age (in z-scores) by baseline anaemia status

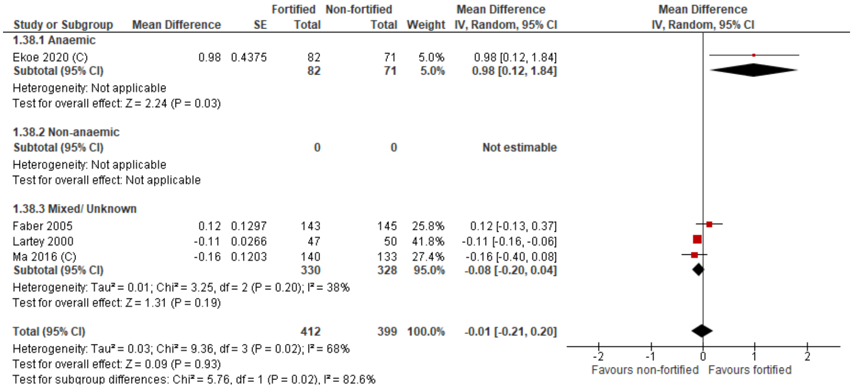

## 1.39 Fortified versus non-fortified complementary food. Outcome: Length-for-age (in z-scores) by country income classification

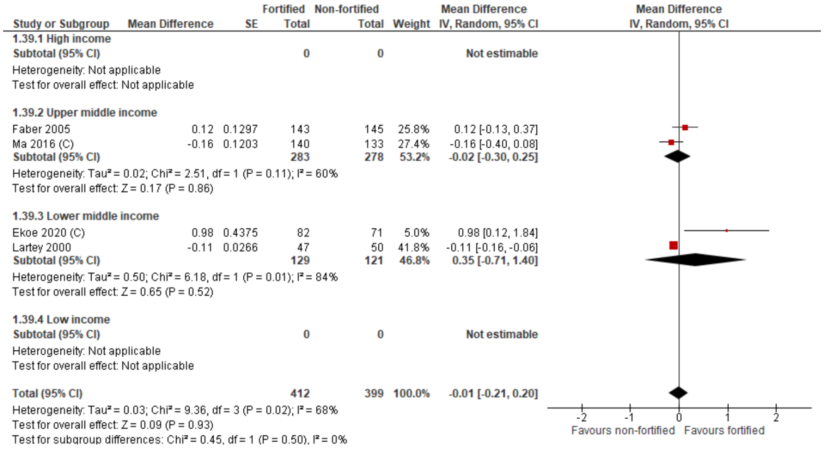


## 1.40 Fortified versus non-fortified complementary food. Outcome: Length-for-age (in z-scores) by study funding

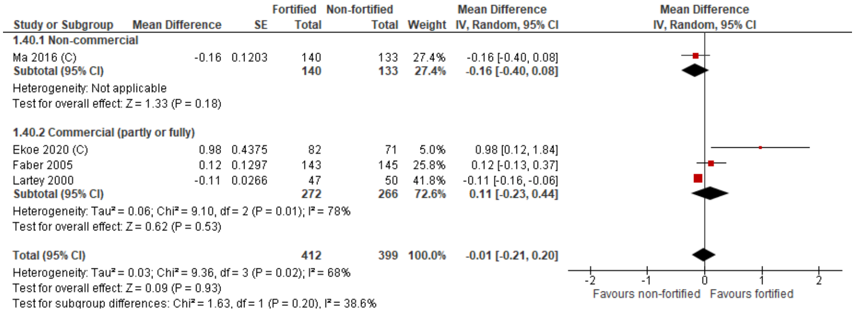


## 1.41. Fortified versus non-fortified complementary food. Outcome: Iron status (ferritin concentrations in µg/L)

*see Figure 4 in manuscript*

## 1.42. Fortified versus non-fortified complementary food. Outcome: Iron status (ferritin) by age at start of the intervention

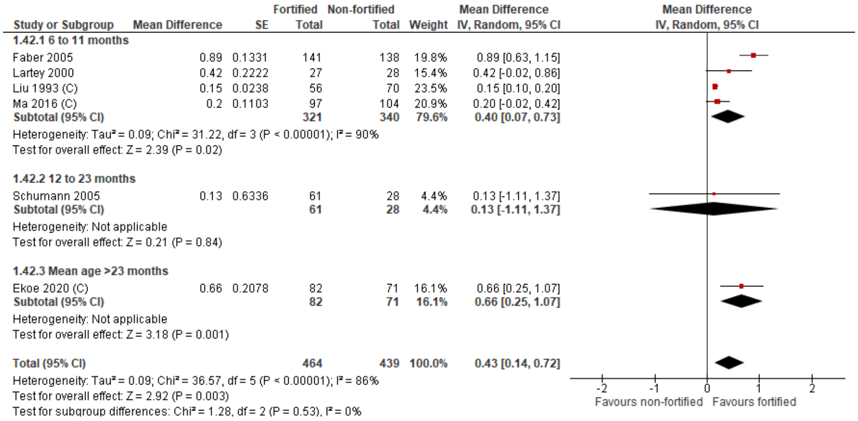

## 1.43. Fortified versus non-fortified complementary food. Outcome: Iron status (ferritin) by types of nutrients added through fortification

| Study or Subgroup | Mean Difference | SE | Fortified Total | Non-fortified Total | Weight | Mean Difference IV, Random, 95% CI |
|---|---|---|---|---|---|---|
| **1.43.1 Iron** | | | | | | |
| Ekoe 2020 (C) | 0.66 | 0.2078 | 82 | 71 | 16.1% | 0.66 [0.25, 1.07] |
| Schumann 2005 | 0.13 | 0.6336 | 61 | 28 | 4.4% | 0.13 [-1.11, 1.37] |
| **Subtotal (95% CI)** | | | **143** | **99** | **20.4%** | **0.61 [0.22, 1.00]** |
| Heterogeneity: Tau² = 0.00; Chi² = 0.63, df = 1 (P = 0.43); I² = 0% | | | | | | |
| Test for overall effect: Z = 3.08 (P = 0.002) | | | | | | |
| **1.43.3 Iron, zinc, vitamin B12** | | | | | | |
| Ma 2016 (C) | 0.2 | 0.1103 | 97 | 104 | 20.9% | 0.20 [-0.02, 0.42] |
| **Subtotal (95% CI)** | | | **97** | **104** | **20.9%** | **0.20 [-0.02, 0.42]** |
| Heterogeneity: Not applicable | | | | | | |
| Test for overall effect: Z = 1.81 (P = 0.07) | | | | | | |
| **1.43.4 Multivitamins and minerals** | | | | | | |
| Faber 2005 | 0.89 | 0.1331 | 141 | 138 | 19.8% | 0.89 [0.63, 1.15] |
| Lartey 2000 | 0.42 | 0.2222 | 27 | 28 | 15.4% | 0.42 [-0.02, 0.86] |
| Liu 1993 (C) | 0.15 | 0.0238 | 56 | 70 | 23.5% | 0.15 [0.10, 0.20] |
| **Subtotal (95% CI)** | | | **224** | **236** | **58.7%** | **0.48 [-0.05, 1.01]** |
| Heterogeneity: Tau² = 0.20; Chi² = 31.18, df = 2 (P < 0.00001); I² = 94% | | | | | | |
| Test for overall effect: Z = 1.78 (P = 0.07) | | | | | | |
| **Total (95% CI)** | | | **464** | **439** | **100.0%** | **0.43 [0.14, 0.72]** |
| Heterogeneity: Tau² = 0.09; Chi² = 36.57, df = 5 (P < 0.00001); I² = 86% | | | | | | |
| Test for overall effect: Z = 2.92 (P = 0.003) | | | | | | |
| Test for subgroup differences: Chi² = 3.67, df = 2 (P = 0.16), I² = 45.5% | | | | | | |

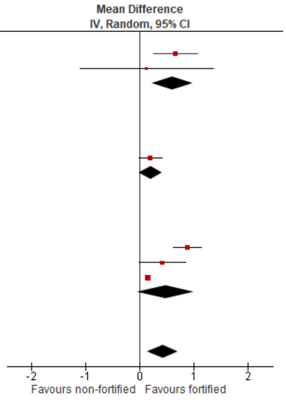


## 1.44. Fortified versus non-fortified complementary food. Outcome: Iron status (ferritin) by types of products fortified

| Study or Subgroup | Mean Difference | SE | Fortified Total | Non-fortified Total | Weight | Mean Difference IV, Random, 95% CI |
|---|---|---|---|---|---|---|
| **1.44.1 Wheat** | | | | | | |
| Ekoe 2020 (C) | 0.66 | 0.2078 | 82 | 71 | 16.1% | 0.66 [0.25, 1.07] |
| Liu 1993 (C) | 0.15 | 0.0238 | 56 | 70 | 23.5% | 0.15 [0.10, 0.20] |
| **Subtotal (95% CI)** | | | **138** | **141** | **39.6%** | **0.36 [-0.13, 0.86]** |
| Heterogeneity: Tau² = 0.11; Chi² = 5.95, df = 1 (P = 0.01); I² = 83% | | | | | | |
| Test for overall effect: Z = 1.44 (P = 0.15) | | | | | | |
| **1.44.2 Maize/Corn** | | | | | | |
| Faber 2005 | 0.89 | 0.1331 | 141 | 138 | 19.8% | 0.89 [0.63, 1.15] |
| **Subtotal (95% CI)** | | | **141** | **138** | **19.8%** | **0.89 [0.63, 1.15]** |
| Heterogeneity: Not applicable | | | | | | |
| Test for overall effect: Z = 6.69 (P < 0.00001) | | | | | | |
| **1.44.3 Rice** | | | | | | |
| Ma 2016 (C) | 0.2 | 0.1103 | 97 | 104 | 20.9% | 0.20 [-0.02, 0.42] |
| **Subtotal (95% CI)** | | | **97** | **104** | **20.9%** | **0.20 [-0.02, 0.42]** |
| Heterogeneity: Not applicable | | | | | | |
| Test for overall effect: Z = 1.81 (P = 0.07) | | | | | | |
| **1.44.4 Cereal-legume blend** | | | | | | |
| Lartey 2000 | 0.42 | 0.2222 | 27 | 28 | 15.4% | 0.42 [-0.02, 0.86] |
| **Subtotal (95% CI)** | | | **27** | **28** | **15.4%** | **0.42 [-0.02, 0.86]** |
| Heterogeneity: Not applicable | | | | | | |
| Test for overall effect: Z = 1.89 (P = 0.06) | | | | | | |
| **1.44.5 Legume** | | | | | | |
| Schumann 2005 | 0.13 | 0.6336 | 61 | 28 | 4.4% | 0.13 [-1.11, 1.37] |
| **Subtotal (95% CI)** | | | **61** | **28** | **4.4%** | **0.13 [-1.11, 1.37]** |
| Heterogeneity: Not applicable | | | | | | |
| Test for overall effect: Z = 0.21 (P = 0.84) | | | | | | |
| **Total (95% CI)** | | | **464** | **439** | **100.0%** | **0.43 [0.14, 0.72]** |
| Heterogeneity: Tau² = 0.09; Chi² = 36.57, df = 5 (P < 0.00001); I² = 86% | | | | | | |
| Test for overall effect: Z = 2.92 (P = 0.003) | | | | | | |
| Test for subgroup differences: Chi² = 16.44, df = 4 (P = 0.002), I² = 75.7% | | | | | | |

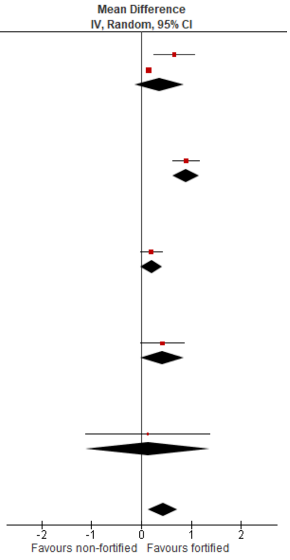


## 1.45. Fortified versus non-fortified complementary food. Outcome: Iron status (ferritin) by duration of intervention

| Study or Subgroup | Mean Difference | SE | Fortified Total | Non-fortified Total | Weight | Mean Difference IV, Random, 95% CI |
|---|---|---|---|---|---|---|
| **1.45.1 Less than six months** | | | | | | |
| Liu 1993 (C) | 0.15 | 0.0238 | 56 | 70 | 23.5% | 0.15 [0.10, 0.20] |
| Schumann 2005 | 0.13 | 0.6336 | 61 | 28 | 4.4% | 0.13 [-1.11, 1.37] |
| **Subtotal (95% CI)** | | | **117** | **98** | **27.9%** | **0.15 [0.10, 0.20]** |
| Heterogeneity: Tau² = 0.00; Chi² = 0.00, df = 1 (P = 0.97); I² = 0% | | | | | | |
| Test for overall effect: Z = 6.31 (P < 0.00001) | | | | | | |
| **1.45.2 Six months or more** | | | | | | |
| Ekoe 2020 (C) | 0.66 | 0.2078 | 82 | 71 | 16.1% | 0.66 [0.25, 1.07] |
| Faber 2005 | 0.89 | 0.1331 | 141 | 138 | 19.8% | 0.89 [0.63, 1.15] |
| Lartey 2000 | 0.42 | 0.2222 | 27 | 28 | 15.4% | 0.42 [-0.02, 0.86] |
| Ma 2016 (C) | 0.2 | 0.1103 | 97 | 104 | 20.9% | 0.20 [-0.02, 0.42] |
| **Subtotal (95% CI)** | | | **347** | **341** | **72.1%** | **0.54 [0.17, 0.91]** |
| Heterogeneity: Tau² = 0.11; Chi² = 16.70, df = 3 (P = 0.0008); I² = 82% | | | | | | |
| Test for overall effect: Z = 2.87 (P = 0.004) | | | | | | |
| **Total (95% CI)** | | | **464** | **439** | **100.0%** | **0.43 [0.14, 0.72]** |
| Heterogeneity: Tau² = 0.09; Chi² = 36.57, df = 5 (P < 0.00001); I² = 86% | | | | | | |
| Test for overall effect: Z = 2.92 (P = 0.003) | | | | | | |
| Test for subgroup differences: Chi² = 4.24, df = 1 (P = 0.04), I² = 76.4% | | | | | | |

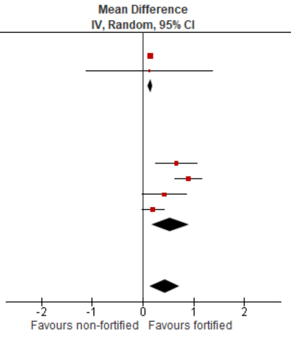

## 1.46. Fortified versus non-fortified complementary food. Outcome: Iron status (ferritin) by baseline anaemia status

| Study or Subgroup | Mean Difference | SE | Fortified Total | Non-fortified Total | Weight | Mean Difference IV, Random, 95% CI |
|---|---|---|---|---|---|---|
| **1.46.1 Anaemic** | | | | | | |
| Ekoe 2020 (C) | 0.66 | 0.2078 | 82 | 71 | 16.1% | 0.66 [0.25, 1.07] |
| Schumann 2005 | 0.13 | 0.6336 | 61 | 28 | 4.4% | 0.13 [-1.11, 1.37] |
| **Subtotal (95% CI)** | | | **143** | **99** | **20.4%** | **0.61 [0.22, 1.00]** |
| Heterogeneity: Tau² = 0.00; Chi² = 0.63, df = 1 (P = 0.43); I² = 0% | | | | | | |
| Test for overall effect: Z = 3.08 (P = 0.002) | | | | | | |
| **1.46.2 Non-anaemic** | | | | | | |
| **Subtotal (95% CI)** | | | **0** | **0** | | **Not estimable** |
| Heterogeneity: Not applicable | | | | | | |
| Test for overall effect: Not applicable | | | | | | |
| **1.46.3 Mixed/Unknown** | | | | | | |
| Faber 2005 | 0.89 | 0.1331 | 141 | 138 | 19.8% | 0.89 [0.63, 1.15] |
| Lartey 2000 | 0.42 | 0.2222 | 27 | 28 | 15.4% | 0.42 [-0.02, 0.86] |
| Liu 1993 (C) | 0.15 | 0.0238 | 56 | 70 | 23.5% | 0.15 [0.10, 0.20] |
| Ma 2016 (C) | 0.2 | 0.1103 | 97 | 104 | 20.9% | 0.20 [-0.02, 0.42] |
| **Subtotal (95% CI)** | | | **321** | **340** | **79.6%** | **0.40 [0.07, 0.73]** |
| Heterogeneity: Tau² = 0.09; Chi² = 31.22, df = 3 (P < 0.00001); I² = 90% | | | | | | |
| Test for overall effect: Z = 2.39 (P = 0.02) | | | | | | |
| **Total (95% CI)** | | | **464** | **439** | **100.0%** | **0.43 [0.14, 0.72]** |
| Heterogeneity: Tau² = 0.09; Chi² = 36.57, df = 5 (P < 0.00001); I² = 86% | | | | | | |
| Test for overall effect: Z = 2.92 (P = 0.003) | | | | | | |
| Test for subgroup differences: Chi² = 0.65, df = 1 (P = 0.42), I² = 0% | | | | | | |

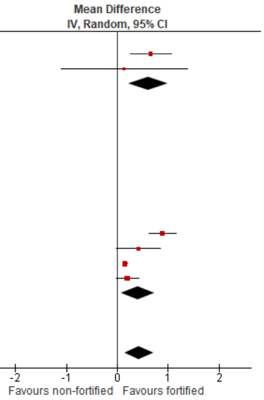


## 1.47. Fortified versus non-fortified complementary food. Outcome: Iron status (ferritin) by country income classification

| Study or Subgroup | Mean Difference | SE | Fortified Total | Non-fortified Total | Weight | Mean Difference IV, Random, 95% CI |
|---|---|---|---|---|---|---|
| **1.47.1 High income** | | | | | | |
| **Subtotal (95% CI)** | | | **0** | **0** | | **Not estimable** |
| Heterogeneity: Not applicable | | | | | | |
| Test for overall effect: Not applicable | | | | | | |
| **1.47.2 Upper middle income** | | | | | | |
| Faber 2005 | 0.89 | 0.1331 | 141 | 138 | 19.8% | 0.89 [0.63, 1.15] |
| Liu 1993 (C) | 0.15 | 0.0238 | 56 | 70 | 23.5% | 0.15 [0.10, 0.20] |
| Ma 2016 (C) | 0.2 | 0.1103 | 97 | 104 | 20.9% | 0.20 [-0.02, 0.42] |
| Schumann 2005 | 0.13 | 0.6336 | 61 | 28 | 4.4% | 0.13 [-1.11, 1.37] |
| **Subtotal (95% CI)** | | | **355** | **340** | **68.6%** | **0.38 [0.01, 0.74]** |
| Heterogeneity: Tau² = 0.10; Chi² = 30.02, df = 3 (P < 0.00001); I² = 90% | | | | | | |
| Test for overall effect: Z = 2.04 (P = 0.04) | | | | | | |
| **1.47.3 Lower middle income** | | | | | | |
| Ekoe 2020 (C) | 0.66 | 0.2078 | 82 | 71 | 16.1% | 0.66 [0.25, 1.07] |
| Lartey 2000 | 0.42 | 0.2222 | 27 | 28 | 15.4% | 0.42 [-0.02, 0.86] |
| **Subtotal (95% CI)** | | | **109** | **99** | **31.4%** | **0.55 [0.25, 0.85]** |
| Heterogeneity: Tau² = 0.00; Chi² = 0.62, df = 1 (P = 0.43); I² = 0% | | | | | | |
| Test for overall effect: Z = 3.61 (P = 0.0003) | | | | | | |
| **1.47.4 Low income** | | | | | | |
| **Subtotal (95% CI)** | | | **0** | **0** | | **Not estimable** |
| Heterogeneity: Not applicable | | | | | | |
| Test for overall effect: Not applicable | | | | | | |
| **Total (95% CI)** | | | **464** | **439** | **100.0%** | **0.43 [0.14, 0.72]** |
| Heterogeneity: Tau² = 0.09; Chi² = 36.57, df = 5 (P < 0.00001); I² = 86% | | | | | | |
| Test for overall effect: Z = 2.92 (P = 0.003) | | | | | | |
| Test for subgroup differences: Chi² = 0.50, df = 1 (P = 0.48), I² = 0% | | | | | | |

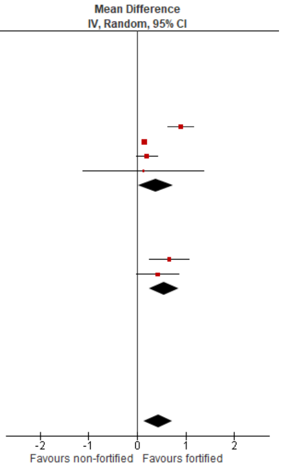


## 1.48. Fortified versus non-fortified complementary food. Outcome: Iron status (ferritin) by study funding

| Study or Subgroup | Mean Difference | SE | Fortified Total | Non-fortified Total | Weight | Mean Difference IV, Random, 95% CI |
|---|---|---|---|---|---|---|
| **1.48.1 Non-commercial** | | | | | | |
| Liu 1993 (C) | 0.15 | 0.0238 | 56 | 70 | 23.5% | 0.15 [0.10, 0.20] |
| Ma 2016 (C) | 0.2 | 0.1103 | 97 | 104 | 20.9% | 0.20 [-0.02, 0.42] |
| **Subtotal (95% CI)** | | | **153** | **174** | **44.4%** | **0.15 [0.11, 0.20]** |
| Heterogeneity: Tau² = 0.00; Chi² = 0.20, df = 1 (P = 0.66); I² = 0% | | | | | | |
| Test for overall effect: Z = 6.54 (P < 0.00001) | | | | | | |
| **1.48.2 Commercial (partly or fully)** | | | | | | |
| Ekoe 2020 (C) | 0.66 | 0.2078 | 82 | 71 | 16.1% | 0.66 [0.25, 1.07] |
| Faber 2005 | 0.89 | 0.1331 | 141 | 138 | 19.8% | 0.89 [0.63, 1.15] |
| Lartey 2000 | 0.42 | 0.2222 | 27 | 28 | 15.4% | 0.42 [-0.02, 0.86] |
| **Subtotal (95% CI)** | | | **250** | **237** | **51.2%** | **0.70 [0.43, 0.98]** |
| Heterogeneity: Tau² = 0.03; Chi² = 3.49, df = 2 (P = 0.17); I² = 43% | | | | | | |
| Test for overall effect: Z = 5.01 (P < 0.00001) | | | | | | |
| **1.48.3 Unknown** | | | | | | |
| Schumann 2005 | 0.13 | 0.6336 | 61 | 28 | 4.4% | 0.13 [-1.11, 1.37] |
| **Subtotal (95% CI)** | | | **61** | **28** | **4.4%** | **0.13 [-1.11, 1.37]** |
| Heterogeneity: Not applicable | | | | | | |
| Test for overall effect: Z = 0.21 (P = 0.84) | | | | | | |
| **Total (95% CI)** | | | **464** | **439** | **100.0%** | **0.43 [0.14, 0.72]** |
| Heterogeneity: Tau² = 0.09; Chi² = 36.57, df = 5 (P < 0.00001); I² = 86% | | | | | | |
| Test for overall effect: Z = 2.92 (P = 0.003) | | | | | | |
| Test for subgroup differences: Chi² = 14.99, df = 2 (P = 0.0006), I² = 86.7% | | | | | | |

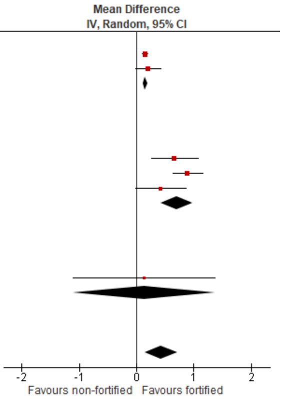


## 1.49. Fortified versus non-fortified complementary food. Outcome: Iron status (body iron in mg/kg)

| Study or Subgroup | Std. Mean Difference | SE | Favours non-fortified Total | Non-fortified Total | Weight | Std. Mean Difference IV, Random, 95% CI |
|---|---|---|---|---|---|---|
| Ma 2016 (C) | 1.47 | 0.4309 | 97 | 104 | 100.0% | 1.47 [0.63, 2.31] |
| **Total (95% CI)** | | | **97** | **104** | **100.0%** | **1.47 [0.63, 2.31]** |
| Heterogeneity: Not applicable | | | | | | |
| Test for overall effect: Z = 3.41 (P = 0.0006) | | | | | | |

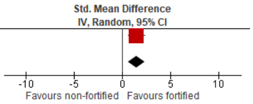

### 1.50. Fortified versus non-fortified complementary food. Outcome: Iron status (free erythrocyte porphyrin in µg/L)

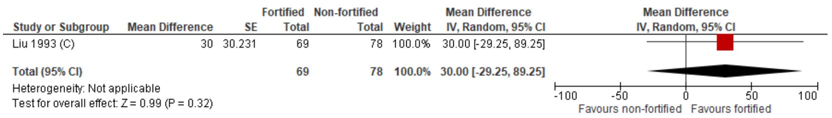


### 1.51. Fortified versus non-fortified complementary food. Outcome: Serum retinol (µmol/L)

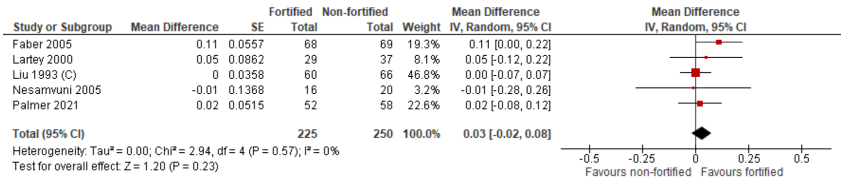


### 1.52. Serum zinc concentration

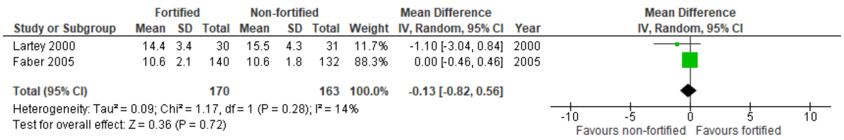


### 1.53. Fortified versus non-fortified complementary food. Outcome: Morbidity

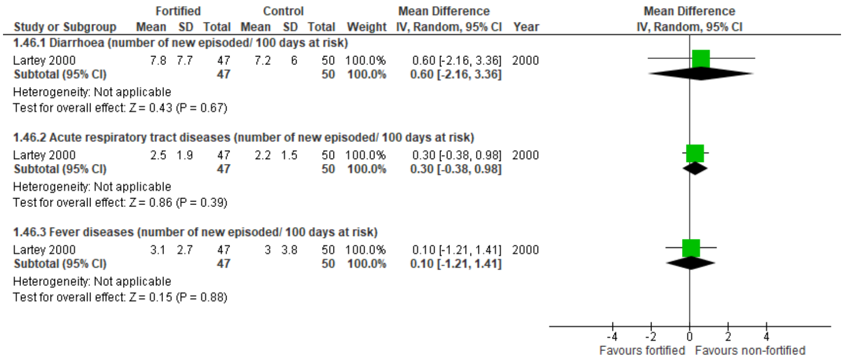


### 1.54. Fortified versus non-fortified complementary food. Outcome: Mental skill development

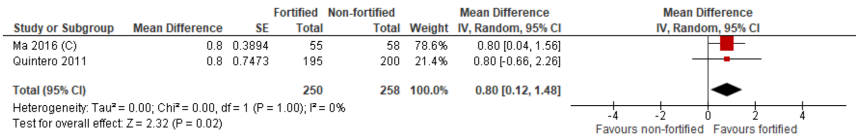

## 1.55. Fortified versus non-fortified complementary food. Outcome: Motor skill development

| Study or Subgroup | Mean Difference | SE | Fortified Total | Non-fortified Total | Weight | Mean Difference IV, Random, 95% CI |
|---|---|---|---|---|---|---|
| **1.55.1 Fine motor score (BSID III)** | | | | | | |
| Ma 2016 (C) | 0 | 0.205 | 55 | 58 | 100.0% | 0.00 [-0.40, 0.40] |
| **Subtotal (95% CI)** | | | **55** | **58** | **100.0%** | **0.00 [-0.40, 0.40]** |
| Heterogeneity: Not applicable | | | | | | |
| Test for overall effect: Z = 0.00 (P = 1.00) | | | | | | |
| **1.55.2 Gross motor score (BSID III)** | | | | | | |
| Ma 2016 (C) | -0.2 | 0.2142 | 55 | 58 | 100.0% | -0.20 [-0.62, 0.22] |
| **Subtotal (95% CI)** | | | **55** | **58** | **100.0%** | **-0.20 [-0.62, 0.22]** |
| Heterogeneity: Not applicable | | | | | | |
| Test for overall effect: Z = 0.93 (P = 0.35) | | | | | | |
| **1.55.3 Motor skill (Baley)** | | | | | | |
| Faber 2005 | 1.1 | 0.4735 | 128 | 138 | 69.8% | 1.10 [0.17, 2.03] |
| Quintero 2011 | 1.2 | 0.7205 | 195 | 200 | 30.2% | 1.20 [-0.21, 2.61] |
| **Subtotal (95% CI)** | | | **323** | **338** | **100.0%** | **1.13 [0.35, 1.91]** |
| Heterogeneity: Tau² = 0.00; Chi² = 0.01, df = 1 (P = 0.91); I² = 0% | | | | | | |
| Test for overall effect: Z = 2.86 (P = 0.004) | | | | | | |

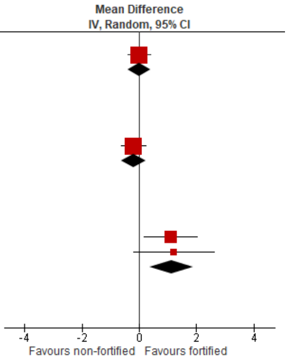


Test for subgroup differences: Chi² = 8.87, df = 2 (P = 0.01), I² = 77.5%

## 1.56. Fortified versus non-fortified complementary food. Outcome: Iron deficiency

| Study or Subgroup | log[Risk Ratio] | SE | Fortified Total | Non-fortified Total | Weight | Risk Ratio IV, Random, 95% CI |
|---|---|---|---|---|---|---|
| Ekoe 2020 (C) | -1.1202 | 0.511 | 106 | 133 | 25.1% | 0.33 [0.12, 0.89] |
| Lartey 2000 | -1.6328 | 0.5694 | 28 | 31 | 21.8% | 0.20 [0.06, 0.60] |
| Ma 2016 (C) | -0.5558 | 0.1948 | 140 | 133 | 53.1% | 0.57 [0.39, 0.84] |
| **Total (95% CI)** | | | **274** | **297** | **100.0%** | **0.39 [0.21, 0.75]** |
| Heterogeneity: Tau² = 0.16; Chi² = 3.89, df = 2 (P = 0.14); I² = 49% | | | | | | |
| Test for overall effect: Z = 2.86 (P = 0.004) | | | | | | |

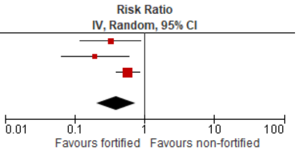


## 1.57. Fortified versus non-fortified complementary food. Outcome: Vitamin A deficiency

| Study or Subgroup | Fortified Events | Fortified Total | Non-fortified Events | Non-fortified Total | Weight | Risk Ratio M-H, Random, 95% CI |
|---|---|---|---|---|---|---|
| Lartey 2000 | 3 | 29 | 10 | 31 | 36.5% | 0.32 [0.10, 1.05] |
| Nesamvuni 2005 | 7 | 16 | 0 | 20 | 16.5% | 18.53 [1.14, 301.79] |
| Palmer 2021 | 29 | 103 | 20 | 58 | 47.0% | 0.82 [0.51, 1.31] |
| **Total (95% CI)** | | **148** | | **109** | **100.0%** | **0.97 [0.24, 3.90]** |
| Total events | 39 | | 30 | | | |
| Heterogeneity: Tau² = 1.01; Chi² = 7.61, df = 2 (P = 0.02); I² = 74% | | | | | | |
| Test for overall effect: Z = 0.04 (P = 0.97) | | | | | | |

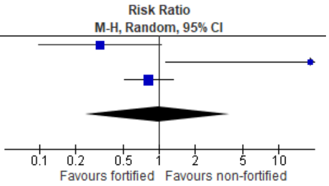


## 1.58. Fortified versus non-fortified complementary food. Outcome:  Zinc deficiency

| Study or Subgroup | Fortified Events | Fortified Total | Non-fortified Events | Non-fortified Total | Weight | Risk Ratio M-H, Random, 95% CI |
|---|---|---|---|---|---|---|
| Lartey 2000 | 3 | 30 | 1 | 31 | | 3.10 [0.34, 28.17] |

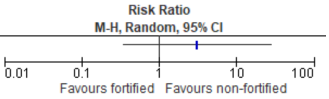

## Supplement 10. Funnel plot for the outcome haemoglobin

**Funnel plot of comparison: Fortified versus non-fortified complementary food; outcome: Haemoglobin (g/L)**

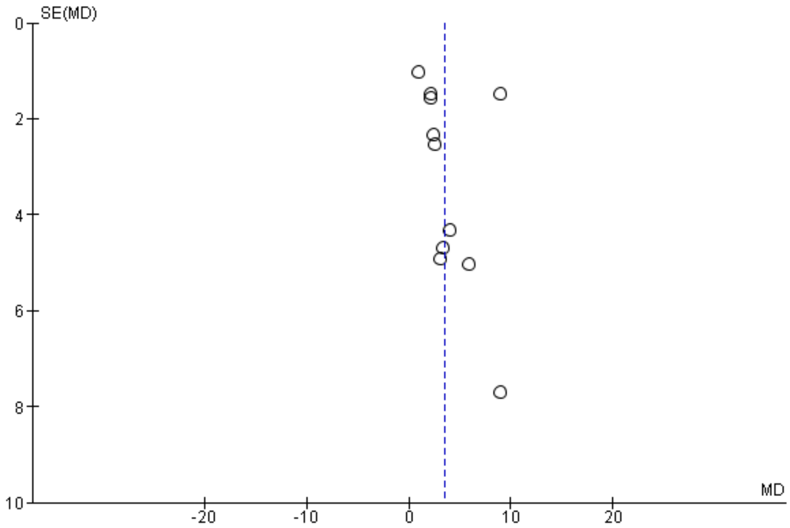

## Supplement 11. Acknowledgments

The lead author (the manuscript's guarantor) affirms that the manuscript is an honest, accurate, and transparent account of the study being reported; that no important aspects of the study have been omitted; and that any discrepancies from the study as originally planned (and, if relevant, registered) have been explained; the protocol was registered when searches were already run and piloting of the study selection process was already started; all changes were detailed to the protocol record with reasons given. WHO agreed to the publication of this systematic review in a scientific journal because it serves as a background evidence review for WHO guidelines on feeding of infants and young children 6 to 23 months of age and should therefore be available widely.
